# New Anti-Hypoxic Metabolites from Co-Culture of Marine-Derived Fungi *Aspergillus carneus* KMM 4638 and *Amphichorda* sp. KMM 4639

**DOI:** 10.3390/biom13050741

**Published:** 2023-04-25

**Authors:** Elena B. Belousova, Olesya I. Zhuravleva, Ekaterina A. Yurchenko, Galina K. Oleynikova, Alexandr S. Antonov, Natalya N. Kirichuk, Viktoria E. Chausova, Yuliya V. Khudyakova, Alexander S. Menshov, Roman S. Popov, Ekaterina S. Menchinskaya, Evgeny A. Pislyagin, Valery V. Mikhailov, Anton N. Yurchenko

**Affiliations:** 1 G.B. Elyakov Pacific Institute of Bioorganic Chemistry, Far Eastern Branch of the Russian Academy of Sciences, Prospect 100-Letiya Vladivostoka, 159, Vladivostok 690022, Russia; 2 Institute of High Technologies and Advanced Materials, Far Eastern Federal University, 10 Ajax Bay, Russky Island, Vladivostok 690922, Russia

**Keywords:** marine-derived fungi, co-culture, secondary metabolites, quinazoline alkaloid, oxyrapentyn, cytoprotection, cobalt chloride (II), antioxidants, ITS, β-tubulin, phylogeny

## Abstract

The KMM 4639 strain was identified as *Amphichorda* sp. based on two molecular genetic markers: ITS and β-tubulin regions. Chemical investigation of co-culture marine-derived fungi *Amphichorda* sp. KMM 4639 and *Aspergillus carneus* KMM 4638 led to the identification of five new quinazolinone alkaloids felicarnezolines A–E (**1**–**5**), a new highly oxygenated chromene derivative oxirapentyn M (**6**) and five previously reported related compounds. Their structures were established using spectroscopic methods and by comparison with related known compounds. The isolated compounds showed low cytotoxicity against human prostate and breast cancer cells but felicarnezoline B (**2**) protected rat cardiomyocytes H9c2 and human neuroblastoma SH-SY5Y cells against CoCl_2_-induced damage.

## 1. Introduction

An in-depth study of the organisms stored in bioresource collections can move us towards achieving one of the United Nations Sustainable Development Goals of 2015, aimed at improving human health [1]. For microorganisms (especially fungi), new results can be achieved using the OSMAC approach, including the co-cultivation of different strains. Their influence on each other can force them to produce new compounds with biological properties, which may also be of practical importance [2].

**Figure 1:**
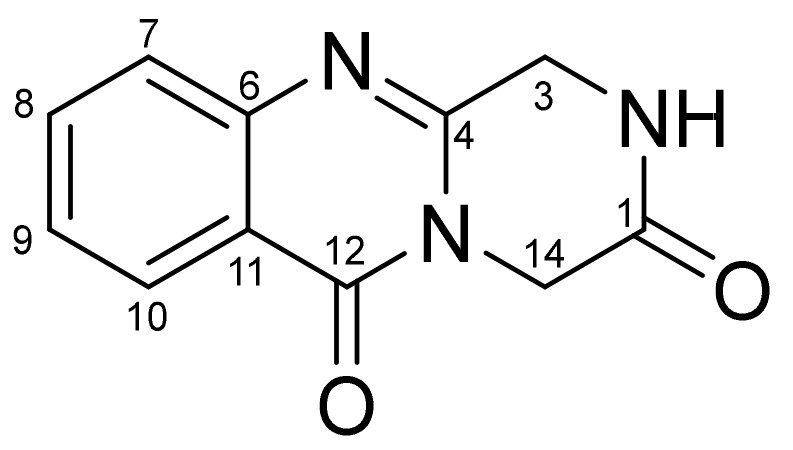
General framework of pyrazinequinazoline alkaloids.

Quinazoline alkaloids with a pyrazino[2,1-b]quinazoline-3,6-dione moiety are not rare metabolites for a number of *Aspergillus* and *Penicillium* species [3,4]. The pyrazinequinazoline core is unchanged in the majority of fungal metabolites (Figure 1). Usually, substituents originate from amino acid residues, which form the framework, and are located only at the C-3 and C-14 positions. The rare exceptions are carnequinazolines B and C from *Aspergillus carneus* Blochwitz which include hydroxy groups in the benzene ring [5] and scedapins A-E from *Scedosporium apiospermum* Sacc. ex Castell. & Chalm. without nitrogen between C-1 and C-3 in a piperazine ring [6].

Piperazine-containing quinazolines demonstrated a wide spectrum of biological activity. For instance, fumiquinazoline C was reported as an inhibitor against *α*-glucosidase [7] and fumiquinazoline Q is a promising drug candidate for cardiovascular disease treatment [8]. Scedapin C exhibited significant antiviral activity against the hepatitis C virus [6]. Polonimides A–C showed low cytotoxic activity against epithelial human cells [9] and fumigatosides E demonstrated significant antifungal activity [10].

Recently a series of twelve drimane sesquiterpenes were produced in response to the addition of a marine fungus *Amphichorda* sp. KMM 4638 (earlier identified and published as *Beauveria felina* (D.C.) J.W. Carmich. and *Isaria felina* (D.C.) Fr.) to the 7-day-old culture of a marine-derived fungus *Aspergillus carneus* KMM 4639 [11]. Further investigation of metabolites from this co-culture made it possible to isolate five new quinazolinone alkaloid felicarnezolines A–E (**1**–**5**), new highly oxygenated chromene derivative oxirapentyn M (**6**) and five known metabolites oxirapentyn B (**7**) [12], cinereain (**8**) [13], carneamide A (**9**) [5], aspergillicin A (**10**) [14], isaridin E (**11**) [15], earlier isolated from axenic cultures of *Amphichorda* sp. KMM 4638 and *Aspergillus carneus* KMM 4639.

In this work, we report on the isolation and structure elucidation of fungal co-culture-derived metabolites as well as the investigation of their cytotoxicity against human prostate PC-3 and breast cancer MCF-7 cells. Moreover, the effects of compounds at non-toxic concentrations against cobalt (II) chloride-induced damage of rat cardiomyocytes H9c2 and human neuroblastoma SH-SY5Y cells are determined.

## 2. Materials and Methods

### 2.1. General Experimental Procedures

Optical rotations were measured on a Perkin–Elmer 343 polarimeter (Perkin Elmer, Waltham, MA, USA). UV spectra were recorded on a Shimadzu UV-1601PC spectrometer (Shimadzu Corporation, Kyoto, Japan) in methanol. CD spectra were measured with a Chirascan-Plus CD spectrometer (Applied Photophysics Ltd., Leatherhead, UK) in methanol. NMR spectra were recorded in CDCl_3_, acetone-*d*_6_ and DMSO-*d*_6_, on a Bruker DPX-300 (Bruker BioSpin GmbH, Rheinstetten, Germany), a Bruker Avance III-500 (Bruker BioSpin GmbH, Rheinstetten, Germany) and a Bruker Avance III-700 (Bruker BioSpin GmbH, Rheinstetten, Germany) spectrometers, using TMS as an internal standard. HRESIMS spectra were measured on a Maxis impact mass spectrometer (Bruker Daltonics GmbH, Rheinstetten, Germany). Microscopic examination and photography of fungal cultures were performed with an Olympus CX41 microscope equipped with an Olympus SC30 digital camera. Detailed examination of ornamentation of the fungal conidia was performed by scanning electron microscopy (SEM) EVO 40.

Low-pressure liquid column chromatography was performed using Si gel (50/100 μm, Imid Ltd., Krasnodar, Russia) and Gel ODS-A (12 nm, S—75 um, YMC Co., Ishikawa, Japan). Plates precoated with Si gel (5–17 μm, 4.5 × 6.0 cm, Imid Ltd., Krasnodar, Russia) and Si gel 60 RP-18 F_254_S (20 × 20 cm, Merck KGaA, Darmstadt, Germany) were used for thin-layer chromatography. Preparative HPLC was carried out on an Agilent 1100 chromatograph (Agilent Technologies, Santa Clara, CA, USA) with an Agilent 1100 refractometer (Agilent Technologies, Santa Clara, CA, USA) and a Shimadzu LC-20 chromatograph (Shimadzu USA Manufacturing, Canby, OR, USA) with a Shimadzu RID-20A refractometer (Shimadzu Corporation, Kyoto, Japan) using YMC ODS-AM (YMC Co., Ishikawa, Japan) (5 µm, 10 × 250 mm), YMC ODS-AM (YMC Co., Ishikawa, Japan) (5 µm, 4.6 × 250 mm) and Hydro-RP (Phenomenex, Torrance, CA, USA) (4 μm, 250 × 10 mm) columns.

### 2.2. Fungal Strains

The *A. carneus* fungal strain was isolated from superficial mycobiota of the brown alga *Laminaria sachalinensis* (Miyabe) collected on Kunashir Island and was identified based on morphological evaluation by Dr. Mikhail V. Pivkin from the Pacific Institute of Bioorganic Chemistry (PIBOC). The strain is stored in the Collection of Marine Microorganisms, PIBOC, Vladivostok, Russia, under the code KMM 4638.

The *Amphichorda* sp. fungal strain was isolated from marine sediments collected at a depth of 10 m (Van Phong Bay, the South China Sea, Vietnam) during the 34th expedition of r/v “Akademik Oparin” and was identified based on morphological evaluation by Dr. Natalya N. Kirichuk from the Pacific Institute of Bioorganic Chemistry (PIBOC). The strain is stored in the Collection of Marine Microorganisms, PIBOC, Vladivostok, Russia, under the code KMM 4639.

### 2.3. DNA Extraction and Amplification

The cultures used for the molecular studies were grown on malt extract agar under 25 °C for 7 d. Genomic DNA was isolated from fungal mycelium grown on MEA (malt extract agar) at 25 °C for 7 days, using the MagJET Plant Genomic DNA Kit (Thermo Fisher Scientific, Waltham, MA, USA), according to the manufacturer’s protocol. PCR was conducted using GoTaq Flexi DNA Polymerase (Promega, Madison, WI, USA). For amplification of the internal transcribed spacer region (ITS) were used the primer pair ITSpr1 (5′-GCGTTGATATACGTCCCTGCC-3′) and ITSpr9 (5′-CCTTGGTCCGTGTTTCAAGA-3′) [16]. The reaction profile was 95 °C for 300 s, 35 cycles of 94 °C for 20 s, 60 °C for 20 s, and 72 °C for 90 s, and finally 72 °C for 300 s. For amplification of the partial beta/β-tubulin gene region the primer pair Bt-2a and Bt-2b was used [17]. The reaction profile was 95 °C for 300 s, 35 cycles of 94 °C for 20 s, 60 °C for 20 s, and 72 °C for 60 s, and finally 72 °C for 300 s. The amplified ITS and partial beta/β-tubulin genes were purified with the ExoSAP-IT™ PCR Product Cleanup Reagent (Thermo Fisher Scientific, Waltham, MA, USA). Sequencing was bidirectionally performed with the same primers on an Applied Biosystems SeqStudio Genetic Analyzer (Thermo Fisher Scientific, Waltham, MA, USA) using the Big Dye Terminator reagent kit, version 3.1. Gene sequences were deposited in GenBank under accession numbers OQ344667 for the ITS gene region and OQ418107 for the partial β-tubulin gene region (Table 1).

### 2.4. Phylogenetic Analysis

The ITS gene and partial β-tubulin gene sequences were aligned by MEGA X software version 11.0.9 [18] using the Clustal W algorithm. The available homologs were searched in the GenBank database (http://ncbi.nlm.nih.gov, accessed on 8 February 2023) using the BLASTN algorithm (http://www.ncbi.nlm.nih.gov/BLAST, accessed on 8 February 2023). Phylogenetic analysis was conducted using MEGA X software version 11.0.9 [18]. Phylogenetic trees were constructed on model-tested alignments according to the maximum likelihood algorithm. The topologies of the trees were evaluated by 1000 bootstrap replicates.

### 2.5. Cultivation of Fungus

The fungi *Aspergillus carneus* and *Amphichorda* sp. were cultivated separately at 22 °C for 7 days in Erlenmeyer flasks (500 mL) each containing 20 g of rice, 20 mg of yeast extract, 10 mg of KH_2_PO_4_, and 40 mL of natural seawater. After that, *Amphichorda* sp. mycelium was inoculated into 20 flasks with *Aspergillus carneus* culture. Then fungal cultures were co-cultivated for 14 days.

### 2.6. Extraction and Isolation

At the end of the incubation period, the mycelia and medium were homogenized and extracted with EtOAc (1 L). The obtained extract was concentrated to dryness. The residue (17.5 g) was dissolved in H_2_O−EtOH (4:1) (100 mL) and extracted with *n*-hexane (0.2 L × 3), EtOAc (0.2 L × 3) and BuOH (0.2 L × 3). After evaporation of the EtOAc layer, the residual material (5.5 g) was passed through a silica column (3 × 14 cm), which was eluted first with *n*-hexane (200 mL), then by a step gradient from 5% to 50% EtOAc in *n*-hexane (total volume 20 L). Fractions of 250 mL were collected and combined on the basis of TLC (Si gel, toluene–isopropanol 6:1 and 3:1, *v*/*v*).

The n-hexane–EtOAc (90:10) eluate (1.2 g) was separated on a Gel ODS-A column (1.5 × 8 cm), which was eluted by a step gradient from 40% to 80% CH_3_OH in H_2_O (total volume 1 L), to yield subfractions I and II. Subfraction I (60% CH_3_OH, 150 mg) was purified by RP HPLC on a Hydro-RP column eluted with CH_3_CN-H_2_O (60:40) to yield individual compound **8** (2.4 mg) and fraction I-1 (62 mg). Fraction I-1 was purified by RP HPLC on a Hydro-RP column eluted with CH_3_CN-H_2_O (40:60) to yield **1** (1.3 mg) and **2** (1.7 mg). Subfraction II (80% CH_3_OH, 53 mg) was purified by RP HPLC on a Hydro-RP column eluted with CH_3_OH-H_2_O (80:20) and then with CH_3_CN-H_2_O (70:30) to yield individual compound **5** (2.0 mg).

The n-hexane-EtOAc (80:20, 646 mg) fraction was separated on a Gel ODS-A column (1.5 × 8 cm), which was eluted by a step gradient from 40% to 80% CH_3_OH in H_2_O (total volume 1 L) to yield subfraction III. Subfraction III (60% CH_3_OH, 205 mg) was purified by RP HPLC on a Hydro-RP column eluting with CH_3_OH-H_2_O (80:20) to yield fractions III-1 (125.5 mg) and III-2 (16.2). Fraction III-1 was purified by RP HPLC on a YMC-Pack Pro C-18 column eluted with CH_3_CN-H_2_O (40:60) and then with CH_3_OH-H_2_O (10:90) to yield **3** (0.7 mg) and **4** (10.0 mg). Fraction III-2 was purified by RP HPLC on a Hydro-RP column eluted with CH_3_CN-H_2_O (55:45) to yield **6** (1.5 mg) and **7** (2.1 mg).

The n-hexane-EtOAc (70:30, 1.0 g) fraction was separated on a Gel ODS-A column (1.5 × 8 cm), which was eluted by a step gradient from 40% to 80% CH_3_OH in H_2_O (total volume 1 L) to yield subfractions IV and V. Subfraction IV (60% CH_3_OH, 282 mg) was purified by RP HPLC on a Hydro-RP column eluting with CH_3_OH-H_2_O (70:30) and then on a YMC ODS-A column eluted with CH_3_CN-H_2_O (40:60) to yield **11** (5.0 mg). Subfraction V (80% CH_3_OH, 168 mg) was purified by RP HPLC on a Hydro-RP column eluted with CH_3_CN-H_2_O (55:45) to yield **9** (16.8 mg) and **10** (17.3 mg).

### 2.7. Spectral Data

Felicarnezoline A (**1**): amorphous solids; ${\left[\alpha \right]}_{D}^{20}$ −24.7 (*c* 0.08, MeOH); CD (*c* 2.9 × 10^−4^, CH_3_OH), λ_max_ (∆ε) 193 (+22.10), 208 (−15.20), 224 (+17.49), 267 (+6.52), 307 (−6.85) nm, see Supplementary Figure S56; UV (CH_3_OH) *λ*_max_ (log *ε*) 307 (3.82), 273 (3.39), 211 (4.39) nm, see Supplementary Figure S57; ^1^H and ^13^C NMR data, see Table 1, Supplementary Figures S1–S6; HRESIMS *m*/*z* 270.0891 [M − H]^−^ (calcd. for C_14_H_12_N_3_O_3_, 270.0884, Δ −2.5 ppm), 294.0852 [M + Na]^+^ (calcd. for C_14_H_13_N_3_O_3_Na, 294.0849, Δ −1.0 ppm).

Felicarnezoline B (**2**): amorphous solids; ${\left[\alpha \right]}_{D}^{20}$ −35.4 (*c* 0.07, MeOH); CD (*c* 4.2 × 10^−4^, CH_3_OH), λ_max_ (∆ε) 196 (+14.1), 213 (−1.99), 239 (+2.78), 261 (+2.92), 287 (−2.57) nm, see Supplementary Figure S58; UV (CH_3_OH) *λ*_max_ (log *ε*) 360 (3.83), 327 (3.62), 317 (3.66), 298 (3.60), 252 (4.11), 241 (4.08), 214 (4.36) nm, see Supplementary Figure S59; ^1^H and ^13^C NMR data, see Supplementary Figures S7–S13; HRESIMS *m*/*z* 286.0842 [M − H]^−^ (calcd. for C_14_H_12_N_3_O_4_, 286.0833, Δ −3.2 ppm), 310.0802 [M + Na]^+^ (calcd. for C_14_H_13_N_3_O_4_Na, 310.0798, Δ −1.2 ppm).

Felicarnezoline C (**3**): amorphous solids; ^1^H NMR data, see Supplementary Figure S14; HRESIMS *m*/*z* 334.1530 [M + Na]^+^ (calcd. for C_18_H_21_N_3_O_2_, 334.1526, Δ −1.2 ppm).

Felicarnezoline D (**4**): amorphous solids; ${\left[\alpha \right]}_{D}^{20}$ −20.0 (c 0.07, MeOH); CD (c 8.0 × 10^−7^, CH_3_OH), λ_max_ (∆ε) 197 (+30.1), 230 (−1.94), 259 (+2.05), 278 (−0.32), 304 (+4.12) nm, see Supplementary Figure S60; UV (CH_3_OH) λ_max_ (log ε) 343 (4.21), 234 (4.54), 214 (4.62), 197 (4.60), nm, see Supplementary Figure S61; ^1^H NMR data, see Supplementary Figure S15; HRESIMS *m*/*z* 350.1471 [M + Na]^+^ (calcd. for C_18_H_23_N_3_O_3_Na, 350.1475, Δ 1.1 ppm).

Felicarnezoline E (**5**): amorphous solids; ^1^H NMR data, see Supplementary Figure S16; HRESIMS *m*/*z* 352.1628 [M + Na]^+^ (calcd. for C_18_H_23_N_3_O_3_Na, 352.1632, Δ 1.1 ppm).

Oxirapentyn M (**6**): amorphous solids; ${\left[\alpha \right]}_{D}^{20}$ −71.8° (*c* 0.04, MeOH); CD (*c* 2.0 × 10^−3^, CH_3_OH), λ_max_ (∆ε) 220 (−0.23), 295 (−0.04) nm, see Supplementary Figure S62; UV (CH_3_OH) *λ*_max_ (log *ε*) 225 (3.74) nm, see Supplementary Figure S63; ^1^H and ^13^C NMR data, see Table 2, Supplementary Figures S17–S23; HRESIMS *m*/*z* 335.1492 [M − H]^−^ (calcd. for C_18_H_23_O_6_, 335.1492, Δ 2.5 ppm), 359.1453 [M + Na]^+^ (calcd. for C_18_H_24_O_6_Na, 359.1453, Δ 3.3 ppm).

### 2.8. Stereo Configuration Analysis of Amino Acids in Compounds ***1***, ***2***, ***5*** and ***6***

The compounds (0.6 mg of each) were placed in glass ampoules and dissolved in 6 N HCl (1.2 mL). Solutions in ampoules were frozen in liquid nitrogen, then vacuumed, sealed, and heated at 105 °C for 24 h. Then, the cooled reaction mixture was diluted with distilled water and concentrated in vacuo. The obtained hydrolysates of compounds **1**–**5** and standard amino acid Val of the *L*- and *D*-configurations (0.2 mg each) were dissolved in 0.1 mL of distilled water, then 0.4 mL of 1M NaHCO_3_ and 0.2 mL of a 1% solution of Marfey’s reagent in acetone were added. The reaction mixtures were kept at 37 °C for 75 min and 0.05 mL of 1M HCl was added. Then, obtained *L*-FDDA derivatives were analyzed by HPLC-UV in a gradient from 25% to 65% of MeCN in H_2_O (0.1% TFA) over 40 min at 20 °C using the YMC C-18 Pro column.

### 2.9. Cell Lines and Culture Conditions

The human prostate cancer PC-3, human breast cancer MCF-7, and human neuroblastoma SH-SY5Y cells were purchased from ATCC (Manassas, VA, USA). Rat cardiomyocyte H9c2 cells were kindly provided by Prof. Dr. Gunhild von Amsberg from Martini-Klinik Prostate Cancer Center, University Hospital Hamburg-Eppendorf, Hamburg, Germany.

PC-3, MCF-7, SH-SY5Y and H9c2 cells were cultured in DMEM medium (Biolot, St. Petersburg, Russia) containing 10% fetal bovine serum (Biolot, St. Petersburg, Russia) and 1% penicillin/streptomycin (Biolot, St. Petersburg, Russia) at 37 °C in a humidified atmosphere with 5% (*v*/*v*) CO_2_.

Initially, the cells were incubated in culture flasks until subconfluent (~80%). For testing, the cells were seeded at concentrations of 5 × 10^3^ cells/well (PC-3, MCF-7, SH-SY5Y cells) or 3 × 10^3^ cells/well (H9c2 cells), and experiments were started after 24 h.

### 2.10. In Vitro MTT-Based Cytotoxicity Assay

The *in vitro* cytotoxicity of individual substances was determined by the MTT method (3-(4,5-dimethylthiazol-2-yl)-2,5-diphenyltetrazolium bromide), according to the manufacturer’s instructions (Sigma-Aldrich, St. Louis, MO, USA).

Investigated compounds were dissolved in DMSO at a concentration of 10 mM. This solution was used to obtain the required concentration of compounds in the cell suspension so that the concentration of DMSO in the cell suspension did not exceed 1%.

The cells were treated with the investigated compounds for 24 h or 48 h, and MTT reagent was added to each well of the plate. The vehicle with DMSO at a concentration of 1% was used as a control. The absorbance of formed formazan was measured at λ = 570 nm using a Multiskan FC microplate photometer (Thermo Scientific, Waltham, MA, USA) and expressed in optical units (o.u.). The results are presented as % of viable cells relative to the vehicle, and 50% inhibition concentration of cell viability (IC_50_) was calculated.

### 2.11. CoCl_2_-Mimic Hypoxia Modeling

The SH-SY5Y and H9c2 cells were treated with a dH_2_O-solution of CoCl_2_ at a concentration of 500 µM for 1 h. Then, compounds at a concentration of 10 µM were added for 23 h (SH-SY5Y cells) or 47 h (H9c2 cells). The viability of the SH-SY5Y and H9c2 cells was measured by an MTT assay as described above.

### 2.12. Reactive Oxygen Species (ROS) Level Assay

The SH-SY5Y and H9c2 cells were treated with a dH_2_O-solution of CoCl_2_ at a concentration of 500 µM for 1 h. Then, compounds at a concentration of 10 µM were added for 3 h. The non-treated cells were used as a control. The 20 μL of 2,7-dichlorodihydrofluorescein diacetate solution (H_2_DCFDA, Molecular Probes, Eugene, OR, USA) was added to each well (10 μM, final concentration) and the plate was incubated for an additional 10 min at 37 °C. The intensity of dichlorofluorescein fluorescence was measured with a PHERAstar FS plate reader (BMG Labtech, Ortenberg, Germany) at λ_ex_ = 485 nm and λ_em_ = 518 nm. The data were processed by MARS Data Analysis v. 3.01R2 (BMG Labtech, Germany). The results were presented as relative fluorescence units.

### 2.13. Superoxide Dismutase Activity Detection

The SH-SY5Y and H9c2 cells were seeded in 6-well plates for 24 h. The dH_2_O-solution of CoCl_2_ at a concentration of 500 µM was added for 1 h and then compounds at a concentration of 10 µM were added for 3 h. The non-treated cells were used as a control.

The cells were washed with PBS twice, collected in 1.5 mL tubes and lysed with RIPA buffer (Sigma-Aldrich, St. Louis, MO, USA). Then the cells were centrifuged at 14,000 per min (Eppendorf, Framingham, MA, USA) and the supernatant was used. The reaction mixture contains 1 mL of 26.9 µM EDTA, 1 mL of 4.04 µM tetrazolium nitroblue tetrazolium chloride (Dia-M, Novosibirsk, Russia), 1 mL of 65 µM 5-methylphenazinium methyl sulfate (Dia-M, Novosibirsk, Russia) and 26 mL PBS. The supernatant, 1 mM NADH and reaction mixture were added to a 96-well plate as 1:1:28 for 10 min in dark and the reaction was stopped by light. The mixture with RIPA buffer instead of supernatant was used as a control. The mixture without NADH was used as a background. The optical density of reaction mixtures was measured at λ = 540 nm using a Multiskan FC microplate photometer (Thermo Scientific, Waltham, MA, USA).

The total protein concentration of each probe was measured by Bradford assay.

The activity of superoxide dismutase (A_sod_, u/mg) was mind as inhibition of nitroblue tetrazolium reduction (T, %):

T (%) = (OD control − OD test)/OD control × 100%

and calculated per total protein content.

The total protein content was measured by Bradford assay. Bovine serum albumin was used to build a calibration curve.

### 2.14. Statistical Data Evaluation

All the data were obtained in three independent replicates, and the calculated values were expressed as a mean ± standard error mean (SEM). A Student’s *t*-test was performed using SigmaPlot 14.0 (Systat Software Inc., San Jose, CA, USA) to determine the statistical significance. The differences were considered statistically significant at *p <* 0.05.

## 3. Results

### 3.1. Molecular Identification of the KMM 4639 Fungal Strain

**Figure 2:**
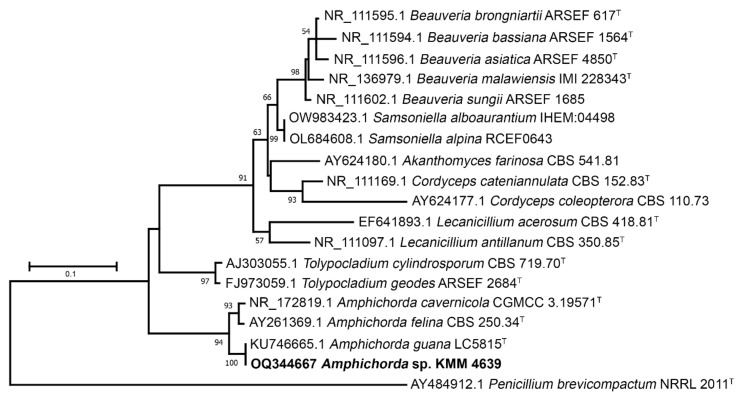
ML tree based on ITS gene sequences showing phylogenetic position of the strain KMM 4639 within family *Cordycipitaceae*. Bootstrap values (%) of 1000 replications. Nodes with confidence values greater than 50% are indicated. The scale bars represent 0.1 substitutions per site. ^T^—ex-type strain.

**Figure 3:**
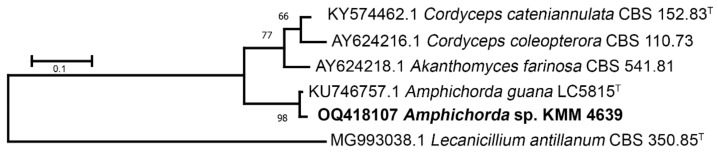
ML tree based on partial β-tubulin gene sequences showing phylogenetic position of the strain KMM 4639. Bootstrap values (%) of 1000 replications. Nodes with confidence values greater than 50% are indicated. The scale bars represent 0.1 substitutions per site. ^T^—ex-type strain.

The KMM 4639 strain was identified using two molecular markers: ITS and β-tubulin regions. A 1300 bp fragment of the ITS gene region and a 350 bp fragment of the β-tubulin gene was successfully amplified. BLAST search results indicated that the sequences were 98% (for β-tubulin) and 100% (for ITS) identical to the sequences of the ex-type strain *Amphichorda guana* Z.F. Zhang, F. Liu & L. Cai (CGMCC 3.17908). Phylogenetic ML trees constructed on the basis of the ITS gene (Figure 2) and partial β-tubulin gene sequences (Figure 3) clearly showed that the strain KMM 4639 clusters with ex-type strain *Amphichorda guana* CGMCC 3.17908 within the family *Cordycipitaceae*.

Within these studies, the strain KMM 4639 was identified as *Amphichorda* sp. Given the percentage of similarity in tubulin, it is necessary to use additional molecular markers to confirm the species affiliation of the strain.

### 3.2. Isolated Compouns from Co-Culture

**Figure 4:**
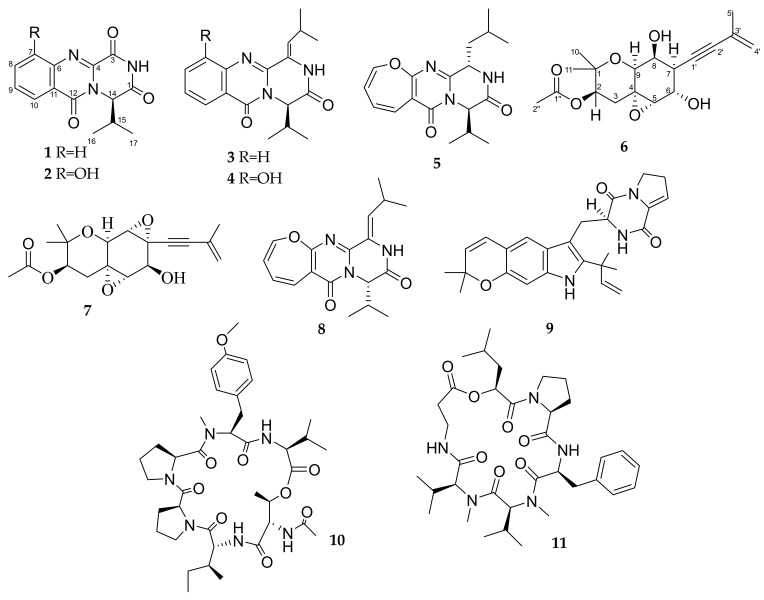
Chemical structures of **1**–**11**.

The fungi *Aspergillus carneus* and *Amphichorda* sp. were cultivated separately for 7 days in a solid rice medium. After that, *Amphichorda* sp. mycelium was inoculated into flasks with *Aspergillus carneus* culture. Then fungal cultures were co-cultivated for 14 days. The EtOAc extract of the mycelium of co-culture was purified by a combination of Si gel and ODS-AM column chromatography and reversed phase HPLC to yield compounds **1**–**12** (Figure 4).

### 3.3. Structure Characterization of New Compounds

The molecular formula of **1** was determined to be C_14_H_13_N_3_O_3_ by an HRESIMS peak at *m*/*z* 272.1034 [M + H]^+^ and was in accordance with ^13^C NMR data. The ^1^H and ^13^C NMR (Table 1), DEPT and HSQC spectra showed the signals of one amide proton (δ_H_ 8.48), 1,2-disubstitute benzene-ring (δ_H_ 7.69, 7.91, 8.06 and 8.36), two methyl (δ_H_ 0.92 and 1.23) and two methine (δ_H_ 2.47 and 5.57) groups, three *sp*^2^-quaternary carbon signals (δ_C_ 121.9, 139.2 and 146.0) along with three amide carbonyls (δ_C_ 156.5, 159.7 and 165.2).

The chemical shift values of C-1, C-4–C-14 carbon atoms in the ^13^C NMR spectrum closely resemble those of carnequinazoline A [5] indicating a quinazoline moiety in **1**. The correlations H-14/H-15/H_3_-16(H_3_-17) observed in the ^1^H-^1^H COSY spectrum and HMBC correlations from H-16 (δ_H_ 0.92) to C-14 (*δ*_C_ 61.7) and from H-14 (δ_H_ 5.57) to C-1 (δ_C_ 165.3), C-4 (δ_C_ 139.2), C-12 (δ_C_ 159.7), C-15 (δ_C_ 33.7) and C-17 (δ_C_ 19.0) revealed the location of an isopropyl group at C-14 in **1**. The presence in the ^13^C NMR spectrum of compound **1** of an additional signal of the amide carbon atom and the absence of signals of the 2-methylpropylidene group compared to carnequinazoline A suggested that this side group at C-3 was oxidized to carbonyl, which corresponds to the molecular formula of the compound.

The absolute configurations of C-14 stereocenter in **1** were established by Marfey’s method [19] as *R*. Analysis of the L-FDAA derivative of the amino acid residue obtained by acid hydrolysis of compound **1** showed it to be derivative of D-Val standard sample (Figures S38 and S39, Supplementary Material). Compound **1** was named felicarnezoline A.

**Figure 5:**
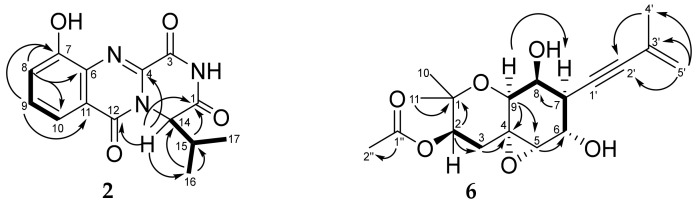
Key COSY (bold lines) and HMBC (arrows) correlations of **2** and **6**.

The molecular formula of **2** was determined to be C_14_H_13_N_3_O_4_ from an HRESIMS peak at *m*/*z* 288.0983 [M + H]^+^ and was in accordance with ^13^C NMR data (Table 2). The ^1^H and ^13^C NMR data for **2** were in good agreement with those for felicarnezoline A (**1**) with the exception of proton and carbon signals of the benzene ring. The molecular mass difference of 16 mass units between **1** and **2**, characteristic of the 1,2,3-trisubstituted benzene ring proton multiplicity and HMBC correlations (Figure 5) from H-8 (δ_H_ 7.40) to C-6 (δ_C_ 134.4), C-7 (δ_C_ 153.6) and C-10 (δ_C_ 117.7), from H-9 (δ_H_ 7.58) to C-6, C-7 and C-11 (δ_C_ 121.9) indicated the location of the hydroxy group at C-7.

The absolute configurations of C-14 stereocenter in **2** were established by Marfey’s method as *R*. Analysis of *L*-FDAA derivative of the amino acid residue obtained by acid hydrolysis of compound **1** showed it to be derivative of *D*-Val standard sample (Figures S40 and S41, Supplementary Material). Compound **2** was named felicarnezoline B.

The NMR data of **3**, **4** and **5** corresponded to the signals of known carnequinazolines A, B [5] and dihydrocinereain [20], respectively. The presence of the *D*-valine in the structures of compounds **1** and **2** lets us suggest this amino acid in structures **3**, **4**, and **5** instead of *L*-valine in known related compounds. To prove the absolute stereochemistry of **3**, **4**, and **5** by Marfey’s method their acid hydrolysis was carried out and *L*-FDAA derivatives of the amino acids were obtained. Thus, the presence of *D*-valine in structures **3**, **4**, and **5** was established (Figures S42–S47, Supplementary Material).

We hypothesize that D-valine may also be present in compounds **8**, **10** and **11** instead of L-valine, as previously described for cinereain, aspergillicin A [14] and isaridin E. However, due to the insufficient amount of these compounds, the use of Marfey’s method turned out to be impossible to determine the configuration of the amino acids included in the structures of these compounds.

The HRESIMS of **6** showed the quasimolecular ion at *m*/*z* 359.1453 [M + Na]^+^. These data, coupled with ^13^C NMR spectral data (DEPT), established the molecular formula of all compounds as C_18_H_24_O_6_. A close inspection of ^1^H and ^13^C NMR data of **6** by DEPT and HSQC (Table 3) indicated the presence of four methyl (δ_H_ 2.11, δ_C_ 20.8, δ_H_ 1.40, δ_C_ 22.0 и δ_H_ 1.90, δ_C_ 23.7, δ_H_ 1.19, δ_C_ 25.4), two methylene (δ_H_ 1.57, 2.55 δ_C_ 32.3, δ_H_ 5.22, 5.30 δ_C_ 122.1) and six methine groups (δ_H_ 2.90, δ_C_ 37.5, δ_H_ 3.08, δ_C_ 64.3, δ_H_ 4.17, δ_C_ 67.8, δ_H_ 4.09, δ_C_ 68.2, δ_H_ 3.84, δ_C_ 72.4, δ_H_ 4.90, δ_C_ 73.8), five of them oxygen-bearing, one *sp*^2^- (δ_C_ 126.5) and three *sp*^3^ (*δ*_C_ 76.3, 88.9 and 90.3) oxygen-bearing carbons, three *sp*^2^ (*δ*_C_ 106.0, 122.7, 145.4) and two *sp*^3^ (δ_C_ 57.9, 74.3) quaternary carbons along with one carboxy group (δ_C_ 170.3).

The general features of ^1^H and ^13^C NMR spectra of **6** indicated that the compound belongs to the family of oxirapentyns, highly oxidized polyketides previously isolated from *Amphichorda* sp. KMM 4639.

A comparison of the NMR spectra of **6** with those of oxirapentyn F [21] revealed some similarities, including three methyl, two methylene and one acetoxy groups. The HMBC correlations (Figure 5) from H-4′a (δ_H_ 5.22) to C-2′ (δ_C_ 84.9) and C-5′ (δ_C_ 23.7), from H_3_-5′ (δ_H_ 1.90) to C-2′, C-3′ (δ_C_ 126.5) and C-4′ (δ_C_ 122.1), from H-6 (δ_H_ 4.17) to C-1′ (δ_C_ 87.1) revealed the presence of a 3-methyl-3-buten-1-ynyl side chain in **6**. The ^13^C NMR spectrum of **6** showed signals at δ_C_ 57.9 and 64.3, which are the characteristic signals of epoxide carbons. The ^1^H NMR spectrum indicated the presence of one epoxy proton at δ_H_ 3.08. These data together with the HMBC correlations from H_2_-3 (δ_H_ 1.57, 2.55) and H-9 (δ_H_ 4.09) to C-4 (δ_C_ 57.9) and C-5 (δ_C_ 64.3) indicated the location of an epoxy group at C-4–C-5. The ^1^H–^1^H COSY correlations of H-6(OH)/H-7/H-8(OH)/H-9 indicated the presence of hydroxy groups at C-6 and C-8 in **6**.

The key correlations observed in ROESY spectrum H-2/H-3α, H-5/H-3β and H-9/H_3_-11, H-3α and ^1^H-^1^H coupling constants suggested the α-orientation of epoxide and hydroxy group at C-6 and β-orientation of acetate and hydroxy group at C-8 in **6**. The compound was named oxirapentyn M. Due to the shortage of the pure sample, Mosher’s method was not feasible to study the absolute configuration of oxirapentyn M.

### 3.4. Cytotoxic Activity of Isolated Compounds

The cytotoxic effects of the compounds **1**–**5** and **7**–**11** against human prostate cancer PC-3, breast cancer MCF-7 and neuroblastoma SH-SY5Y cells as well as rat normal cardiomyocytes H9c2 were investigated and presented in Table 4. Compound **6** was isolated in an insufficient amount and was not tested.

The investigated compounds did not show significant cytotoxic activity toward both cancer and normal cells. Only compound **4** had weak toxicity for MCF-7 and SH-SY5Y cells and prolonged treatment of the cells with this one did not result in stronger cytotoxicity.

### 3.5. Effects of Isolated Compounds in CoCl_2_-Mimic Hypoxia

The cytoprotective effects of isolated compounds were investigated in cobalt (II) chloride (CoCl_2_)-mimic hypoxic model using neuronal SH-SY5Y cells and normal cardiomyocytes H9c2.

**Figure 6:**
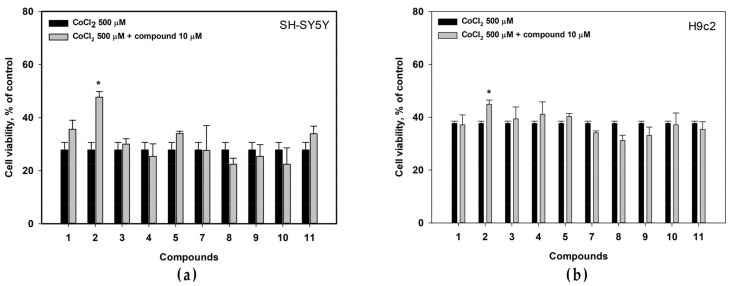
Influence of the compounds on viability of CoCl_2_-treated (**a**) SH-SY5Y and (**b**) H9c2 cells. The viability of nontreated (control) cells were 100.3 ± 1.2%. All experiments were carried out in three independent replicates and the data are presented as a mean ± SEM. * indicated the statistically significant differences (*p* < 0.05).

The viability of CoCl_2_-treated SH-SY5Y cells dramatically decreased and was only 27.8% in comparison with non-treated cells (Figure 6a). Felicarnezoline B (**2**) statistically significantly increased the viability of CoCl_2_-treated SH-SY5Y cells by 72.6%. The effect of compounds **1**, **5** and **11** was observed but was not statistically significant.

The viability of CoCl_2_-treated cardiomyocytes H9c2 was 37.8% only in comparison with non-treated cells (Figure 6b). Felicarnezoline B (**2**) statistically significantly increased the viability of CoCl_2_-treated H9c2 cells by 19.1%. The effect of other compounds was not statistically significant.

**Figure 7:**
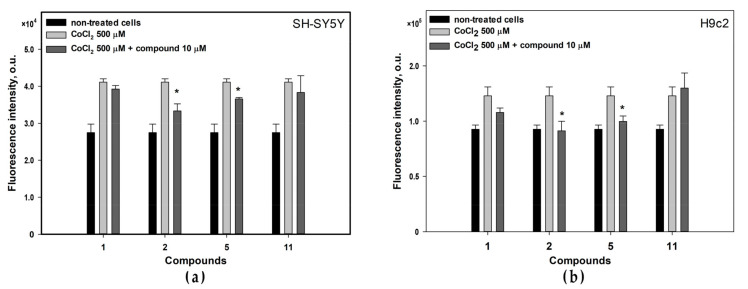
Influence of the compounds on ROS level in CoCl_2_-treated (**a**) SH-SY5Y and (**b**) H9c2 cells. All experiments were carried out in three independent replicates and the data are presented as a mean ± SEM. * indicated the statistically significant differences (*p* < 0.05).

Treating with CoCl_2_ induces oxidative stress in cells and the protective effect of compounds may be caused by their antioxidant properties. To detect this, the effects of active compounds **1**, **2**, **5** and **11** on reactive oxygen species (ROS) level in CoCl_2_-treated cells was investigated (Figure 7).

The ROS level in SH-SY5Y cells treated with CoCl_2_ for 4 h was increased by 49.6% (Figure 7a). Compounds **2** and **5** statistically significantly decreased the ROS level in these cells by 18.8% and 11.0%, respectively. The ROS level in CoCl2-treated H9c2 cells increases by 32.6% after 4 h of treatment (Figure 7b). Compounds **2** and **5** statistically diminished the ROS level in these cells by 25.7% and 18.6%, respectively.

**Figure 8:**
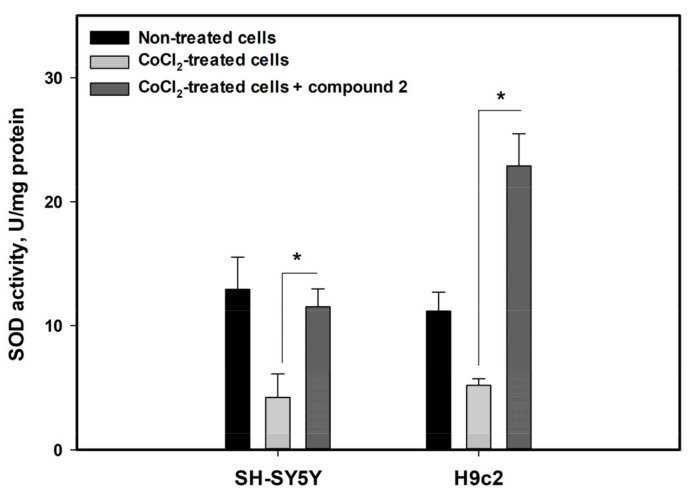
Influence of felicarnezoline B (**2**) on superoxide dismutase activity in CoCl_2_-treated cells. All experiments were carried out in three independent replicates and the data are presented as a mean ± SEM. * indicated the statistically significant differences.

In addition, the effect of felicarnezoline B (**2**) on the activity of the superoxide dismutase (SOD) antioxidant enzyme was investigated to verify its influence on the intracellular antioxidant system in CoCl_2_-treated cells (Figure 8).

The activity of SOD in SH-SY5Y and H9c2 cells treated with CoCl_2_ for 4 h was dramatically diminished by 67.4% and 53.5%, respectively. The incubation with felicarnezoline B (**2**) resulted in a significant increase in SOD activity in both cases.

## 4. Discussions

**Figure 9:**
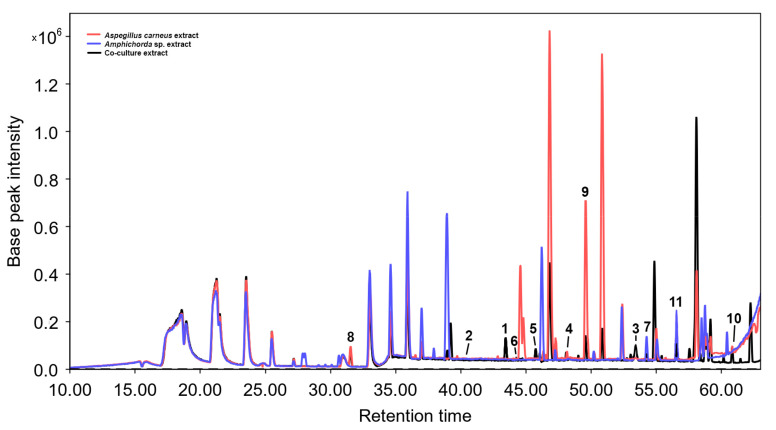
HPLC MS chromatogram of extracts of *Aspergillus carneus* and *Amphichorda* sp. monocultures as well as their co-culture. The numbers correspond to the numbers of the isolated compounds.

Previously, we described the isolation of drimane sesquiterpenoids from a co-culture of *Aspergillus carneus* KMM 4638 and *Amphichorda* sp. KMM 4639, some of which had a pronounced cytotoxic activity and inhibited the cell cycle of human breast cancer MCF-7 cells [11]. Now we have managed to isolate new substances with cytoprotective properties, and it is obvious that co-cultivation led to their production (Table 5, Figure 9).

Earlier, carnequinazolines A-C were isolated from *Aspergillus carneus* KMM 4638 [5]. These compounds have *L*-valine, at the same time new alkaloids have *D*-valine in their structures. In addition, felicarnezolines A (**1**) and B (**2**) contain a 1,3-diketopiperazine moiety in their structure, which is unique for quinazoline alkaloids and may be biosynthesized by the action of oxidative enzymes of *Amphichorda* sp.

It should be noted that “undetected” compounds may still be present in the extracts in amounts beyond detection. This is partly confirmed by the results of HPLC-MS (Figure 9). Moreover, a detailed evaluation of the obtained results of Marfey’s analysis revealed that we cannot exactly prove the absence of *L*-valine impurity in the hydrolyzate of samples of compounds **1**–**4**. The chromatograms of these samples show small peaks with an RT close to *L*-valine. The ratio of peak areas assigned to *D*- and *L*-valine is approximately 4:1. Thus, we can deal with a mixture of enantiomers in the case of these compounds. At the same time, the chromatogram of the FDAA-derivatives of the hydrolyzate of compound **5** does not contain visible peaks with the RT of *L*-valine; therefore, we can accurately state the absence of an impurity of the “original” stereoisomer (dihydrocinereain).

Stereo conversion of amino acids is not unusual in microorganisms, including fungi. As a rule, this occurs either under the action of amino acid racemase or amino acid oxidase, followed by reductive amination [22]. For example, *L*-alanine racemase has been described in the fungus *Amphichorda felina* (D.C.) Fr., which produces cyclosporin C (contains *D*-alanine in the structure) [23]. Unfortunately, no relevant data about fungal valine racemase or valine oxidase could be found. Finally, epimerization of L-valine can occur as part of a dipeptide precursor of **1**–**5**, similar to the proposed mechanism for the penicillin producing *Acremonium chrysogenum* (Thirum. & Sukapure) W. Gams [24]. Thus, the presence of *D*-valine in compounds **1**–**5** instead of *L*-valine in related compounds from *Aspergillus carneus* KMM 4638 monoculture is most likely the result of the action of the fungus *Amphichorda* sp. KMM 4639. However, the proof of this assumption should be the subject of further detailed studies.

The KMM 4639 strain of the *Amphichorda* sp. fungus is very interesting due to the high production of oxidated secondary metabolites [21] and the co-cultivation of various fungal strain stable resulted in the isolation of new biologically active compounds.

It should be noted that the earlier fungal strain KMM 4639 was misidentified using morphological features and published as *Isaria felina* and *Beauveria felina*. These species as well as the genus *Amphichorda* belong to the *Cordycipitaceae* family and may have similar metabolism [25,26,27]. So, a number of depsipeptides isolated earlier by us from the KMM 4639 fungal strain were isolated from *Amphichorda guana* fungus [25] as well as *Beauveria felina* [27] and *Isaria* sp. [15]. However, the ability of this strain to produce highly oxygenated metabolites is not typical for this group of fungi, which requires further research for a more complete realization of its biotechnological potential.

Under the highly competitive conditions of densely populated microbial communities, marine fungi are forced to produce secondary metabolites both for aggression against competitors and for defense against them [28]. The first purpose is served by various substances with cytotoxic activity, the second one is provided, first of all, by antioxidants. However, in most cases, conclusions about antioxidant properties are made by the presence of DPPH-scavenging activity in a cell-free test [29]. Obviously, the manifestation of these radical-scavenging properties in a living system can be very limited and the investigation of the antioxidant properties of compounds in *in vitro* conditions is necessary. Earlier the antioxidant effects of fungal echinulin-related indoldiketopiperazine and desoxyisoaustamide alkaloids were investigated in cell models of oxidative stress induced with neurotoxins [30,31]. Now we used the cobalt (II) chloride solution for modeling hypoxia in two different cell lines.

The CoCl_2_-induced hypoxia-mimic *in vitro* model is widely used to search for cytoprotective compounds, despite some limitations [32]. Similar to real oxygen deprivation, treating with CoCl_2_ induces oxidative stress and mitochondrial DNA damage [33]. Various investigations found that chronic low oxygen level or hypoxia/reoxygenation conditions results in a significant increase in ROS level in H9c2 cells [34,35]. It is considered that the decrease in the activity of the mitochondrial electron transport chain during hypoxia slows down electron transfer, increasing the likelihood of an undesired electron transition to molecular oxygen, which produces a highly efficient reactive superoxide anion (O^2−^) [36]. However, some literature data confirm that CoCl_2_ caused an increase in ROS level in H9c2 cells while 1% O_2_ hypoxia resulted in a decrease in ROS level, but the SOD activity was decreased in both cases [37]. In addition, the activation of cellular antioxidant machinery can protect the cell from CoCl_2_ -caused damage similar to oxygen deprivation induced cell death [38,39].

In our experiments, the CoCl_2_ treatment decreases human neuroblastoma SH-SY5Y cells and rat H9c2 cardiomyocytes viability and was accompanied by diminished SOD activity and increased intracellular ROS level. The cytoprotective effect of the new alkaloid felicarnezoline B against CoCl_2_-induced damage is obviously related to SOD activity enhancement. SOD is one of the components of the first line of the antioxidant defense system [40] and may be induced via Keap-1/Nrf2- or NF-κB-dependent pathways as well as more specifically the specificity protein (Sp)-1, CCAAT-Enhancer-Binding Proteins (C/EBP), and the activator proteins (AP)-1 and-2, which exert similar effects on the regulation of *SOD* genes [41]. So, the influence of felicarnezoline B (**2**) on the cellular antioxidant system in hypoxia-mimic conditions is unknown now and is interesting for future investigation. 

## 5. Conclusions

As a result of the mixed cultivation of two microfilamentous fungi, *Aspergillus carneus* KMM 4638 and *Amphichorda* sp. KMM 4639, five new alkaloids and one new chromene derivative were obtained. Felicarnezoline B has shown a good protective effect in hypoxia-mimic conditions via antioxidant pathways.

## Figures and Tables

**Table 1:** Strains used in phylogenetic analysis and GenBank accession numbers of ITS and β-tubulin molecular data.

Taxon	Collection Number	GenBank Accession Numbers	
		ITS	β-Tubulin
*Akanthomyces farinosa*	CBS 541.81	AY624180	AY624218
*Amphichorda cavernicola*	CGMCC 3.19571^T^	NR 172819	‒
*Amphichorda guana*	CGMCC 3.17908^T^	KU746665	KU746757
*Amphichorda felina*	CBS 250.34^T^	AY261369	‒
***Amphichorda*** **sp.**	**KMM 4639**	**OQ344667**	**OQ418107**
*Tolypocladium cylindrosporum*	CBS 719.70^T^	AJ303055	‒
*Tolypocladium geodes*	ARSEF 2684^T^	FJ973059	‒
*Beauveria malawiensis*	IMI 228343^T^	NR_136979	‒
*Beauveria sungii*	ARSEF 1685^T^	NR_111602	‒
*Beauveria asiatica*	ARSEF 4850^T^	NR_111596	‒
*Beauveria brongniartii*	ARSEF 617^T^	NR_111595	‒
*Beauveria bassiana*	ARSEF 1564^T^	NR_111594	‒
*Cordyceps cateniannulata*	CBS 152.83^T^	NR_111169	KY574462
*Cordyceps coleopterora*	CBS 110.73	AY624177	AY624216
*Lecanicillium acerosum*	CBS 418.81^T^	EF641893	‒
*Lecanicillium antillanum*	CBS 350.85^T^	NR_111097	MG993038
*Penicillium brevicompactum*	NRRL 2011^T^	AY484912	DQ645784
*Samsoniella alboaurantium*	IHEM:04498	OW983423	‒
*Samsoniella alpina*	RCEF0643	OL684608	‒

^T^—ex-type strain.

**Table 2:** ^1^H and ^13^C NMR data for compounds **1** and **2**.

Position	1 ^a^		2 ^b^	
	δ_C_, Type	δ_H_, Mult, *J* in Hz	δ_C_, Type	δ_H_, Mult, *J* in Hz
1	165.2, C		165.1, C	
2		8.48, brs		8.54, s
3	156.5, C		156.0, C	
4	139.2, C		138.0, C	
5				
6	146.0, C		134.3, C	
7	129.8, CH	8.06, d (8.2)	153.6, C	
8	135.5, CH	7.91, td (8.1, 1.6)	119.1, CH	7.40, d (8.0)
9	130.1, CH	7.69, td (7.9, 1.1)	131.5, CH	7.58, t (8.0)
10	127.2, CH	8.36, dd (8.0, 1.2)	117.7, CH	7.81, d (8.0)
11	121.9, C		121.9, C	
12	159.7, C		159.3, C	
13				
14	61.7, CH	5.57, dd (4.2, 0.8)	61.8, CH	5.55, d (4.3)
15	33.7, CH	2.27, m	33.6, CH	2.46, m
16	16.9, CH_3_	0.92, d (6.9)	16.8, CH_3_	0.93, d (6.9)
17	19.0, CH_3_	1.23, d (6.9)	19.0, CH_3_	1.23, d (6.9)

^a^ chemical shifts were measured in CDCl_3_ at 500.13 MHz for ^1^H and 125.77 MHz for ^13^C. ^b^ chemical shifts were measured in CDCl_3_ at 700.13 MHz for ^1^H and 176.04 MHz for ^13^C.

**Table 3:** ^1^H and ^13^C NMR data for compound **6**.

Position	6 ^a^	
	δ_C_, Type	δ_H_, Mult, *J* in Hz
1	74.3, C	
2	73.8, CH	4.90 t (3.2)
3	32.3, CH_2_	α: 2.55, dd (14.3, 2.8) β: 1.57, dd (14.3, 3.4)
4	57.9, C	
5	64.3, CH	3.08, s
6	67.8, CH	4.17, dd (9.9, 3.2)
7	37.5, CH	2.90, dd (10.2, 2.0)
8	72.4, CH	3.84, dt (10.4, 2.5)
9	68.2, CH	4.09, d (2.9)
10	25.4, CH_3_	1.19, s
11	22.0, CH_3_	1.40, s
1′	87.1, C	
2′	84.9, C	
3′	126.5, C	
4′	122.1, CH_2_	5.30, brs 5.22, m
5′	23.7, CH_3_	1.90, brs
1″	170.3, C	
2″	20.8, CH_3_	2.11, s
6-OH	OH	2.21, d (3.6)
8-OH	OH	2.31, d (10.4)

^a^ chemical shifts were measured in CDCl_3_ at 500.13 MHz for ^1^H and 125.77 MHz for ^13^C.

**Table 4:** The cytotoxic activity of compounds **1**–**5** and **7**–**11**.

Compound	Cell Lines			
	PC-3	MCF-7	SH-SY5Y	H9c2
	IC_50_, µM			
**1**	>100	>100	>100	>100
**2**	>100	>100	>100	>100
**3**	>100	92.5 ± 3.1	>100	>100
**4**	>100	68.7 ± 1.6	72.9 ± 2.8	>100
**5**	83.8 ± 5.5	86.3 ± 2.3	>100	>100
**7**	>100	>100	>100	>100
**8**	>100	>100	93.8 ± 3.8	>100
**9**	>100	>100	>100	>100
**10**	>100	>100	>100	>100
**11**	>100	>100	>100	>100

The concentration of half maximum effect (IC_50_) is presented as a mean ± standard error of mean (SEM). All experiments were carried out in three independent replicates.

**Table 5:** Metabolites of co-culture of *Aspergillus carneus* KMM 4638 and *Amphichorda* sp. KMM 4639.

Compound	Source		
	*Amphichorda* sp. KMM 4639	*Aspergillus carneus* KMM 4638	Co-Culture
**1**	–	–	+
**2**	–	–	+
**3**	–	–	+
**4**	–	–	+
**5**	–	–	+
**6**	–	–	+
**7**	+ [12]	–	+
**8**	–	–	+
**9**	–	+ [5]	+
**10**	–	+ [14]	+
**11**	+ [15]	–	+

“+”—detected in the extract, “–”—not detected in the extract.

## Data Availability

Not applicable.

## Supplementary Materials

The following supporting information can be downloaded at: https://www.mdpi.com/article/10.3390/biom13050741/s1, Figures S1–S37: NMR spectra of compounds **1**–**11**; Figures S38–S57: HPLC profile of FDAA-derivatives of compounds **1**–**5**; Figures S58–S61: MS spectrum of compounds **1**, **2**, **4**, **6**; Figures S62, S64, S66, S68: CD spectra of compounds **1**, **2**, **4**, **6**; Figures S63, S65, S67, S69: UV spectra of compounds **1**, **2**, **4**, **6**.
