# Supplementary material for: New Anti-Hypoxic Metabolites from Co-Culture of Marine-Derived Fungi Aspergillus carneus KMM 4638 and Amphichorda sp. KMM 4639
Source: Biomolecules. 2023 Apr 25;13(5):741. doi: 10.3390/biom13050741 (PMC10216716; doi:10.3390/biom13050741)
Supplement: Supplementary file 1 [file biomolecules-13-00741-s001.zip › biomolecules-2283352-supplementary.pdf]

SUPPLEMENTARY MATERIAL

New anti-hypoxic metabolites from Co-Culture of Marine-Derived Fungi *Aspergillus carneus* KMM 4638 and *Amphichorda* sp. KMM 4639

Elena B. Belousova[1], Olesya I. Zhuravleva[1,2], Ekaterina A. Yurchenko[1,*], Galina K. Oleynikova[1], Alexandr S. Antonov[1], Natalya N. Kirichuk[1], Viktoria E. Chausova[1], Yuliya V. Khudyakova[1], Alexander S. Menshov[1], Roman S. Popov[1], Ekaterina S. Menchinskaya[1], Evgeny A. Pislyagin[1], Valery V. Mikhailov[1,*] and Anton N. Yurchenko[1,*]

[1] G.B. Elyakov Pacific Institute of Bioorganic Chemistry, Far Eastern Branch of the Russian Academy of Sciences, Prospect 100-Letiya Vladivostoka, 159, Vladivostok 690022, Russia
[2] Institute of High Technologies and Advanced Materials, Far Eastern Federal University, 10 Ajax Bay, Russky Island, Vladivostok 690922, Russia
* Correspondence: eyurch@piboc.dvo.ru (E.A.Y.); mikhailov@piboc.dvo.ru (V.V.M.); yurchenkoan@piboc.dvo.ru (A.N.Y.); Tel.: +7-423-231-1168 (A.N.Y.)

Abstract: The KMM 4639 strain was identified as *Amphichorda* sp. based on two molecular genetic markers: ITS and β-tubulin regions. Chemical investigation of co-culture marine-derived fungi *Amphichorda* sp. KMM 4639 and *Aspergillus carneus* KMM 4638 led to the identification of five new quinazolinone alkaloids felicarnezolines A–E (**1**–**5**), a new highly oxygenated chromene derivative oxirapentyn M (**6**) and five previously reported related compounds. Their structures were established using spectroscopic methods and by comparison with related known compounds. The isolated compounds shown low cytotoxicity against human prostate and breast cancer cells but felicarnezoline B (**2**) protected rat cardiomyocytes H9c2 and human neuroblastoma SH-SY5Y cells against $CoCl_2$-induced damage.

Keywords: marine-derived fungi; co-culture; secondary metabolites; quinazoline alkaloid; oxyrapentyn; cytoprotection; cobalt chloride (II); antioxidants; ITS; β-tubulin; phylogeny.

Content

Figure S1. ^{1}H NMR spectrum of felicarnezoline A (1).. 5
Figure S2. ^{13}C NMR spectrum of felicarnezoline A (1).. 9
Figure S3. ^{1}H-^{1}H COSY spectrum of felicarnezoline A (1).. 10
Figure S4. HSQC spectrum of felicarnezoline A (1)... 11
Figure S5. HMBC spectrum of felicarnezoline A (1) 12
Figure S6. ROESY spectrum of felicarnezoline A (1) 13
Figure S7. ^{1}H NMR spectrum of felicarnezoline B (2) 14
Figure S8. ^{13}C NMR spectrum of felicarnezoline B (2) 18
Figure S9. DEPT-135 spectrum of felicarnezoline B (2) 19
Figure S10. ^{1}H-^{1}H COSY spectrum of felicarnezoline B (2) 20
Figure S11. HSQC spectrum of felicarnezoline B (2).. 21
Figure S12. HMBC spectrum of felicarnezoline B (2).. 22
Figure S13. ROESY spectrum of felicarnezoline B (2) 23
Figure S14. ^{1}H NMR spectrum of felicarnezoline C (3) 24
Figure S15. ^{1}H NMR spectrum of felicarnezoline D (4)... 28
Figure S16. ^{1}H NMR spectrum of felicarnezoline E (5) 34
Figure S17. ^{1}H NMR spectrum of oxyrapentin M (6).. 37
Figure S18. ^{13}C NMR spectrum of oxyrapentin M (6).. 42
Figure S19. DEPT-135 spectrum of oxyrapentin M (6) 43
Figure S20. ^{1}H-^{1}H COSY spectrum of oxyrapentin M (6).. 44
Figure S21. HSQC spectrum of oxyrapentin M (6).. 45
Figure S22. HMBC spectrum of oxyrapentin M (6) 46
Figure S23. ROESY spectrum of oxyrapentin M (6) 47
Figure S24. ^{1}H NMR spectrum of oxyrapentin B (7).. 48
Figure S25. ^{1}H NMR spectrum of cinereain (8)... 52
Figure S26. ^{1}H NMR spectrum of carneamide A (9) 55
Figure S27. ^{1}H NMR spectrum of aspergillicine A (10) 61
Figure S28. ^{1}H-^{1}H COSY spectrum of aspergillicine A (10) 66
Figure S29. HSQC spectrum of aspergillicine A (10).. 67
Figure S30. HMBC spectrum of aspergillicine A (10).. 68
Figure S31. ^{1}H NMR spectrum of isaridine E (11) 69
Figure S32. ^{13}C NMR spectrum of isaridine E (11) 73

Figure S33. DEPT-135 spectrum of isaridine E (11) 74
Figure S34. ^{1}H-^{1}H COSY spectrum of isaridine E (11) 75
Figure S35. HSQC spectrum of isaridine E (11) 76
Figure S36. HMBC spectrum of isaridine E (11) 77
Figure S37. NOESY spectrum of isaridine E (11) 78
Figure S38. HPLC profile of *L*-FDAA -derivatives of felicarnezoline A (1) hydrolysate 79
Figure S39. HPLC profile of *L*-FDAA -derivatives of felicarnezoline A (1) HP+*L*-Val80
Figure S40. HPLC profile of *L*-FDAA -derivatives of felicarnezoline A (1) HP+*D*,*L*-Val 81
Figure S41. HPLC profiles of *L*-FDAA -derivatives of *L*-Val (a), *L*-FDAA -derivatives of *D*,*L*-Val (b), *L*-FDAA -derivatives of felicarnezoline A (1) HP (c), felicarnezoline A (1) HP+*D*,*L*-Val (d) and felicarnezoline A (1) HP+*L*-Val (f) 82
Figure S42. HPLC profile of *L*-FDAA-derivatives of felicarnezoline B (2) HP 83
Figure S43. HPLC profile of *L*-FDAA -derivatives of felicarnezoline B (2) HP+*L*-Val84
Figure S44. HPLC profile of *L*-FDAA -derivatives of felicarnezoline B (2) HP+*D*,*L*-Val 85
Figure S45. HPLC profiles of *L*-FDAA -derivatives of *L*-Val (a), *L*-FDAA -derivatives of *D*,*L*-Val (b), *L*-FDAA -derivatives of felicarnezoline B (2) HP (c), felicarnezoline B (2) HP+*D*,*L*-Val (d) and felicarnezoline B HP+*L*-Val (f) 86
Figure S46. HPLC profile of *L*-FDAA -derivatives of felicarnezoline C (3) HP 87
Figure S47. HPLC profile of *L*-FDAA -derivatives of felicarnezoline C (3) HP +*L*-Val 88
Figure S48. HPLC profile of *L*-FDAA -derivatives of felicarnezoline C (3) HP *D*,*L*-Val 89
Figure S49. HPLC profiles of *L*-FDAA -derivatives of *L*-Val (a), *L*-FDAA -derivatives of *D*,*L*-Val (b), *L*-FDAA -derivatives of felicarnezoline C (3) HP (c), felicarnezoline C (3) HP+*D*,*L*-Val (d) and felicarnezoline C (3) HP+*L*-Val (f).. 90
Figure S50. HPLC profile of *L*-FDAA -derivatives of felicarnezoline D (4) HP 91
Figure S51. HPLC profile of *L*-FDAA -derivatives of felicarnezoline D (4) HP +*L*-Val 92
Figure S52. HPLC profile of *L*-FDAA -derivatives of felicarnezoline D (4) HP *D*,*L*-Val 93
Figure S53. HPLC profiles of *L*-FDAA -derivatives of *L*-Val (a), *L*-FDAA -derivatives of *D*,*L*-Val (b), *L*-FDAA -derivatives of felicarnezoline D (4) HP (c), felicarnezoline D (4) HP+*D*,*L*-Val (d) and felicarnezoline D (4) HP+*L*-Val (f) 94
Figure S54. HPLC profile of *L*-FDAA -derivatives of felicarnezoline E (5) HP........... 95

Figure S55. HPLC profile of *L*-FDAA -derivatives of felicarnezoline E (5) HP +*L*-Val 96
Figure S56. HPLC profile of *L*-FDAA -derivatives of felicarnezoline E (5) HP *D*,*L*-Val 97
Figure S57. HPLC profiles of *L*-FDAA -derivatives of *L*-Val (a), *L*-FDAA -derivatives of *D*,*L*-Val (b), *L*-FDAA -derivatives of felicarnezoline E (5) HP (c), felicarnezoline E (5) HP +*D*,*L*-Val (d) and felicarnezoline E (5) HP+*L*-Val (f).. 98
Figure S58. MS spectrum of felicarnezoline A (1) 99
Figure S59. MS spectrum of felicarnezoline B (2).. 100
Figure S60. MS spectrum of felicarnezoline D (4) 101
Figure S61. MS spectrum of oxyrapentin M (6) 103
Figure S62. CD spectrum of felicarnezoline A (1).. 105
Figure S63. UV spectrum of felicarnezoline A (1) 106
Figure S64. CD spectrum of felicarnezoline B (2).. 107
Figure S65. UV spectrum of felicarnezoline B (2).. 108
Figure S66. CD spectrum of felicarnezoline D (4).. 109
Figure S67. UV spectrum of felicarnezoline D (4) 110
Figure S68. CD spectrum of oxyrapentin M (6).. 111
Figure S69. UV spectrum of oxyrapentin M (6) 112
Table S1. ^{1}H NMR data for carnequinazoline A [1] and felicarnezoline C (**3**)........... 113
Table S2. ^{1}H and ^{13}C NMR data ($CDCl_3$, 700 MHz) for carnequinazoline B [1] and felicarnezoline D (**4**)... 114
Table S3. ^{1}H and ^{13}C NMR data ($CDCl_3$, 500 MHz) for dihydrocinereain [1] and compound felicarnezoline E (**5**). 115

Figure S1. ^{1}**H NMR spectrum of felicarnezoline A (1)**

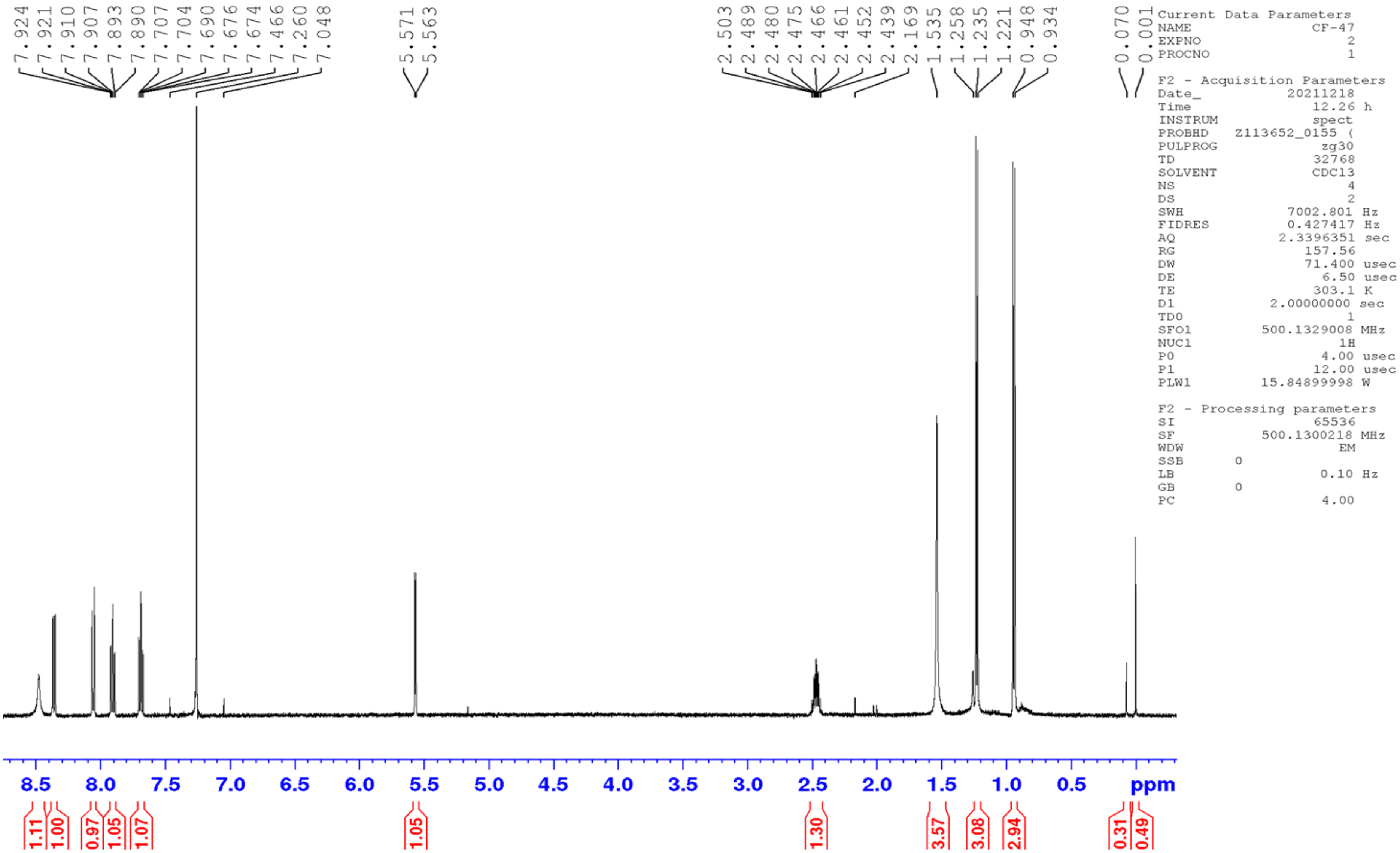

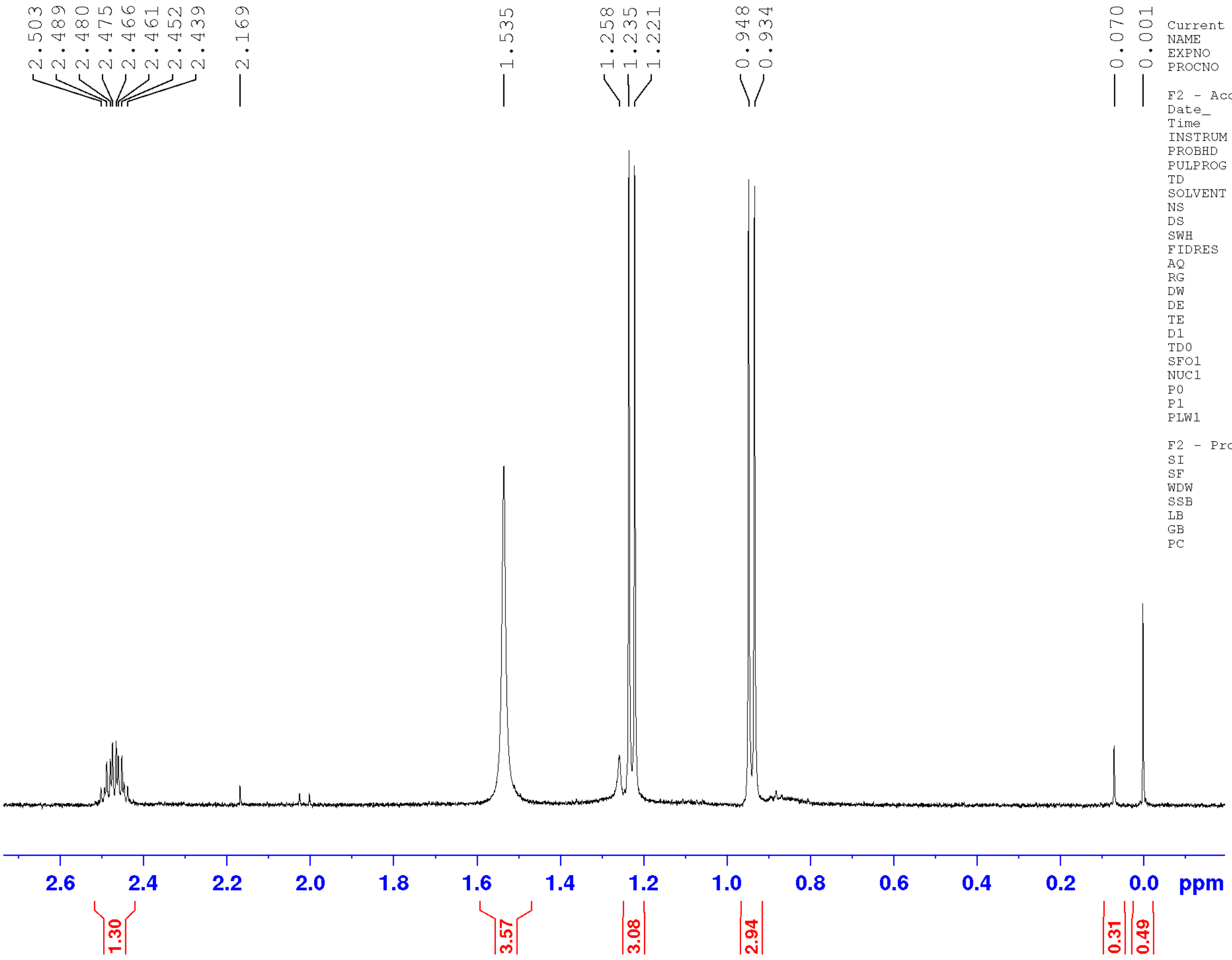

Current Data Parameters
NAME CF-47
EXPNO 2
PROCNO 1

F2 - Acquisition Parameters
Date_ 20211218
Time 12.26 h
INSTRUM spect
PROBHD Z113652_0155 (
PULPROG zg30
TD 32768
SOLVENT CDCl3
NS 4
DS 2
SWH 7002.801 Hz
FIDRES 0.427417 Hz
AQ 2.3396351 sec
RG 157.56
DW 71.400 usec
DE 6.50 usec
TE 303.1 K
D1 2.00000000 sec
TD0 1
SFO1 500.1329008 MHz
NUC1 1H
P0 4.00 usec
P1 12.00 usec
PLW1 15.84899998 W

F2 - Processing parameters
SI 65536
SF 500.1300218 MHz
WDW EM
SSB 0
LB 0.10 Hz
GB 0
PC 4.00

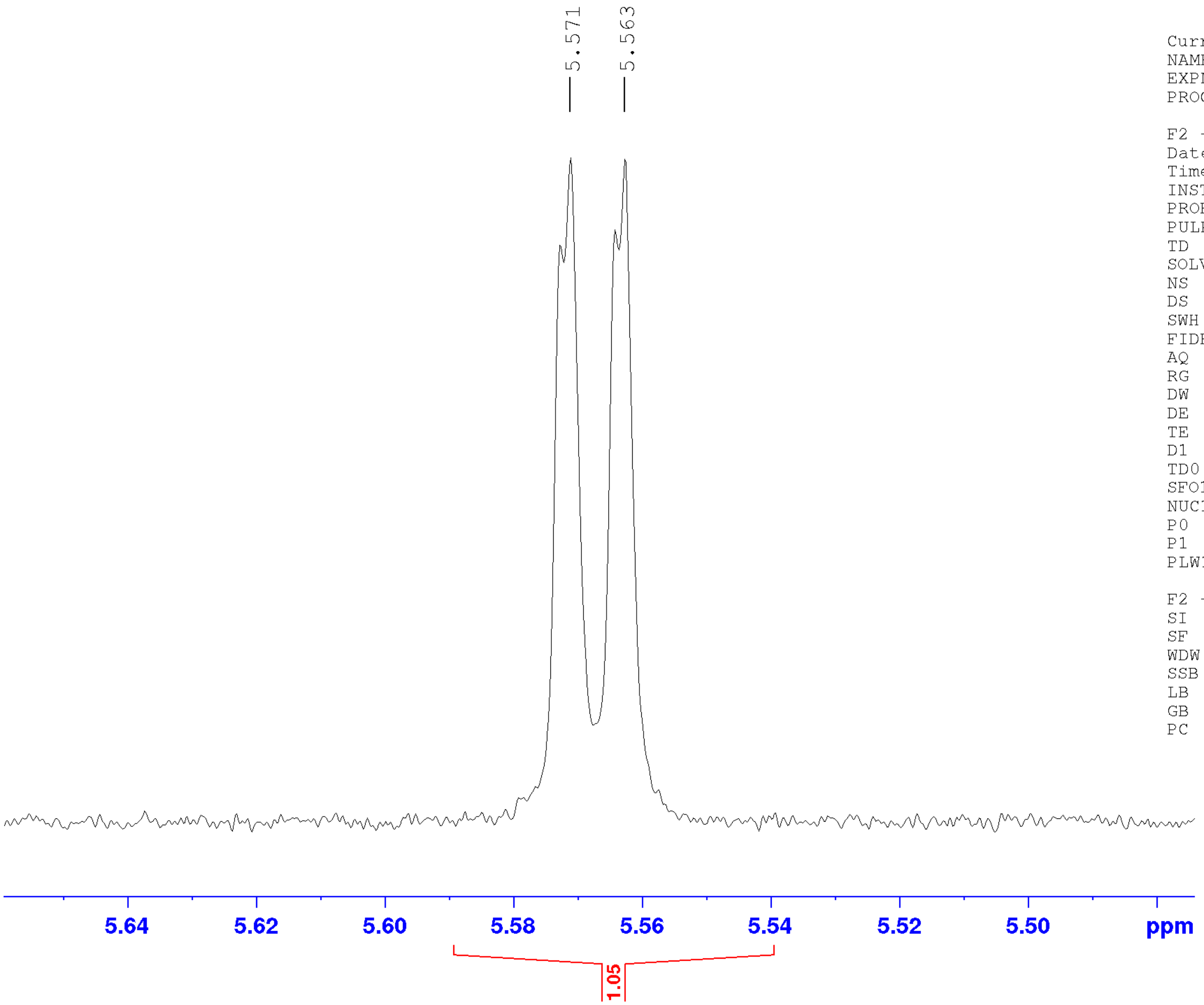

Current Data Parameters
NAME CF-47
EXPNO 2
PROCNO 1

F2 - Acquisition Parameters
Date_ 20211218
Time 12.26 h
INSTRUM spect
PROBHD Z113652_0155 (
PULPROG zg30
TD 32768
SOLVENT CDCl3
NS 4
DS 2
SWH 7002.801 Hz
FIDRES 0.427417 Hz
AQ 2.3396351 sec
RG 157.56
DW 71.400 usec
DE 6.50 usec
TE 303.1 K
D1 2.00000000 sec
TD0 1
SFO1 500.1329008 MHz
NUC1 1H
P0 4.00 usec
P1 12.00 usec
PLW1 15.84899998 W

F2 - Processing parameters
SI 65536
SF 500.1300218 MHz
WDW EM
SSB 0
LB 0.10 Hz
GB 0
PC 4.00

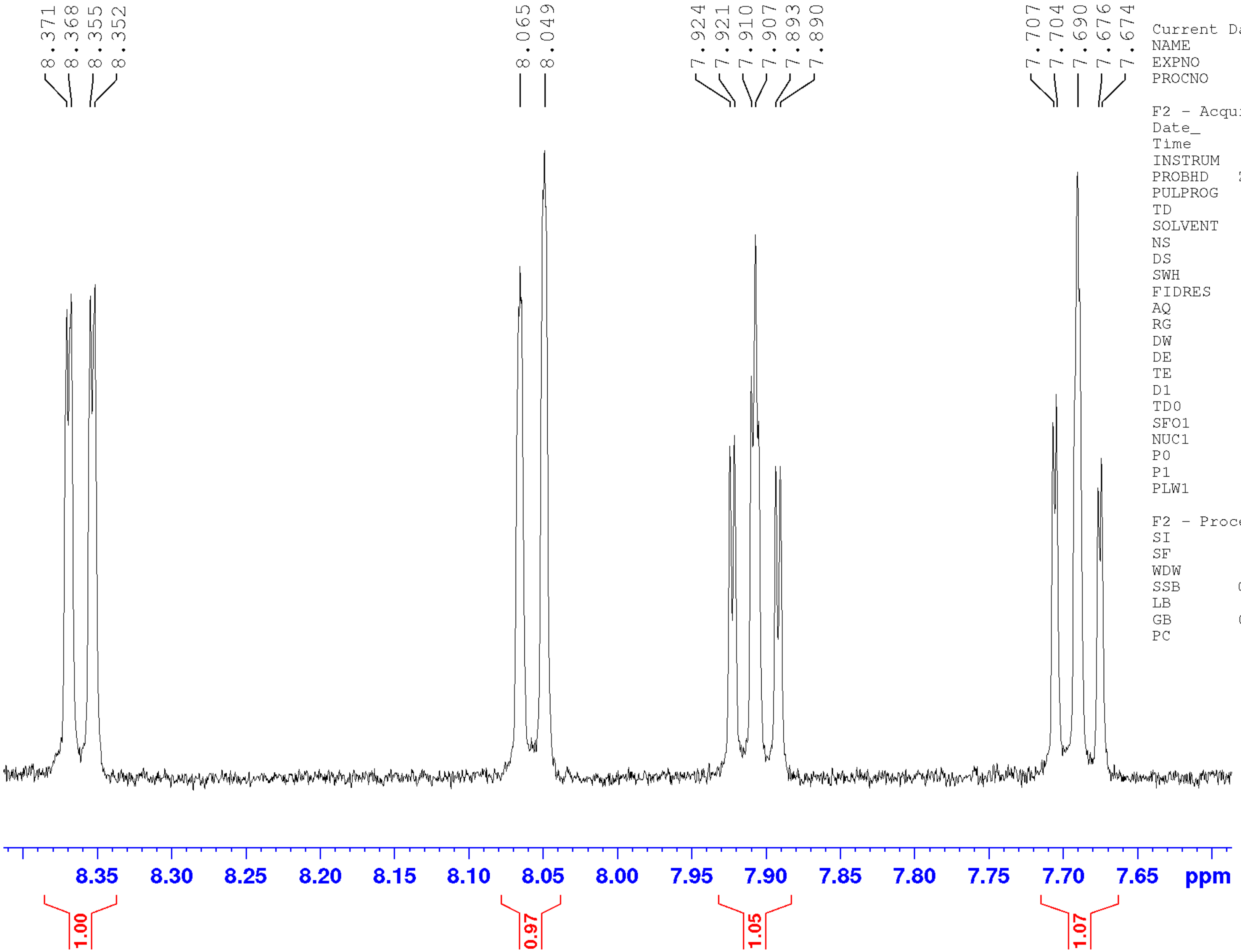


```
Current Data Parameters
NAME                CF-47
EXPNO                   2
PROCNO                  1

F2 - Acquisition Parameters
Date_            20211218
Time                12.26 h
INSTRUM             spect
PROBHD   Z113652_0155 (
PULPROG              zg30
TD                  32768
SOLVENT             CDCl3
NS                      4
DS                      2
SWH              7002.801 Hz
FIDRES           0.427417 Hz
AQ              2.3396351 sec
RG                 157.56
DW                 71.400 usec
DE                   6.50 usec
TE                  303.1 K
D1             2.00000000 sec
TD0                     1
SFO1          500.1329008 MHz
NUC1                   1H
P0                   4.00 usec
P1                  12.00 usec
PLW1          15.84899998 W

F2 - Processing parameters
SI                  65536
SF            500.1300218 MHz
WDW                    EM
SSB      0
LB                   0.10 Hz
GB       0
PC                   4.00
```

Figure S2. [13]**C NMR spectrum of felicarnezoline A (1)**

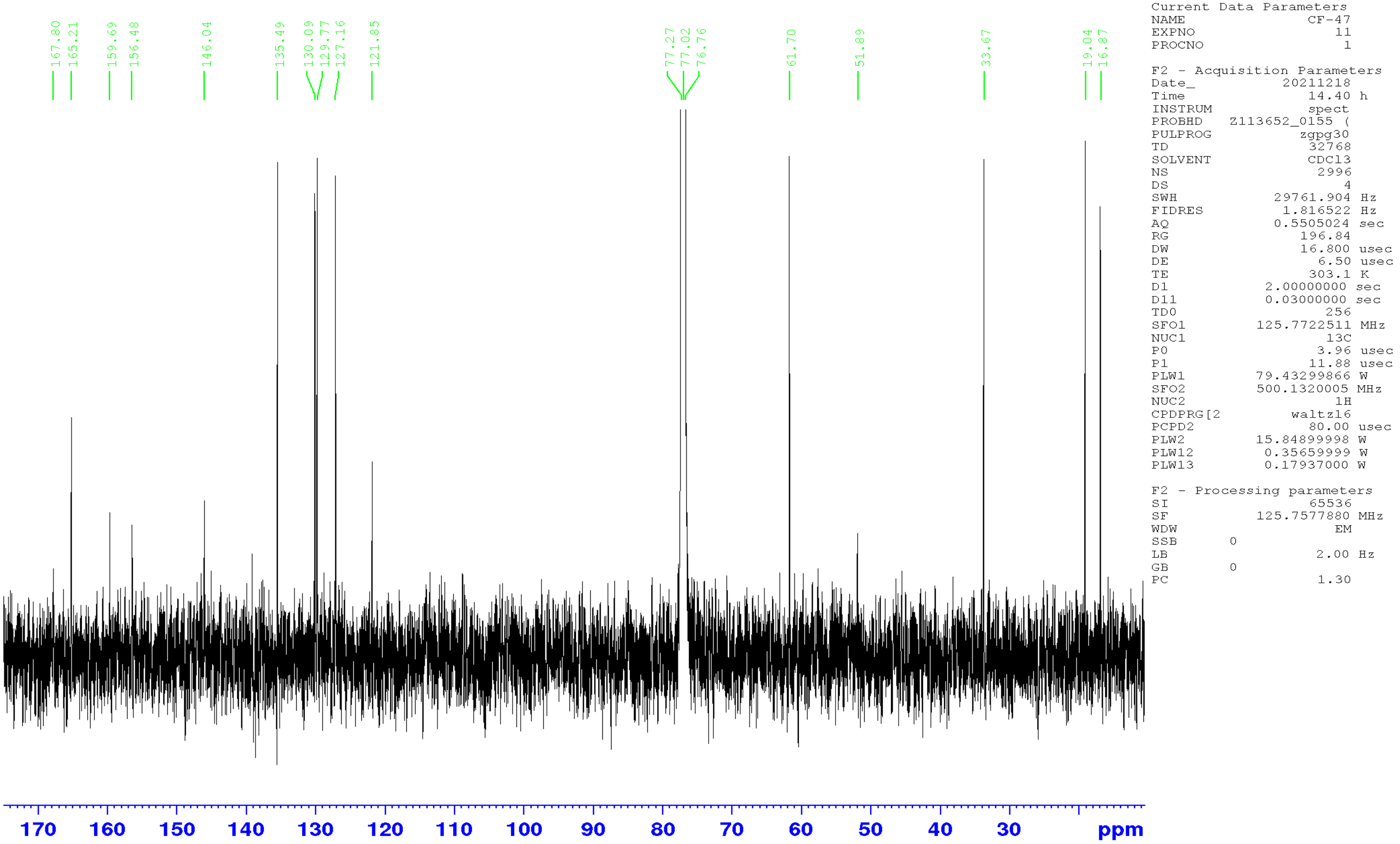

Figure S3. ^{1}H-^{1}H COSY spectrum of felicarnezoline A (1)

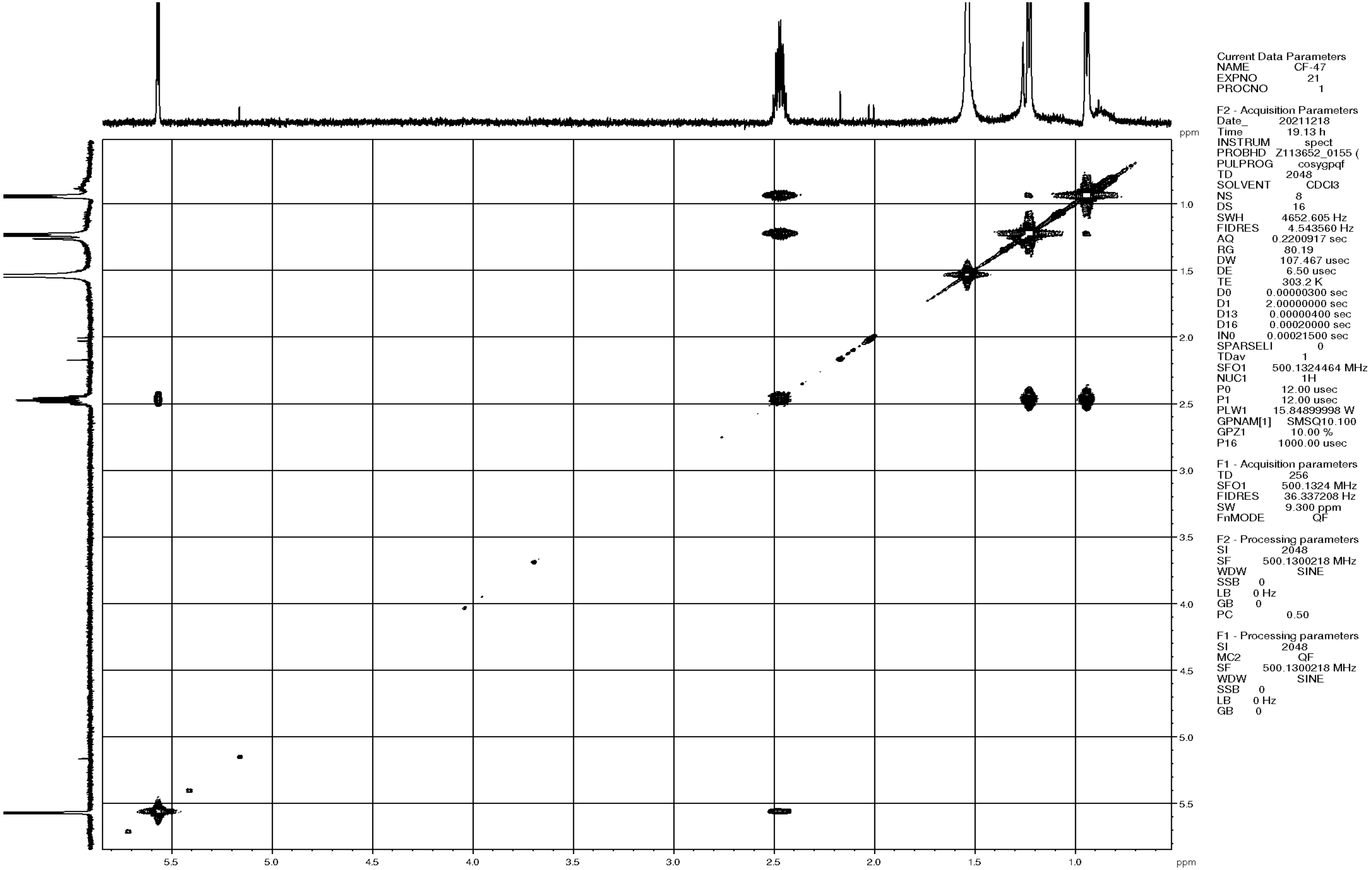

Figure S4. HSQC spectrum of felicarnezoline A (1)

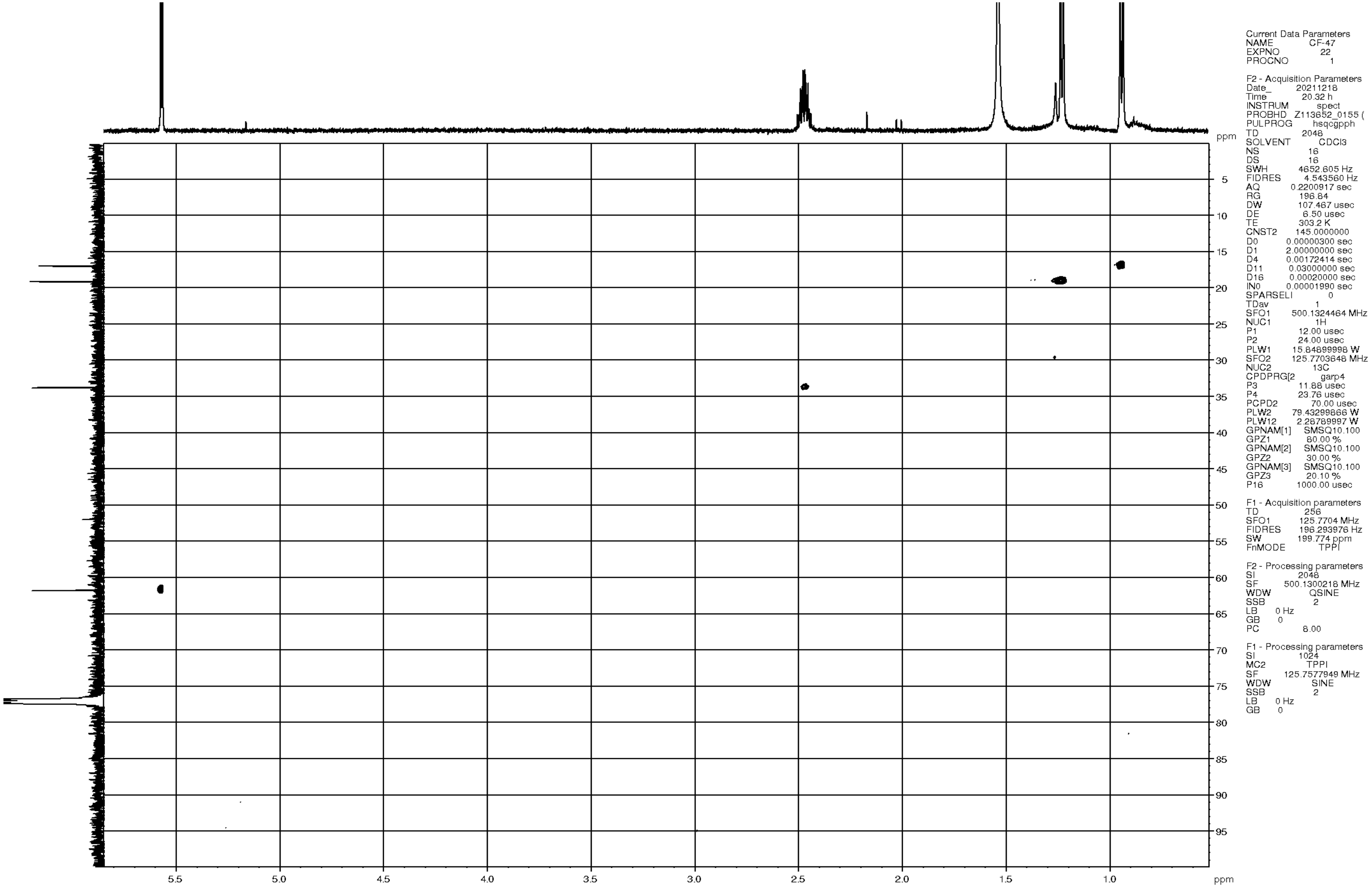

Figure S5. HMBC spectrum of felicarnezoline A (1)

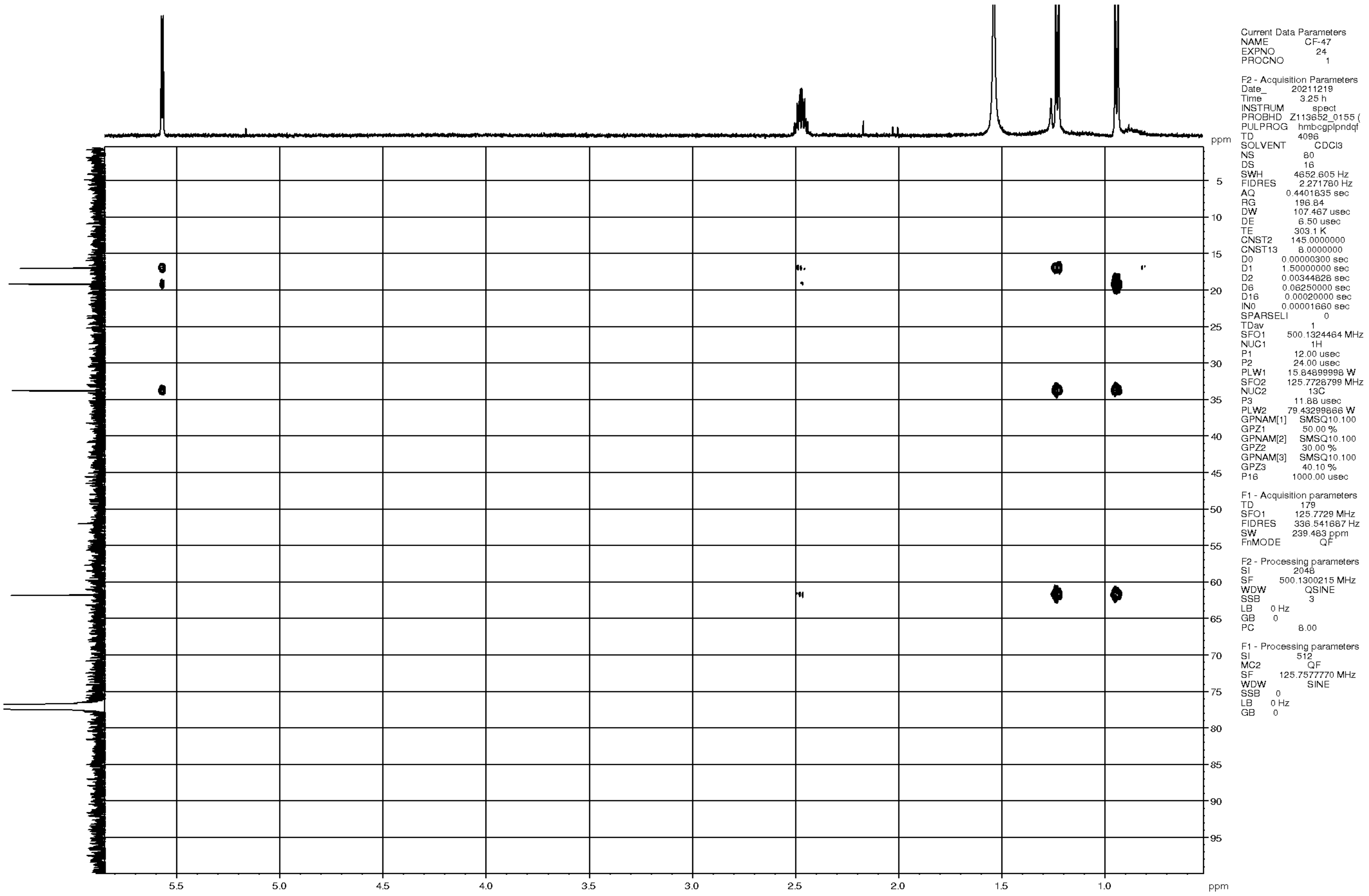

Figure S6. ROESY spectrum of felicarnezoline A (1)

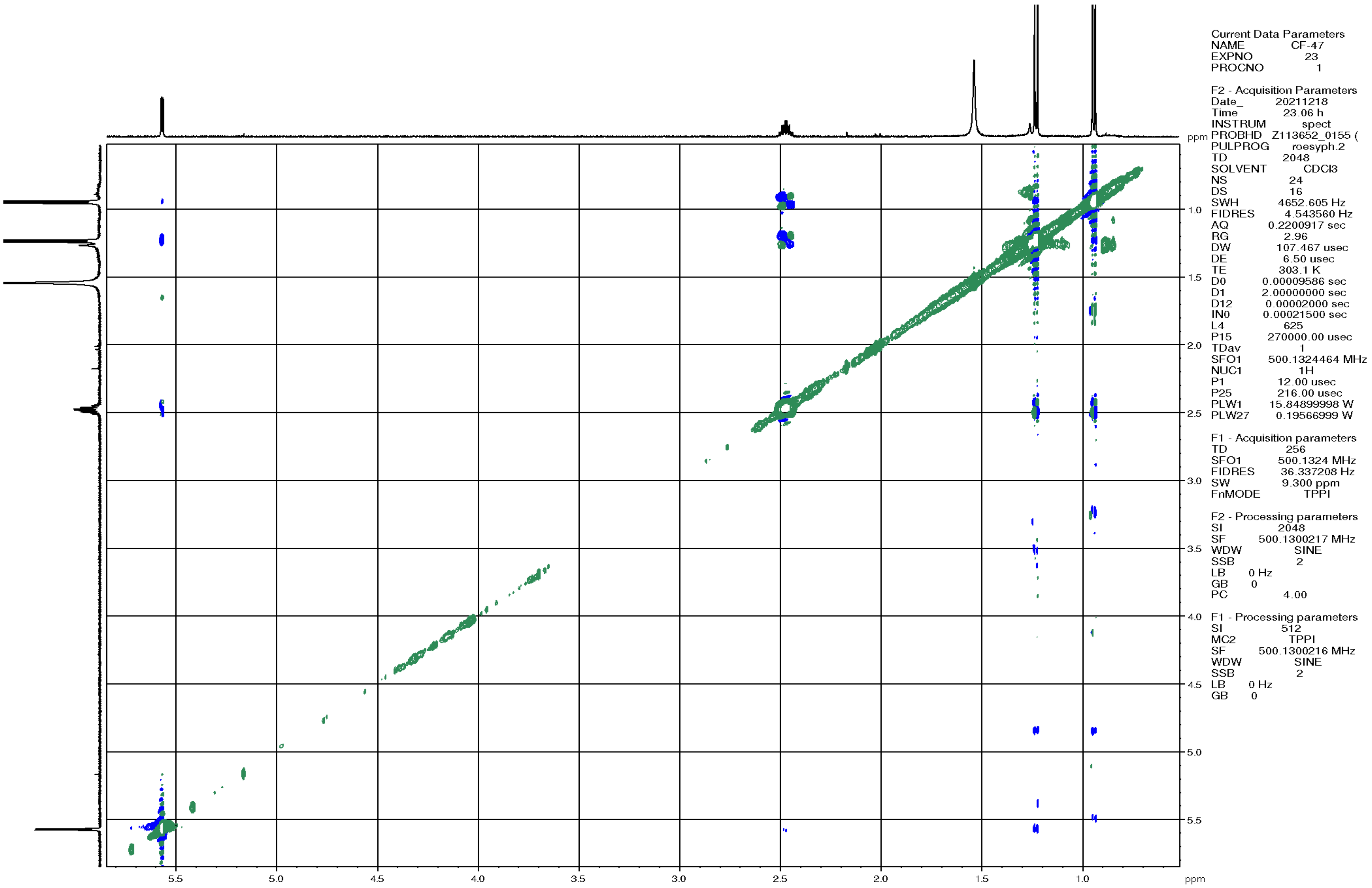

Figure S7. ¹H NMR spectrum of felicarnezoline B (**2**)

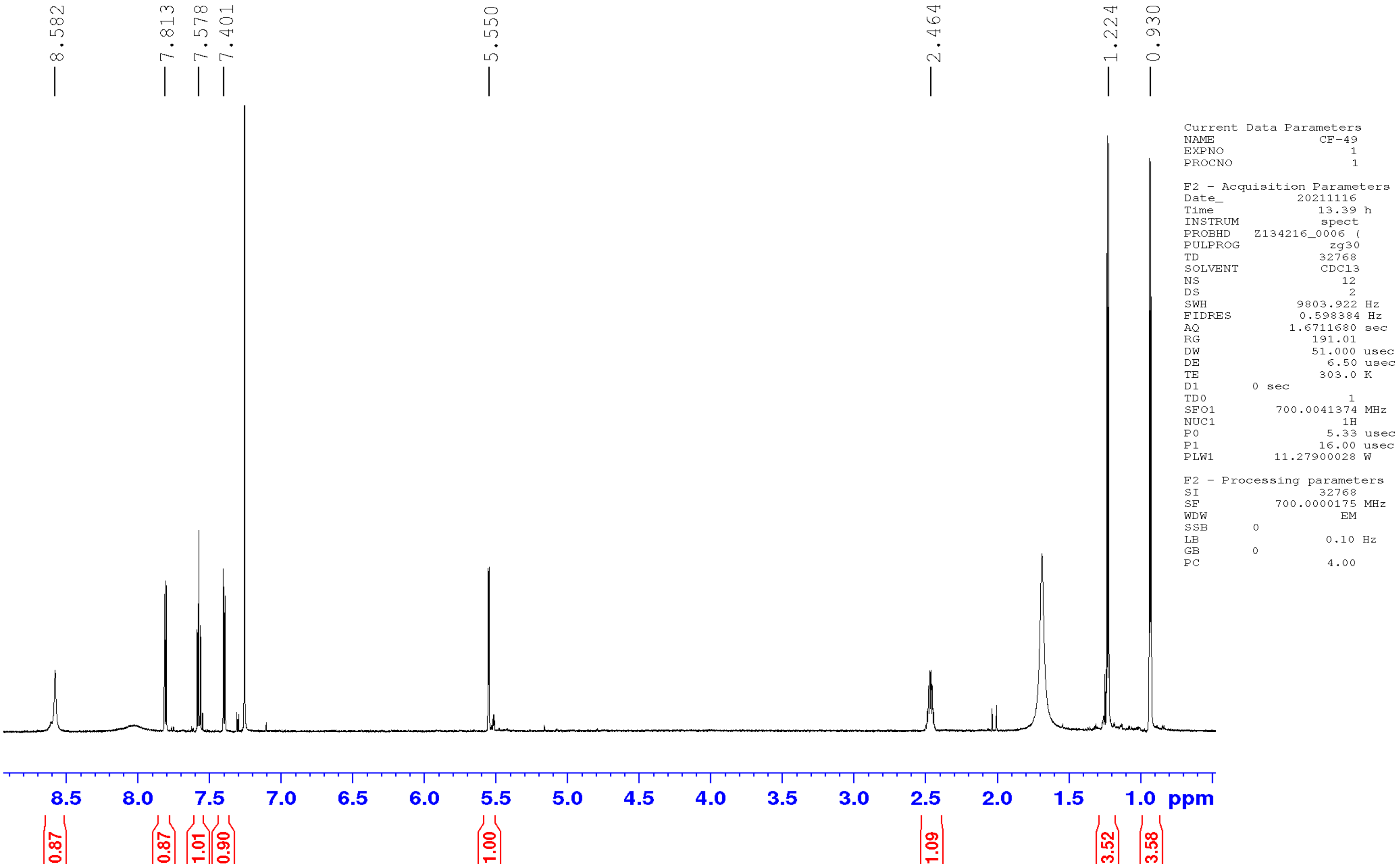

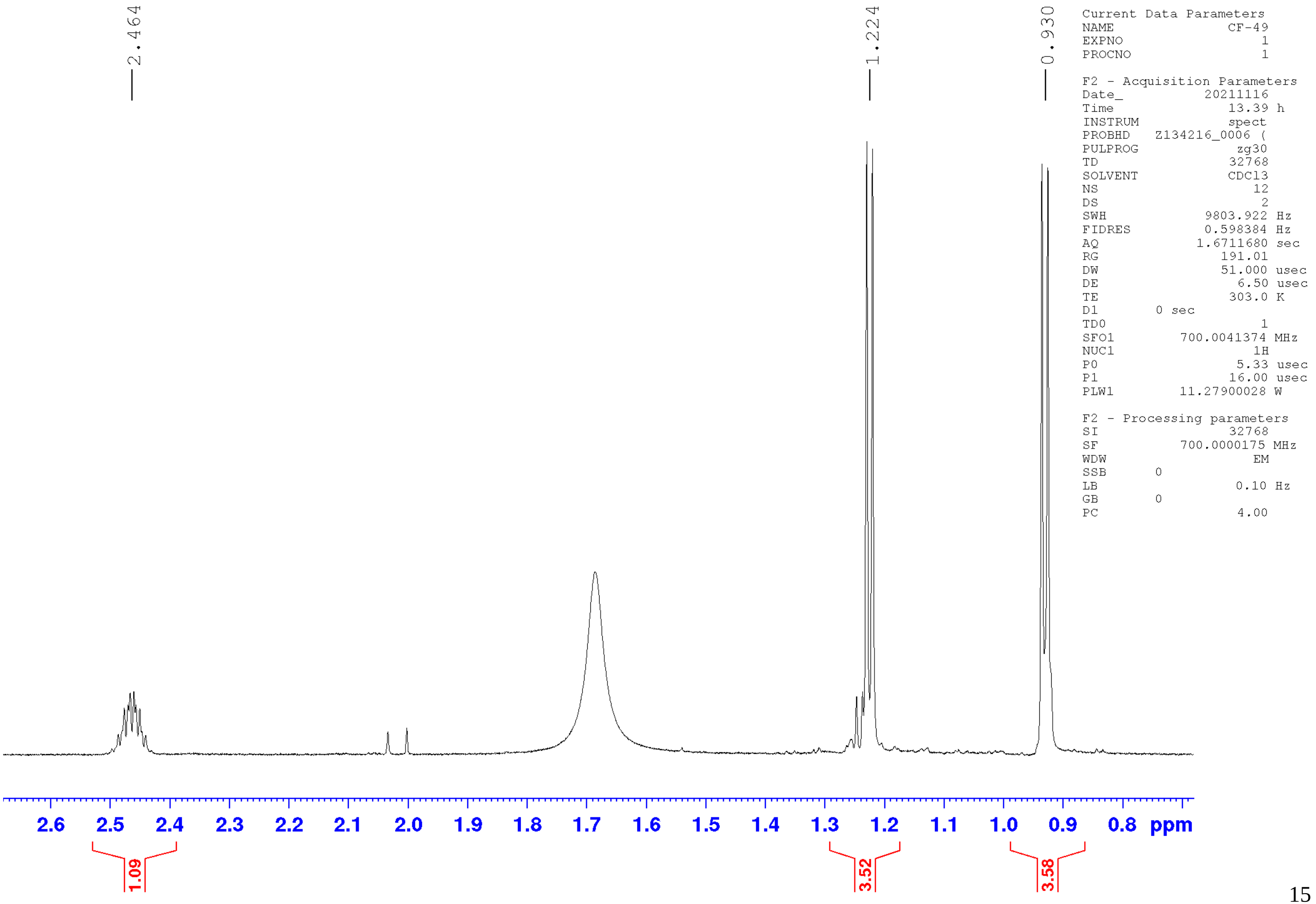
2.464
1.224
0.930
Current Data Parameters
NAME CF-49
EXPNO 1
PROCNO 1
F2 - Acquisition Parameters
Date_ 20211116
Time 13.39 h
INSTRUM spect
PROBHD Z134216_0006 (
PULPROG zg30
TD 32768
SOLVENT CDCl3
NS 12
DS 2
SWH 9803.922 Hz
FIDRES 0.598384 Hz
AQ 1.6711680 sec
RG 191.01
DW 51.000 usec
DE 6.50 usec
TE 303.0 K
D1 0 sec
TD0 1
SFO1 700.0041374 MHz
NUC1 1H
P0 5.33 usec
P1 16.00 usec
PLW1 11.27900028 W
F2 - Processing parameters
SI 32768
SF 700.0000175 MHz
WDW EM
SSB 0
LB 0.10 Hz
GB 0
PC 4.00
2.6 2.5 2.4 2.3 2.2 2.1 2.0 1.9 1.8 1.7 1.6 1.5 1.4 1.3 1.2 1.1 1.0 0.9 0.8 ppm
1.09
3.52
3.58

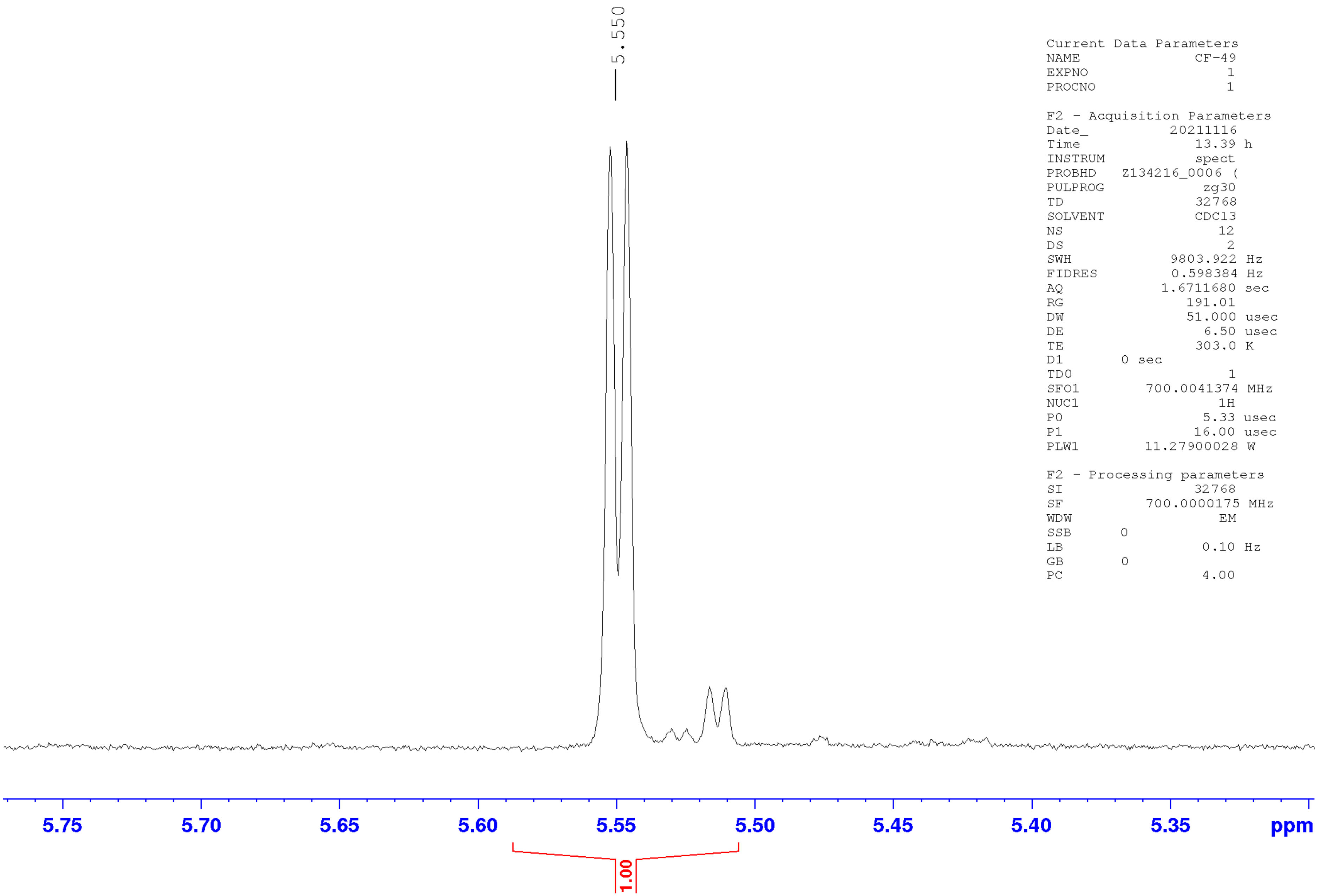

Current Data Parameters
NAME CF-49
EXPNO 1
PROCNO 1

F2 - Acquisition Parameters
Date_ 20211116
Time 13.39 h
INSTRUM spect
PROBHD Z134216_0006 (
PULPROG zg30
TD 32768
SOLVENT CDCl3
NS 12
DS 2
SWH 9803.922 Hz
FIDRES 0.598384 Hz
AQ 1.6711680 sec
RG 191.01
DW 51.000 usec
DE 6.50 usec
TE 303.0 K
D1 0 sec
TD0 1
SFO1 700.0041374 MHz
NUC1 1H
P0 5.33 usec
P1 16.00 usec
PLW1 11.27900028 W

F2 - Processing parameters
SI 32768
SF 700.0000175 MHz
WDW EM
SSB 0
LB 0.10 Hz
GB 0
PC 4.00

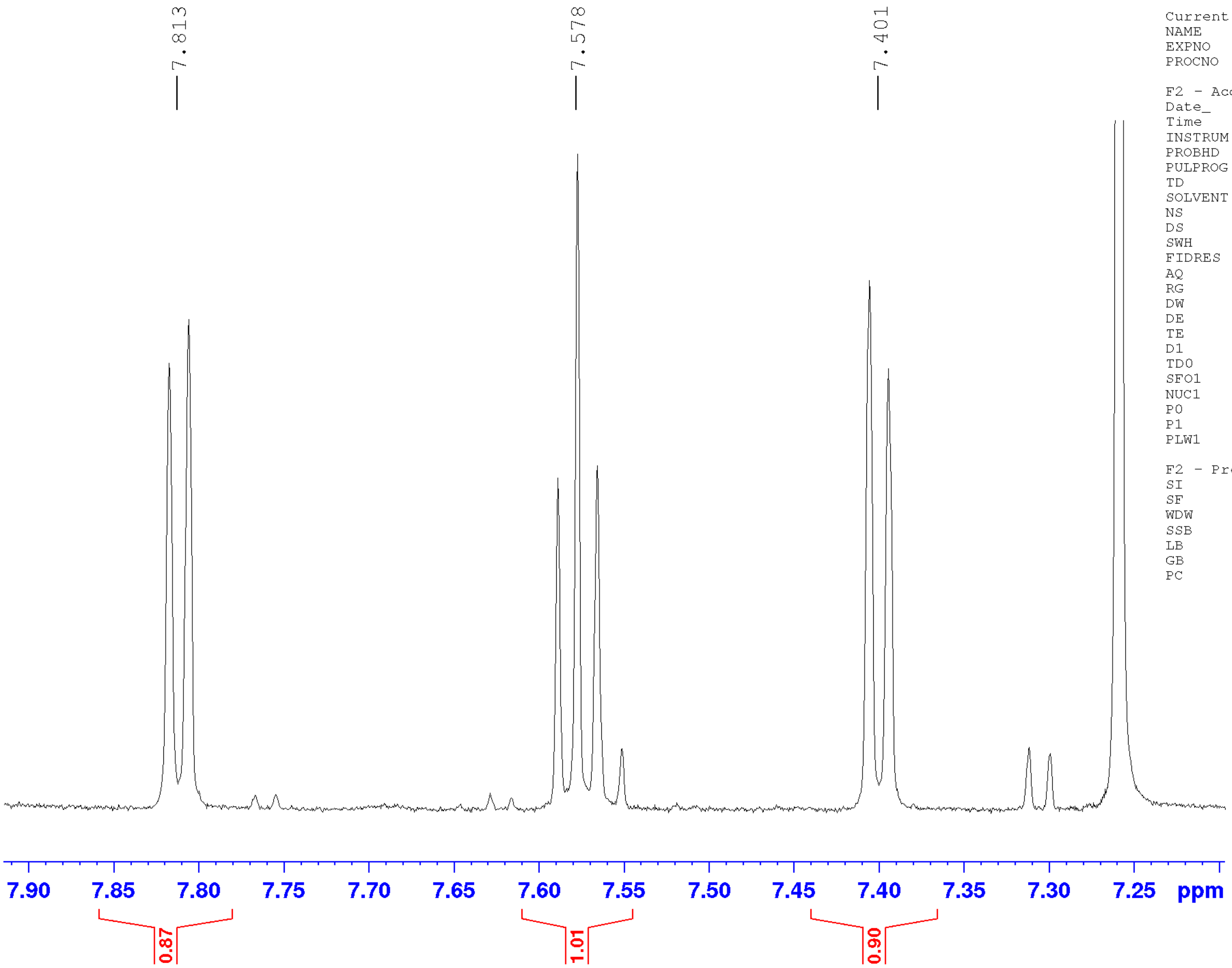

Current Data Parameters
NAME CF-49
EXPNO 1
PROCNO 1

F2 - Acquisition Parameters
Date_ 20211116
Time 13.39 h
INSTRUM spect
PROBHD Z134216_0006 (
PULPROG zg30
TD 32768
SOLVENT CDCl3
NS 12
DS 2
SWH 9803.922 Hz
FIDRES 0.598384 Hz
AQ 1.6711680 sec
RG 191.01
DW 51.000 usec
DE 6.50 usec
TE 303.0 K
D1 0 sec
TD0 1
SFO1 700.0041374 MHz
NUC1 1H
P0 5.33 usec
P1 16.00 usec
PLW1 11.27900028 W

F2 - Processing parameters
SI 32768
SF 700.0000175 MHz
WDW EM
SSB 0
LB 0.10 Hz
GB 0
PC 4.00

Figure S8. ^{13}C NMR spectrum of felicarnezoline B (2)

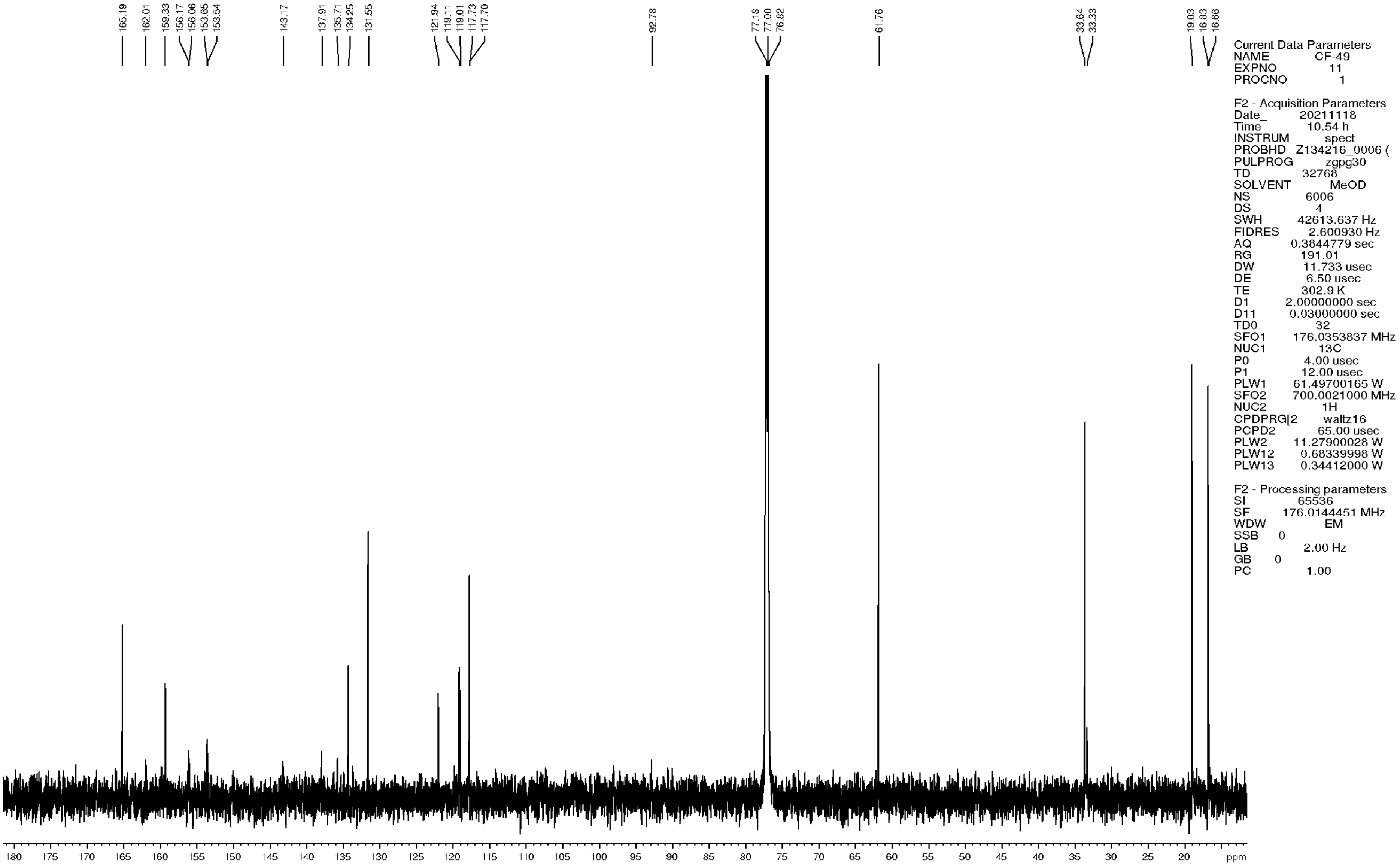

Figure S9. DEPT-135 spectrum of felicarnezoline B (2)

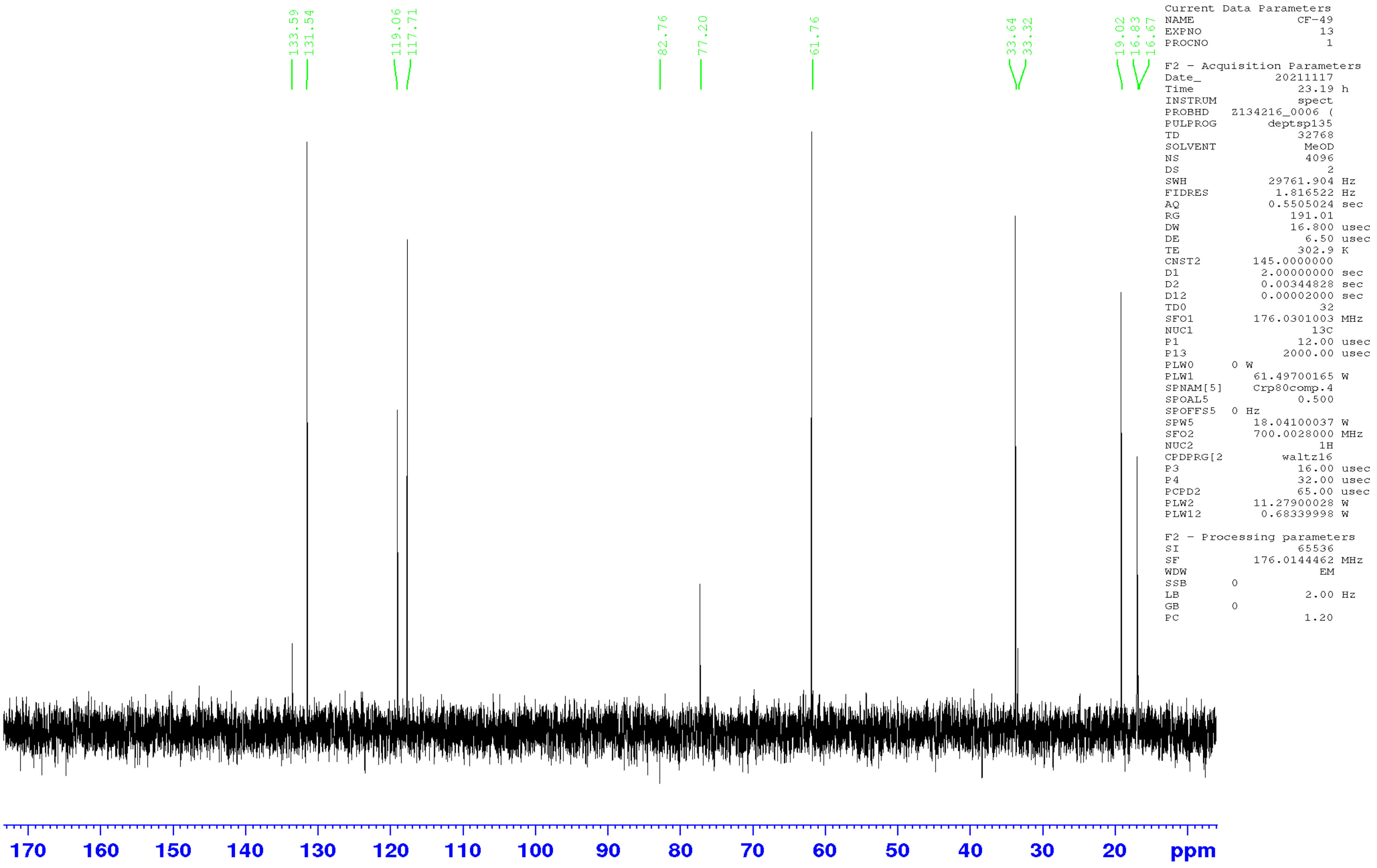

Figure S10. ^1H-^1H COSY spectrum of felicarnezoline B (2)

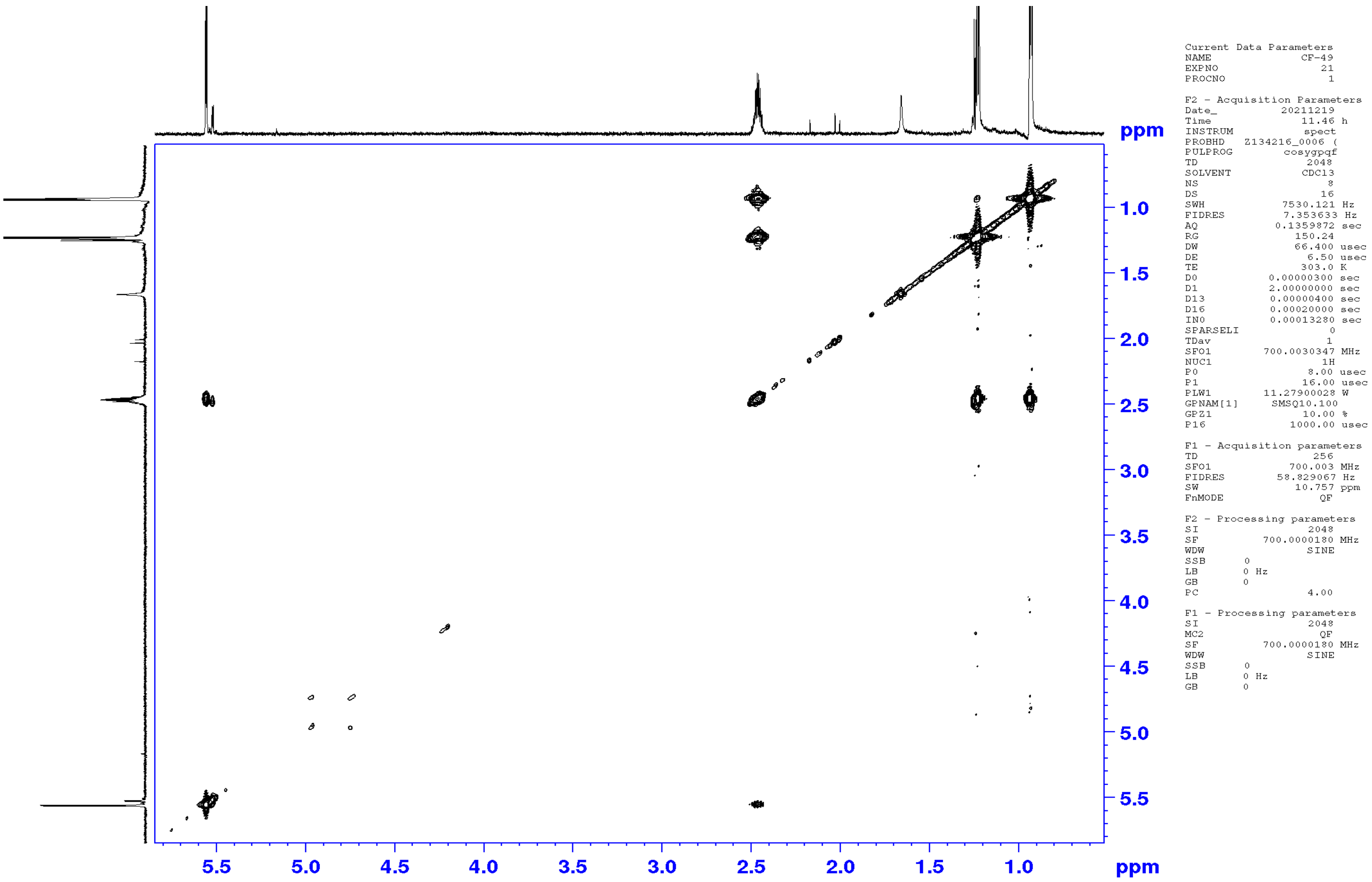

Figure S11. HSQC spectrum of felicarnezoline B (2)

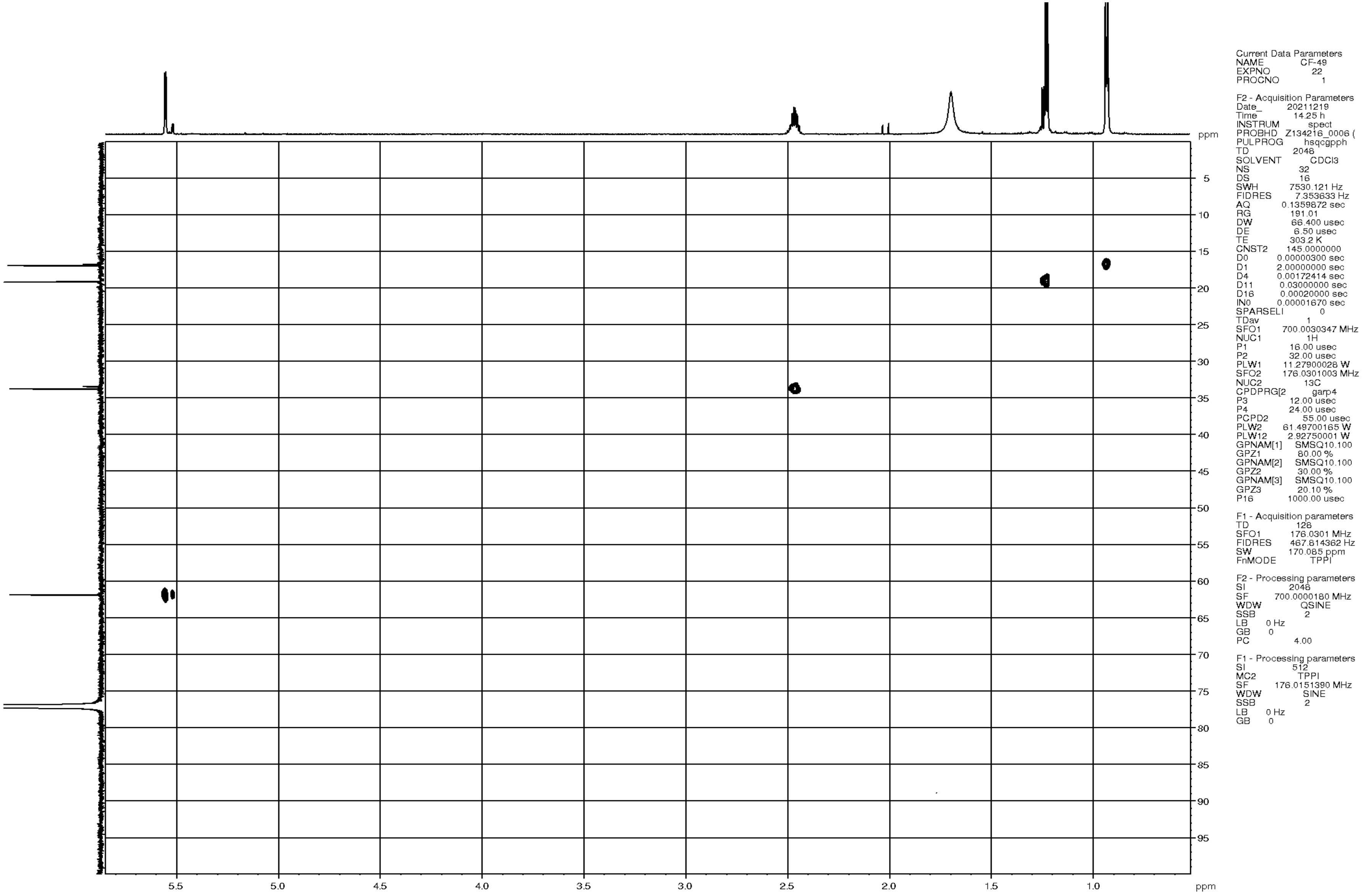

Figure S12. HMBC spectrum of felicarnezoline B (2)

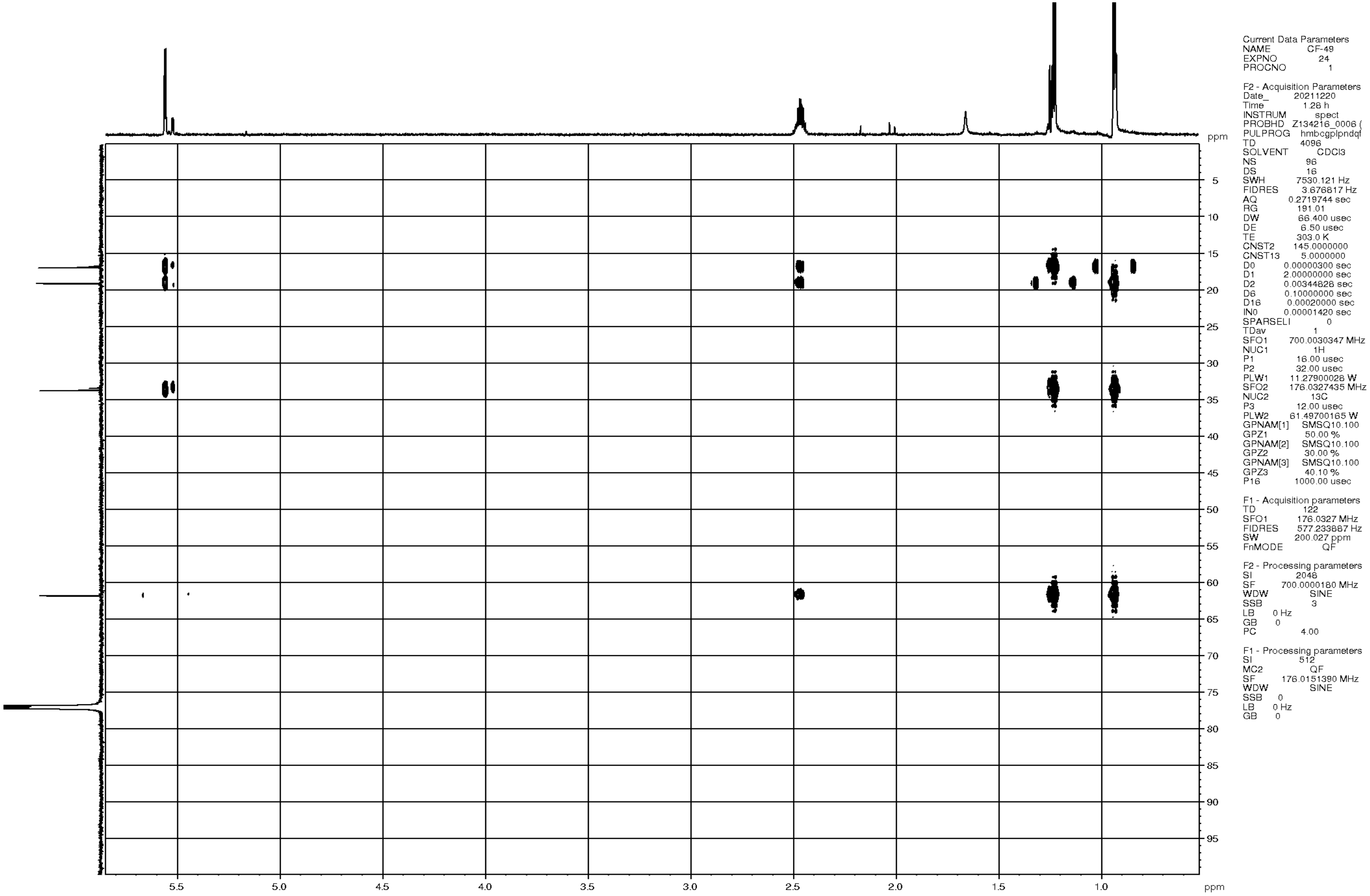

Figure S13. ROESY spectrum of felicarnezoline B (2)

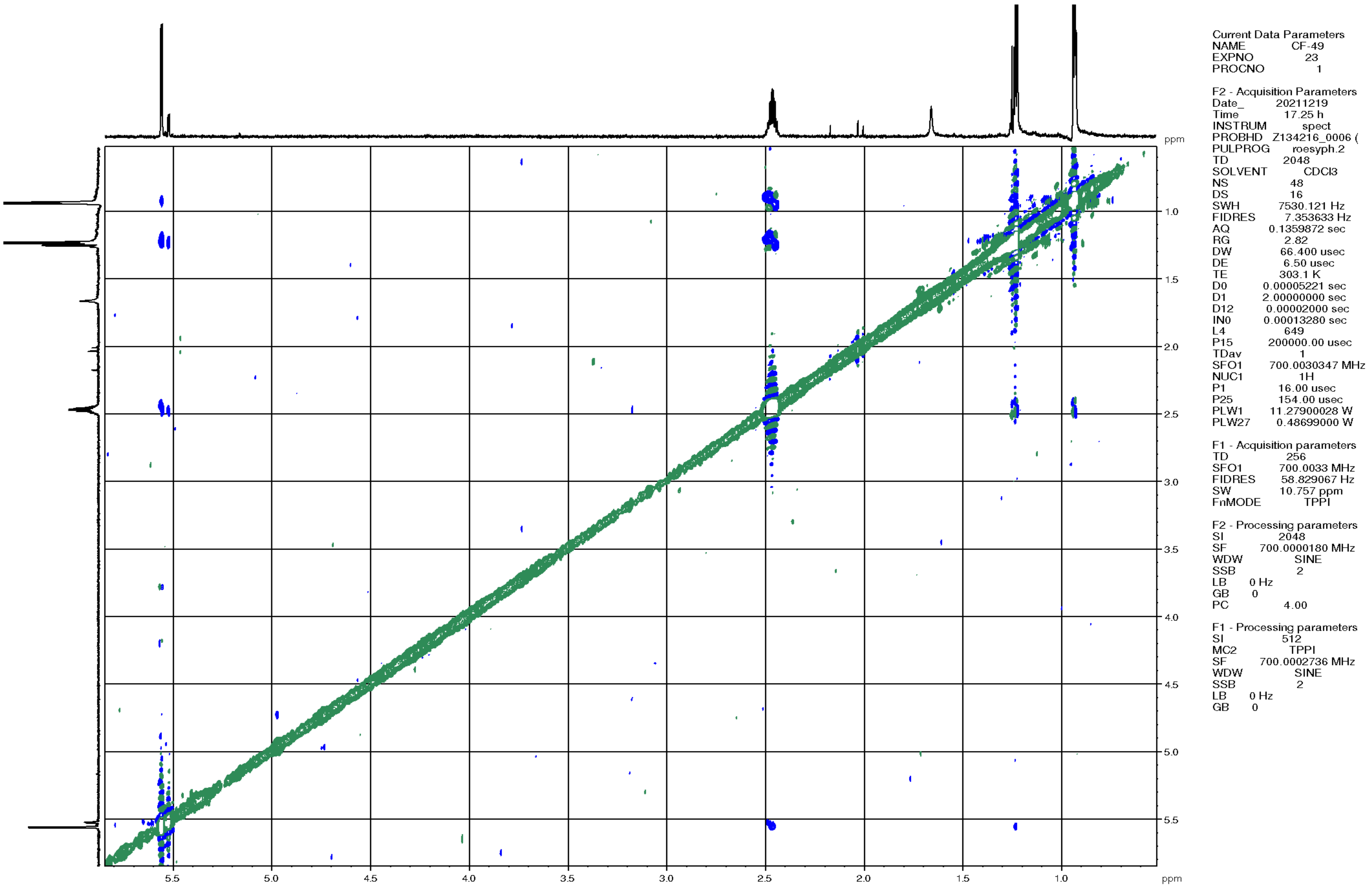

Figure S14. ^{1}H NMR spectrum of felicarnezoline C (3)

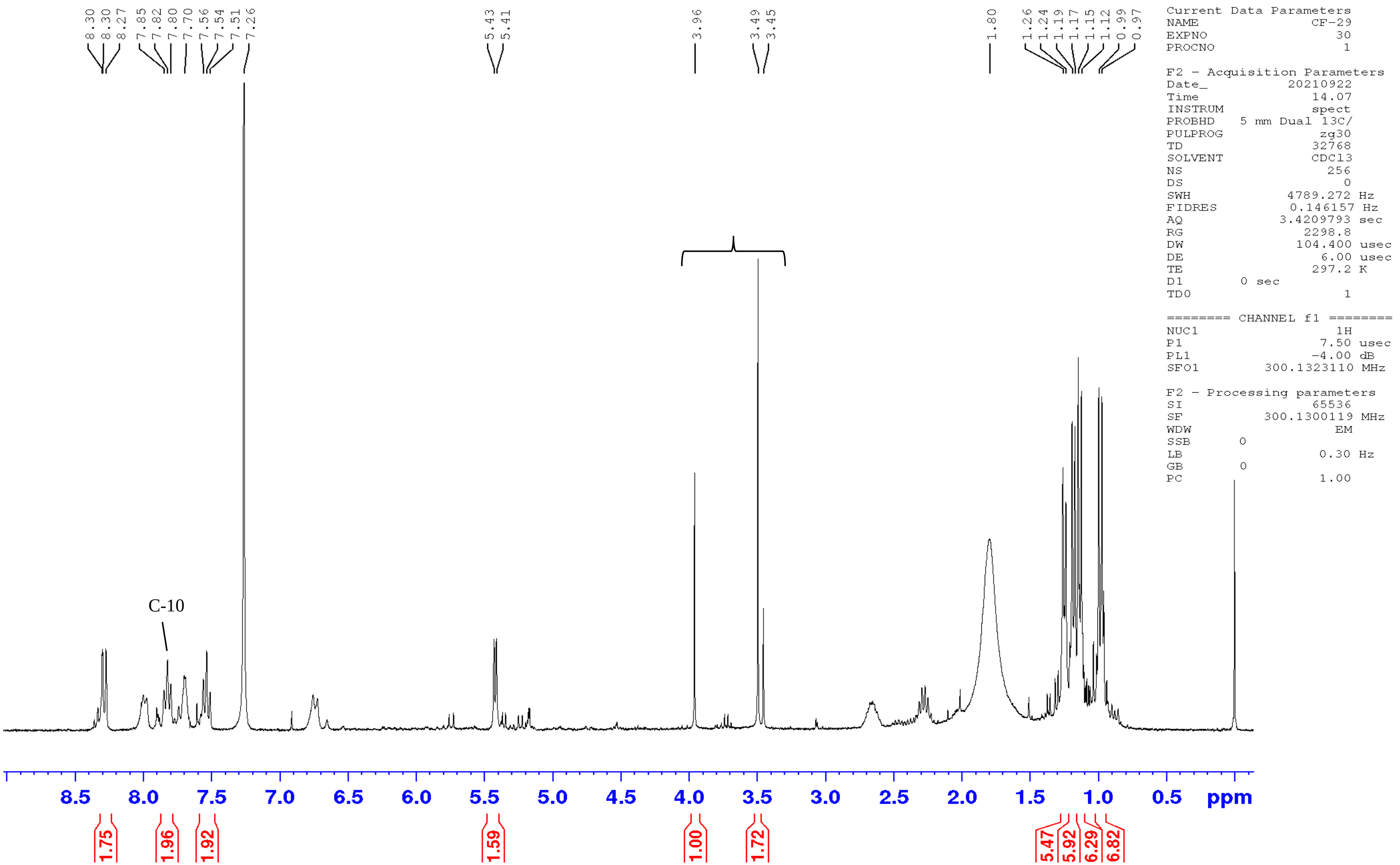

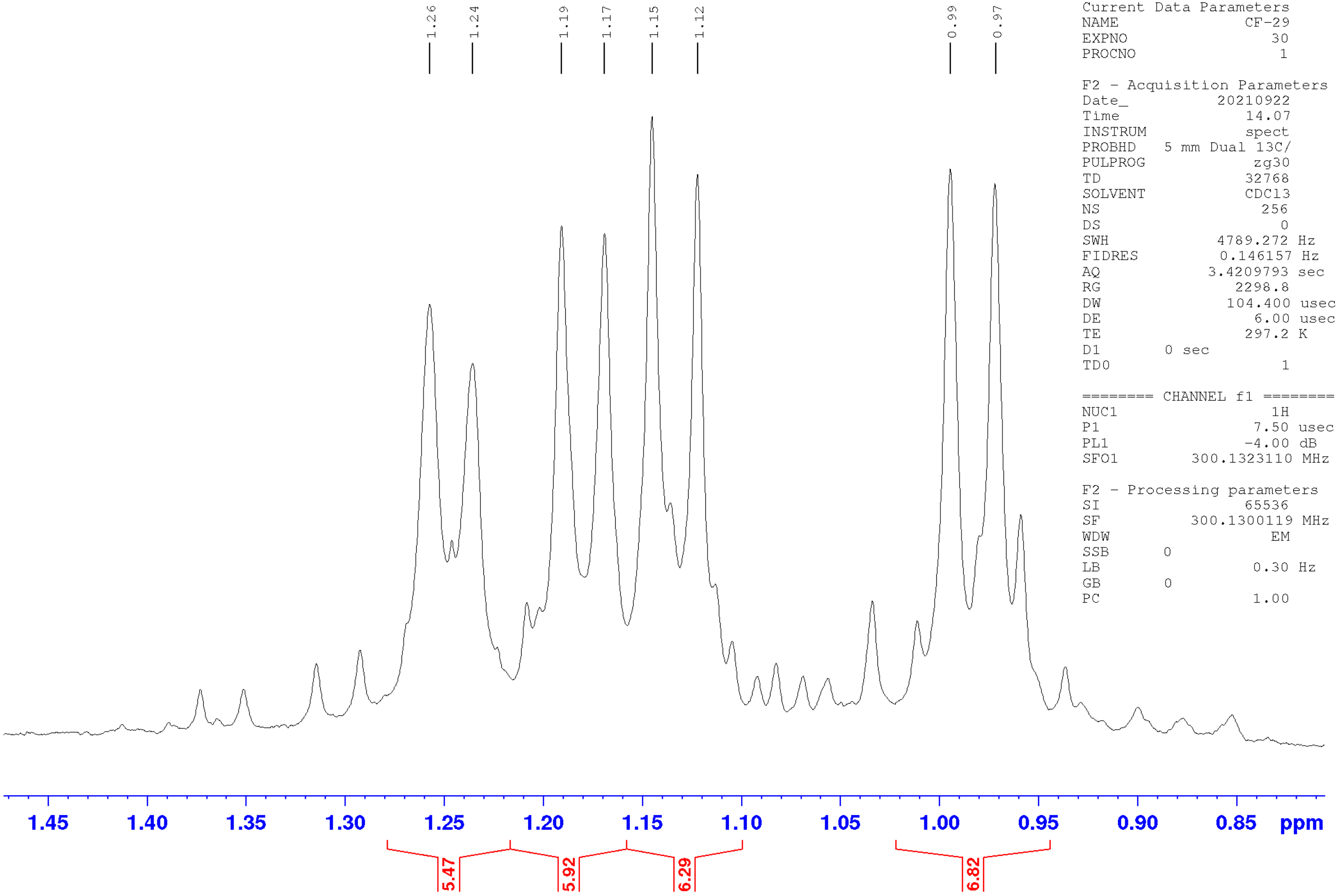

Current Data Parameters
NAME CF-29
EXPNO 30
PROCNO 1

F2 - Acquisition Parameters
Date_ 20210922
Time 14.07
INSTRUM spect
PROBHD 5 mm Dual 13C/
PULPROG zg30
TD 32768
SOLVENT CDCl3
NS 256
DS 0
SWH 4789.272 Hz
FIDRES 0.146157 Hz
AQ 3.4209793 sec
RG 2298.8
DW 104.400 usec
DE 6.00 usec
TE 297.2 K
D1 0 sec
TD0 1

======== CHANNEL f1 ========
NUC1 1H
P1 7.50 usec
PL1 -4.00 dB
SFO1 300.1323110 MHz

F2 - Processing parameters
SI 65536
SF 300.1300119 MHz
WDW EM
SSB 0
LB 0.30 Hz
GB 0
PC 1.00

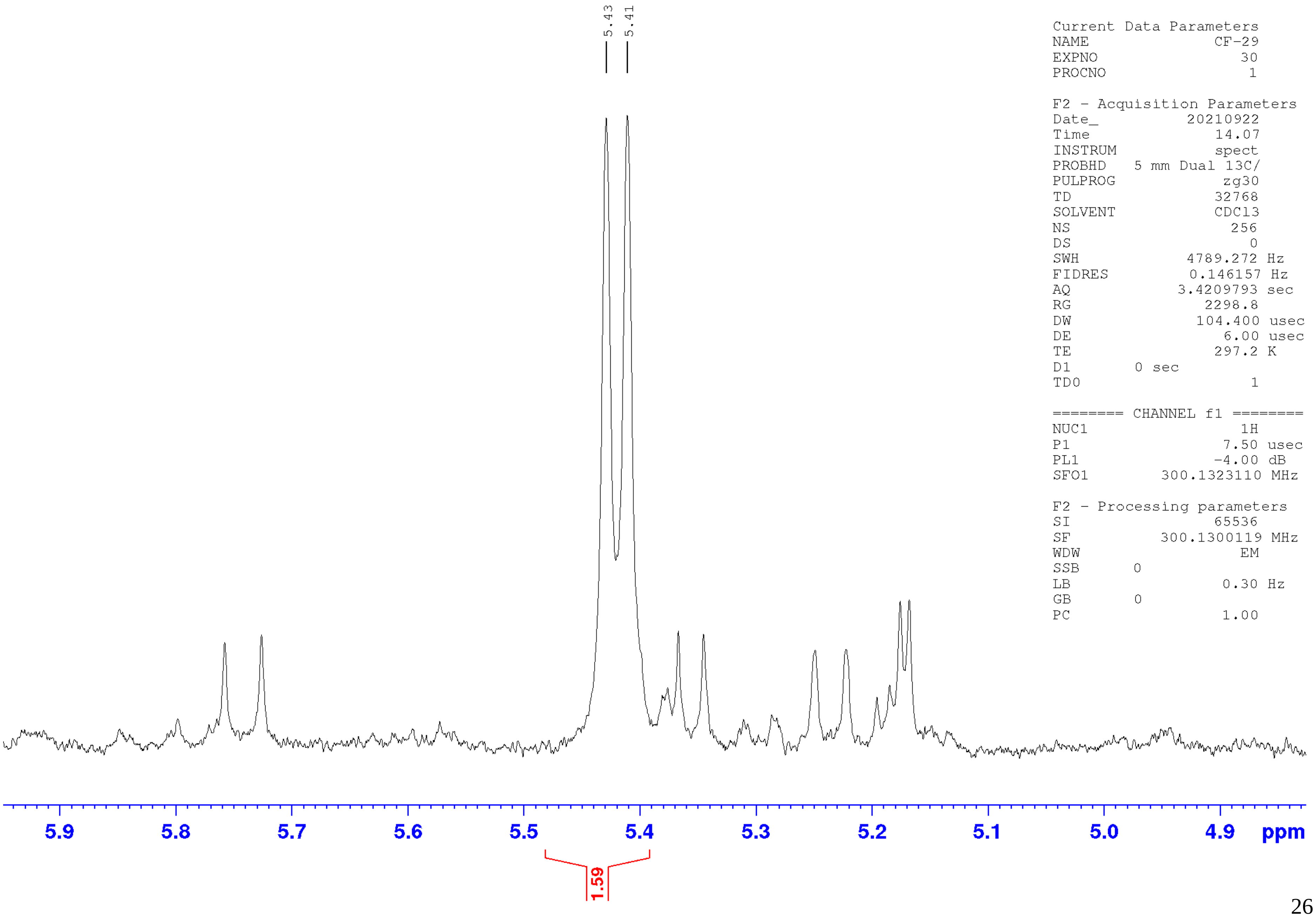
5.43
5.41
Current Data Parameters
NAME CF-29
EXPNO 30
PROCNO 1
F2 - Acquisition Parameters
Date_ 20210922
Time 14.07
INSTRUM spect
PROBHD 5 mm Dual 13C/
PULPROG zg30
TD 32768
SOLVENT CDCl3
NS 256
DS 0
SWH 4789.272 Hz
FIDRES 0.146157 Hz
AQ 3.4209793 sec
RG 2298.8
DW 104.400 usec
DE 6.00 usec
TE 297.2 K
D1 0 sec
TD0 1
======== CHANNEL f1 ========
NUC1 1H
P1 7.50 usec
PL1 -4.00 dB
SFO1 300.1323110 MHz
F2 - Processing parameters
SI 65536
SF 300.1300119 MHz
WDW EM
SSB 0
LB 0.30 Hz
GB 0
PC 1.00
5.9
5.8
5.7
5.6
5.5
5.4
5.3
5.2
5.1
5.0
4.9
ppm
1.59

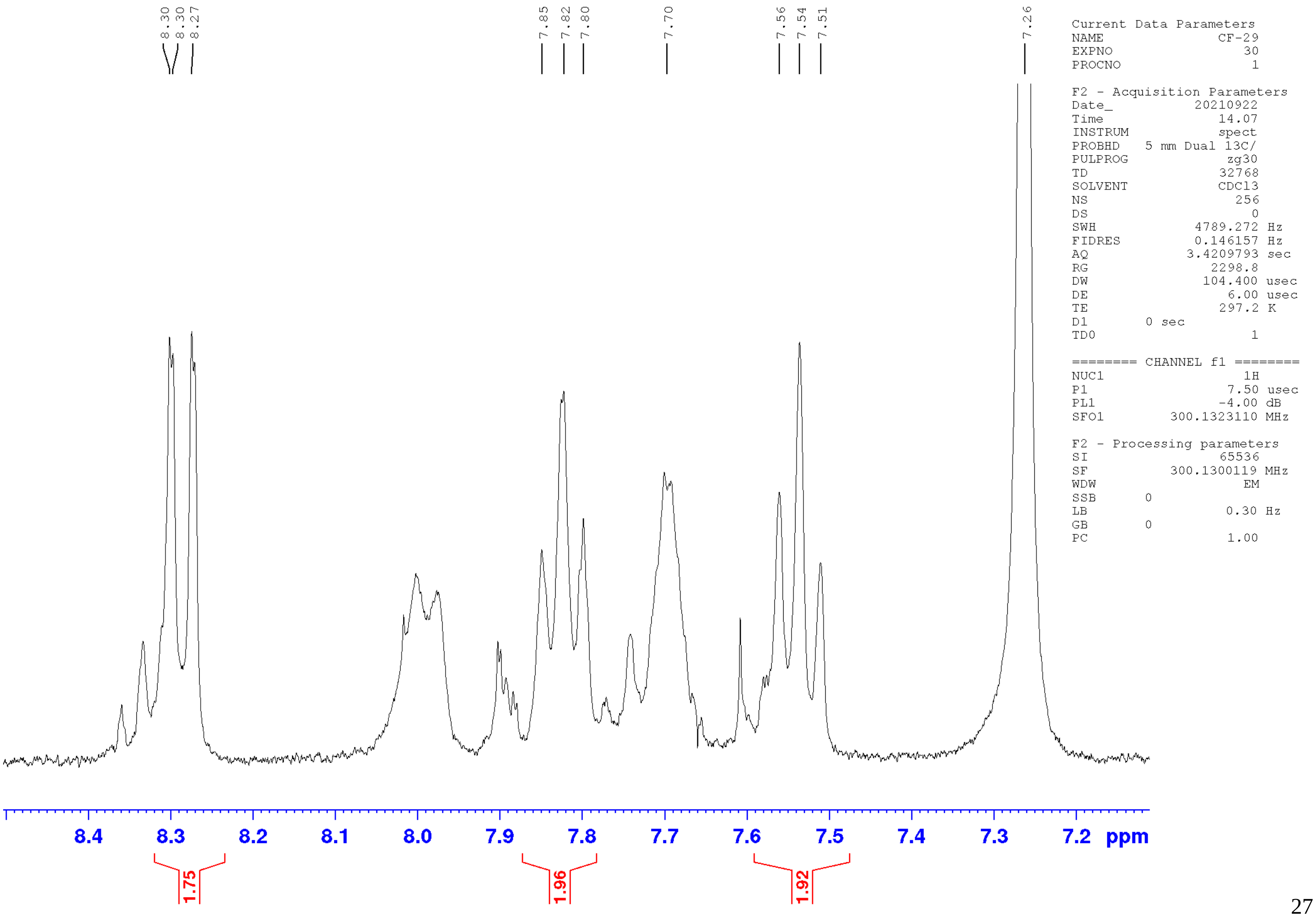
8.30 8.30 8.27
7.85 7.82 7.80
7.70
7.56 7.54 7.51
7.26
8.4 8.3 8.2 8.1 8.0 7.9 7.8 7.7 7.6 7.5 7.4 7.3 7.2 ppm
1.75
1.96
1.92
Current Data Parameters
NAME CF-29
EXPNO 30
PROCNO 1
F2 - Acquisition Parameters
Date_ 20210922
Time 14.07
INSTRUM spect
PROBHD 5 mm Dual 13C/
PULPROG zg30
TD 32768
SOLVENT CDCl3
NS 256
DS 0
SWH 4789.272 Hz
FIDRES 0.146157 Hz
AQ 3.4209793 sec
RG 2298.8
DW 104.400 usec
DE 6.00 usec
TE 297.2 K
D1 0 sec
TD0 1
======== CHANNEL f1 ========
NUC1 1H
P1 7.50 usec
PL1 -4.00 dB
SFO1 300.1323110 MHz
F2 - Processing parameters
SI 65536
SF 300.1300119 MHz
WDW EM
SSB 0
LB 0.30 Hz
GB 0
PC 1.00

Figure S15. ^{1}**H NMR spectrum of felicarnezoline D (4)**

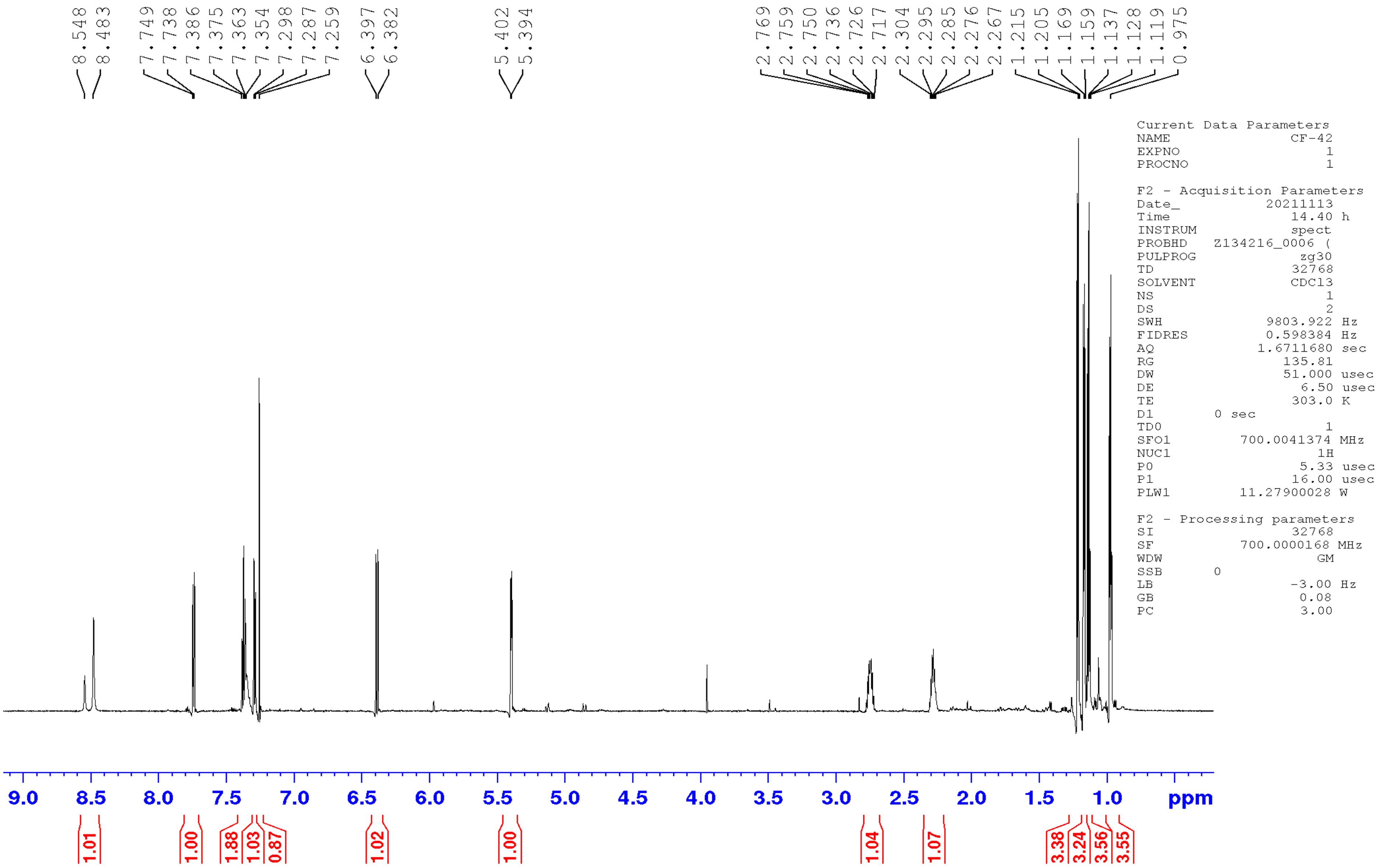

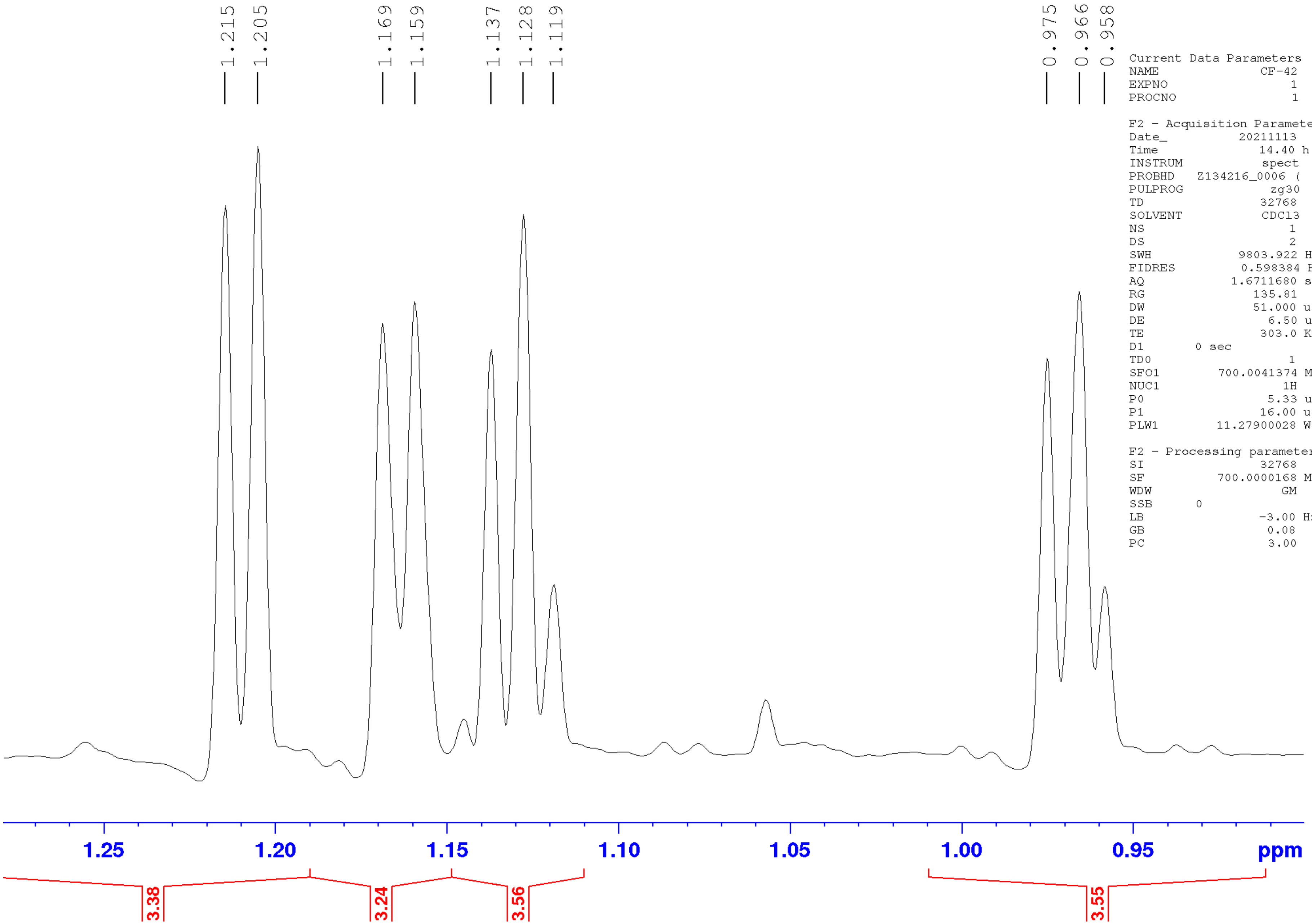


```
Current Data Parameters
NAME                CF-42
EXPNO                   1
PROCNO                  1

F2 - Acquisition Parameters
Date_            20211113
Time              14.40 h
INSTRUM             spect
PROBHD   Z134216_0006 (
PULPROG              zg30
TD                  32768
SOLVENT             CDCl3
NS                      1
DS                      2
SWH            9803.922 Hz
FIDRES         0.598384 Hz
AQ            1.6711680 sec
RG                 135.81
DW               51.000 usec
DE                 6.50 usec
TE                303.0 K
D1          0 sec
TD0                     1
SFO1        700.0041374 MHz
NUC1                   1H
P0                  5.33 usec
P1                 16.00 usec
PLW1        11.27900028 W

F2 - Processing parameters
SI                  32768
SF          700.0000168 MHz
WDW                    GM
SSB         0
LB                 -3.00 Hz
GB                   0.08
PC                   3.00
```

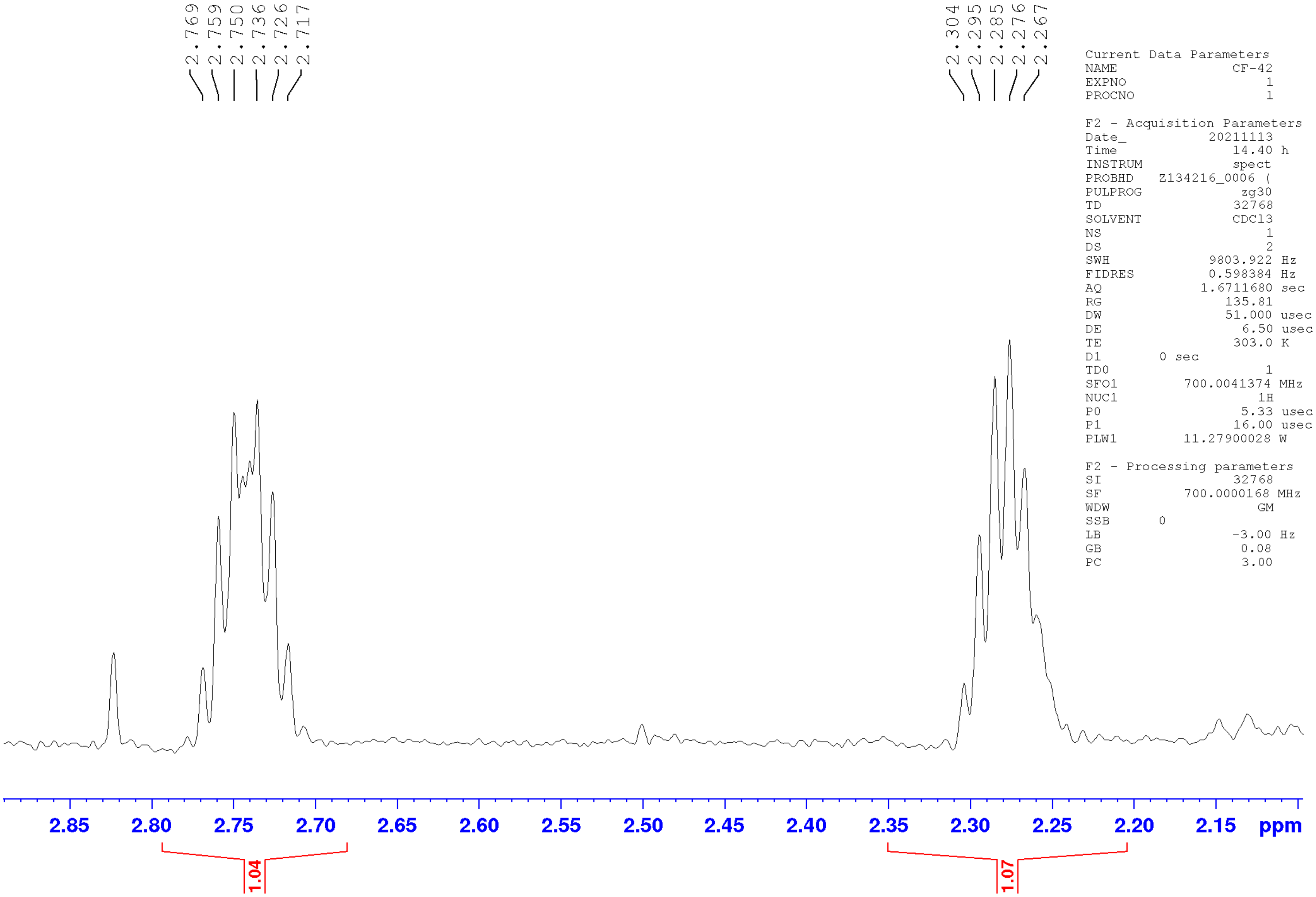


```
Current Data Parameters
NAME                CF-42
EXPNO                   1
PROCNO                  1

F2 - Acquisition Parameters
Date_            20211113
Time                14.40 h
INSTRUM             spect
PROBHD   Z134216_0006 (
PULPROG              zg30
TD                  32768
SOLVENT             CDCl3
NS                      1
DS                      2
SWH              9803.922 Hz
FIDRES           0.598384 Hz
AQ              1.6711680 sec
RG                 135.81
DW                 51.000 usec
DE                   6.50 usec
TE                  303.0 K
D1         0 sec
TD0                     1
SFO1          700.0041374 MHz
NUC1                   1H
P0                   5.33 usec
P1                  16.00 usec
PLW1          11.27900028 W

F2 - Processing parameters
SI                  32768
SF            700.0000168 MHz
WDW                    GM
SSB        0
LB                  -3.00 Hz
GB                   0.08
PC                   3.00
```

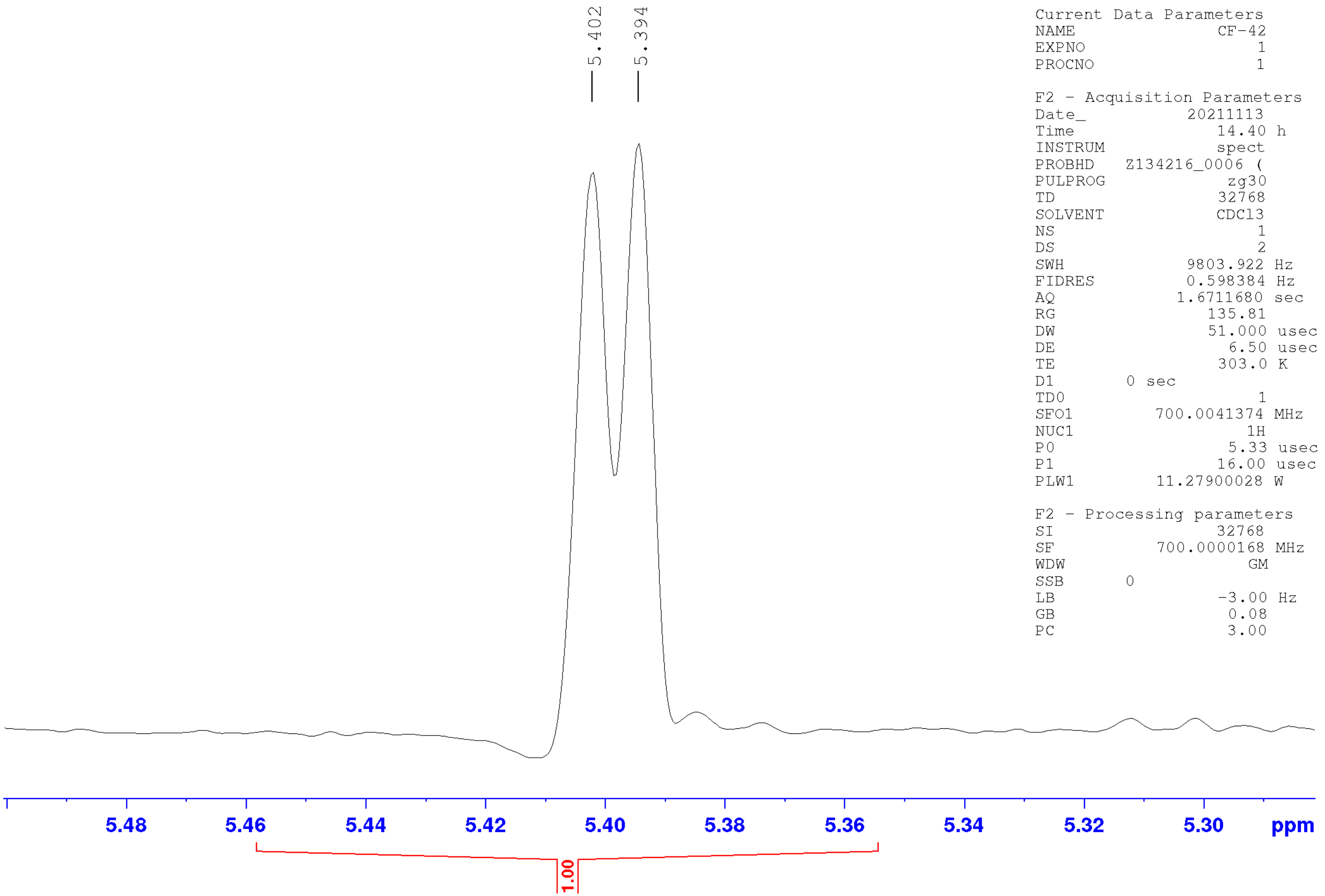
5.402
5.394
Current Data Parameters
NAME CF-42
EXPNO 1
PROCNO 1
F2 - Acquisition Parameters
Date_ 20211113
Time 14.40 h
INSTRUM spect
PROBHD Z134216_0006 (
PULPROG zg30
TD 32768
SOLVENT CDCl3
NS 1
DS 2
SWH 9803.922 Hz
FIDRES 0.598384 Hz
AQ 1.6711680 sec
RG 135.81
DW 51.000 usec
DE 6.50 usec
TE 303.0 K
D1 0 sec
TD0 1
SFO1 700.0041374 MHz
NUC1 1H
P0 5.33 usec
P1 16.00 usec
PLW1 11.27900028 W
F2 - Processing parameters
SI 32768
SF 700.0000168 MHz
WDW GM
SSB 0
LB -3.00 Hz
GB 0.08
PC 3.00
5.48
5.46
5.44
5.42
5.40
5.38
5.36
5.34
5.32
5.30
ppm
1.00

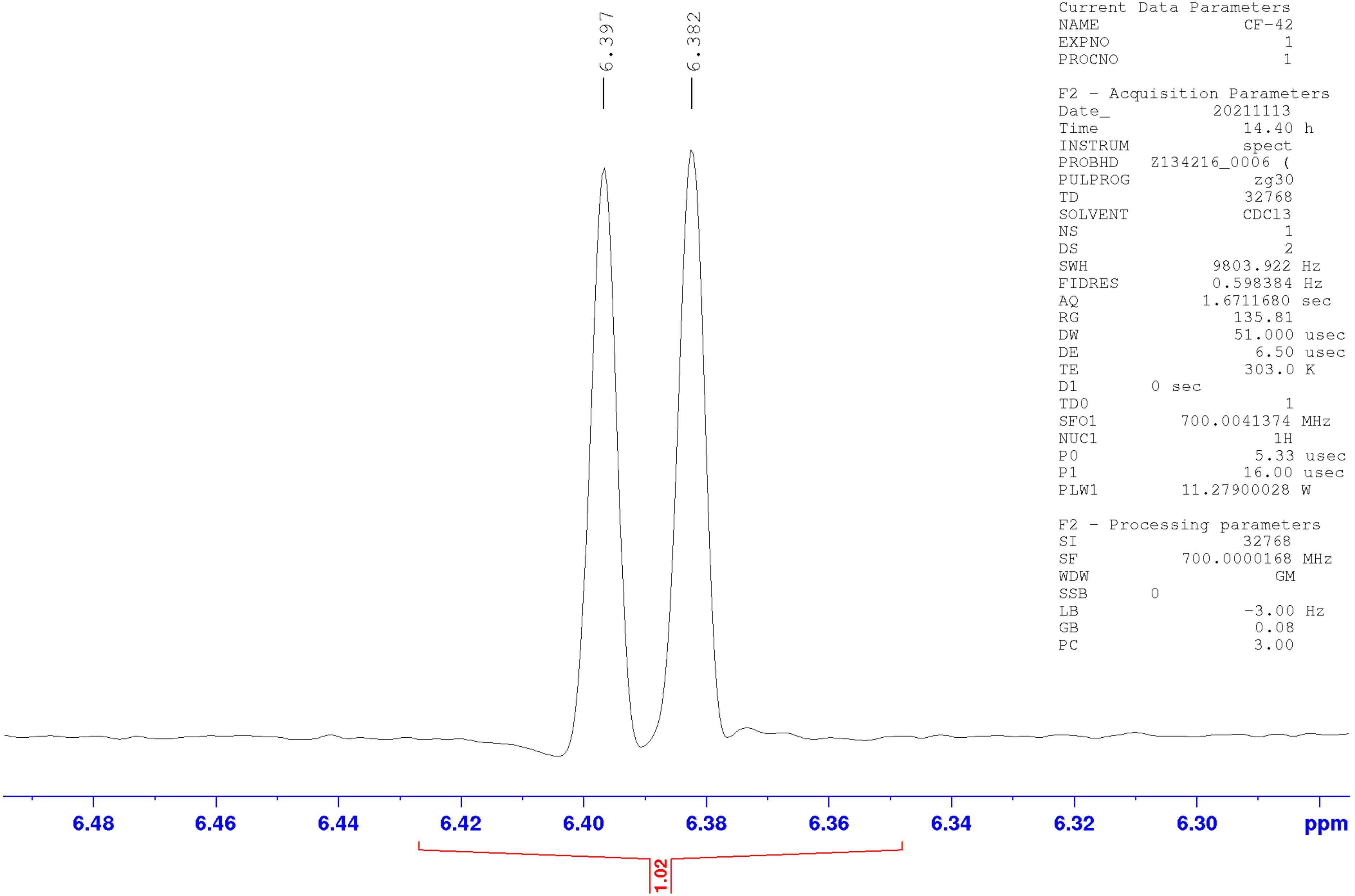
6.397
6.382
Current Data Parameters
NAME CF-42
EXPNO 1
PROCNO 1
F2 - Acquisition Parameters
Date_ 20211113
Time 14.40 h
INSTRUM spect
PROBHD Z134216_0006 (
PULPROG zg30
TD 32768
SOLVENT CDCl3
NS 1
DS 2
SWH 9803.922 Hz
FIDRES 0.598384 Hz
AQ 1.6711680 sec
RG 135.81
DW 51.000 usec
DE 6.50 usec
TE 303.0 K
D1 0 sec
TD0 1
SFO1 700.0041374 MHz
NUC1 1H
P0 5.33 usec
P1 16.00 usec
PLW1 11.27900028 W
F2 - Processing parameters
SI 32768
SF 700.0000168 MHz
WDW GM
SSB 0
LB -3.00 Hz
GB 0.08
PC 3.00
6.48
6.46
6.44
6.42
6.40
6.38
6.36
6.34
6.32
6.30
ppm
1.02

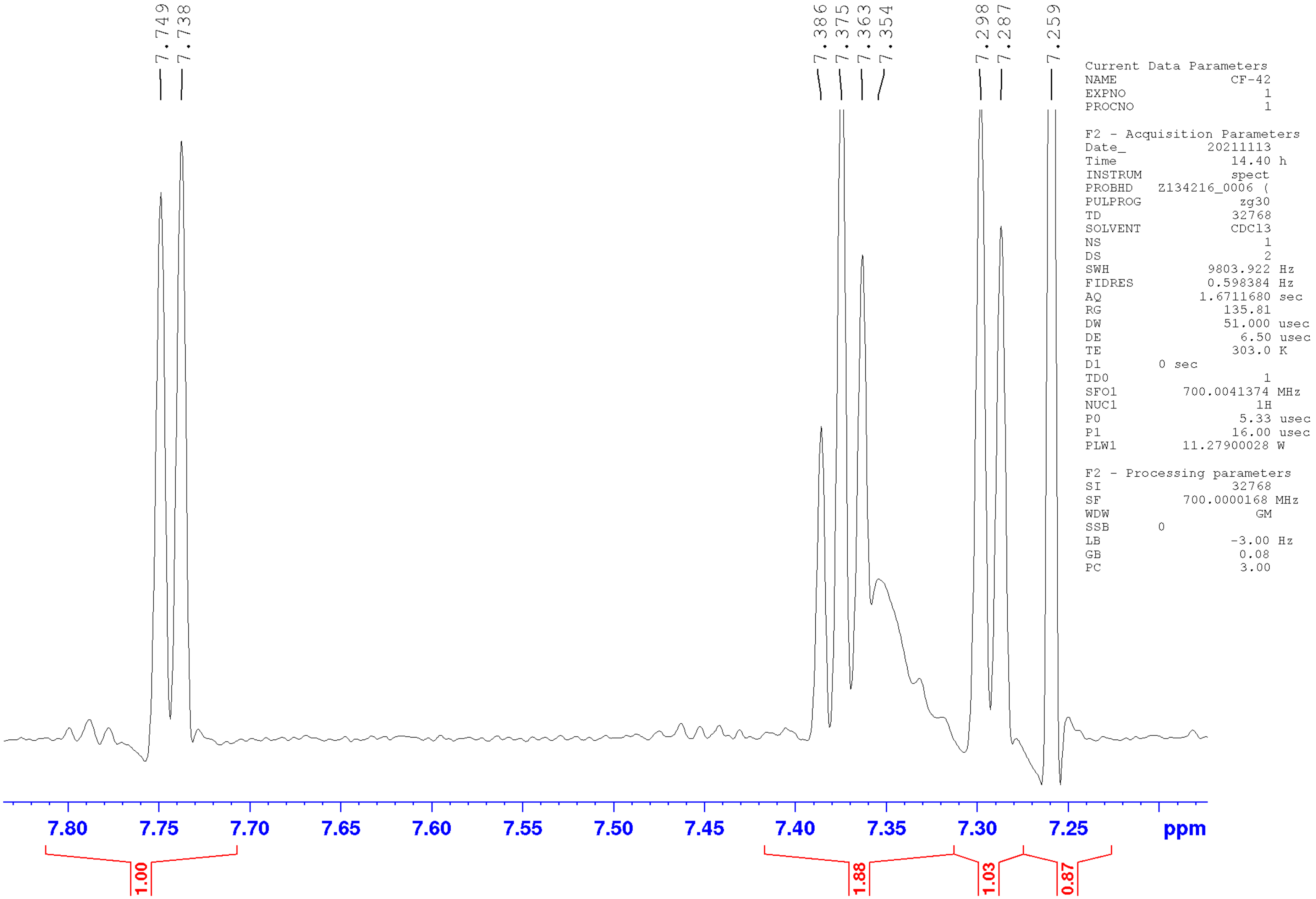
7.749
7.738
7.386
7.375
7.363
7.354
7.298
7.287
7.259
Current Data Parameters
NAME CF-42
EXPNO 1
PROCNO 1
F2 - Acquisition Parameters
Date_ 20211113
Time 14.40 h
INSTRUM spect
PROBHD Z134216_0006 (
PULPROG zg30
TD 32768
SOLVENT CDCl3
NS 1
DS 2
SWH 9803.922 Hz
FIDRES 0.598384 Hz
AQ 1.6711680 sec
RG 135.81
DW 51.000 usec
DE 6.50 usec
TE 303.0 K
D1 0 sec
TD0 1
SFO1 700.0041374 MHz
NUC1 1H
P0 5.33 usec
P1 16.00 usec
PLW1 11.27900028 W
F2 - Processing parameters
SI 32768
SF 700.0000168 MHz
WDW GM
SSB 0
LB -3.00 Hz
GB 0.08
PC 3.00
7.80
7.75
7.70
7.65
7.60
7.55
7.50
7.45
7.40
7.35
7.30
7.25
ppm
1.00
1.88
1.03
0.87

Figure S16. ¹H NMR spectrum of felicarnezoline E (5)

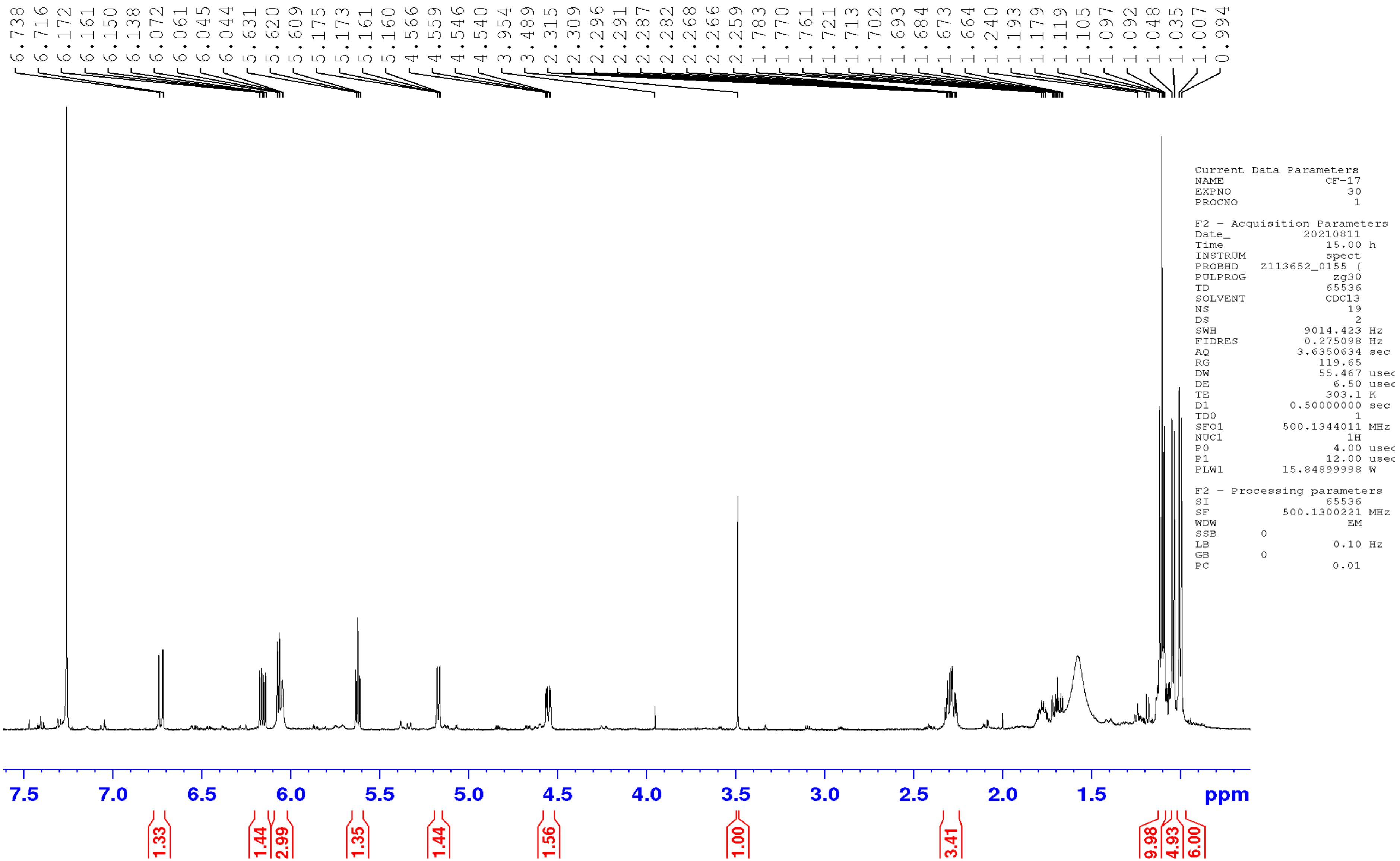

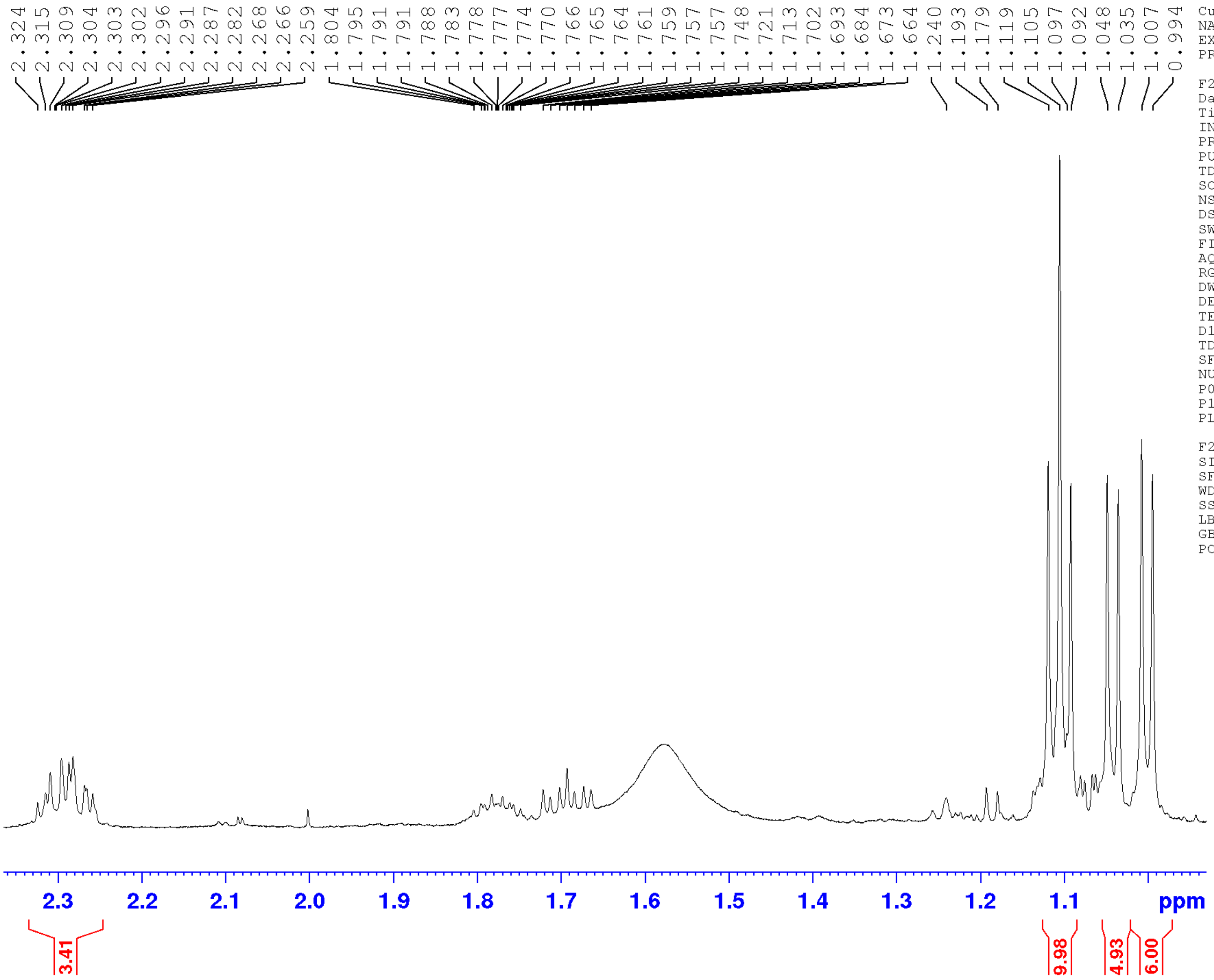

Current Data Parameters
NAME CF-17
EXPNO 30
PROCNO 1

F2 - Acquisition Parameters
Date_ 20210811
Time 15.00 h
INSTRUM spect
PROBHD Z113652_0155 (
PULPROG zg30
TD 65536
SOLVENT CDCl3
NS 19
DS 2
SWH 9014.423 Hz
FIDRES 0.275098 Hz
AQ 3.6350634 sec
RG 119.65
DW 55.467 usec
DE 6.50 usec
TE 303.1 K
D1 0.50000000 sec
TD0 1
SFO1 500.1344011 MHz
NUC1 1H
P0 4.00 usec
P1 12.00 usec
PLW1 15.84899998 W

F2 - Processing parameters
SI 65536
SF 500.1300221 MHz
WDW EM
SSB 0
LB 0.10 Hz
GB 0
PC 0.01

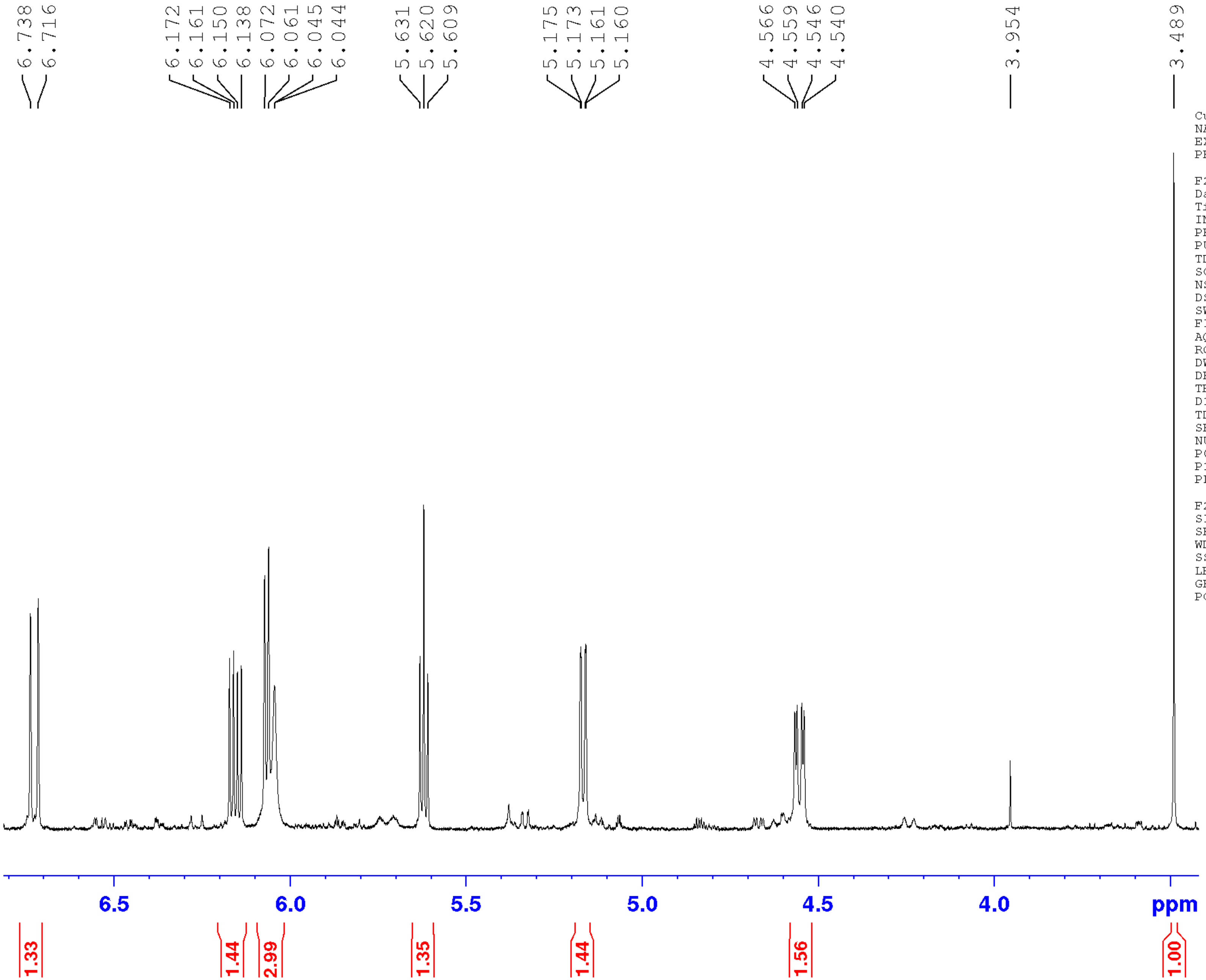

Current Data Parameters
NAME CF-17
EXPNO 30
PROCNO 1

F2 - Acquisition Parameters
Date_ 20210811
Time 15.00 h
INSTRUM spect
PROBHD Z113652_0155 (
PULPROG zg30
TD 65536
SOLVENT CDCl3
NS 19
DS 2
SWH 9014.423 Hz
FIDRES 0.275098 Hz
AQ 3.6350634 sec
RG 119.65
DW 55.467 usec
DE 6.50 usec
TE 303.1 K
D1 0.50000000 sec
TD0 1
SFO1 500.1344011 MHz
NUC1 1H
P0 4.00 usec
P1 12.00 usec
PLW1 15.84899998 W

F2 - Processing parameters
SI 65536
SF 500.1300221 MHz
WDW EM
SSB 0
LB 0.10 Hz
GB 0
PC 0.01

Figure S17. ^{1}H **NMR spectrum of oxyrapentin M (6)**

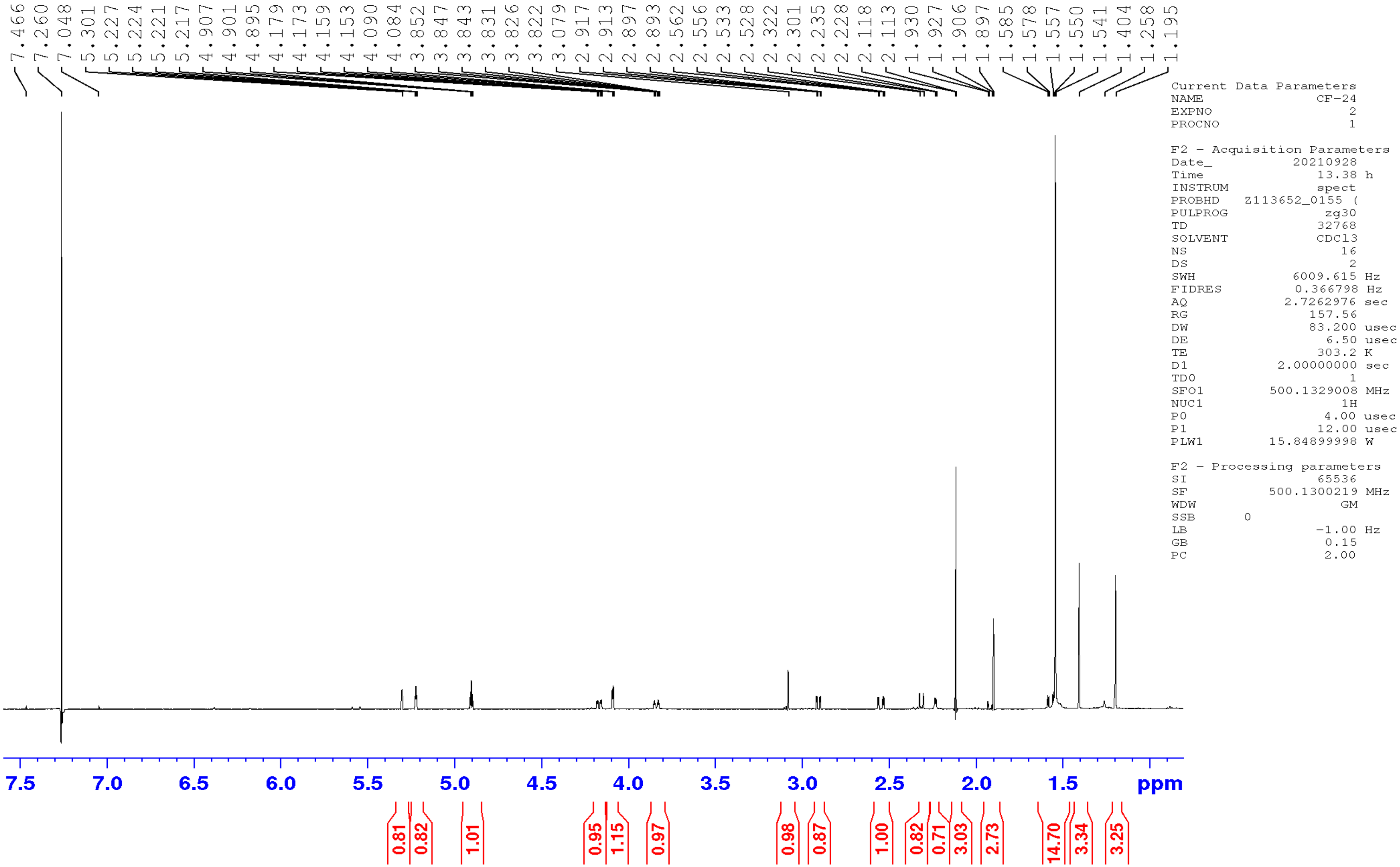

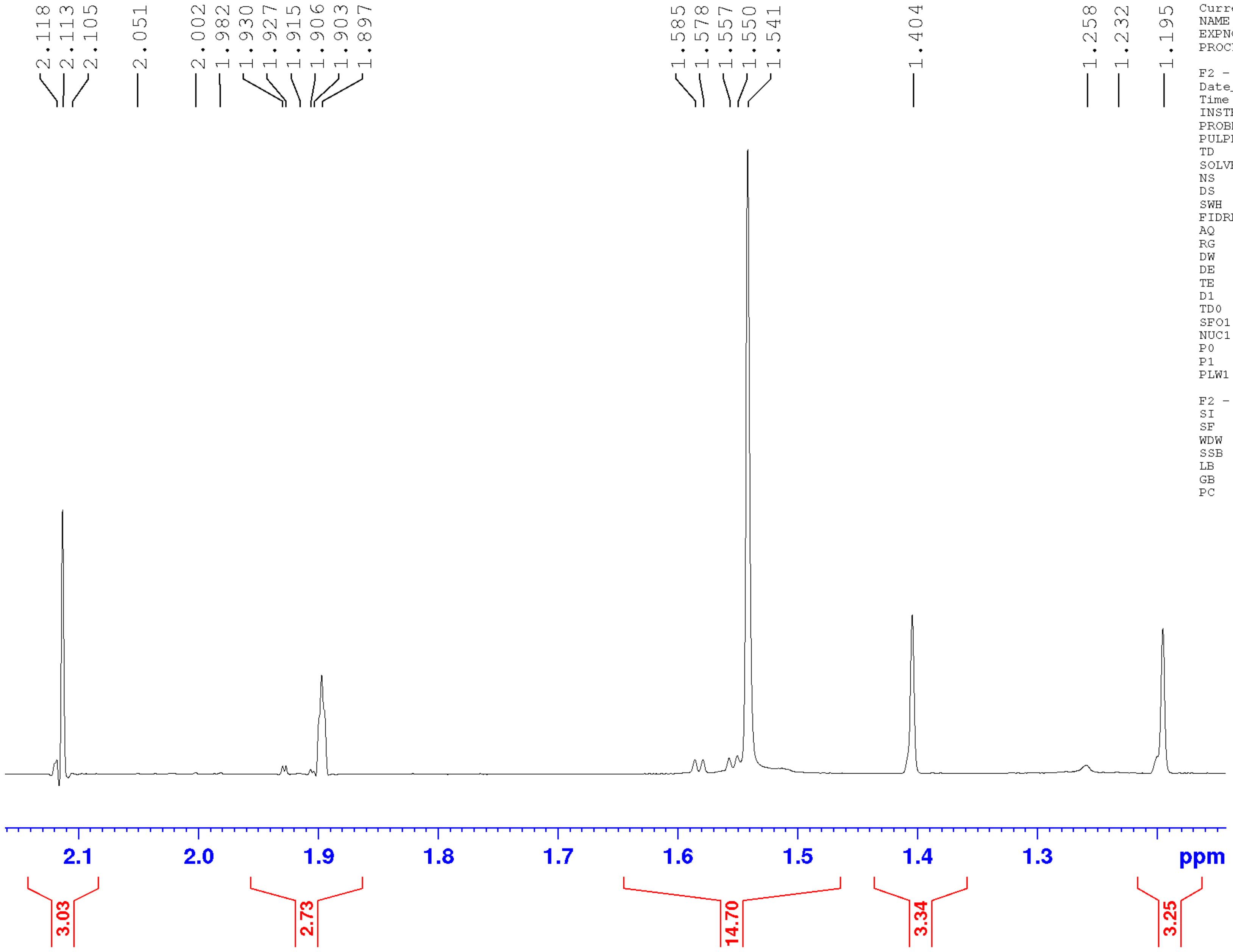


```
Current Data Parameters
NAME                CF-24
EXPNO                   2
PROCNO                  1

F2 - Acquisition Parameters
Date_            20210928
Time              13.38 h
INSTRUM             spect
PROBHD    Z113652_0155 (
PULPROG              zg30
TD                  32768
SOLVENT             CDCl3
NS                     16
DS                      2
SWH              6009.615 Hz
FIDRES           0.366798 Hz
AQ              2.7262976 sec
RG                 157.56
DW                 83.200 usec
DE                   6.50 usec
TE                  303.2 K
D1             2.00000000 sec
TD0                     1
SFO1          500.1329008 MHz
NUC1                   1H
P0                   4.00 usec
P1                  12.00 usec
PLW1          15.84899998 W

F2 - Processing parameters
SI                  65536
SF            500.1300219 MHz
WDW                    GM
SSB      0
LB                  -1.00 Hz
GB                   0.15
PC                   2.00
```

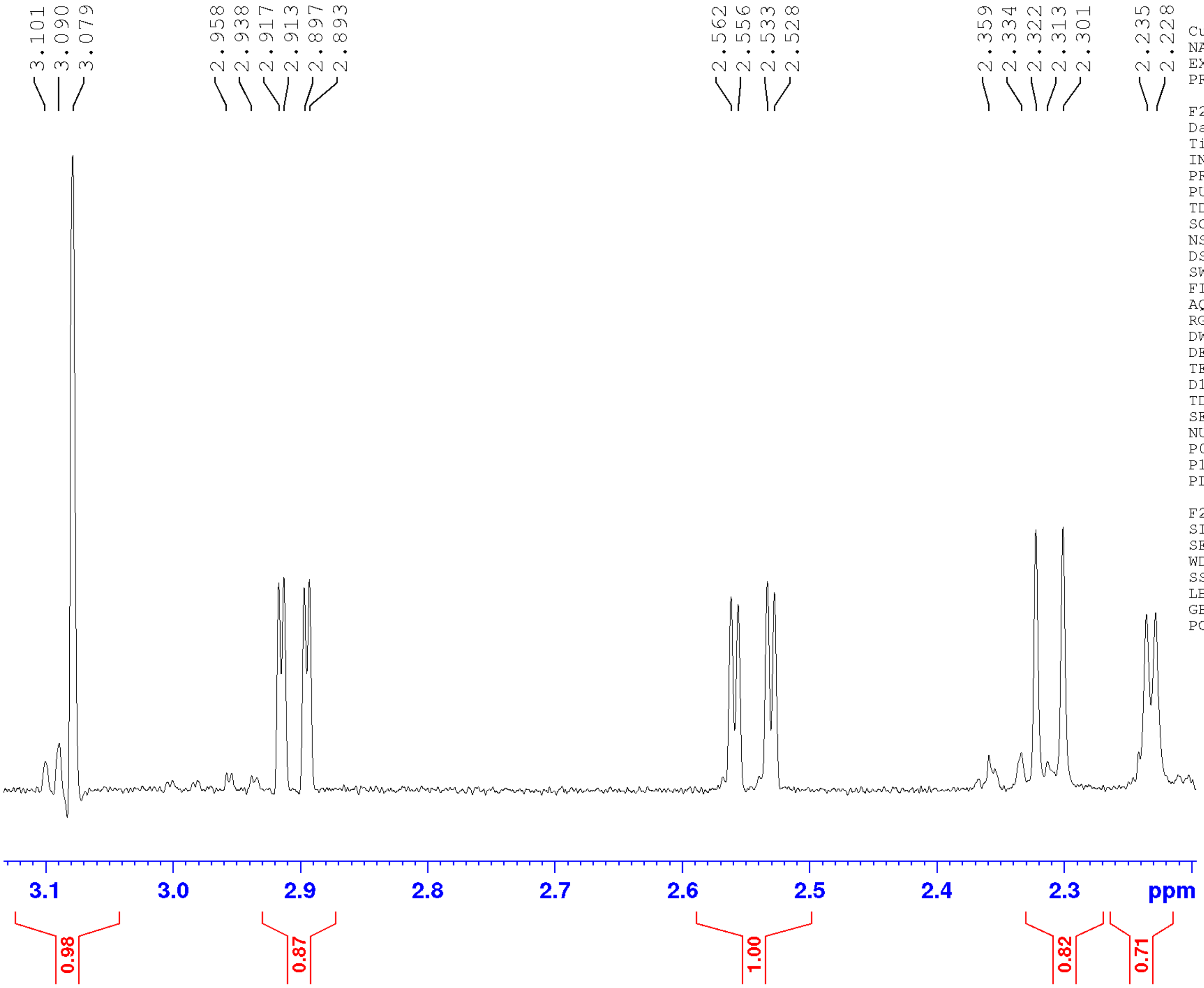

Current Data Parameters
NAME CF-24
EXPNO 2
PROCNO 1

F2 - Acquisition Parameters
Date_ 20210928
Time 13.38 h
INSTRUM spect
PROBHD Z113652_0155 (
PULPROG zg30
TD 32768
SOLVENT CDCl3
NS 16
DS 2
SWH 6009.615 Hz
FIDRES 0.366798 Hz
AQ 2.7262976 sec
RG 157.56
DW 83.200 usec
DE 6.50 usec
TE 303.2 K
D1 2.00000000 sec
TD0 1
SFO1 500.1329008 MHz
NUC1 1H
P0 4.00 usec
P1 12.00 usec
PLW1 15.84899998 W

F2 - Processing parameters
SI 65536
SF 500.1300219 MHz
WDW GM
SSB 0
LB -1.00 Hz
GB 0.15
PC 2.00

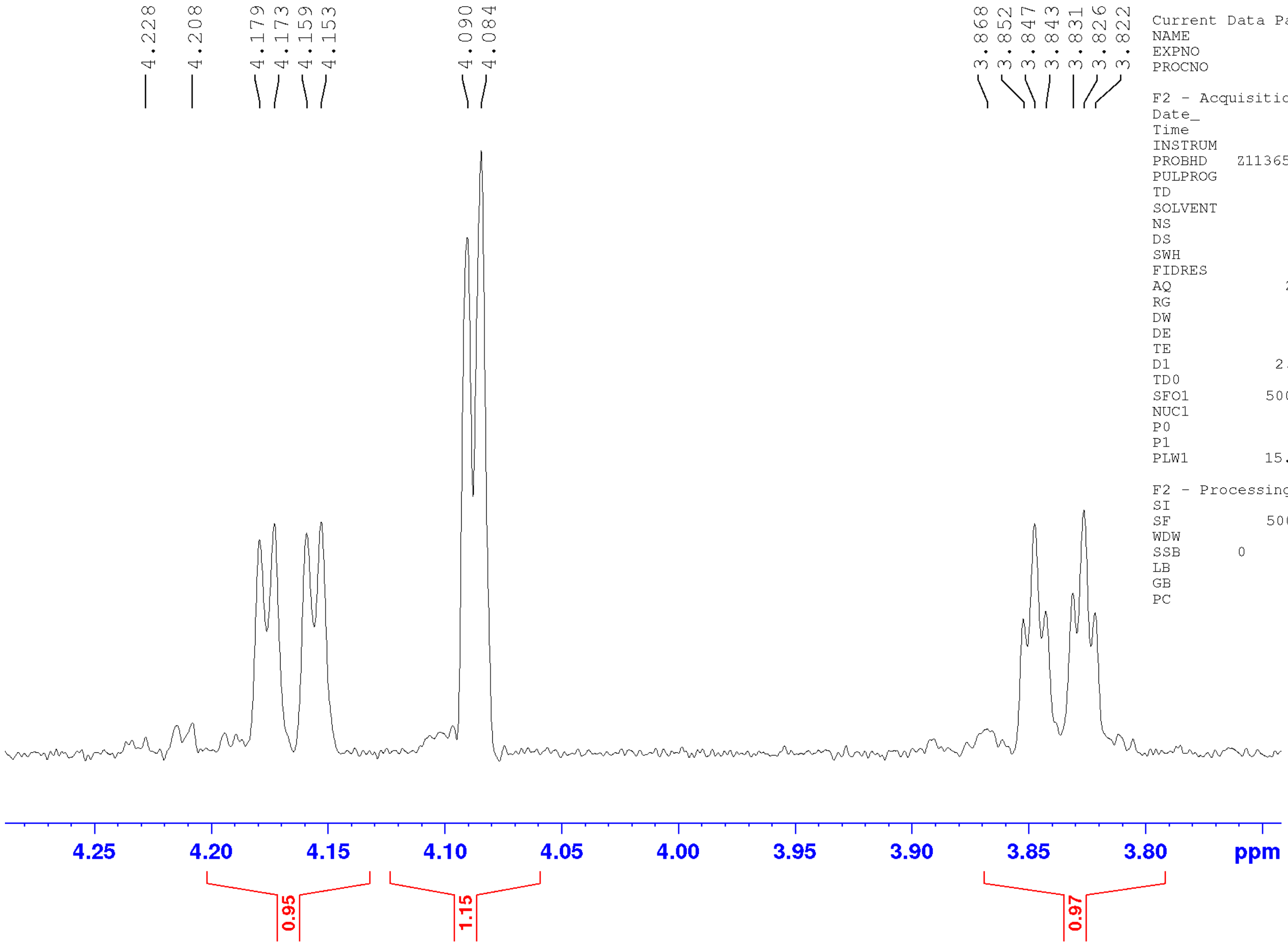

Current Data Parameters
NAME CF-24
EXPNO 2
PROCNO 1

F2 - Acquisition Parameters
Date_ 20210928
Time 13.38 h
INSTRUM spect
PROBHD Z113652_0155 (
PULPROG zg30
TD 32768
SOLVENT CDCl3
NS 16
DS 2
SWH 6009.615 Hz
FIDRES 0.366798 Hz
AQ 2.7262976 sec
RG 157.56
DW 83.200 usec
DE 6.50 usec
TE 303.2 K
D1 2.00000000 sec
TD0 1
SFO1 500.1329008 MHz
NUC1 1H
P0 4.00 usec
P1 12.00 usec
PLW1 15.84899998 W

F2 - Processing parameters
SI 65536
SF 500.1300219 MHz
WDW GM
SSB 0
LB -1.00 Hz
GB 0.15
PC 2.00

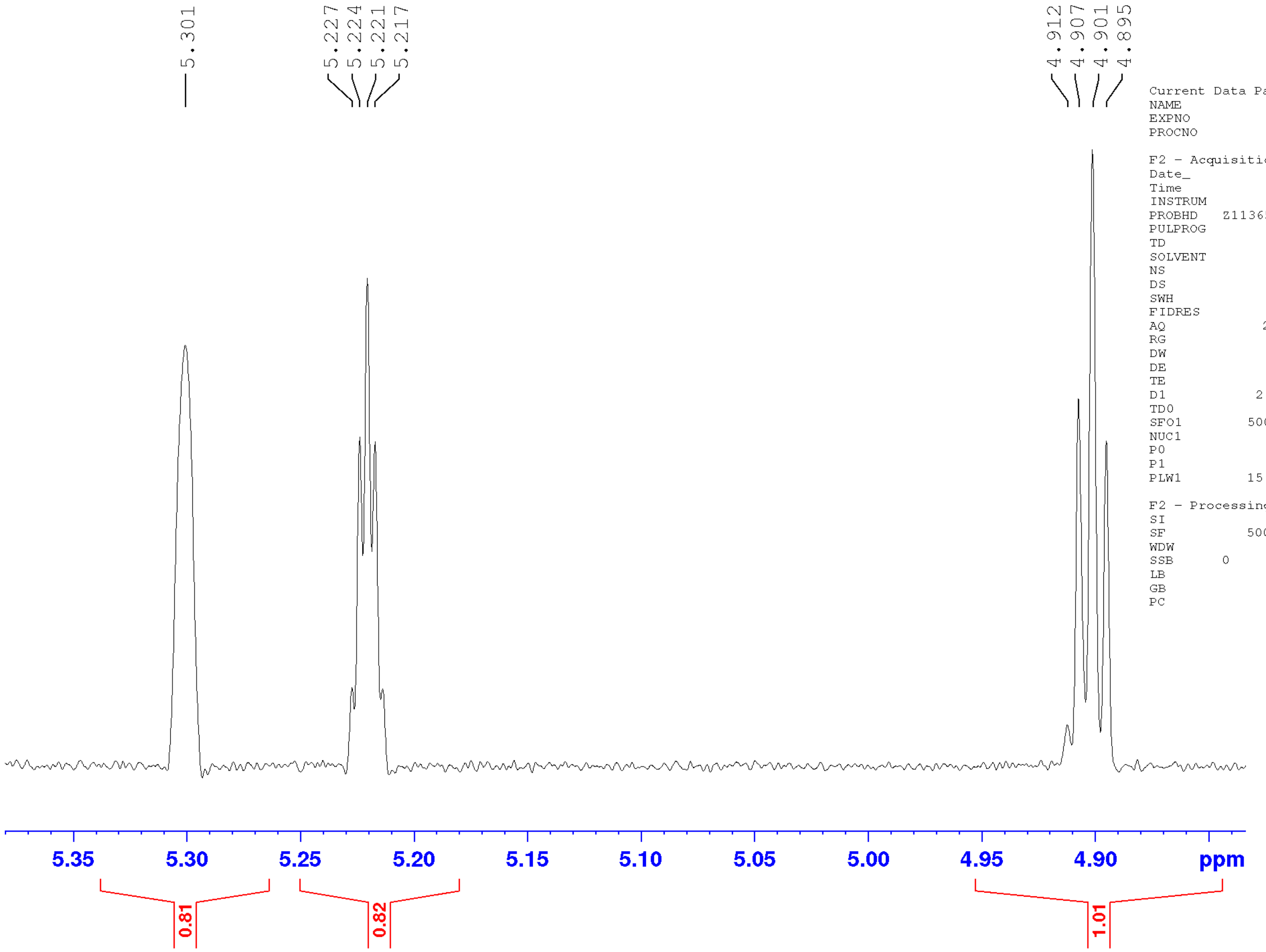

Current Data Parameters
NAME CF-24
EXPNO 2
PROCNO 1

F2 - Acquisition Parameters
Date_ 20210928
Time 13.38 h
INSTRUM spect
PROBHD Z113652_0155 (
PULPROG zg30
TD 32768
SOLVENT CDCl3
NS 16
DS 2
SWH 6009.615 Hz
FIDRES 0.366798 Hz
AQ 2.7262976 sec
RG 157.56
DW 83.200 usec
DE 6.50 usec
TE 303.2 K
D1 2.00000000 sec
TD0 1
SFO1 500.1329008 MHz
NUC1 1H
P0 4.00 usec
P1 12.00 usec
PLW1 15.84899998 W

F2 - Processing parameters
SI 65536
SF 500.1300219 MHz
WDW GM
SSB 0
LB -1.00 Hz
GB 0.15
PC 2.00

Figure S18. ^{13}C NMR spectrum of oxyrapentin M (6)

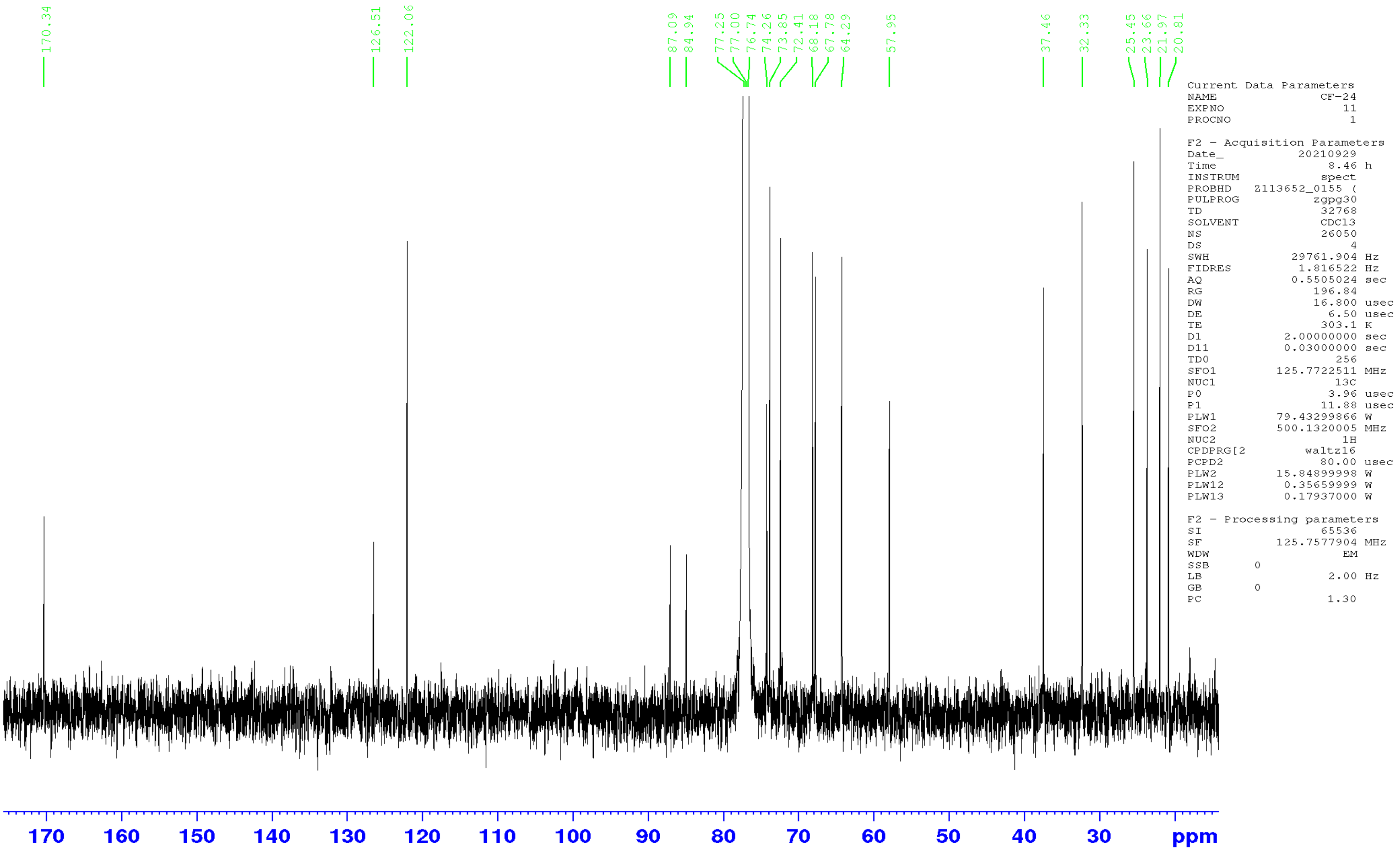

Figure S19. DEPT-135 spectrum of oxyrapentin M (6)

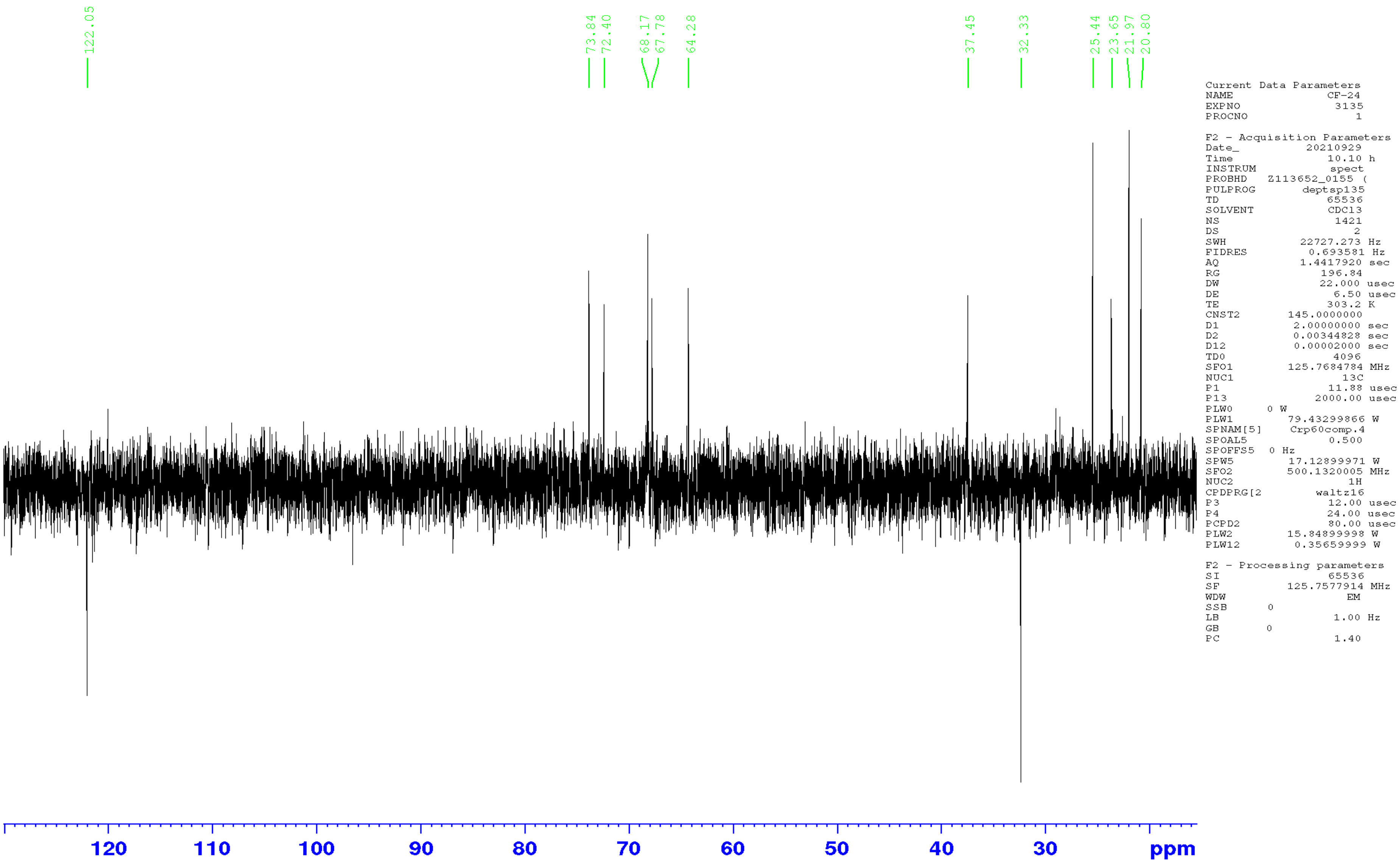

Figure S20. ^{1}H-^{1}H COSY spectrum of oxyrapentin M (6)

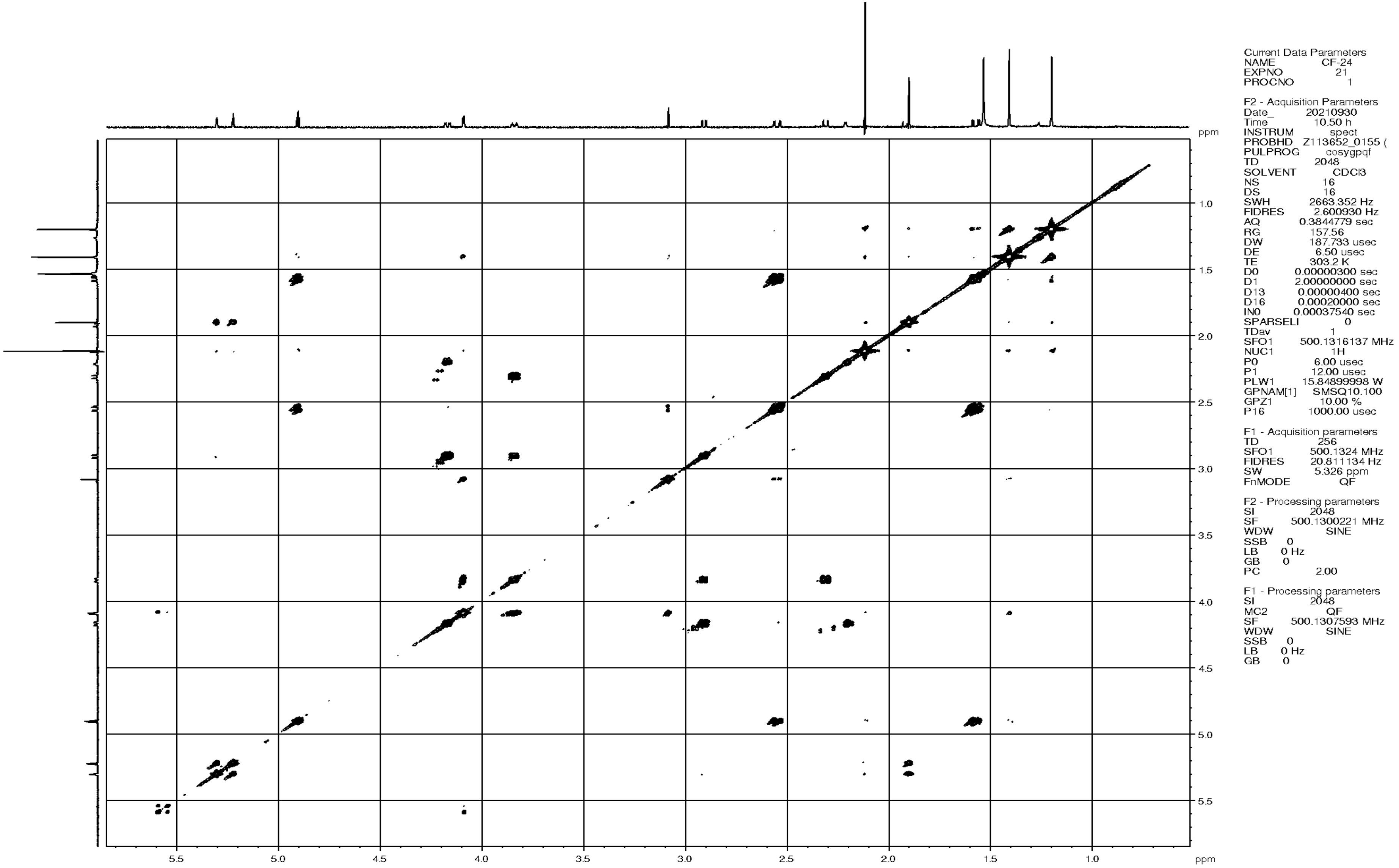

Figure S21. HSQC spectrum of oxyrapentin M (6)

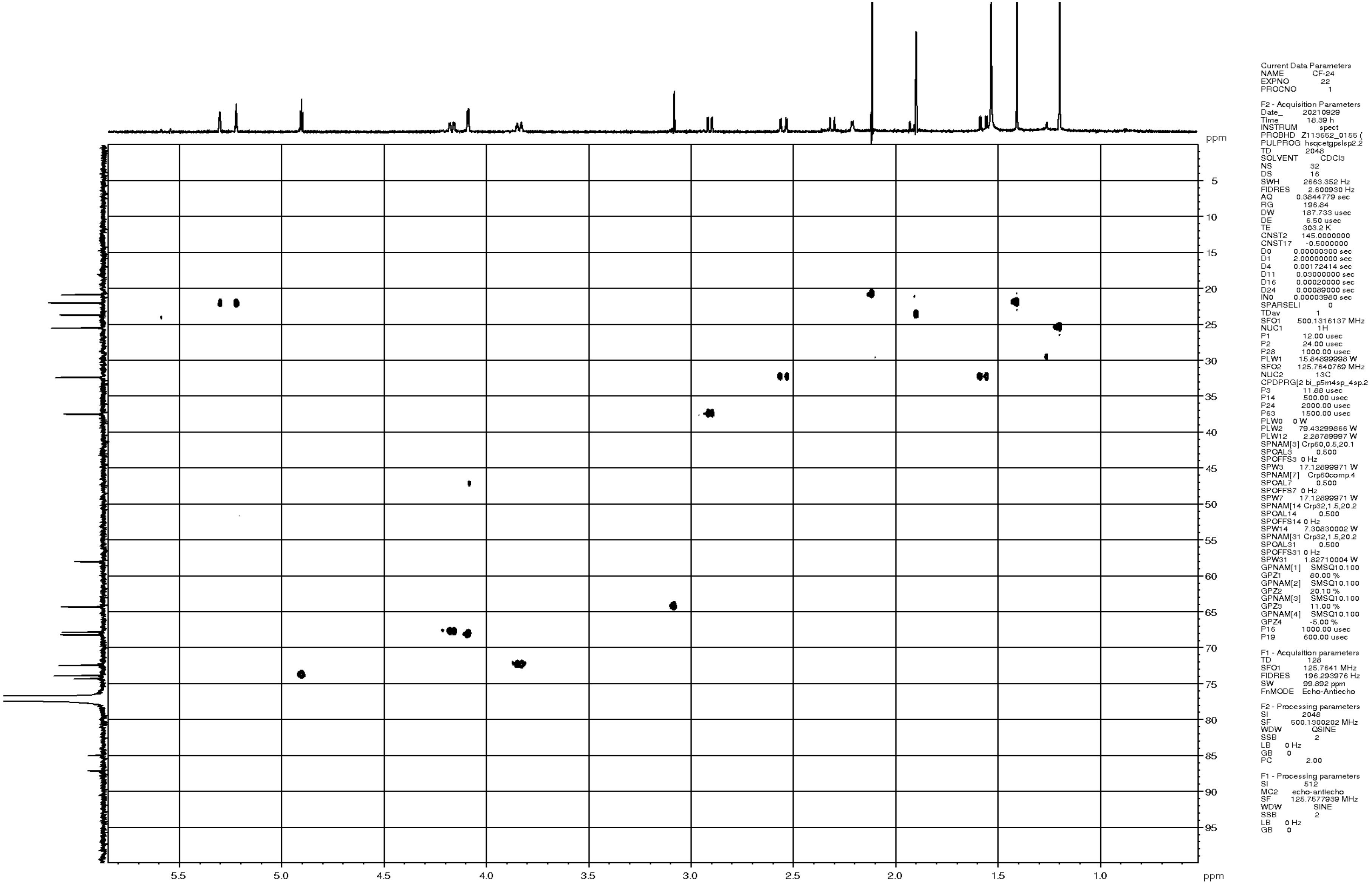

Figure S22. HMBC spectrum of oxyrapentin M (6)

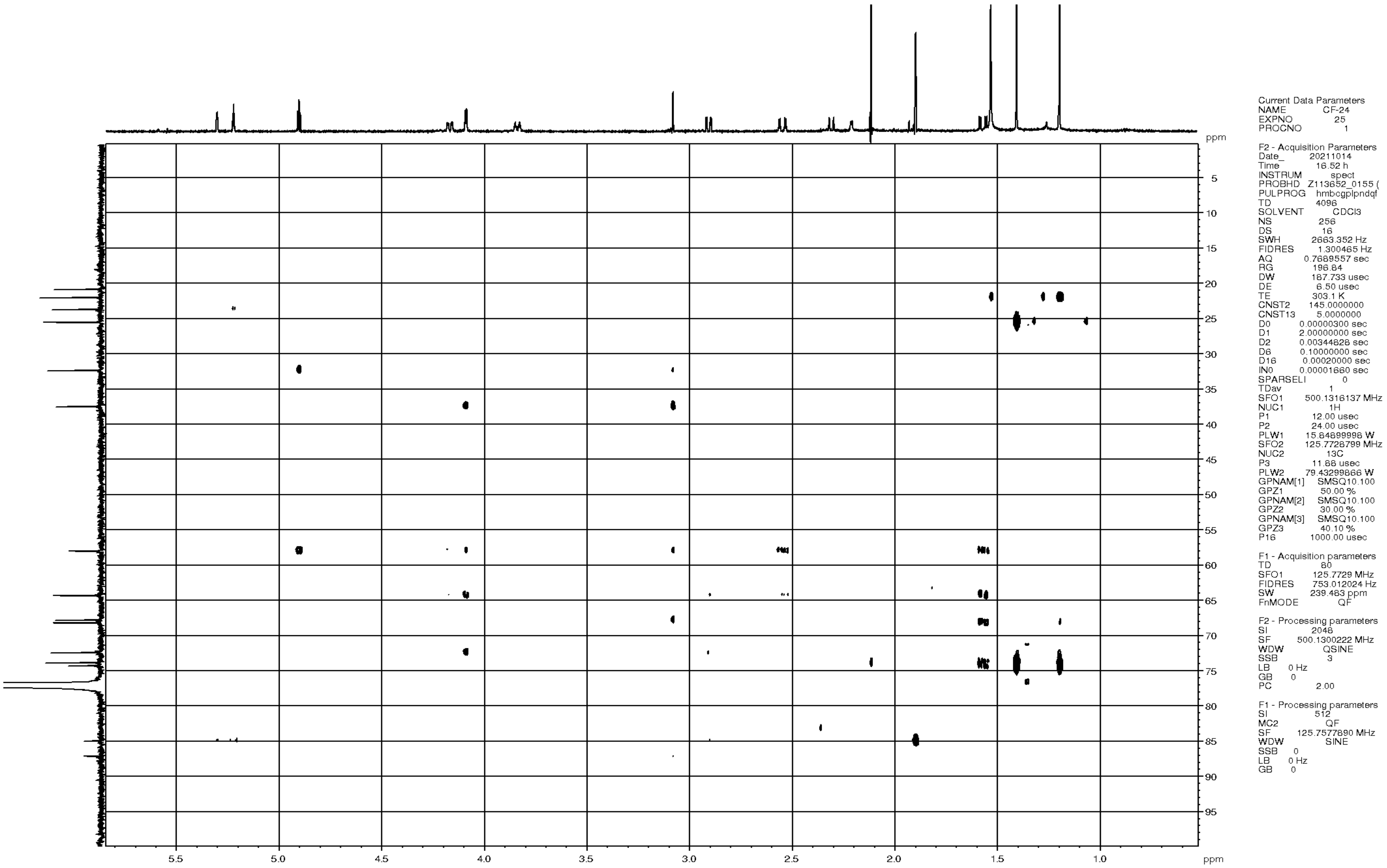

Figure S23. ROESY spectrum of oxyrapentin M (6)

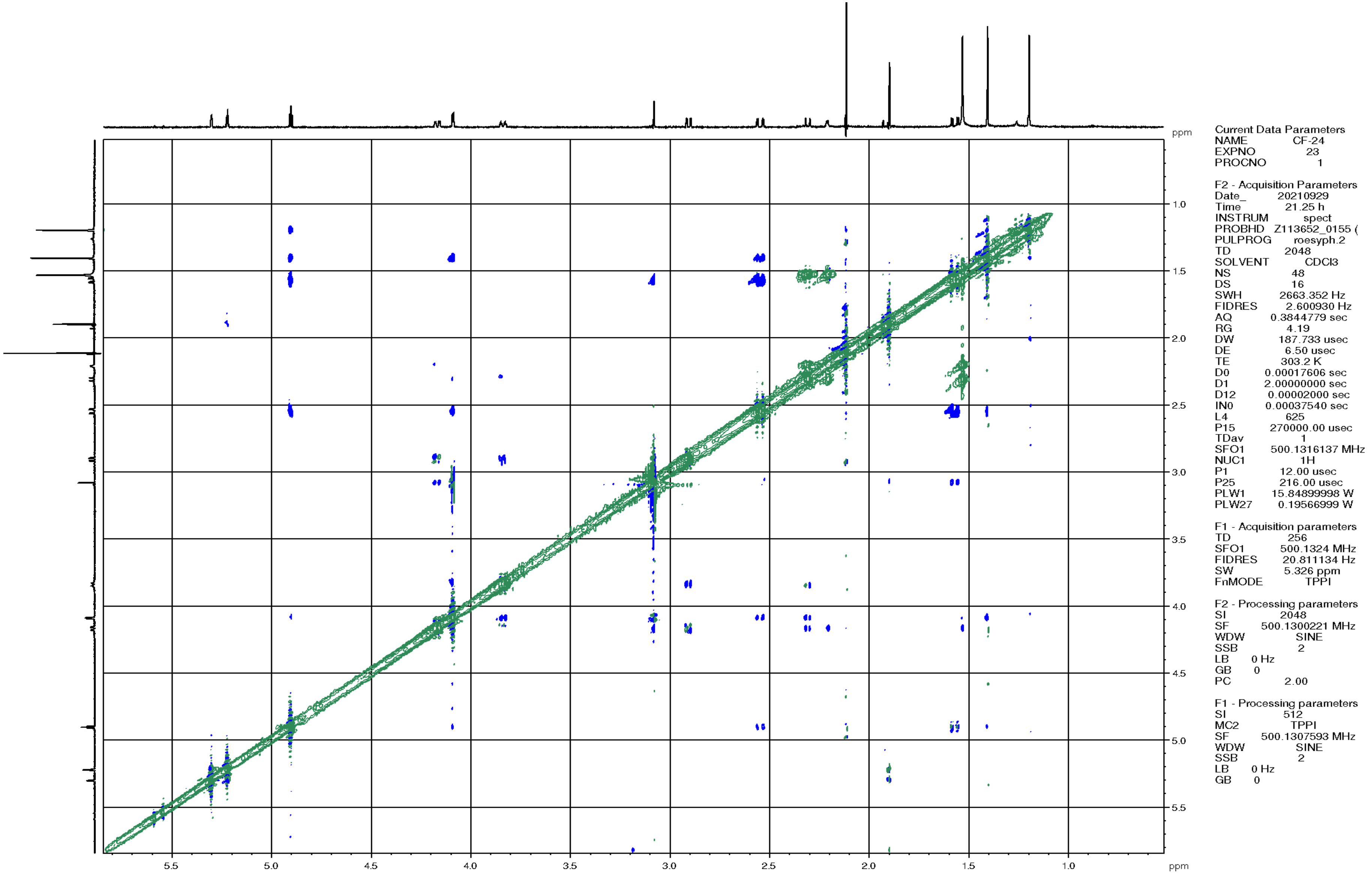

Figure S24. ^{1}**H NMR spectrum of oxyrapentin B (7)**

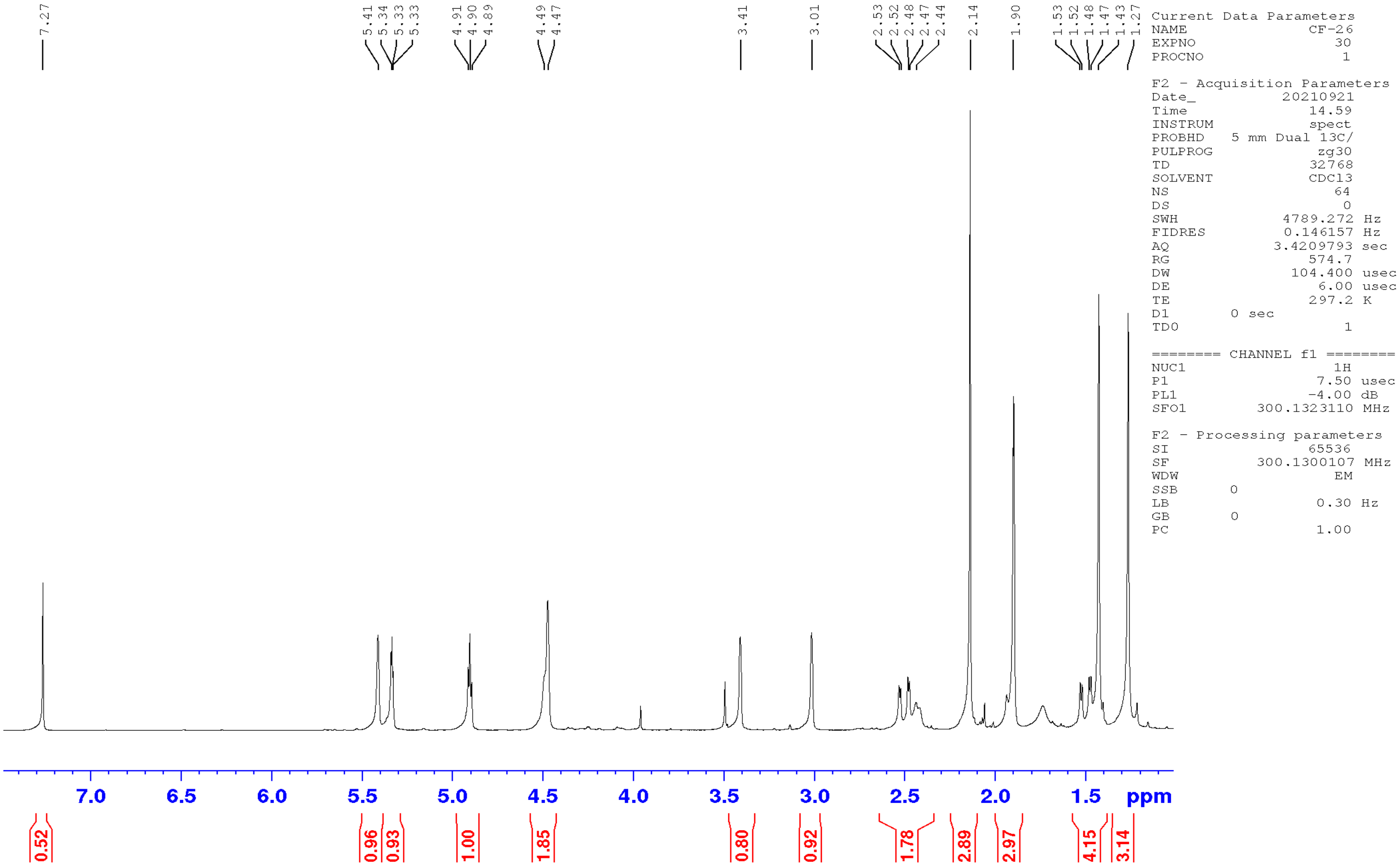

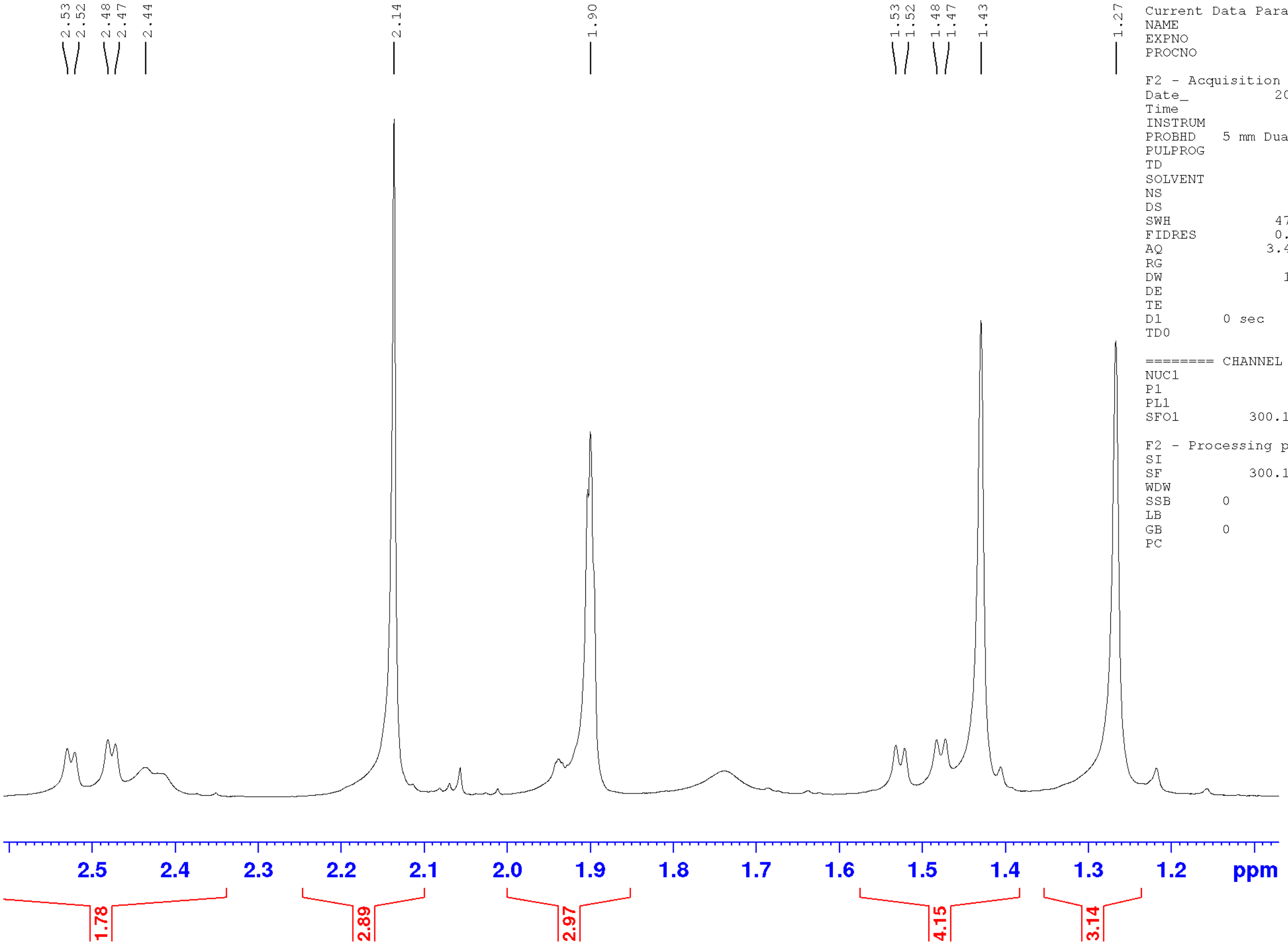


```
Current Data Parameters
NAME                CF-26
EXPNO                  30
PROCNO                  1

F2 - Acquisition Parameters
Date_            20210921
Time                14.59
INSTRUM             spect
PROBHD    5 mm Dual 13C/
PULPROG              zg30
TD                  32768
SOLVENT             CDCl3
NS                     64
DS                      0
SWH              4789.272 Hz
FIDRES           0.146157 Hz
AQ              3.4209793 sec
RG                  574.7
DW                104.400 usec
DE                   6.00 usec
TE                  297.2 K
D1        0 sec
TD0                     1

======== CHANNEL f1 ========
NUC1                   1H
P1                   7.50 usec
PL1                 -4.00 dB
SFO1          300.1323110 MHz

F2 - Processing parameters
SI                  65536
SF            300.1300107 MHz
WDW                    EM
SSB       0
LB                   0.30 Hz
GB        0
PC                   1.00
```

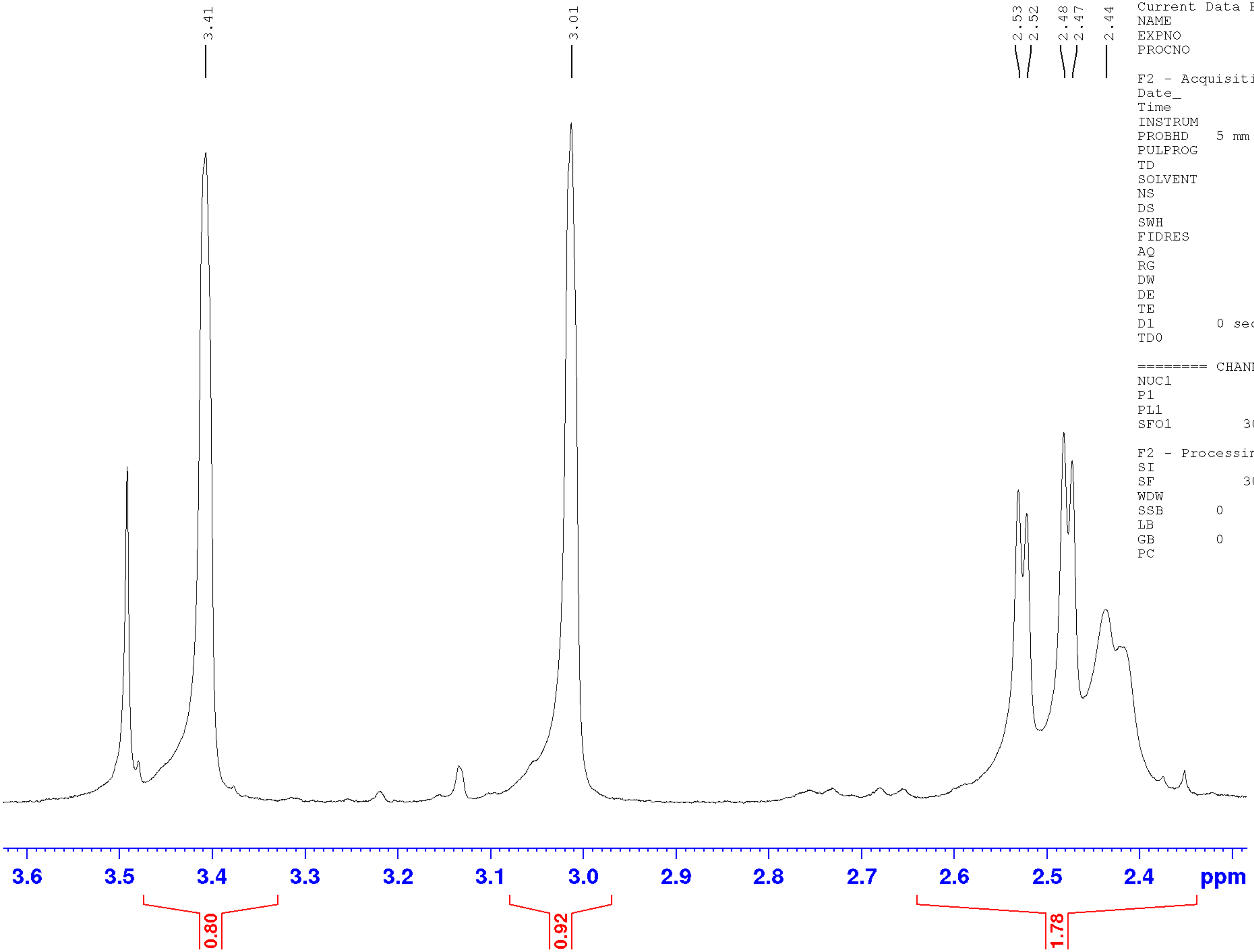

Current Data Parameters
NAME CF-26
EXPNO 30
PROCNO 1

F2 - Acquisition Parameters
Date_ 20210921
Time 14.59
INSTRUM spect
PROBHD 5 mm Dual 13C/
PULPROG zg30
TD 32768
SOLVENT CDCl3
NS 64
DS 0
SWH 4789.272 Hz
FIDRES 0.146157 Hz
AQ 3.4209793 sec
RG 574.7
DW 104.400 usec
DE 6.00 usec
TE 297.2 K
D1 0 sec
TD0 1

======== CHANNEL f1 ========
NUC1 1H
P1 7.50 usec
PL1 -4.00 dB
SFO1 300.1323110 MHz

F2 - Processing parameters
SI 65536
SF 300.1300107 MHz
WDW EM
SSB 0
LB 0.30 Hz
GB 0
PC 1.00

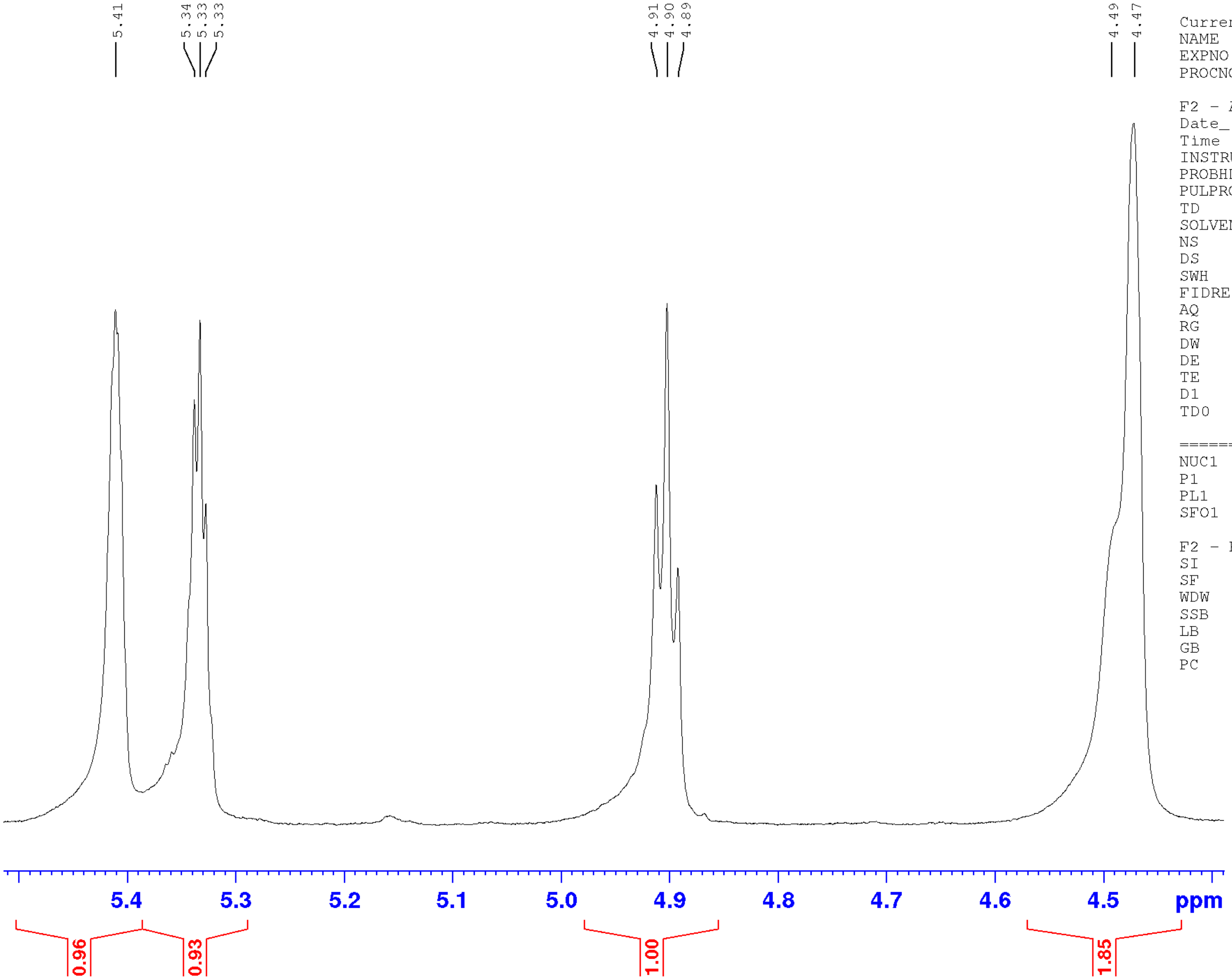

Current Data Parameters
NAME CF-26
EXPNO 30
PROCNO 1

F2 - Acquisition Parameters
Date_ 20210921
Time 14.59
INSTRUM spect
PROBHD 5 mm Dual 13C/
PULPROG zg30
TD 32768
SOLVENT CDCl3
NS 64
DS 0
SWH 4789.272 Hz
FIDRES 0.146157 Hz
AQ 3.4209793 sec
RG 574.7
DW 104.400 usec
DE 6.00 usec
TE 297.2 K
D1 0 sec
TD0 1

======== CHANNEL f1 ========
NUC1 1H
P1 7.50 usec
PL1 -4.00 dB
SFO1 300.1323110 MHz

F2 - Processing parameters
SI 65536
SF 300.1300107 MHz
WDW EM
SSB 0
LB 0.30 Hz
GB 0
PC 1.00

Figure S25. ¹H NMR spectrum of cinereain (8)

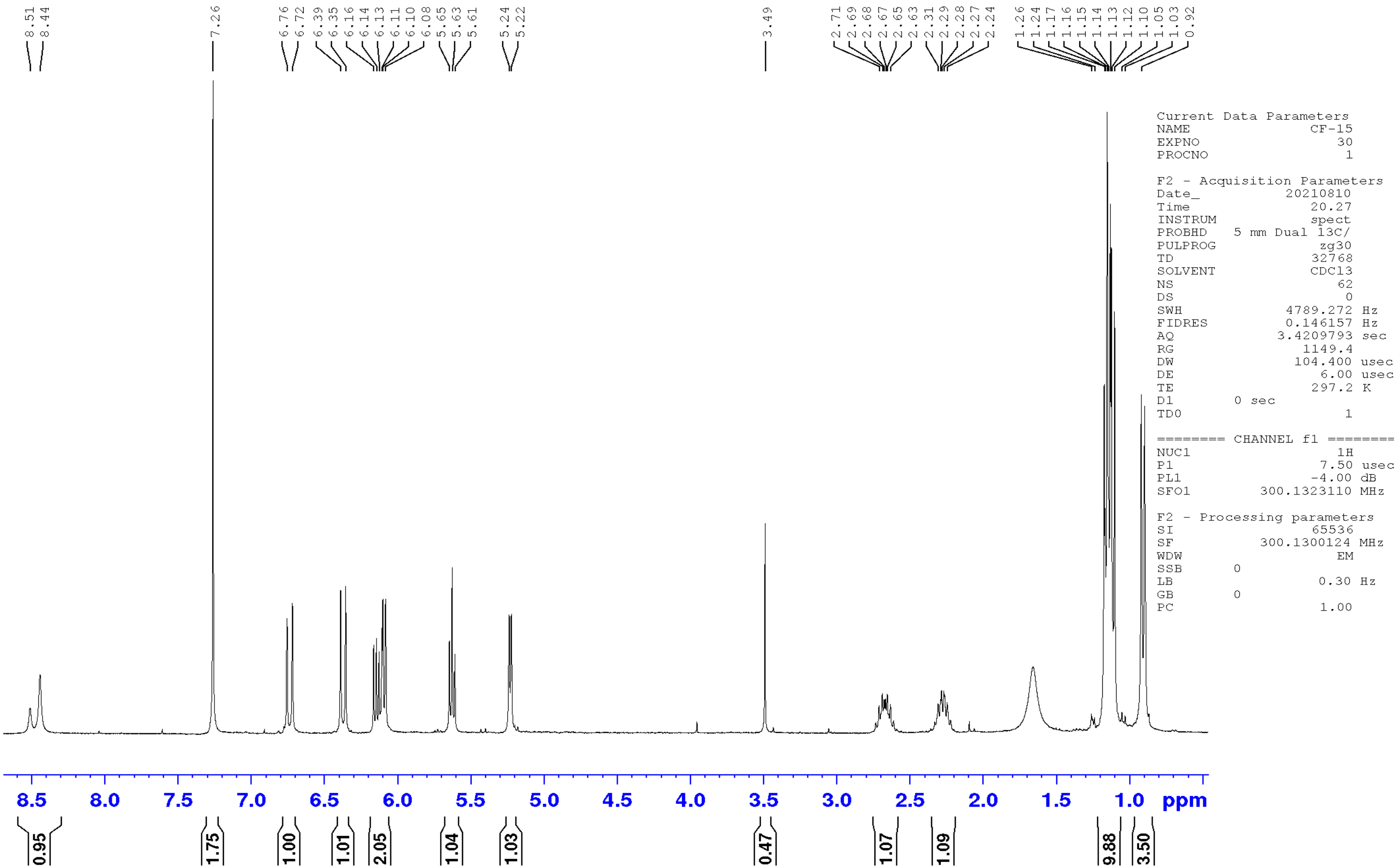

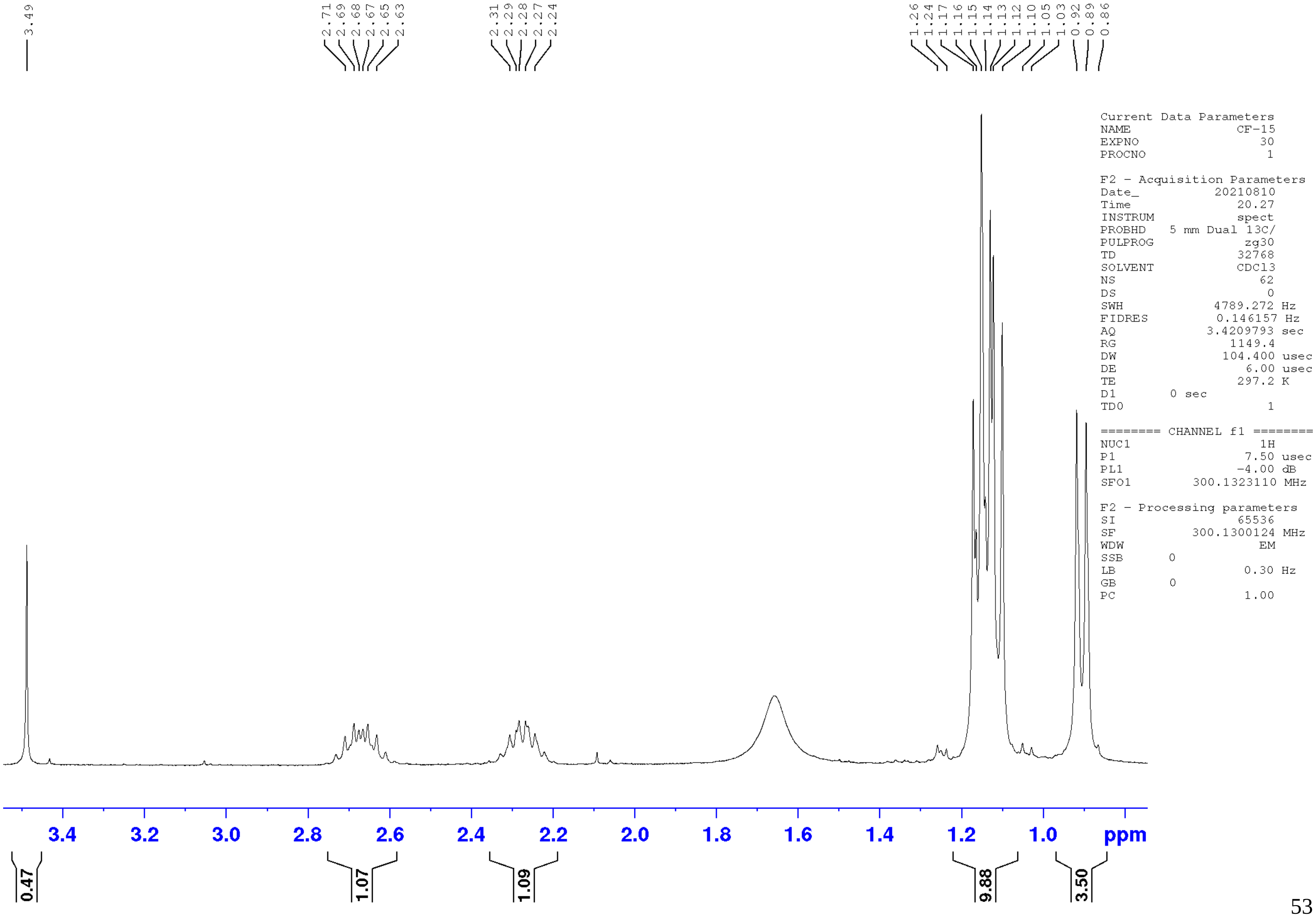
3.49
2.71 2.69 2.68 2.67 2.65 2.63
2.31 2.29 2.28 2.27 2.24
1.26 1.24 1.17 1.16 1.15 1.14 1.13 1.12 1.10 1.05 1.03 0.92 0.89 0.86
Current Data Parameters
NAME CF-15
EXPNO 30
PROCNO 1
F2 - Acquisition Parameters
Date_ 20210810
Time 20.27
INSTRUM spect
PROBHD 5 mm Dual 13C/
PULPROG zg30
TD 32768
SOLVENT CDCl3
NS 62
DS 0
SWH 4789.272 Hz
FIDRES 0.146157 Hz
AQ 3.4209793 sec
RG 1149.4
DW 104.400 usec
DE 6.00 usec
TE 297.2 K
D1 0 sec
TD0 1
======== CHANNEL f1 ========
NUC1 1H
P1 7.50 usec
PL1 -4.00 dB
SFO1 300.1323110 MHz
F2 - Processing parameters
SI 65536
SF 300.1300124 MHz
WDW EM
SSB 0
LB 0.30 Hz
GB 0
PC 1.00
3.4 3.2 3.0 2.8 2.6 2.4 2.2 2.0 1.8 1.6 1.4 1.2 1.0 ppm
0.47
1.07
1.09
9.88
3.50

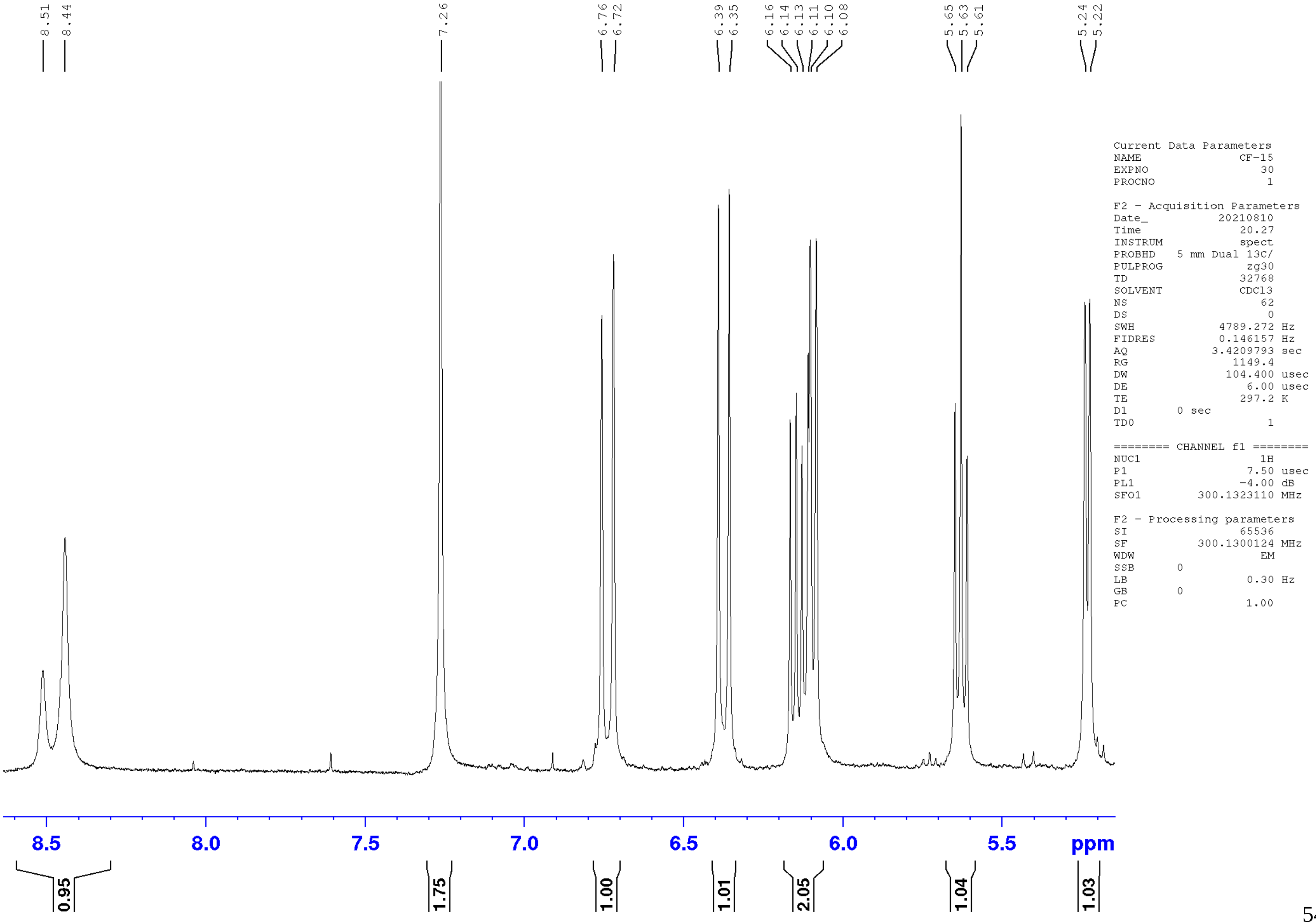
8.51
8.44
7.26
6.76
6.72
6.39
6.35
6.16
6.14
6.13
6.11
6.10
6.08
5.65
5.63
5.61
5.24
5.22
Current Data Parameters
NAME CF-15
EXPNO 30
PROCNO 1
F2 - Acquisition Parameters
Date_ 20210810
Time 20.27
INSTRUM spect
PROBHD 5 mm Dual 13C/
PULPROG zg30
TD 32768
SOLVENT CDCl3
NS 62
DS 0
SWH 4789.272 Hz
FIDRES 0.146157 Hz
AQ 3.4209793 sec
RG 1149.4
DW 104.400 usec
DE 6.00 usec
TE 297.2 K
D1 0 sec
TD0 1
======== CHANNEL f1 ========
NUC1 1H
P1 7.50 usec
PL1 -4.00 dB
SFO1 300.1323110 MHz
F2 - Processing parameters
SI 65536
SF 300.1300124 MHz
WDW EM
SSB 0
LB 0.30 Hz
GB 0
PC 1.00
8.5
8.0
7.5
7.0
6.5
6.0
5.5
ppm
0.95
1.75
1.00
1.01
2.05
1.04
1.03

Figure S26. ¹H NMR spectrum of carneamide A (9)

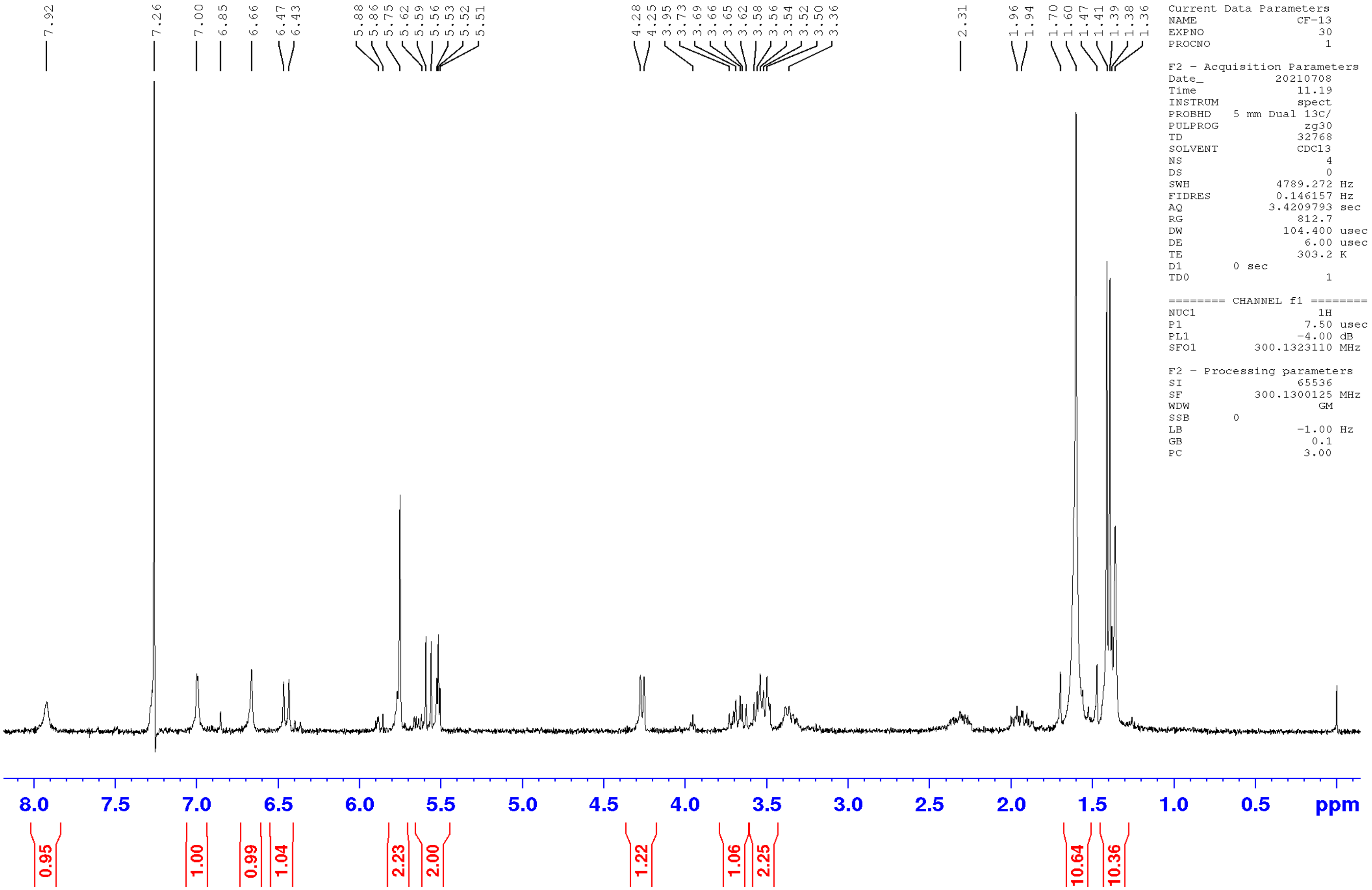

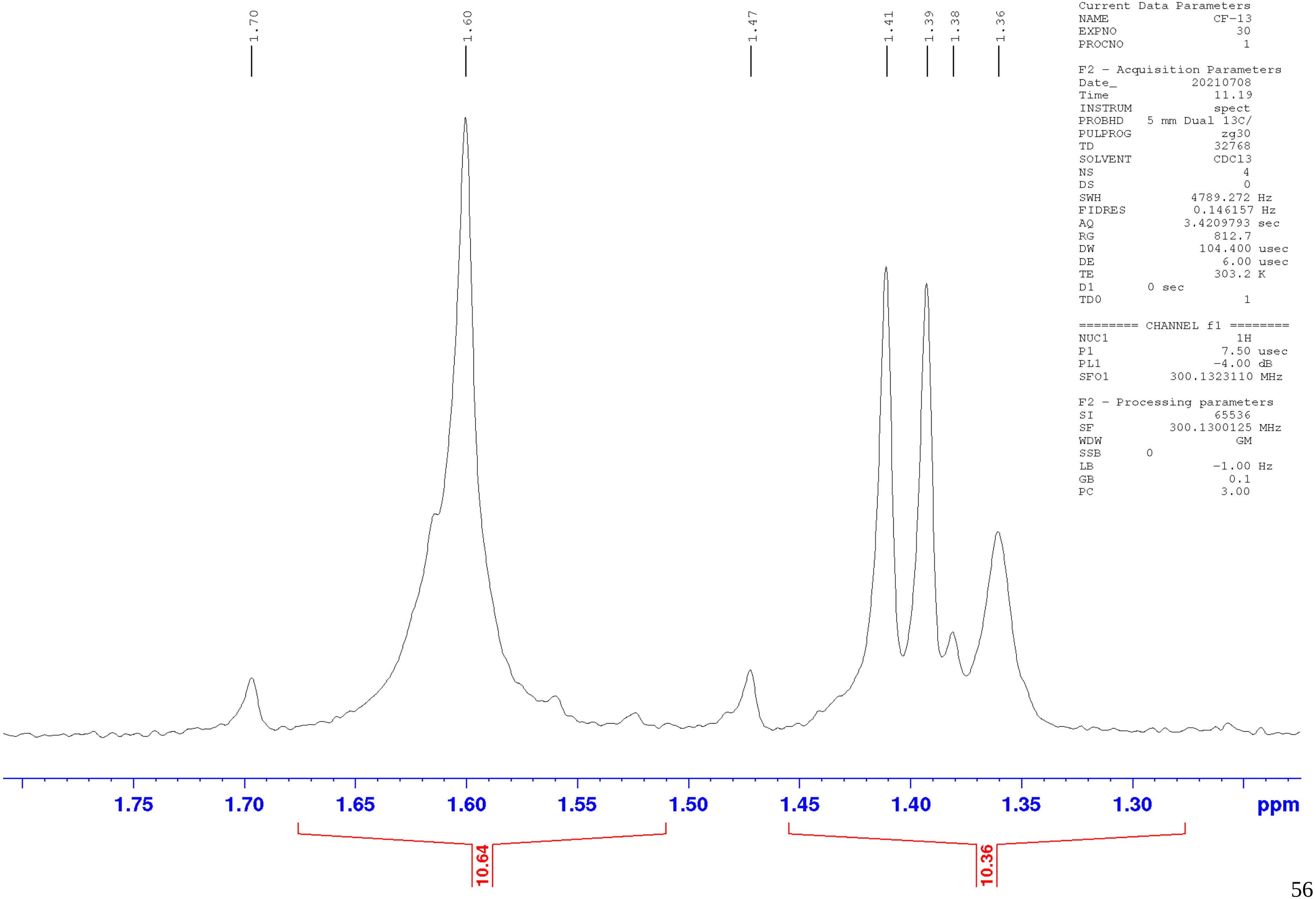
1.70
1.60
1.47
1.41
1.39
1.38
1.36
Current Data Parameters
NAME CF-13
EXPNO 30
PROCNO 1
F2 - Acquisition Parameters
Date_ 20210708
Time 11.19
INSTRUM spect
PROBHD 5 mm Dual 13C/
PULPROG zg30
TD 32768
SOLVENT CDCl3
NS 4
DS 0
SWH 4789.272 Hz
FIDRES 0.146157 Hz
AQ 3.4209793 sec
RG 812.7
DW 104.400 usec
DE 6.00 usec
TE 303.2 K
D1 0 sec
TD0 1
======== CHANNEL f1 ========
NUC1 1H
P1 7.50 usec
PL1 -4.00 dB
SFO1 300.1323110 MHz
F2 - Processing parameters
SI 65536
SF 300.1300125 MHz
WDW GM
SSB 0
LB -1.00 Hz
GB 0.1
PC 3.00
1.75
1.70
1.65
1.60
1.55
1.50
1.45
1.40
1.35
1.30
ppm
10.64
10.36

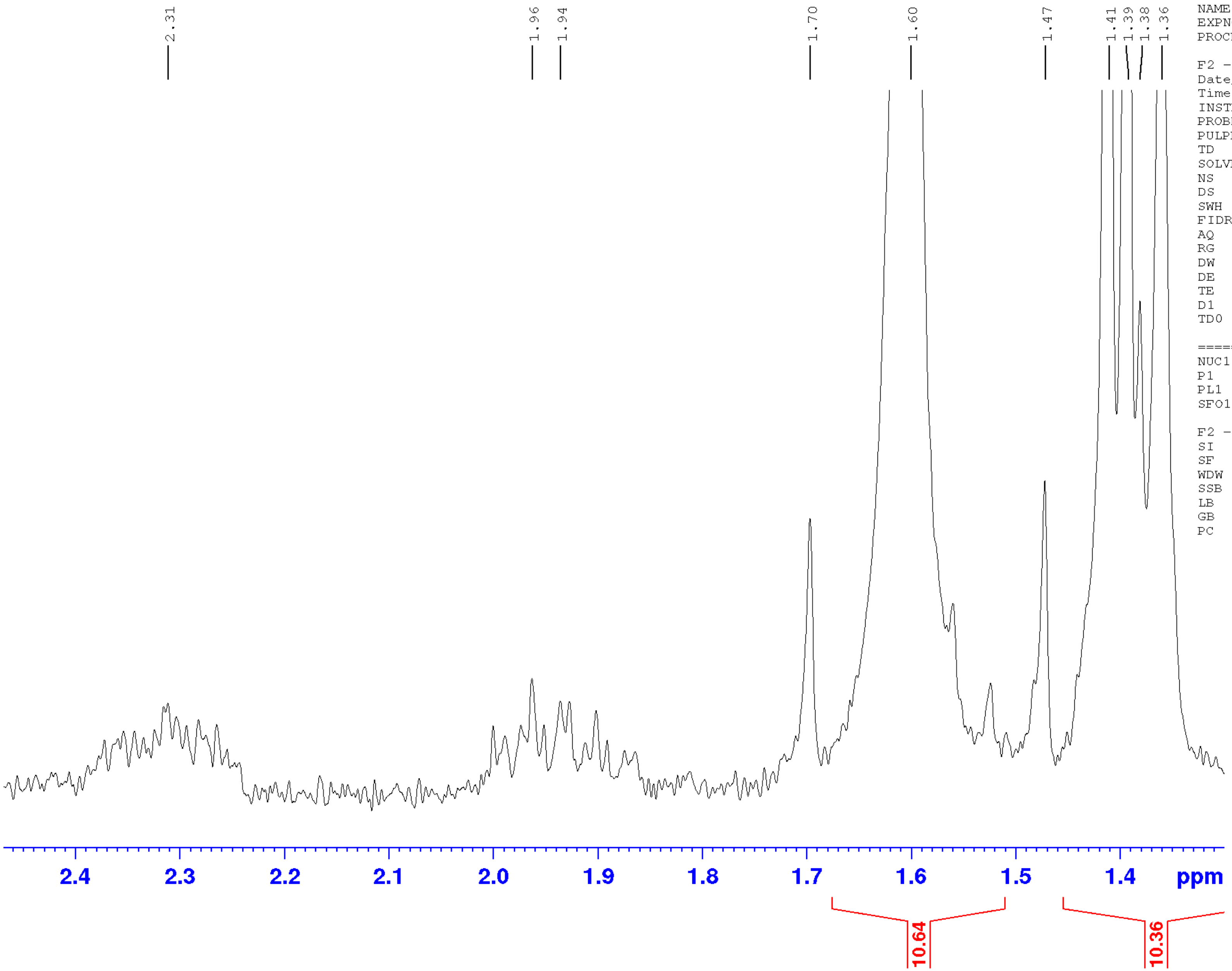

Current Data Parameters
NAME CF-13
EXPNO 30
PROCNO 1

F2 - Acquisition Parameters
Date_ 20210708
Time 11.19
INSTRUM spect
PROBHD 5 mm Dual 13C/
PULPROG zg30
TD 32768
SOLVENT CDCl3
NS 4
DS 0
SWH 4789.272 Hz
FIDRES 0.146157 Hz
AQ 3.4209793 sec
RG 812.7
DW 104.400 usec
DE 6.00 usec
TE 303.2 K
D1 0 sec
TD0 1

======== CHANNEL f1 ========
NUC1 1H
P1 7.50 usec
PL1 -4.00 dB
SFO1 300.1323110 MHz

F2 - Processing parameters
SI 65536
SF 300.1300125 MHz
WDW GM
SSB 0
LB -1.00 Hz
GB 0.1
PC 3.00

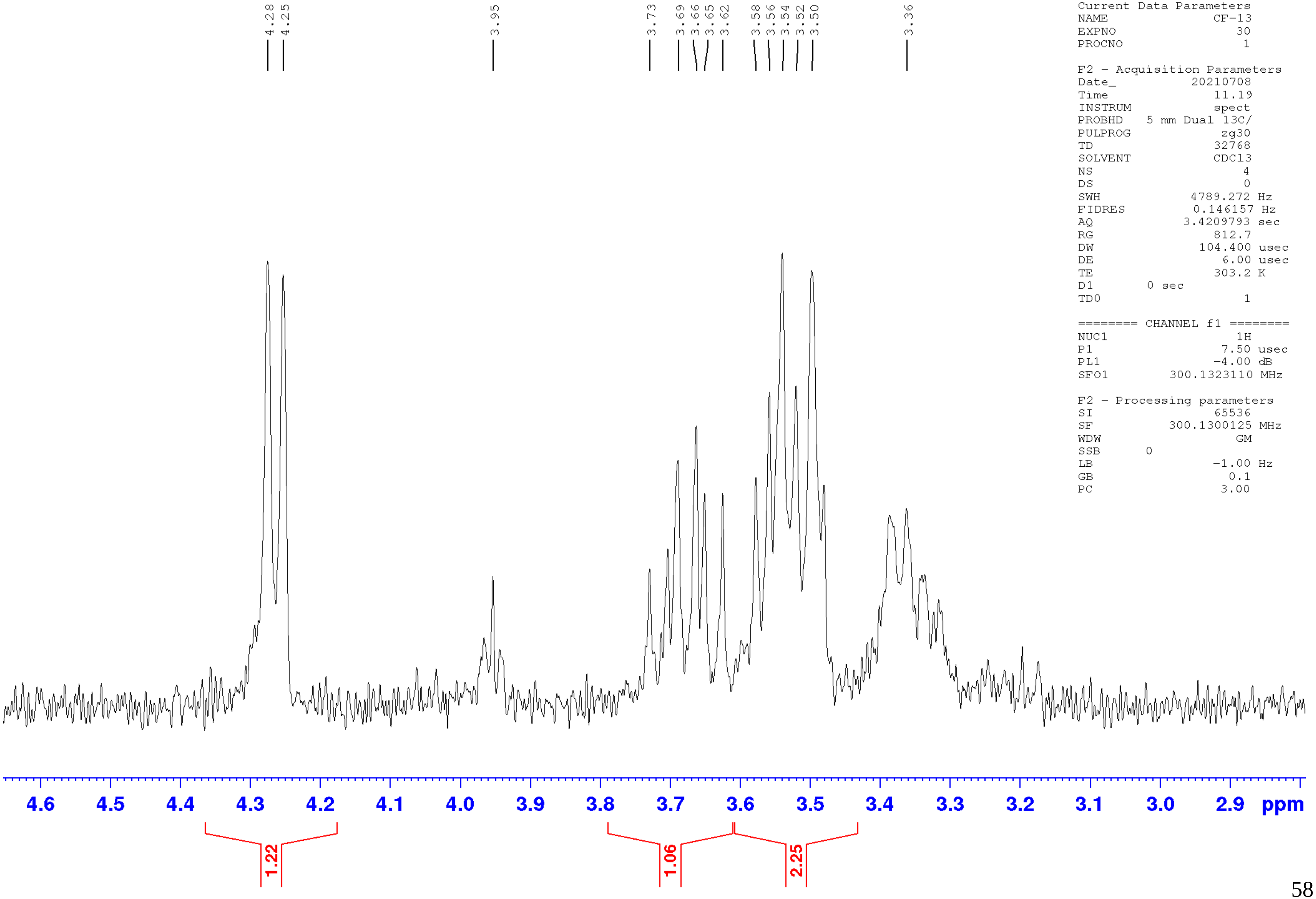
4.28
4.25
3.95
3.73
3.69
3.66
3.65
3.62
3.58
3.56
3.54
3.52
3.50
3.36
Current Data Parameters
NAME CF-13
EXPNO 30
PROCNO 1
F2 - Acquisition Parameters
Date_ 20210708
Time 11.19
INSTRUM spect
PROBHD 5 mm Dual 13C/
PULPROG zg30
TD 32768
SOLVENT CDCl3
NS 4
DS 0
SWH 4789.272 Hz
FIDRES 0.146157 Hz
AQ 3.4209793 sec
RG 812.7
DW 104.400 usec
DE 6.00 usec
TE 303.2 K
D1 0 sec
TD0 1
======== CHANNEL f1 ========
NUC1 1H
P1 7.50 usec
PL1 -4.00 dB
SFO1 300.1323110 MHz
F2 - Processing parameters
SI 65536
SF 300.1300125 MHz
WDW GM
SSB 0
LB -1.00 Hz
GB 0.1
PC 3.00
4.6
4.5
4.4
4.3
4.2
4.1
4.0
3.9
3.8
3.7
3.6
3.5
3.4
3.3
3.2
3.1
3.0
2.9
ppm
1.22
1.06
2.25

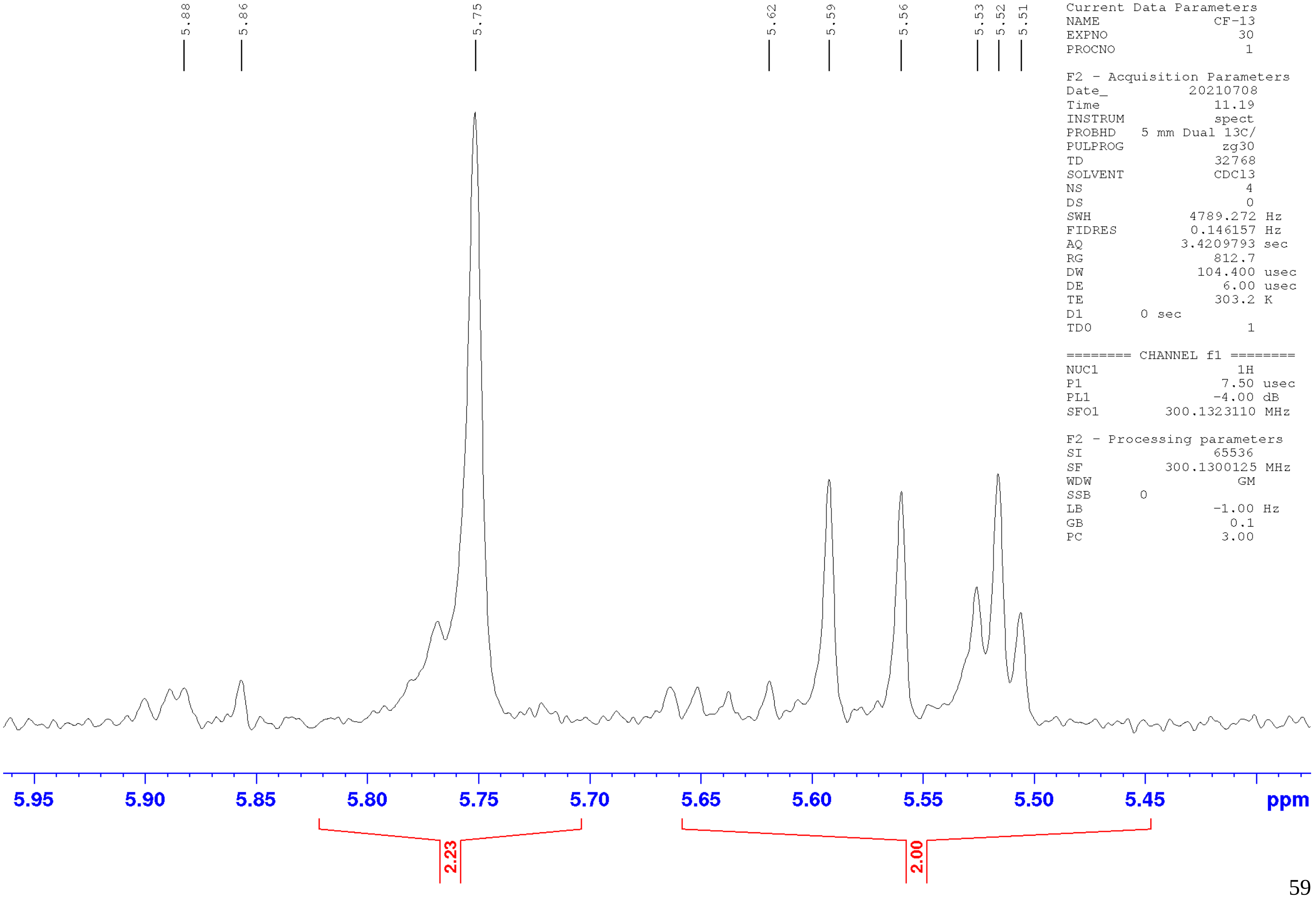

Current Data Parameters
NAME CF-13
EXPNO 30
PROCNO 1

F2 - Acquisition Parameters
Date_ 20210708
Time 11.19
INSTRUM spect
PROBHD 5 mm Dual 13C/
PULPROG zg30
TD 32768
SOLVENT CDCl3
NS 4
DS 0
SWH 4789.272 Hz
FIDRES 0.146157 Hz
AQ 3.4209793 sec
RG 812.7
DW 104.400 usec
DE 6.00 usec
TE 303.2 K
D1 0 sec
TD0 1

======== CHANNEL f1 ========
NUC1 1H
P1 7.50 usec
PL1 -4.00 dB
SFO1 300.1323110 MHz

F2 - Processing parameters
SI 65536
SF 300.1300125 MHz
WDW GM
SSB 0
LB -1.00 Hz
GB 0.1
PC 3.00

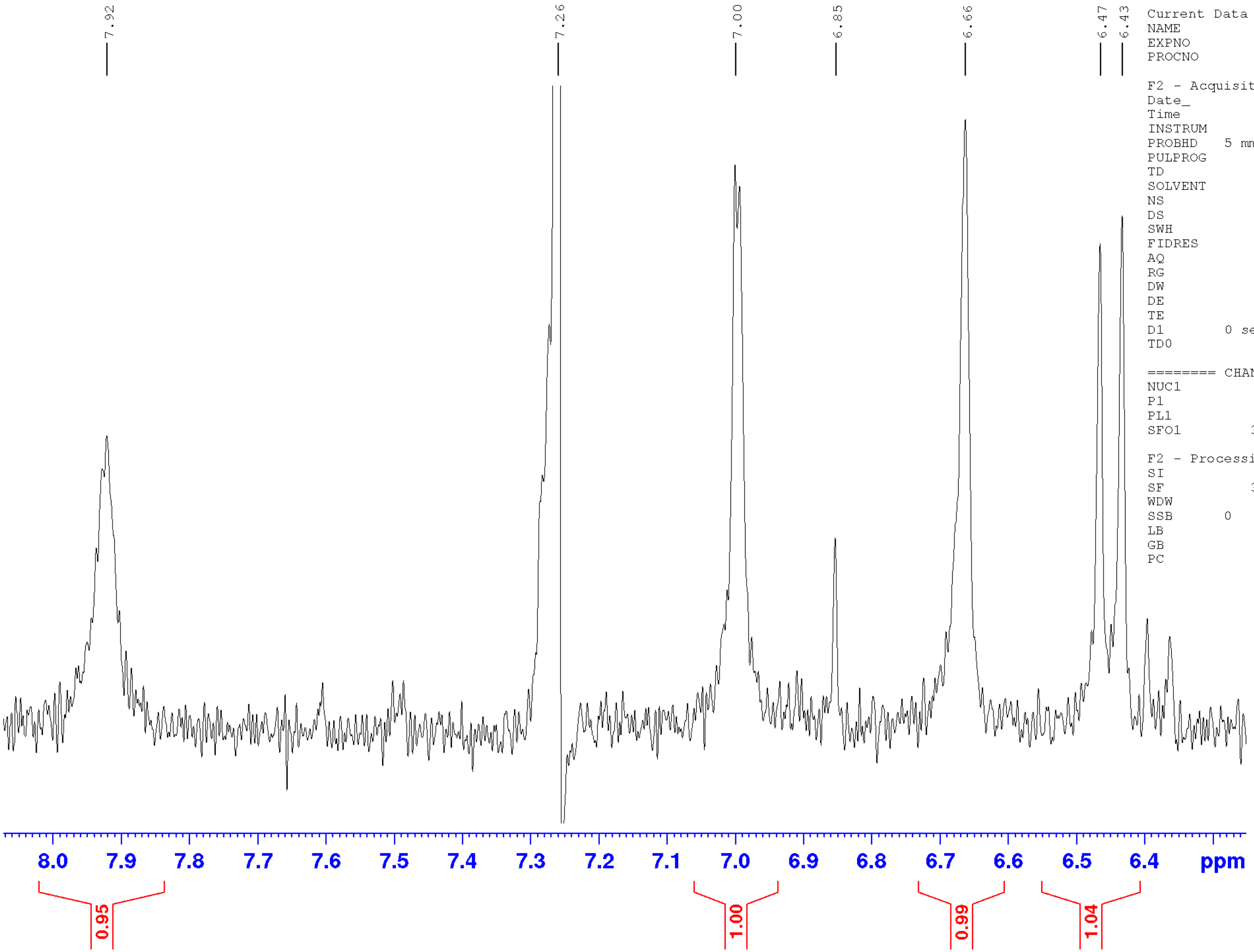

Current Data Parameters
NAME CF-13
EXPNO 30
PROCNO 1

F2 - Acquisition Parameters
Date_ 20210708
Time 11.19
INSTRUM spect
PROBHD 5 mm Dual 13C/
PULPROG zg30
TD 32768
SOLVENT CDCl3
NS 4
DS 0
SWH 4789.272 Hz
FIDRES 0.146157 Hz
AQ 3.4209793 sec
RG 812.7
DW 104.400 usec
DE 6.00 usec
TE 303.2 K
D1 0 sec
TD0 1

======== CHANNEL f1 ========
NUC1 1H
P1 7.50 usec
PL1 -4.00 dB
SFO1 300.1323110 MHz

F2 - Processing parameters
SI 65536
SF 300.1300125 MHz
WDW GM
SSB 0
LB -1.00 Hz
GB 0.1
PC 3.00

Figure S27. ¹H NMR spectrum of aspergillicine A (10)

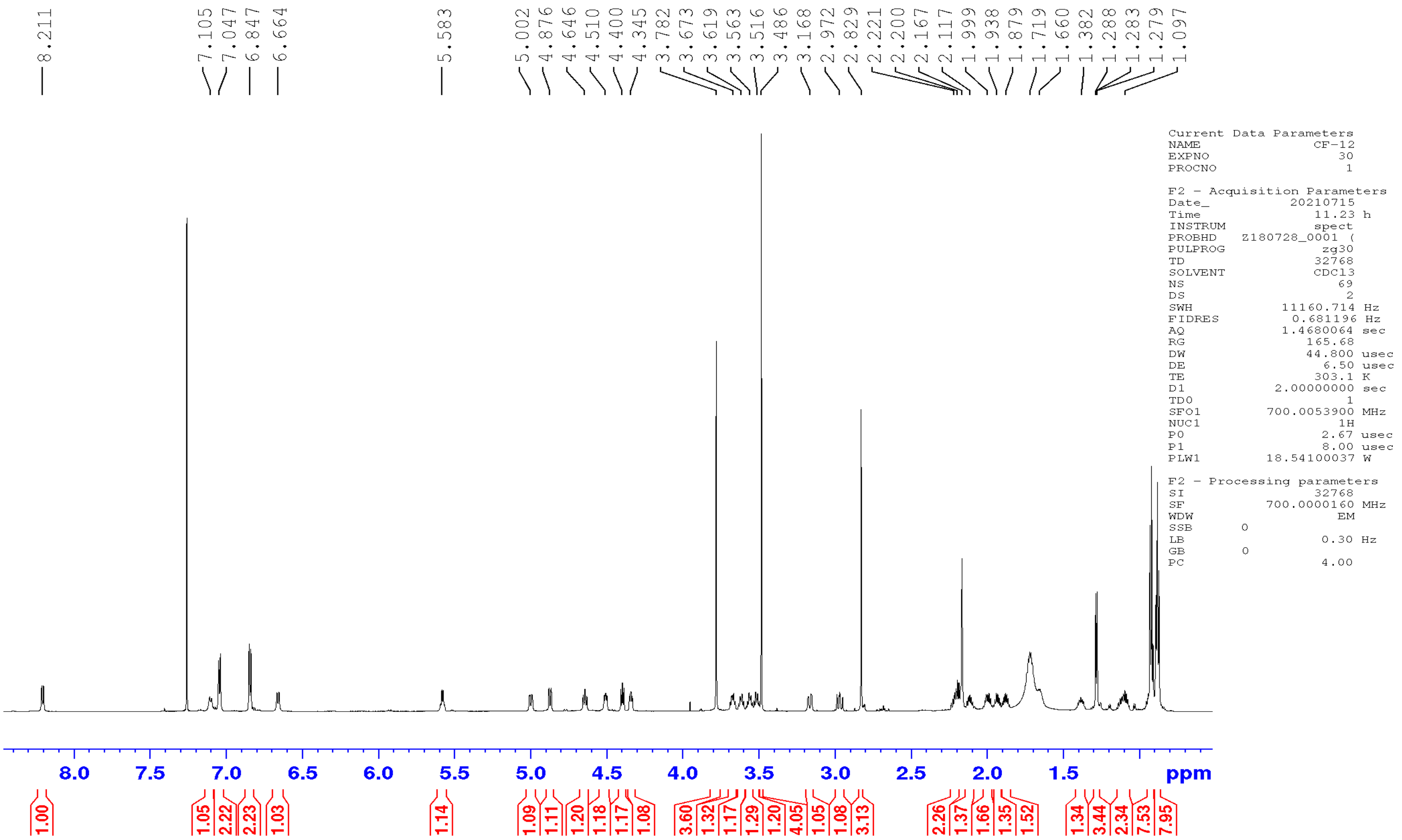

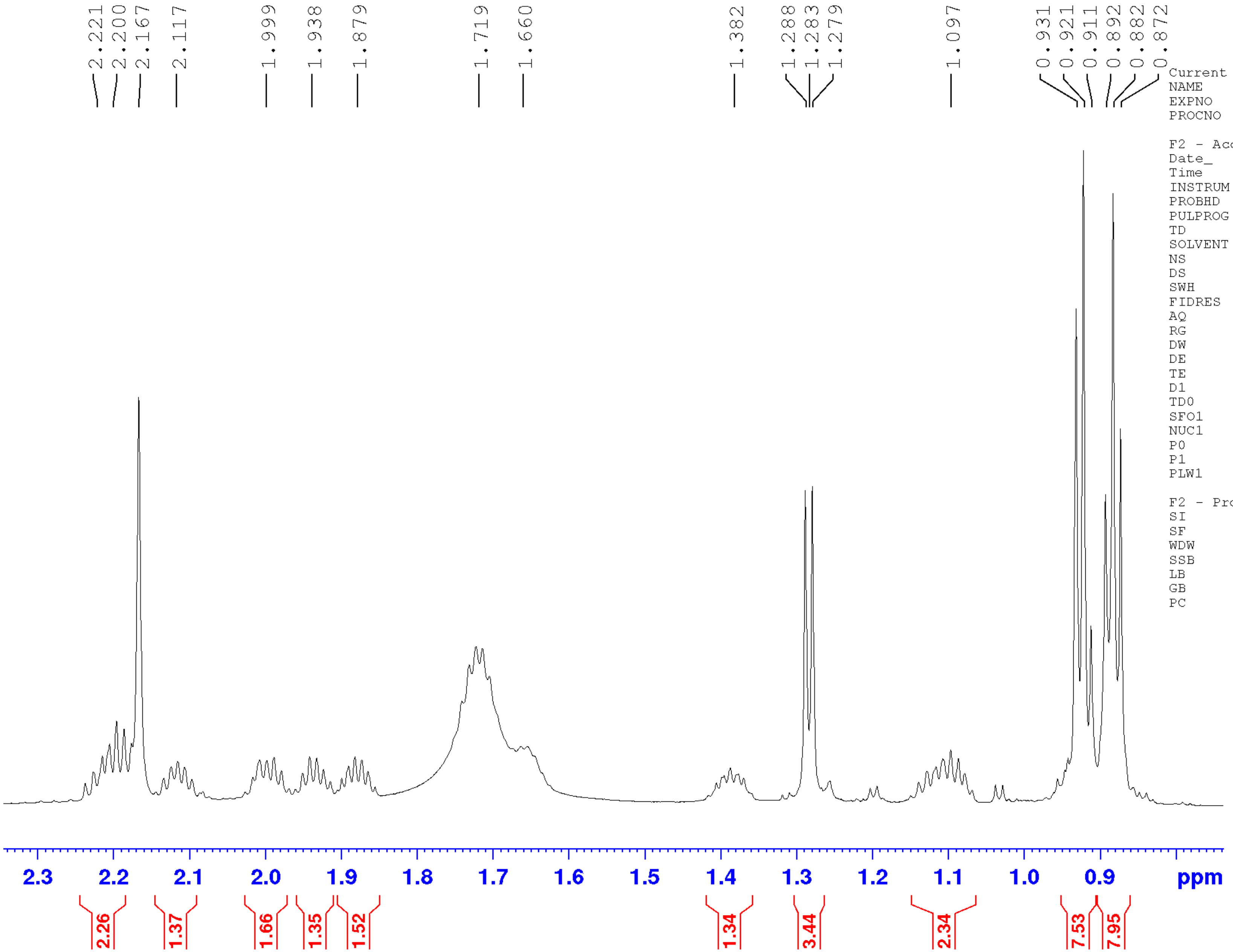

Current Data Parameters
NAME CF-12
EXPNO 30
PROCNO 1

F2 - Acquisition Parameters
Date_ 20210715
Time 11.23 h
INSTRUM spect
PROBHD Z180728_0001 (
PULPROG zg30
TD 32768
SOLVENT CDCl3
NS 69
DS 2
SWH 11160.714 Hz
FIDRES 0.681196 Hz
AQ 1.4680064 sec
RG 165.68
DW 44.800 usec
DE 6.50 usec
TE 303.1 K
D1 2.00000000 sec
TD0 1
SFO1 700.0053900 MHz
NUC1 1H
P0 2.67 usec
P1 8.00 usec
PLW1 18.54100037 W

F2 - Processing parameters
SI 32768
SF 700.0000160 MHz
WDW EM
SSB 0
LB 0.30 Hz
GB 0
PC 4.00

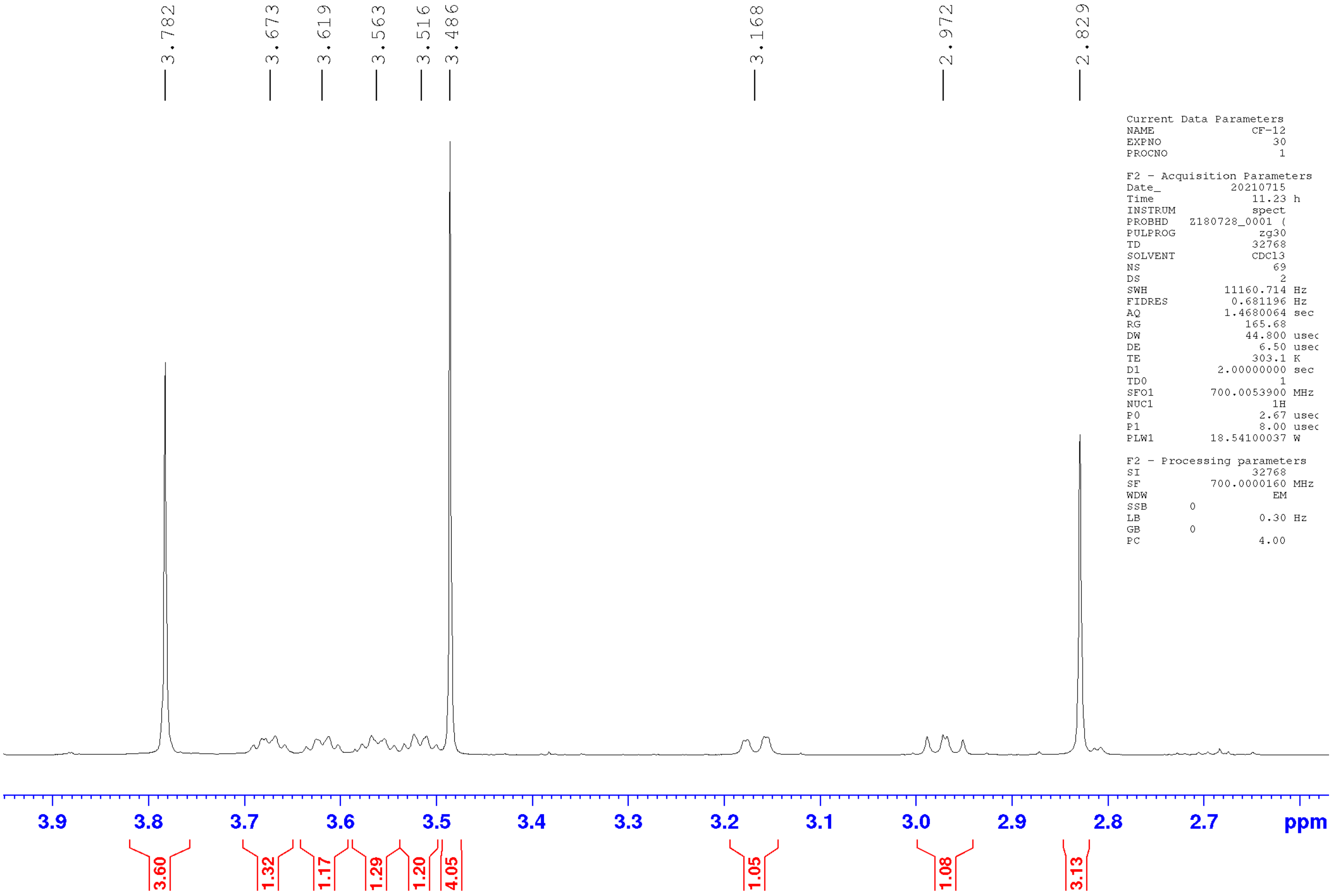
3.782
3.673
3.619
3.563
3.516
3.486
3.168
2.972
2.829
Current Data Parameters
NAME CF-12
EXPNO 30
PROCNO 1
F2 - Acquisition Parameters
Date_ 20210715
Time 11.23 h
INSTRUM spect
PROBHD Z180728_0001 (
PULPROG zg30
TD 32768
SOLVENT CDCl3
NS 69
DS 2
SWH 11160.714 Hz
FIDRES 0.681196 Hz
AQ 1.4680064 sec
RG 165.68
DW 44.800 usec
DE 6.50 usec
TE 303.1 K
D1 2.00000000 sec
TD0 1
SFO1 700.0053900 MHz
NUC1 1H
P0 2.67 usec
P1 8.00 usec
PLW1 18.54100037 W
F2 - Processing parameters
SI 32768
SF 700.0000160 MHz
WDW EM
SSB 0
LB 0.30 Hz
GB 0
PC 4.00
3.9
3.8
3.7
3.6
3.5
3.4
3.3
3.2
3.1
3.0
2.9
2.8
2.7
ppm
3.60
1.32
1.17
1.29
1.20
4.05
1.05
1.08
3.13

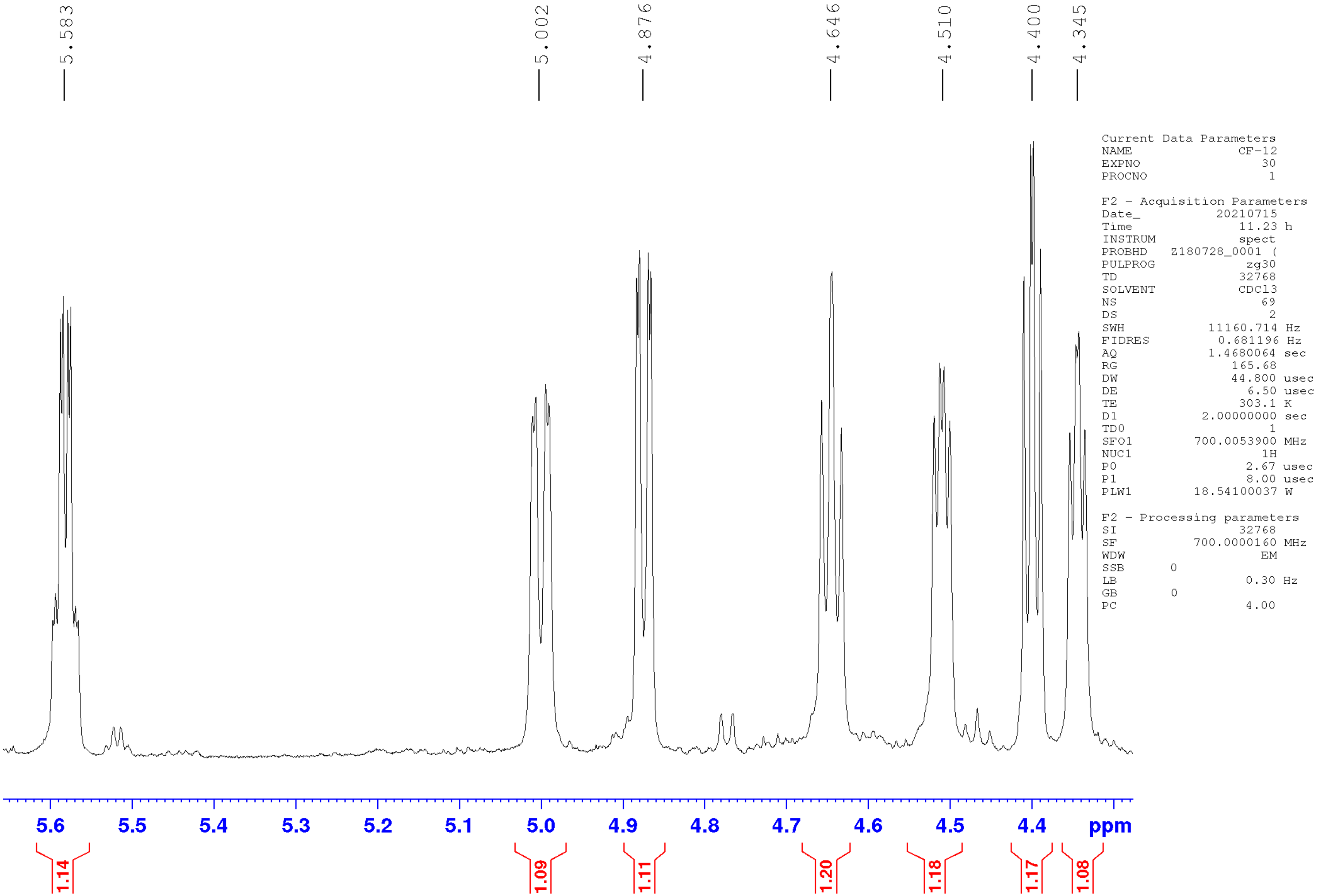
5.583
5.002
4.876
4.646
4.510
4.400
4.345
Current Data Parameters
NAME CF-12
EXPNO 30
PROCNO 1
F2 - Acquisition Parameters
Date_ 20210715
Time 11.23 h
INSTRUM spect
PROBHD Z180728_0001 (
PULPROG zg30
TD 32768
SOLVENT CDCl3
NS 69
DS 2
SWH 11160.714 Hz
FIDRES 0.681196 Hz
AQ 1.4680064 sec
RG 165.68
DW 44.800 usec
DE 6.50 usec
TE 303.1 K
D1 2.00000000 sec
TD0 1
SFO1 700.0053900 MHz
NUC1 1H
P0 2.67 usec
P1 8.00 usec
PLW1 18.54100037 W
F2 - Processing parameters
SI 32768
SF 700.0000160 MHz
WDW EM
SSB 0
LB 0.30 Hz
GB 0
PC 4.00
5.6
5.5
5.4
5.3
5.2
5.1
5.0
4.9
4.8
4.7
4.6
4.5
4.4
ppm
1.14
1.09
1.11
1.20
1.18
1.17
1.08

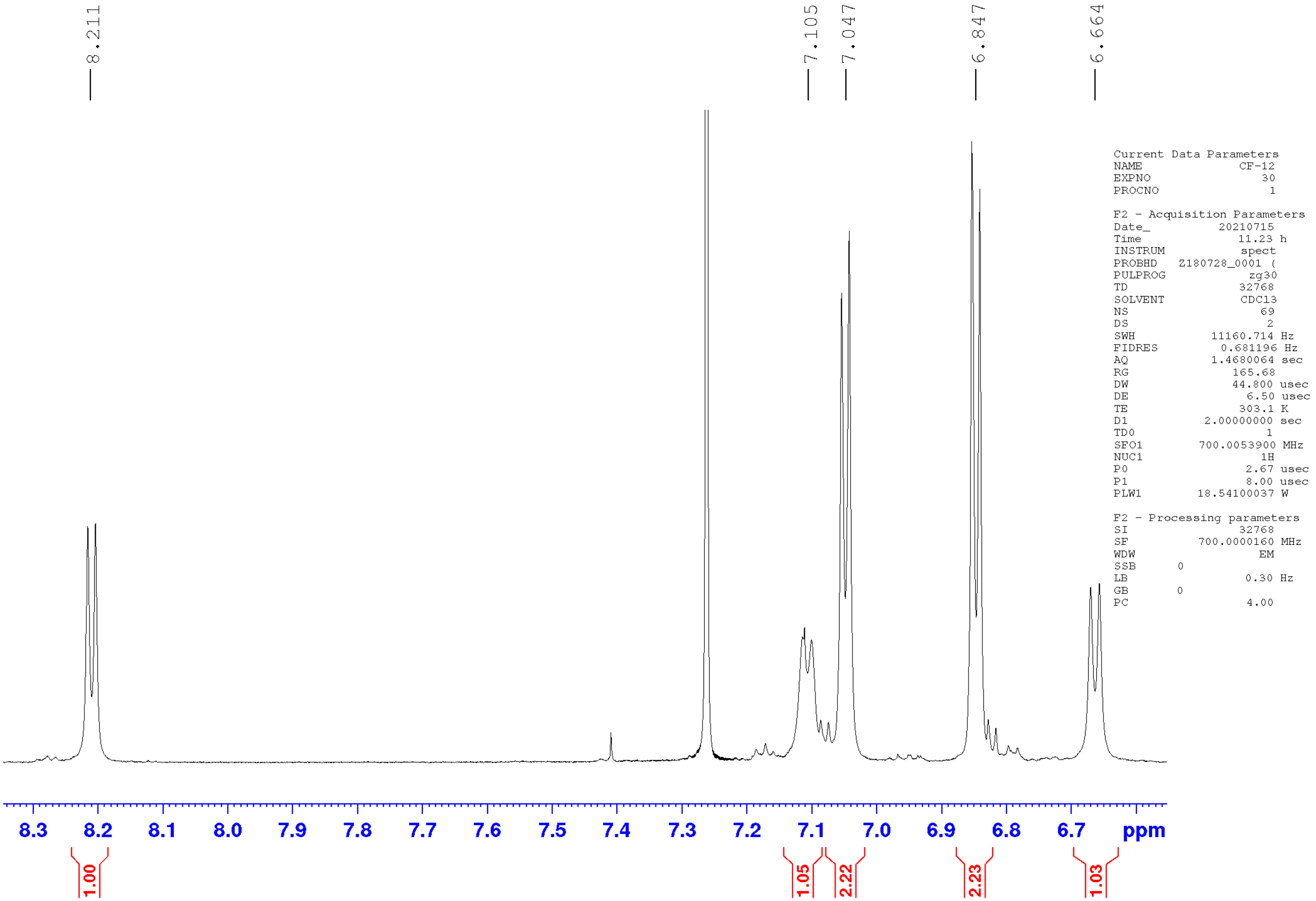
8.211
7.105
7.047
6.847
6.664
Current Data Parameters
NAME CF-12
EXPNO 30
PROCNO 1
F2 - Acquisition Parameters
Date_ 20210715
Time 11.23 h
INSTRUM spect
PROBHD Z180728_0001 (
PULPROG zg30
TD 32768
SOLVENT CDCl3
NS 69
DS 2
SWH 11160.714 Hz
FIDRES 0.681196 Hz
AQ 1.4680064 sec
RG 165.68
DW 44.800 usec
DE 6.50 usec
TE 303.1 K
D1 2.00000000 sec
TD0 1
SFO1 700.0053900 MHz
NUC1 1H
P0 2.67 usec
P1 8.00 usec
PLW1 18.54100037 W
F2 - Processing parameters
SI 32768
SF 700.0000160 MHz
WDW EM
SSB 0
LB 0.30 Hz
GB 0
PC 4.00
8.3
8.2
8.1
8.0
7.9
7.8
7.7
7.6
7.5
7.4
7.3
7.2
7.1
7.0
6.9
6.8
6.7
ppm
1.00
1.05
2.22
2.23
1.03

Figure S28. ^{1}H-^{1}H COSY spectrum of aspergillicine A (10)

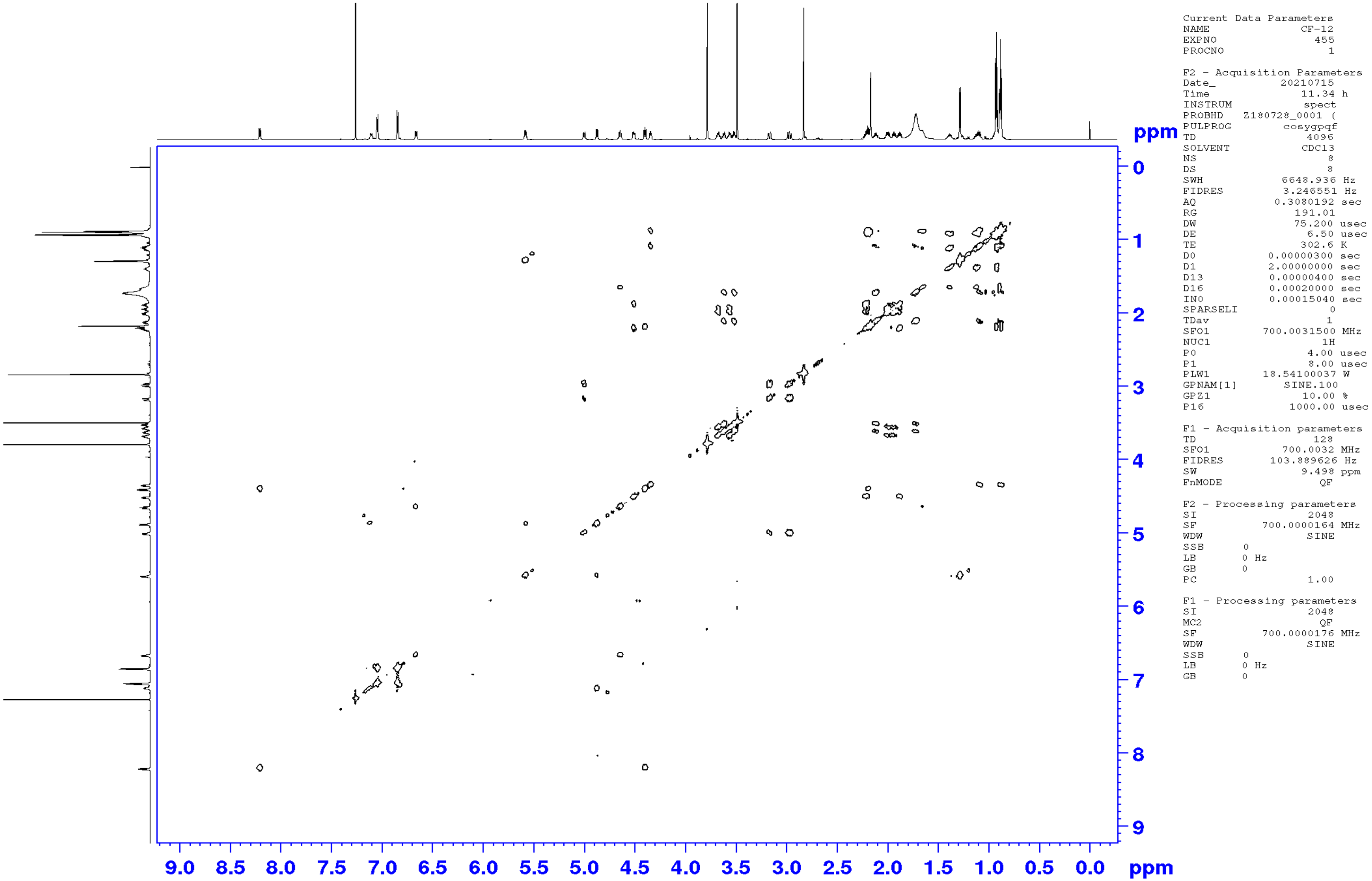

Figure S29. HSQC spectrum of aspergillicine A (10)

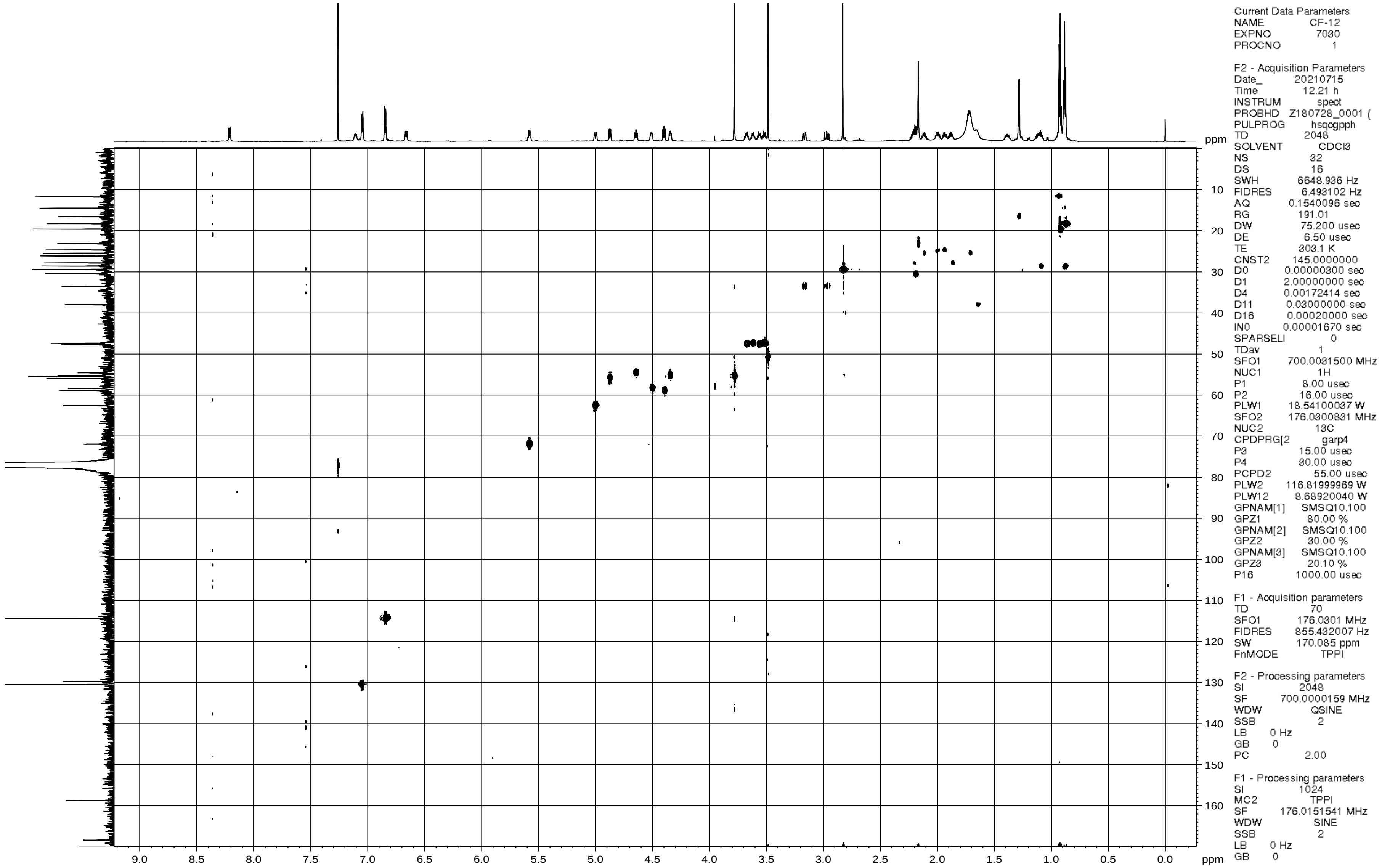

Figure S30. HMBC spectrum of aspergillicine A (10)

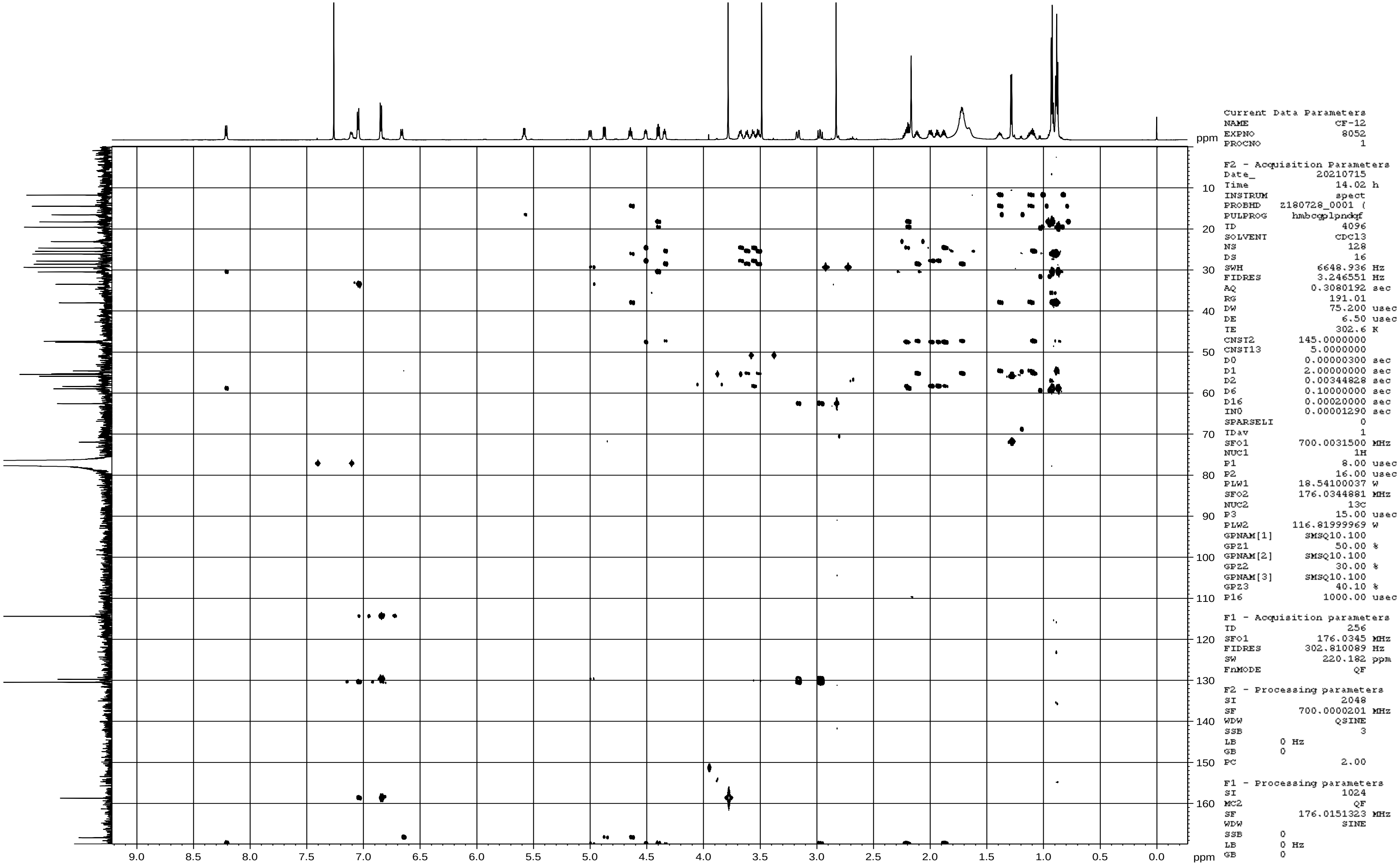

Figure S31. ^{1}H NMR spectrum of isaridine E (11)

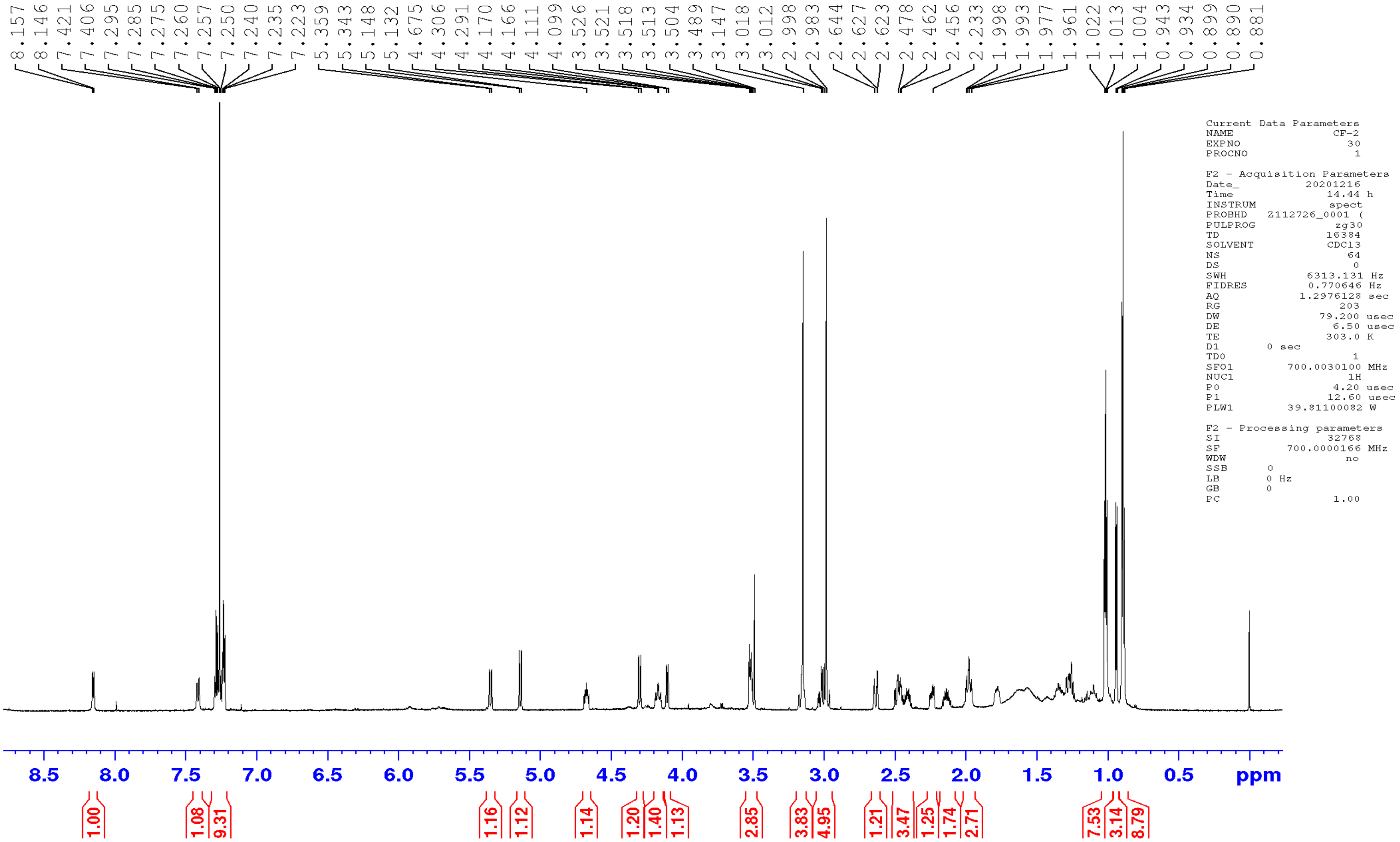

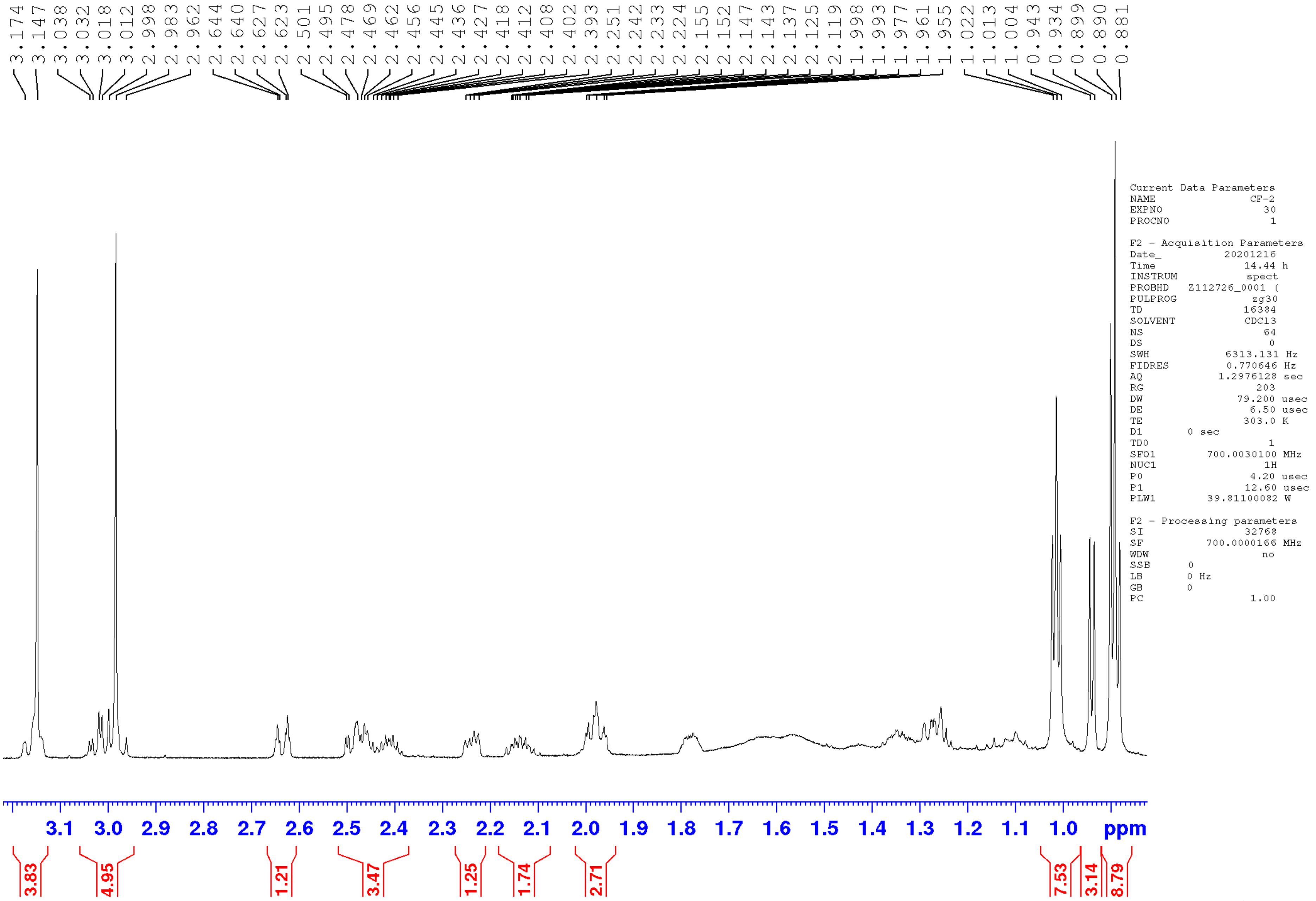
3.174
3.147
3.038
3.032
3.018
3.012
2.998
2.983
2.962
2.644
2.640
2.627
2.623
2.501
2.495
2.478
2.469
2.462
2.456
2.445
2.436
2.427
2.418
2.412
2.408
2.402
2.393
2.251
2.242
2.233
2.224
2.155
2.152
2.147
2.143
2.137
2.125
2.119
1.998
1.993
1.977
1.961
1.955
1.022
1.013
1.004
0.943
0.934
0.899
0.890
0.881
Current Data Parameters
NAME CF-2
EXPNO 30
PROCNO 1
F2 - Acquisition Parameters
Date_ 20201216
Time 14.44 h
INSTRUM spect
PROBHD Z112726_0001 (
PULPROG zg30
TD 16384
SOLVENT CDCl3
NS 64
DS 0
SWH 6313.131 Hz
FIDRES 0.770646 Hz
AQ 1.2976128 sec
RG 203
DW 79.200 usec
DE 6.50 usec
TE 303.0 K
D1 0 sec
TD0 1
SFO1 700.0030100 MHz
NUC1 1H
P0 4.20 usec
P1 12.60 usec
PLW1 39.81100082 W
F2 - Processing parameters
SI 32768
SF 700.0000166 MHz
WDW no
SSB 0
LB 0 Hz
GB 0
PC 1.00
3.1
3.0
2.9
2.8
2.7
2.6
2.5
2.4
2.3
2.2
2.1
2.0
1.9
1.8
1.7
1.6
1.5
1.4
1.3
1.2
1.1
1.0
ppm
3.83
4.95
1.21
3.47
1.25
1.74
2.71
7.53
3.14
8.79

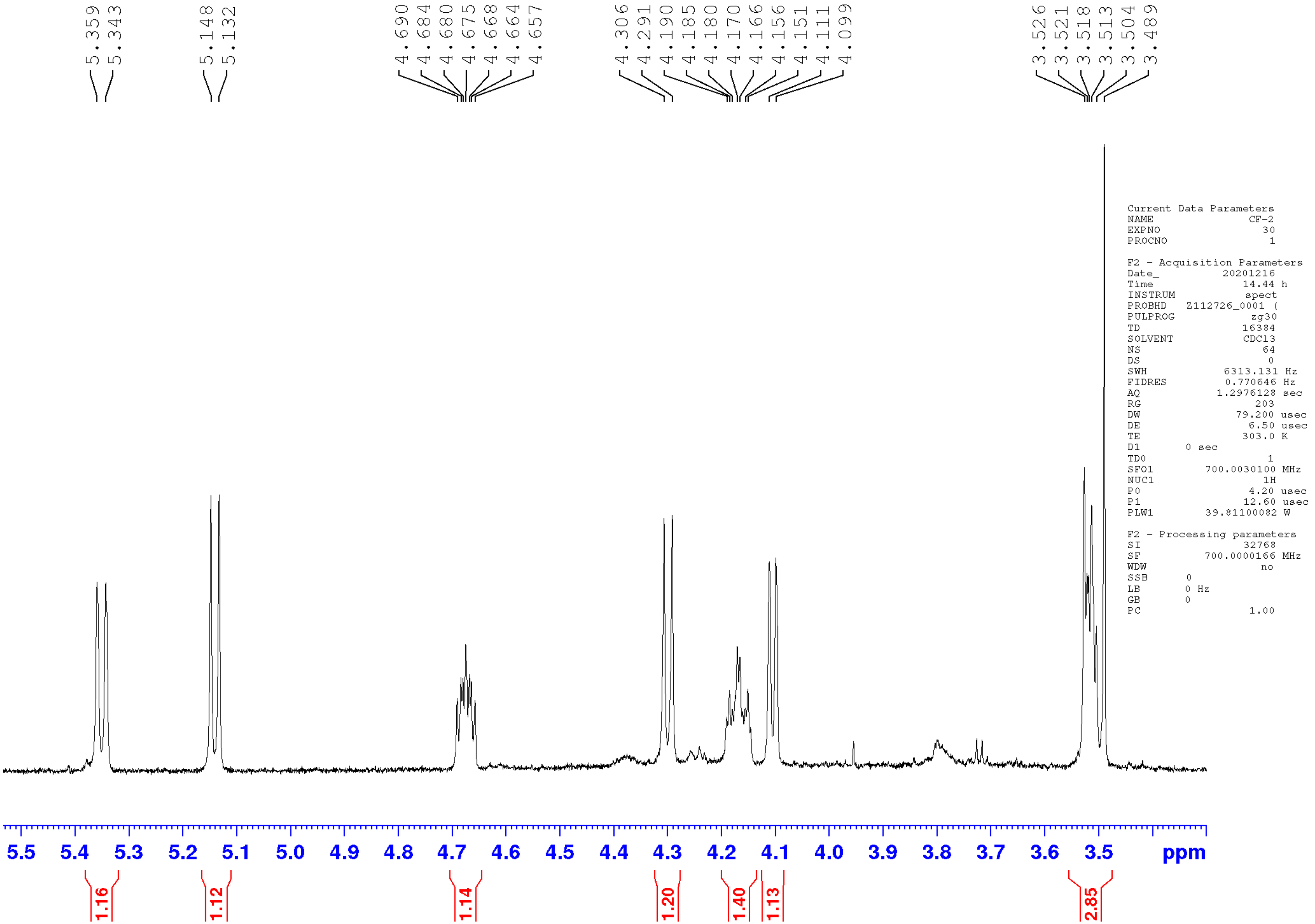
5.359
5.343
5.148
5.132
4.690
4.684
4.680
4.675
4.668
4.664
4.657
4.306
4.291
4.190
4.185
4.180
4.170
4.166
4.156
4.151
4.111
4.099
3.526
3.521
3.518
3.513
3.504
3.489
Current Data Parameters
NAME CF-2
EXPNO 30
PROCNO 1
F2 - Acquisition Parameters
Date_ 20201216
Time 14.44 h
INSTRUM spect
PROBHD Z112726_0001 (
PULPROG zg30
TD 16384
SOLVENT CDCl3
NS 64
DS 0
SWH 6313.131 Hz
FIDRES 0.770646 Hz
AQ 1.2976128 sec
RG 203
DW 79.200 usec
DE 6.50 usec
TE 303.0 K
D1 0 sec
TD0 1
SFO1 700.0030100 MHz
NUC1 1H
P0 4.20 usec
P1 12.60 usec
PLW1 39.81100082 W
F2 - Processing parameters
SI 32768
SF 700.0000166 MHz
WDW no
SSB 0
LB 0 Hz
GB 0
PC 1.00
5.5 5.4 5.3 5.2 5.1 5.0 4.9 4.8 4.7 4.6 4.5 4.4 4.3 4.2 4.1 4.0 3.9 3.8 3.7 3.6 3.5 ppm
1.16
1.12
1.14
1.20
1.40
1.13
2.85

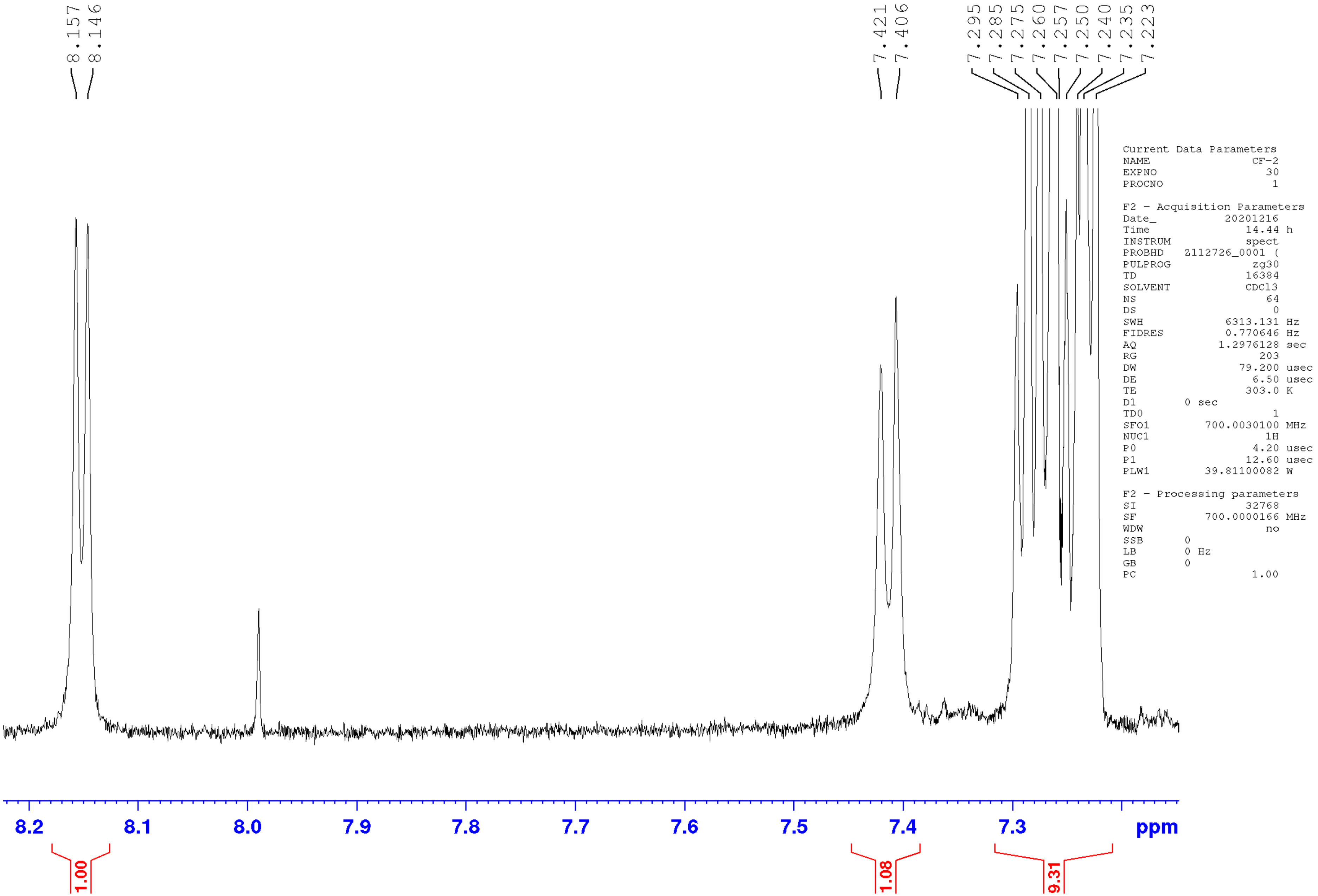
8.157
8.146
7.421
7.406
7.295
7.285
7.275
7.260
7.257
7.250
7.240
7.235
7.223
Current Data Parameters
NAME CF-2
EXPNO 30
PROCNO 1
F2 - Acquisition Parameters
Date_ 20201216
Time 14.44 h
INSTRUM spect
PROBHD Z112726_0001 (
PULPROG zg30
TD 16384
SOLVENT CDCl3
NS 64
DS 0
SWH 6313.131 Hz
FIDRES 0.770646 Hz
AQ 1.2976128 sec
RG 203
DW 79.200 usec
DE 6.50 usec
TE 303.0 K
D1 0 sec
TD0 1
SFO1 700.0030100 MHz
NUC1 1H
P0 4.20 usec
P1 12.60 usec
PLW1 39.81100082 W
F2 - Processing parameters
SI 32768
SF 700.0000166 MHz
WDW no
SSB 0
LB 0 Hz
GB 0
PC 1.00
8.2
8.1
8.0
7.9
7.8
7.7
7.6
7.5
7.4
7.3
ppm
1.00
1.08
9.31

Figure S32. ^{13}C NMR spectrum of isaridine E (11)

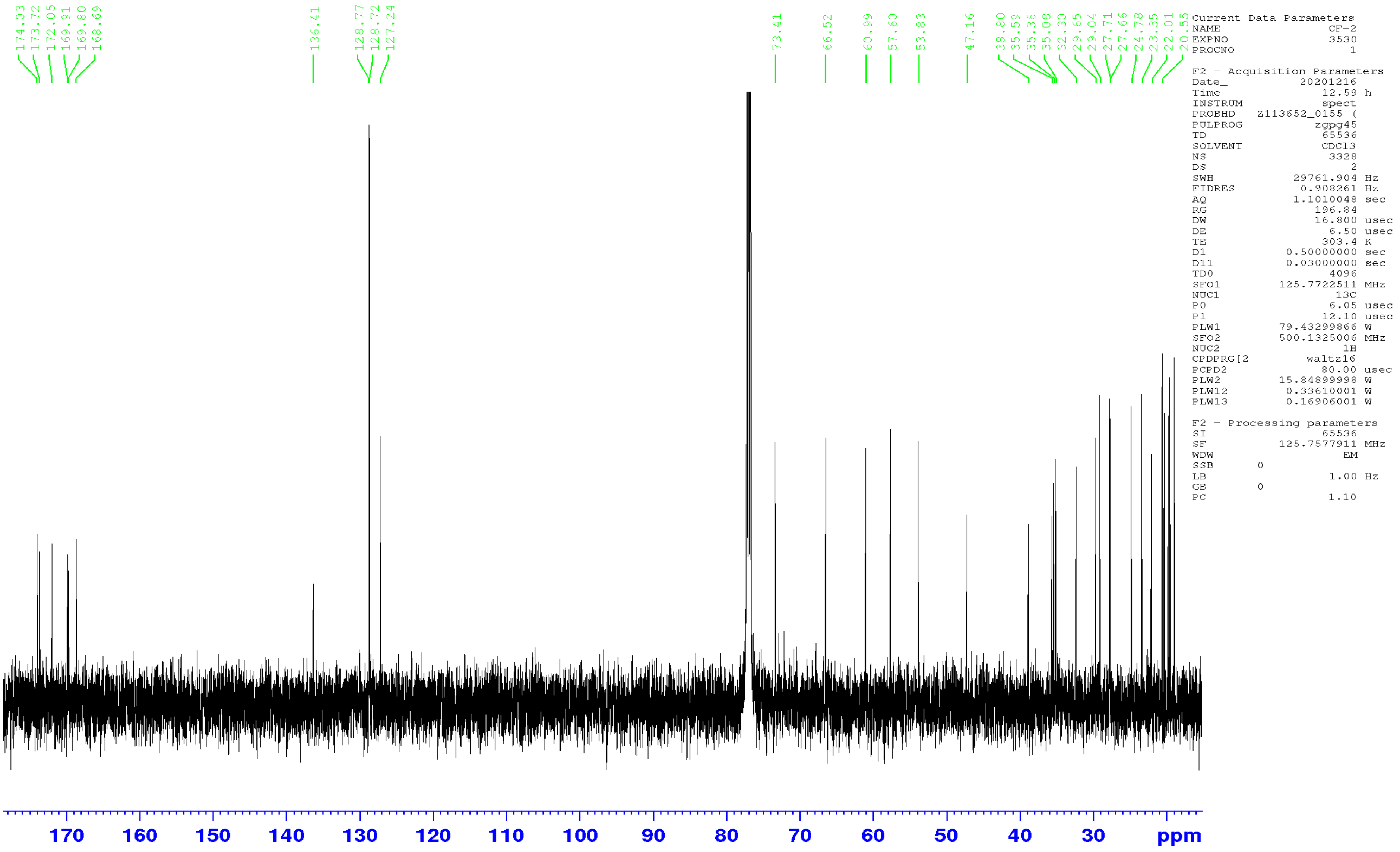

Figure S33. DEPT-135 spectrum of isaridine E (11)

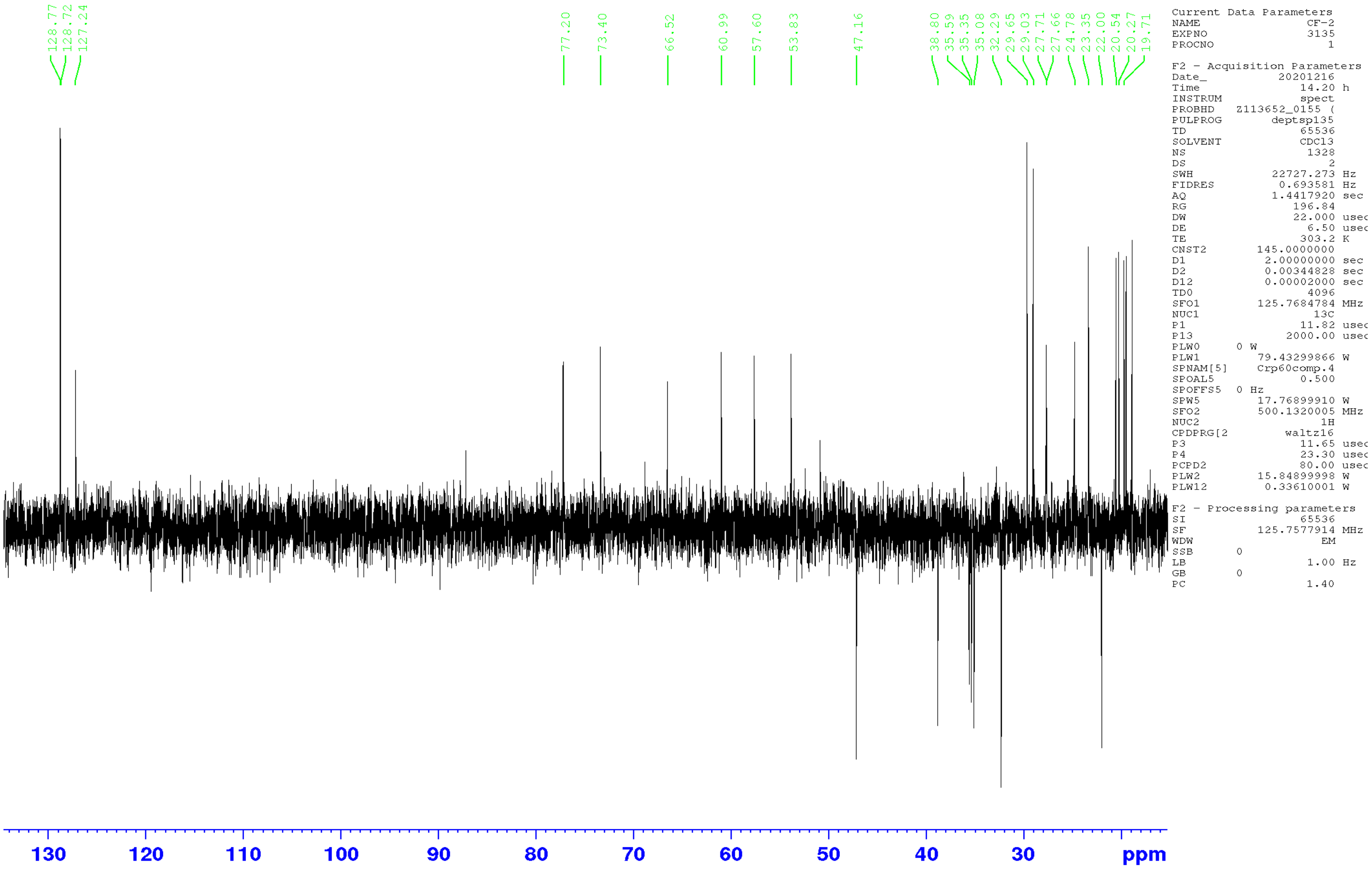

Figure S34. ^1H-^1H COSY spectrum of isaridine E (11)

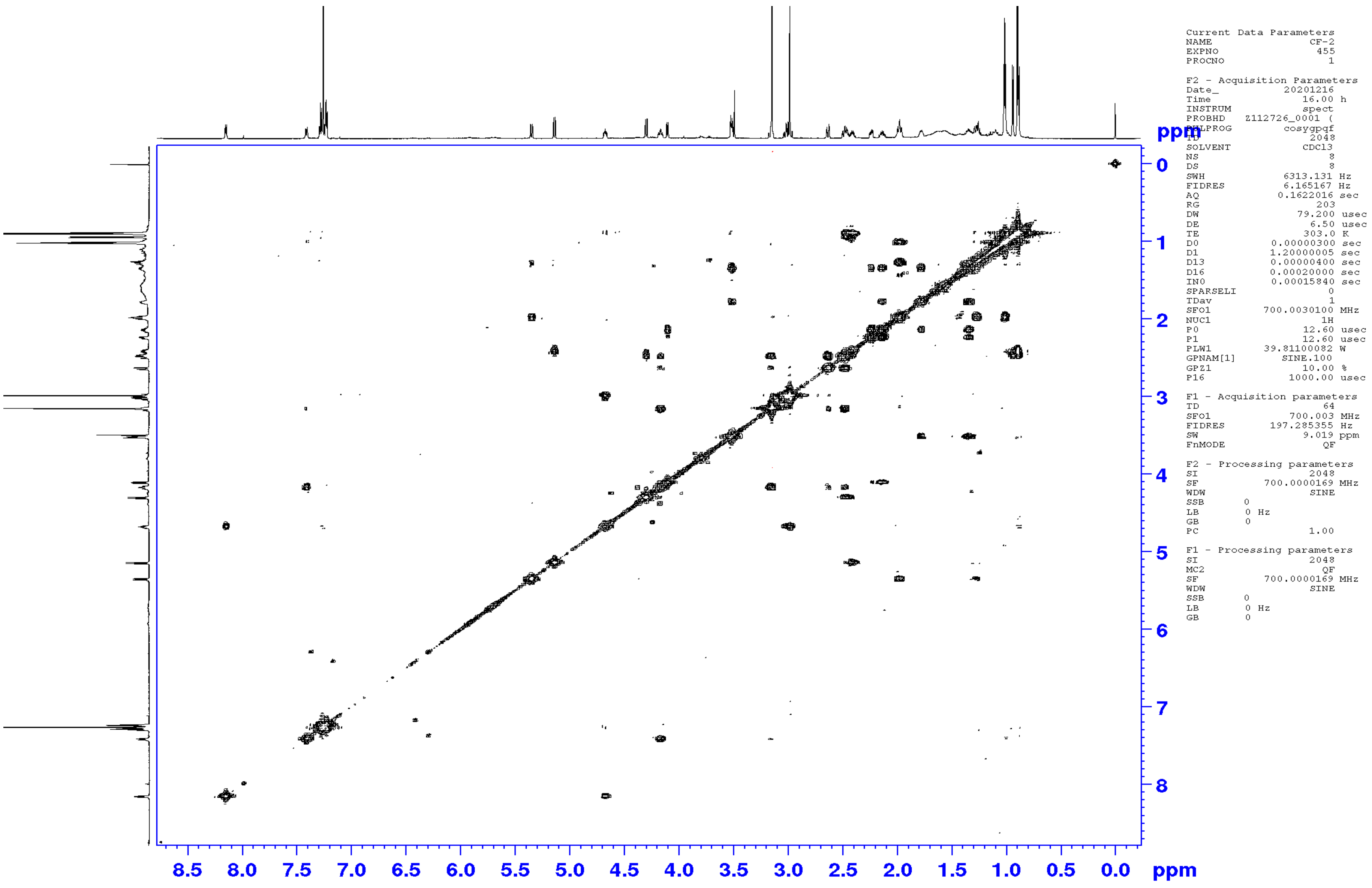

Figure S35. HSQC spectrum of isaridine E (11)

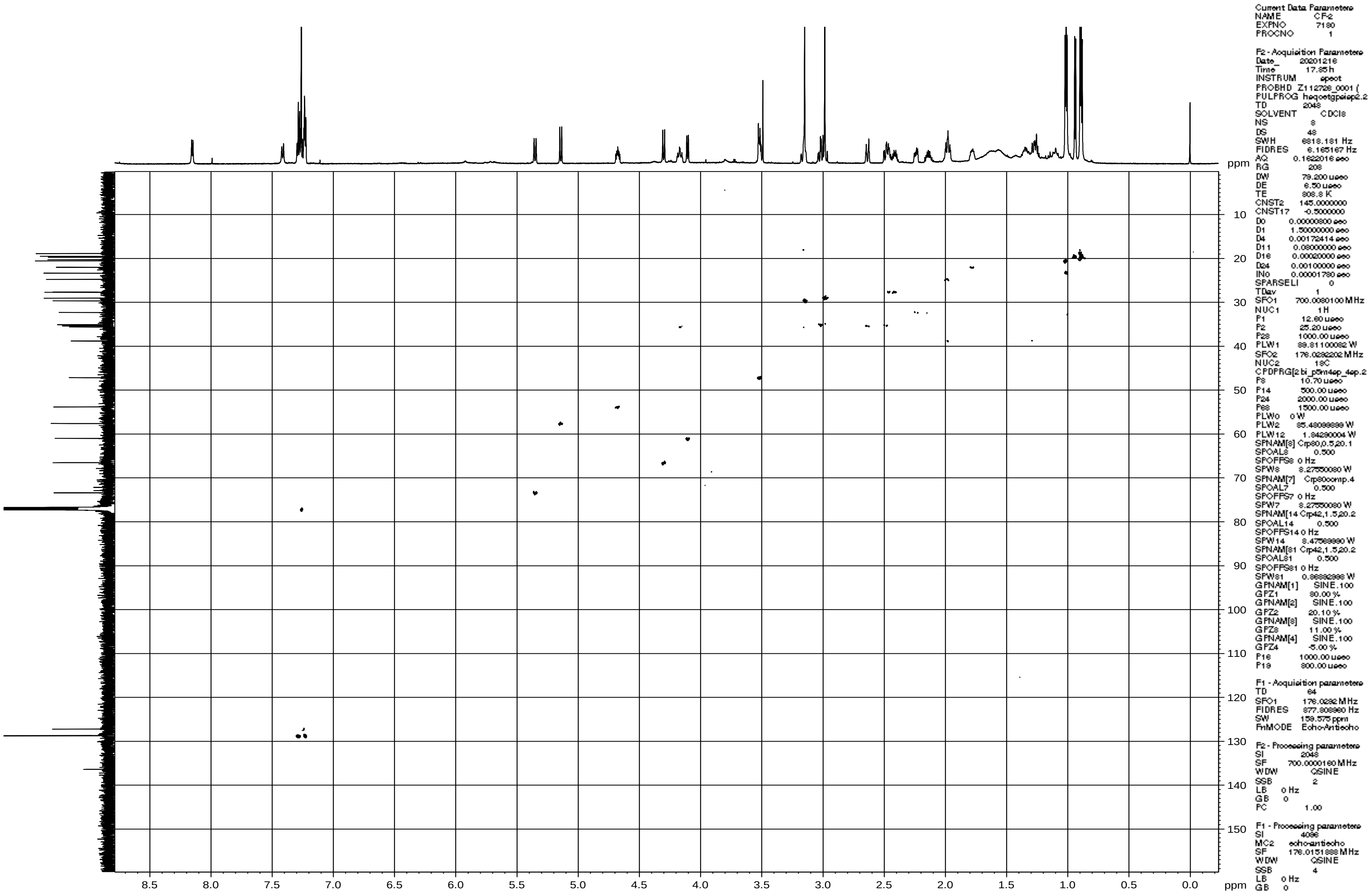

Figure S36. HMBC spectrum of isaridine E (11)

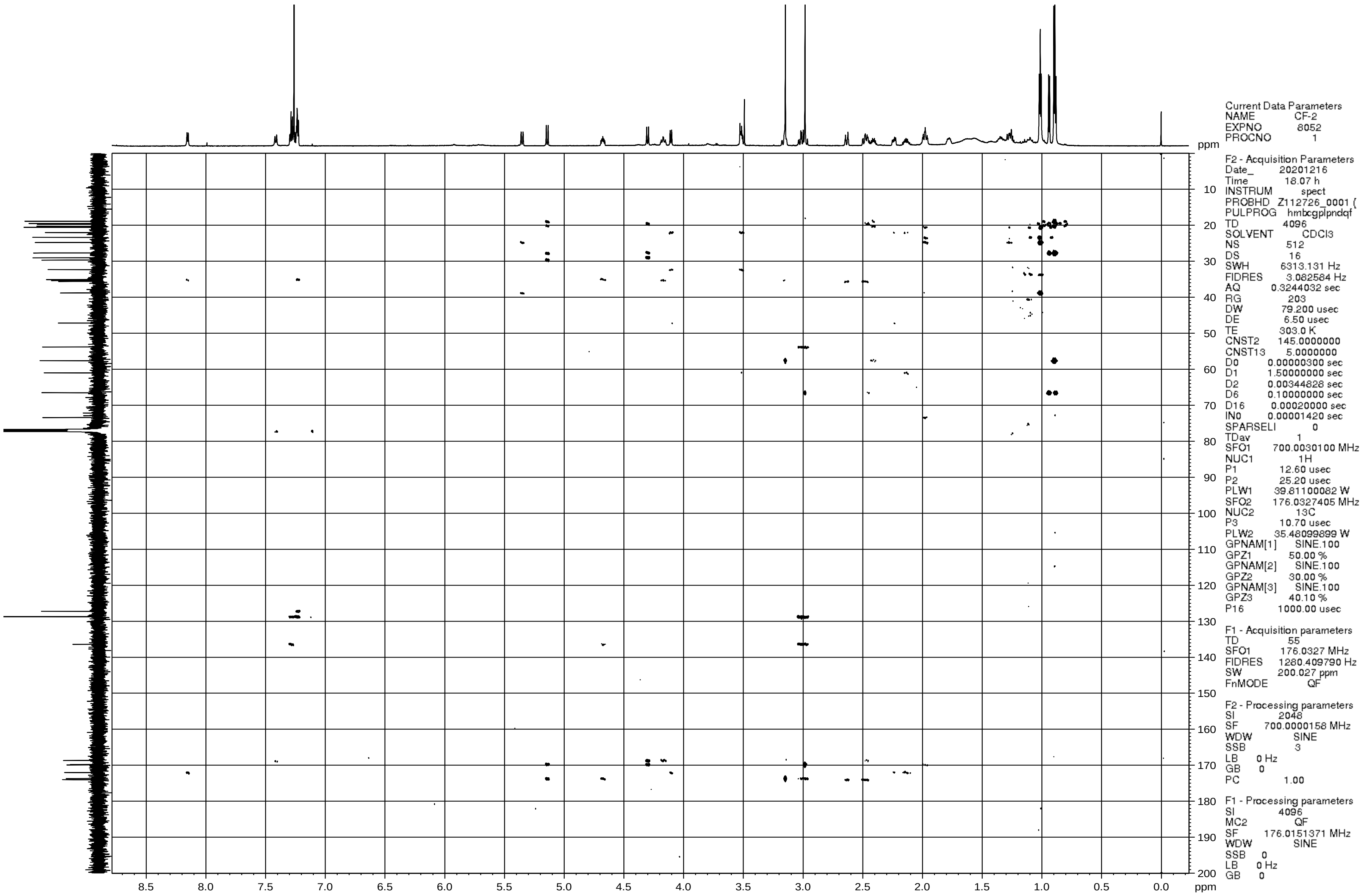

Figure S37. NOESY spectrum of isaridine E (11)

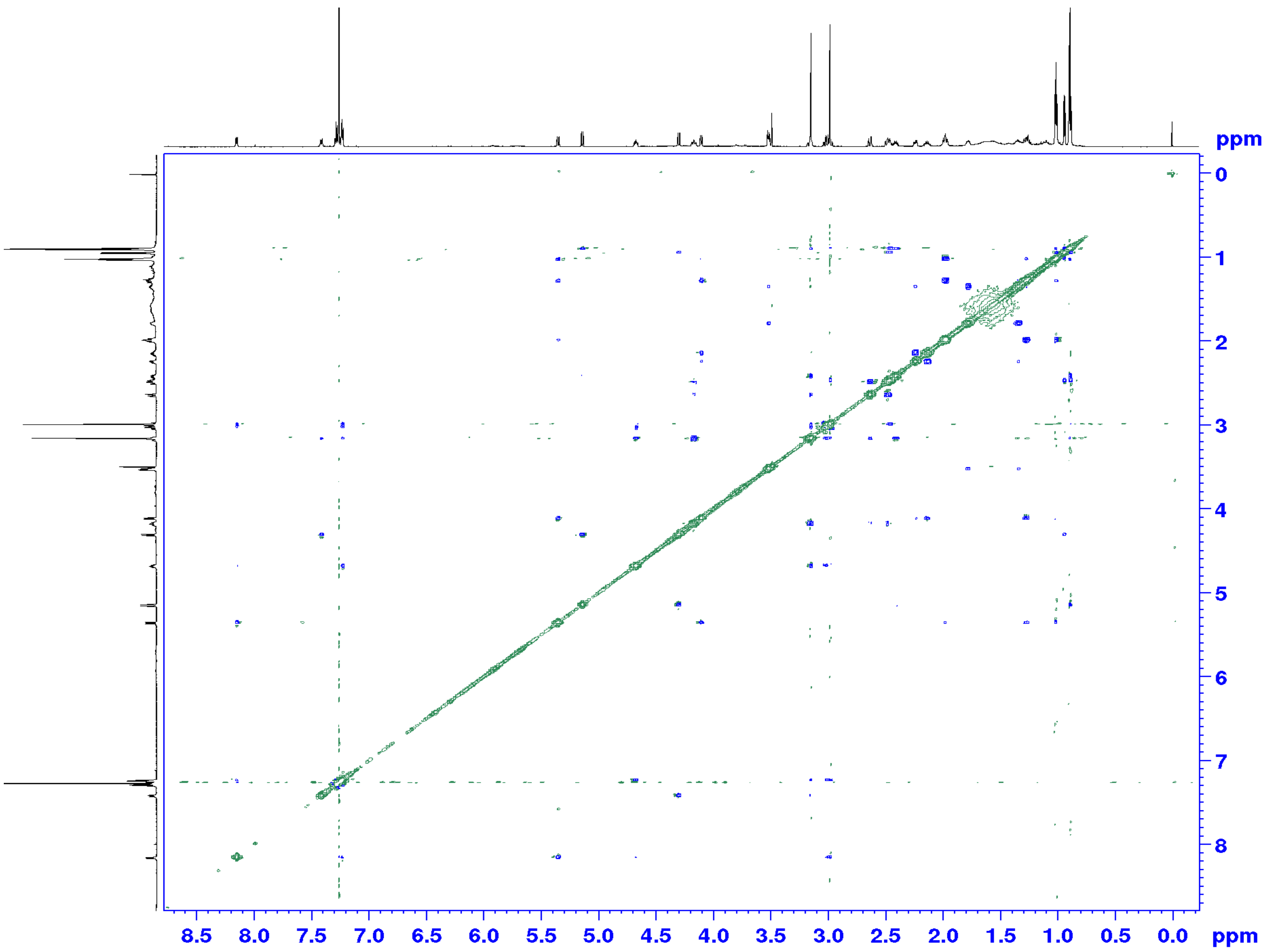


```
Current Data Parameters
NAME                CF-2
EXPNO               6030
PROCNO                 1

F2 - Acquisition Parameters
Date_           20201216
Time               16.20 h
INSTRUM            spect
PROBHD   Z112726_0001 (
PULPROG        noesygpph
TD                  2048
SOLVENT            CDCl3
NS                    16
DS                    16
SWH             6313.131 Hz
FIDRES          6.165167 Hz
AQ             0.1622016 sec
RG                   203
DW                79.200 usec
DE                  6.50 usec
TE                 303.0 K
D0            0.00006316 sec
D1            1.00000000 sec
D8            1.00000000 sec
D16           0.00020000 sec
IN0           0.00015840 sec
SPARSELI               0
TDav                   1
SFO1         700.0030100 MHz
NUC1                  1H
P1                 12.60 usec
P2                 25.20 usec
PLW1         39.81100082 W
GPNAM[1]        SINE.100
GPZ1               20.00 %
P16              1000.00 usec

F1 - Acquisition parameters
TD                   128
SFO1             700.003 MHz
FIDRES         98.642677 Hz
SW                 9.019 ppm
FnMODE              TPPI

F2 - Processing parameters
SI                  2048
SF           700.0000164 MHz
WDW                QSINE
SSB                    2
LB        0 Hz
GB        0
PC                  1.00

F1 - Processing parameters
SI                  2048
MC2                 TPPI
SF           700.0000165 MHz
WDW                QSINE
SSB                    2
LB        0 Hz
GB        0
```

Figure S38. HPLC profile of *L*-FDAA -derivatives of felicarnezoline A (1) hydrolysate

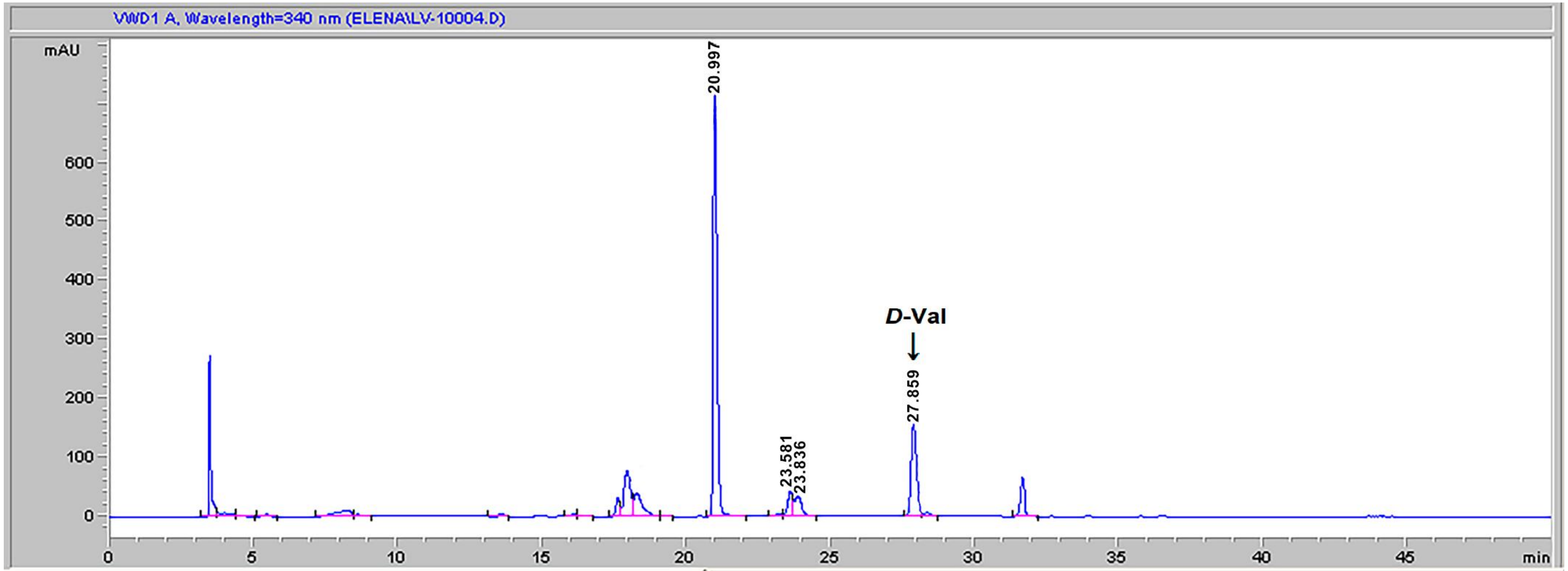

File Information

LC-File	LV-10004.D
File Path	C:\HPCHEM\1\DATA\ELENA\
Date	09-Jun-22, 16:33:36
Sample	CF-47 gidrolise
Sample Info	
Barcode	
Operator	OKSANA
Method	DEFH_LC.M
Analysis Time	49.984 min
Sampling Rate	0.0049 min (0.294 sec), 10300 datapoints

#	Time	Area	Height	Width	Symmetry
11	17.96	1253.5	78.5	0.2402	1.105
12	18.284	788.3	40.1	0.2743	0.495
13	19.262	21.9	1.9	0.1721	0.837
14	20.997	7247.8	713.3	0.1579	0.875
15	23.184	82	5.4	0.2338	1.17
16	23.581	533	41.8	0.1896	0.995
17	23.836	573.9	34.8	0.2332	0.579
18	27.859	2020.7	156.5	0.203	0.742
19	28.28	87.6	5.3	0.2492	0.51
20	31.621	710.1	65.4	0.1689	0.917

Figure S39. HPLC profile of *L*-FDAA -derivatives of felicarnezoline A (1) HP+*L*-Val

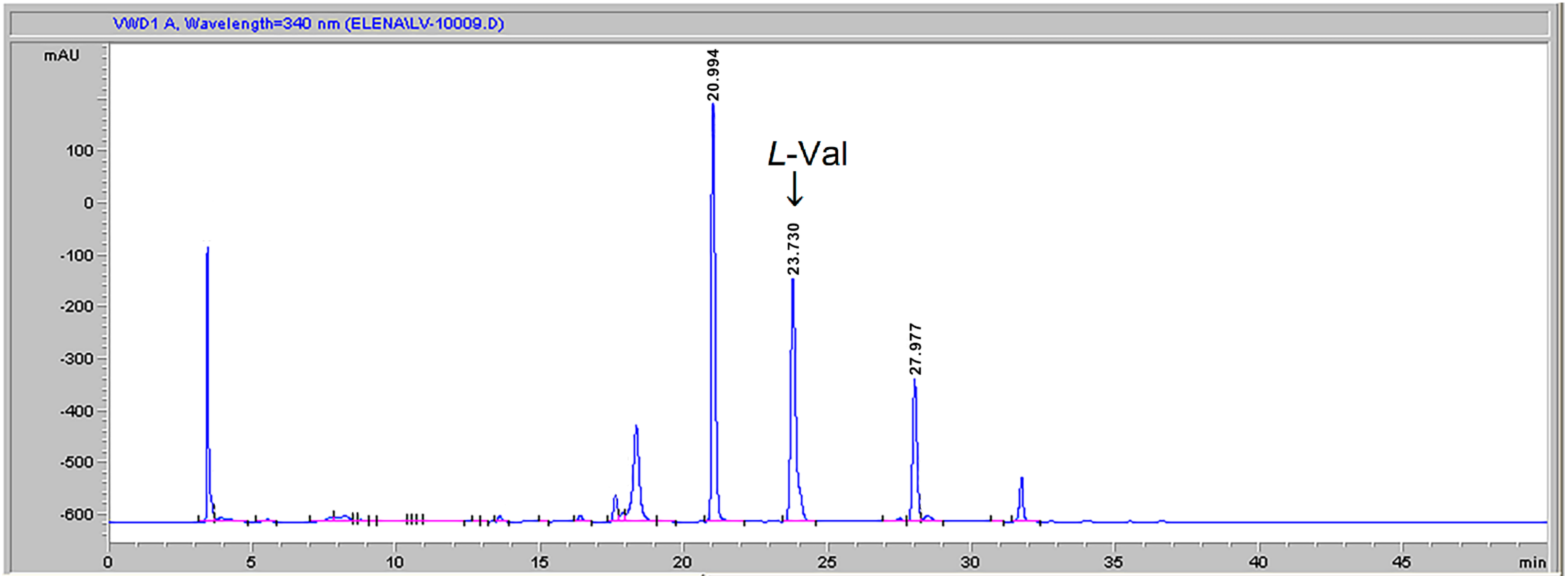

File Information

LC-File	LV-10009.D
File Path	C:\HPCHEM\1\DATA\ELENA\
Date	10-Jun-22, 14:32:12
Sample	CF-47 LVal-2
Sample Info	
Barcode	
Operator	OKSANA
Method	DEFH_LC.M
Analysis Time	49.984 min
Sampling Rate	0.0049 min (0.294 sec), 10300 datapoints

#	Time	Area	Height	Width	Symmetry
20	17.849	228.8	17.8	0.1921	1.091
21	18.315	2798.4	184.9	0.2217	1.029
22	19.248	35.8	2.8	0.1856	0.733
23	20.994	8085.1	805.7	0.1549	0.881
24	23.73	5943.5	466.9	0.1893	0.631
25	27.459	70.1	6.5	0.166	0.92
26	27.977	2762.2	271.8	0.1593	0.886
27	28.394	161.7	8.8	0.2912	0.607
28	30.81	19.6	1.9	0.1551	0.981
29	31.678	904.5	83.6	0.167	0.926

Figure S40. HPLC profile of *L*-FDAA -derivatives of felicarnezoline A (1) HP+*D*,*L*-Val

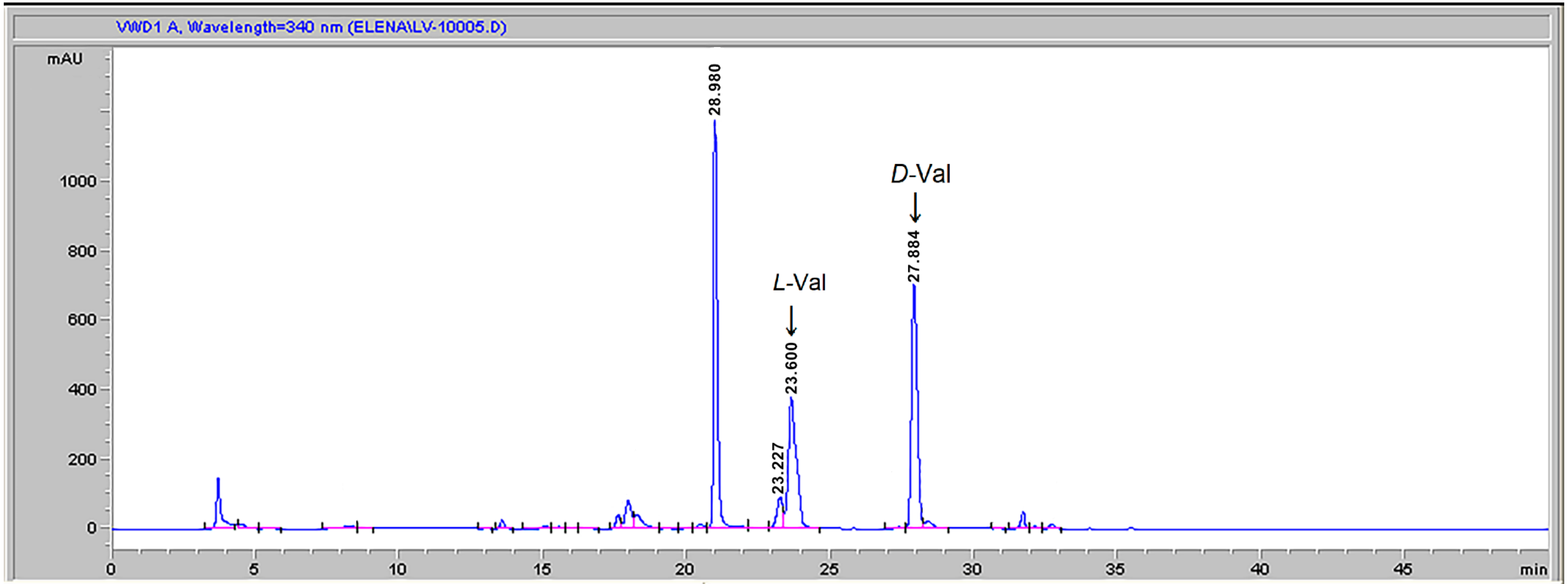

File Information

LC-File	LV-10005.D
File Path	C:\HPCHEM\1\DATA\ELENA\
Date	09-Jun-22, 17:48:13
Sample	CF-47-LD-Val
Sample Info	
Barcode	
Operator	OKSANA
Method	DEFH_LC.M
Analysis Time	49.984 min
Sampling Rate	0.0049 min (0.294 sec), 10300 datapoints

#	Time	Area	Height	Width	Symmetry
16	20.057	57.5	4.3	0.2047	1.089
17	20.419	207	12.1	0.2432	0.582
18	20.98	12982.3	1206.7	0.1663	0.807
19	22.241	156.3	4.5	0.4527	0.18
20	23.227	1330	90.3	0.2243	1.187
21	23.6	7204.3	376.3	0.2699	0.529
22	27.338	76.1	5	0.2224	0.638
23	27.884	9699.6	703.2	0.2192	0.727
24	28.366	362	21	0.2651	0.593
25	30.795	26.1	2.4	0.1629	1.035
26	31.661	513.8	46.5	0.1724	0.954

Figure S41. HPLC profiles of *L*-FDAA -derivatives of *L*-Val (a), *L*-FDAA -derivatives of *D*,*L*-Val (b), *L*-FDAA -derivatives of felicarnezoline A (1) HP (c), felicarnezoline A (1) HP+*D*,*L*-Val (d) and felicarnezoline A (1) HP+*L*-Val (f)

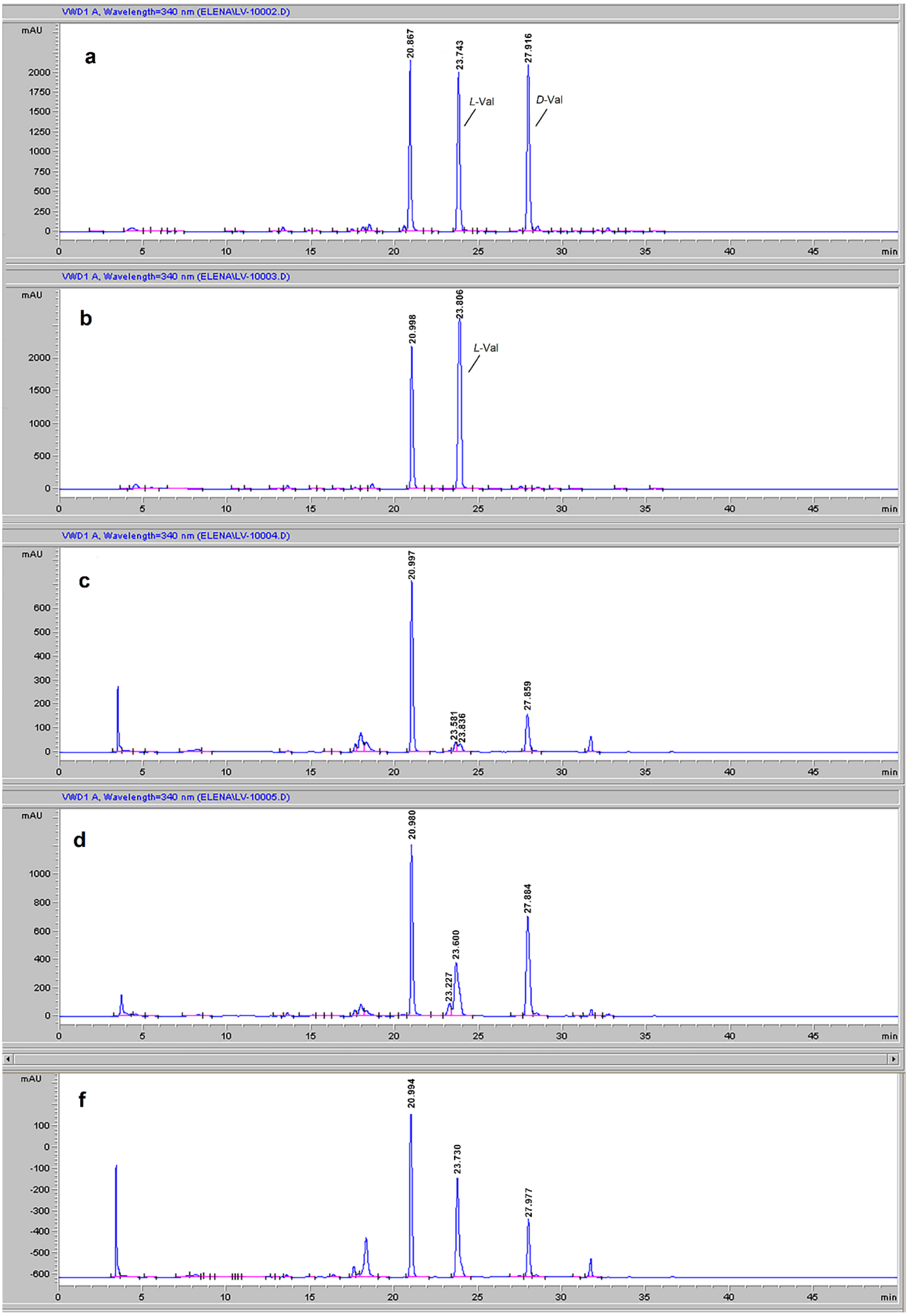

Figure S42. HPLC profile of *L*-FDAA-derivatives of felicarnezoline B (2) HP

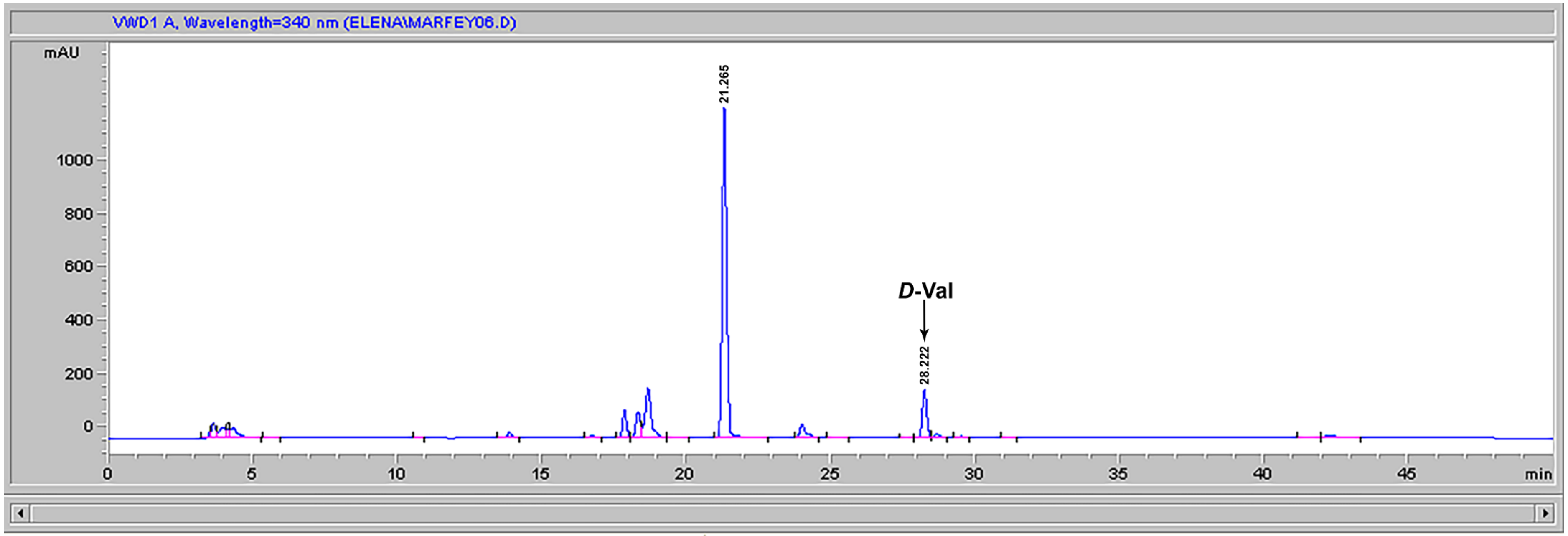

File Information	
LC-File	MARFEY06.D
File Path	C:\HPCHEM\1\DATA\ELENA\
Date	14-Jul-22, 15:35:09
Sample	CF-49-FDAA 2
Sample Info	Superdex 30
	CF-49-FDAA 2 10 mkl
Barcode	
Operator	belik
Method	LCMM.M
Analysis Time	49.994 min
Sampling Rate	0.0049 min (0.294 sec), 10302 datapoints

#	Time	Area	Height	Width	Symmetry
11	18.304	1102.7	100.6	0.1657	0.897
12	18.643	2626.4	185.2	0.2091	0.835
13	19.537	22.7	2	0.1759	0.762
14	21.265	13282.9	1237.9	0.166	0.803
15	23.995	724.4	52.9	0.2004	0.535
16	25.056	37.8	2.1	0.2492	0.567
17	27.674	27.2	1.7	0.2534	1.142
18	28.222	1888.1	180.3	0.1629	0.868
19	28.63	189.8	13	0.2252	0.771
20	29.454	63.2	5.9	0.1622	0.832
21	31.063	43.4	4	0.1655	0.814
22	41.707	83.5	4.6	0.264	1.629
23	42.235	236.1	10.2	0.3245	0.354

Figure S43. HPLC profile of *L*-FDAA -derivatives of felicarnezoline B (2) HP+*L*-Val

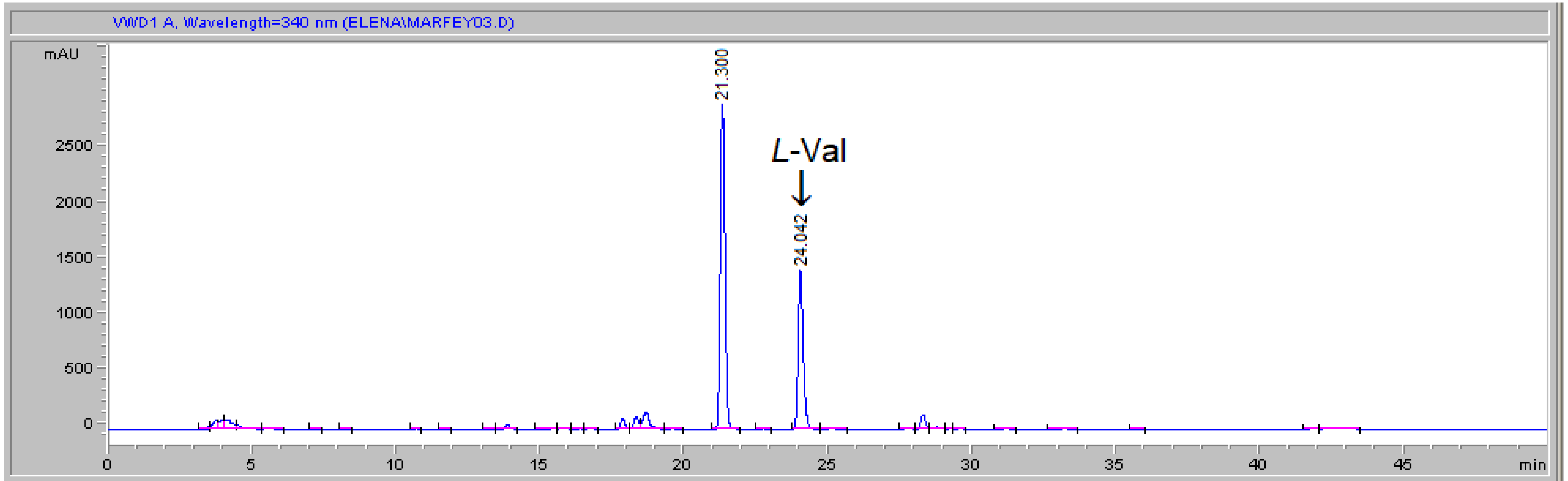

File Information

LC-File	MARFEY03.D
File Path	C:\HPCHEM\1\DATA\ELENA\
Date	14-Jul-22, 13:02:20
Sample	CF-49-L-VAL-FDAA
Sample Info	Superdex 30
	CF-49-L-VAL-FDAA 15 mkl
Barcode	
Operator	belik
Method	LCMM.M
Analysis Time	49.984 min
Sampling Rate	0.0049 min (0.294 sec), 10300 datapoints

#	Time	Area	Height	Width	Symmetry
19	18.66	2294	154.4	0.22	0.836
20	19.556	40.1	3.4	0.1745	0.803
21	21.3	32042.7	2916.4	0.1719	0.859
22	22.703	28.3	2.5	0.172	0.732
23	24.042	16924.5	1422.9	0.1824	0.762
24	25.087	65.8	4	0.2432	0.798
25	27.774	198.6	16.7	0.1821	0.923
26	28.294	1374.6	128.9	0.1652	0.868
27	28.758	282.8	17.3	0.2607	1.036
28	29.214	32.5	2.6	0.19	1.089
29	29.539	48.2	4.3	0.1725	0.859
30	31.147	63.8	5.9	0.1666	0.859
31	33.178	52.5	3.9	0.1961	1.132

Figure S44. HPLC profile of *L*-FDAA -derivatives of felicarnezoline B (2) HP+*D*,*L*-Val

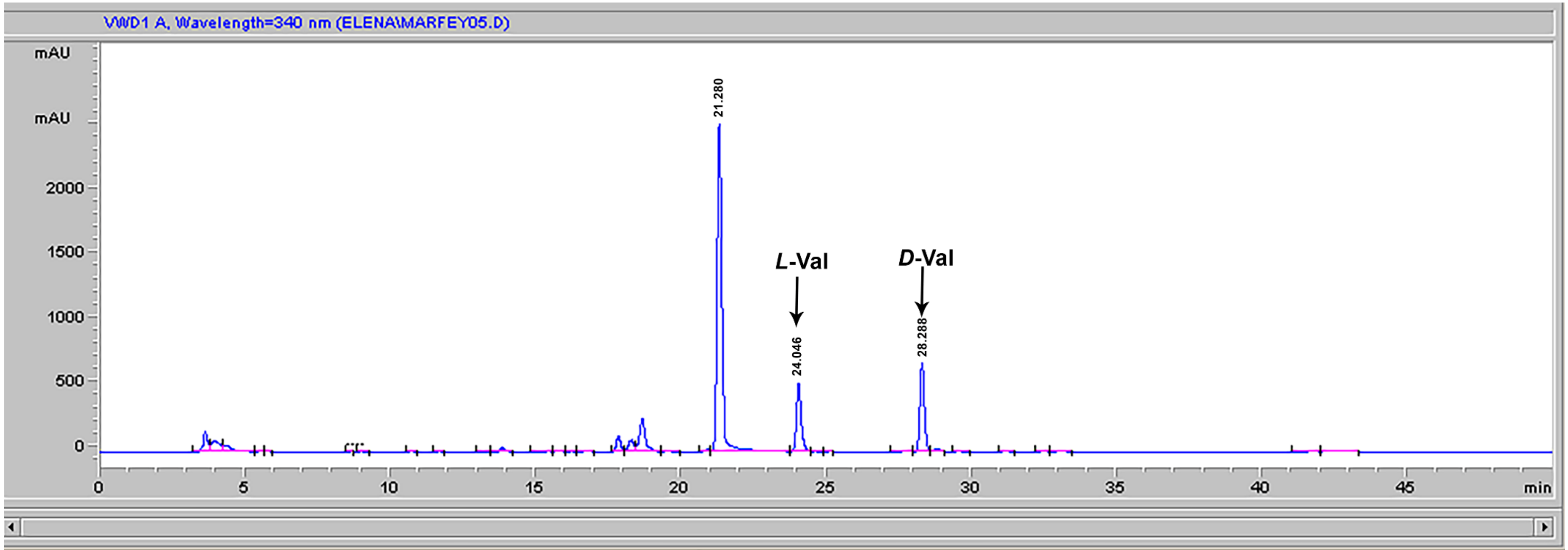

File Information

LC-File	MARFEY05.D
File Path	C:\HPCHEM\1\DATA\ELENA\
Date	14-Jul-22, 14:44:19
Sample	CF-49LD-VAL-FDAA
Sample Info	Superdex 30
	CF-49LD-VAL-FDAA 15 mkl
Barcode	
Operator	belik
Method	LCMM.M
Analysis Time	49.994 min
Sampling Rate	0.0049 min (0.294 sec), 10302 datapoints

#	Time	Area	Height	Width	Symmetry
17	18.258	1172.3	92.6	0.1914	0.789
18	18.646	3529	251.5	0.2074	0.837
19	19.536	61.4	5.4	0.17	0.77
20	20.845	201.5	19.8	0.1564	0.84
21	21.28	29994.7	2535.6	0.1817	0.696
22	24.046	5925.8	522.9	0.173	0.771
23	24.584	32.6	2.4	0.1906	0.57
24	25.094	34.1	2.5	0.2034	1.014
25	27.769	84.3	6.1	0.2018	1.168
26	28.288	7234.9	691.8	0.1599	0.867
27	28.781	381.8	24.1	0.2544	1.227
28	29.529	61.4	5.7	0.1664	0.841
29	31.133	72.5	6.9	0.1607	0.843

Figure S45. HPLC profiles of *L*-FDAA -derivatives of *L*-Val (a), *L*-FDAA -derivatives of *D*,*L*-Val (b), *L*-FDAA -derivatives of felicarnezoline B (2) HP (c), felicarnezoline B (2) HP+*D*,*L*-Val (d) and felicarnezoline B HP+*L*-Val (f)

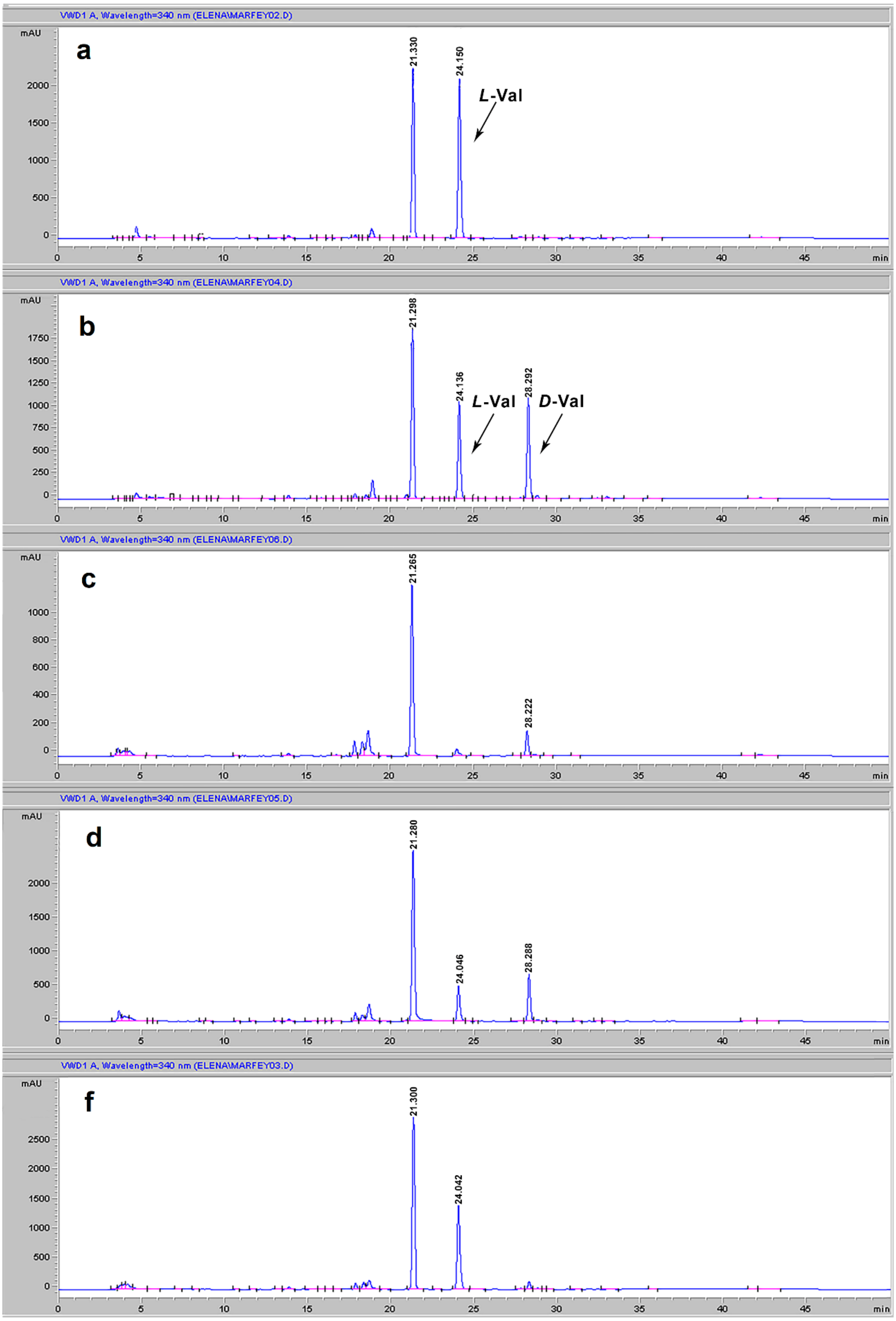

Figure S46. HPLC profile of *L*-FDAA -derivatives of felicarnezoline C (3) HP

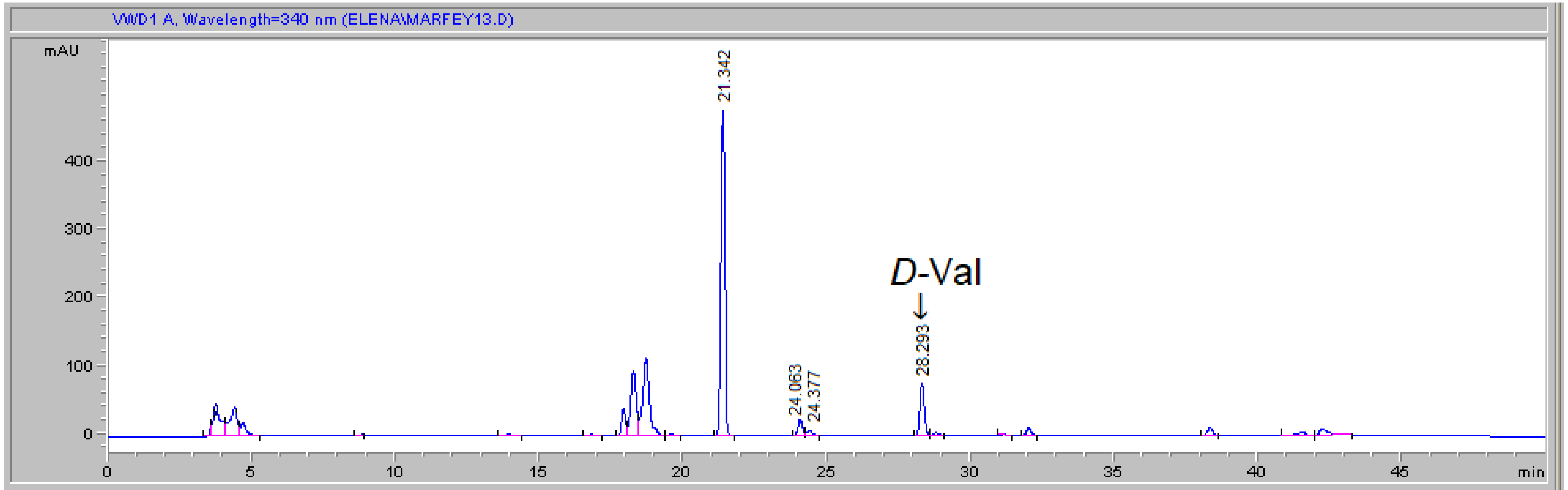

File Information

LC-File	MARFEY13.D
File Path	C:\HPCHEM\1\DATA\ELENA\
Date	15-Jul-22, 15:30:34
Sample	CF-29
Sample Info	Superdex 30
	CF-29 10 mkl
Barcode	
Operator	belik
Method	LCMM.M
Analysis Time	49.994 min
Sampling Rate	0.0049 min (0.294 sec), 10302 datapoints

#	Time	Area	Height	Width	Symmetry
9	18.256	1284.1	96.9	0.2026	0.945
10	18.686	1803.7	114.9	0.2312	0.911
11	19.597	32.6	3	0.1629	0.851
12	21.342	4859.2	474.9	0.1587	0.884
13	24.063	248.3	20.6	0.1843	0.833
14	24.377	101.1	8.2	0.1883	0.791
15	28.293	825.1	77.7	0.1647	0.871
16	28.756	65.6	3.8	0.2751	0.787
17	31.125	19.6	1.9	0.1606	0.97
18	32.015	136.8	12	0.1766	0.948
19	38.315	142	10.8	0.2024	0.89
20	41.468	114.9	5.2	0.3205	1.506
21	42.227	243.5	10.2	0.3305	0.352

Figure S47. HPLC profile of *L*-FDAA -derivatives of felicarnezoline C (3) HP +*L*-Val

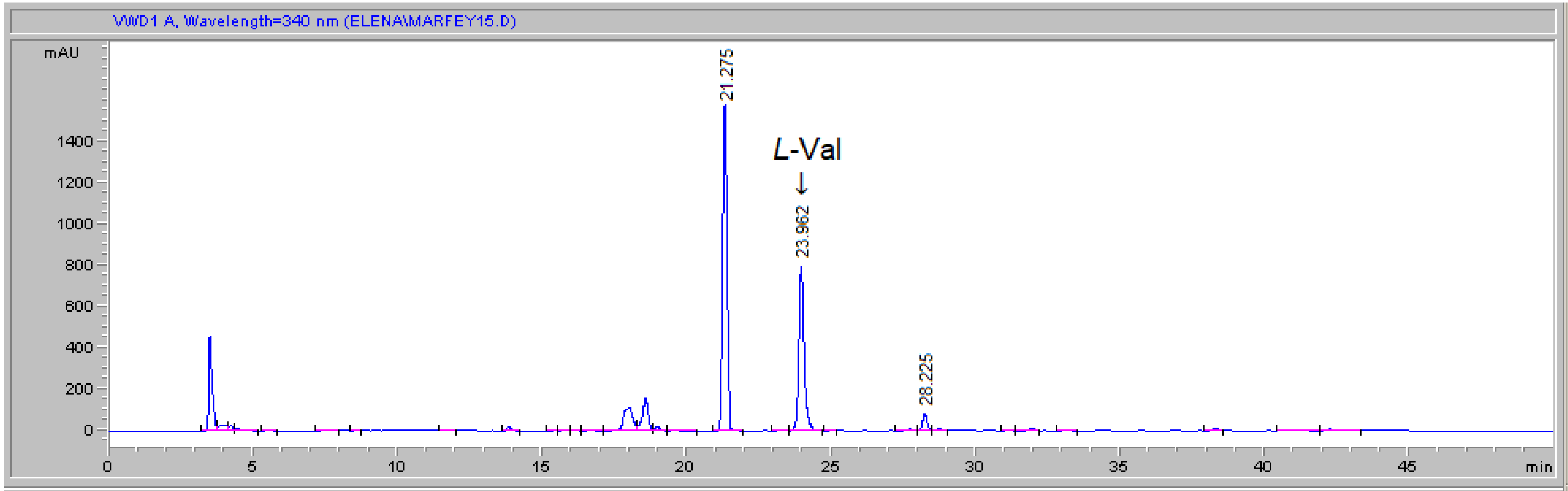

File Information

LC-File	MARFEY15.D
File Path	C:\HPCHEM\1\DATA\ELENA\
Date	15-Jul-22, 17:12:00
Sample	CF-29-L-VAL
Sample Info	Superdex 30
	CF-29-L-VAL 15 mkl
Barcode	
Operator	belik
Method	LCMM.M
Analysis Time	49.994 min
Sampling Rate	0.0049 min (0.294 sec), 10302 datapoints

#	Time	Area	Height	Width	Symmetry
11	15.783	44.5	3.1	0.2218	1.017
12	16.112	28.4	2.1	0.1983	0.845
13	16.632	58.3	4	0.2083	0.772
14	17.989	2522.6	110.5	0.3101	1.33
15	18.556	2221.2	159.7	0.203	1.207
16	18.927	249.2	19.9	0.1871	0.793
17	19.528	56.1	5	0.1724	0.81
18	21.275	16360.6	1577.8	0.1617	0.862
19	23.274	125	8.4	0.2295	0.928
20	23.962	9750.2	796.5	0.1836	0.789
21	25.002	34.3	2.5	0.2015	1.111
22	27.692	144.4	10.5	0.2017	1.022
23	28.225	923.2	84.6	0.1681	0.845

Figure S48. HPLC profile of *L*-FDAA -derivatives of felicarnezoline C (3) HP *D,L*-Val

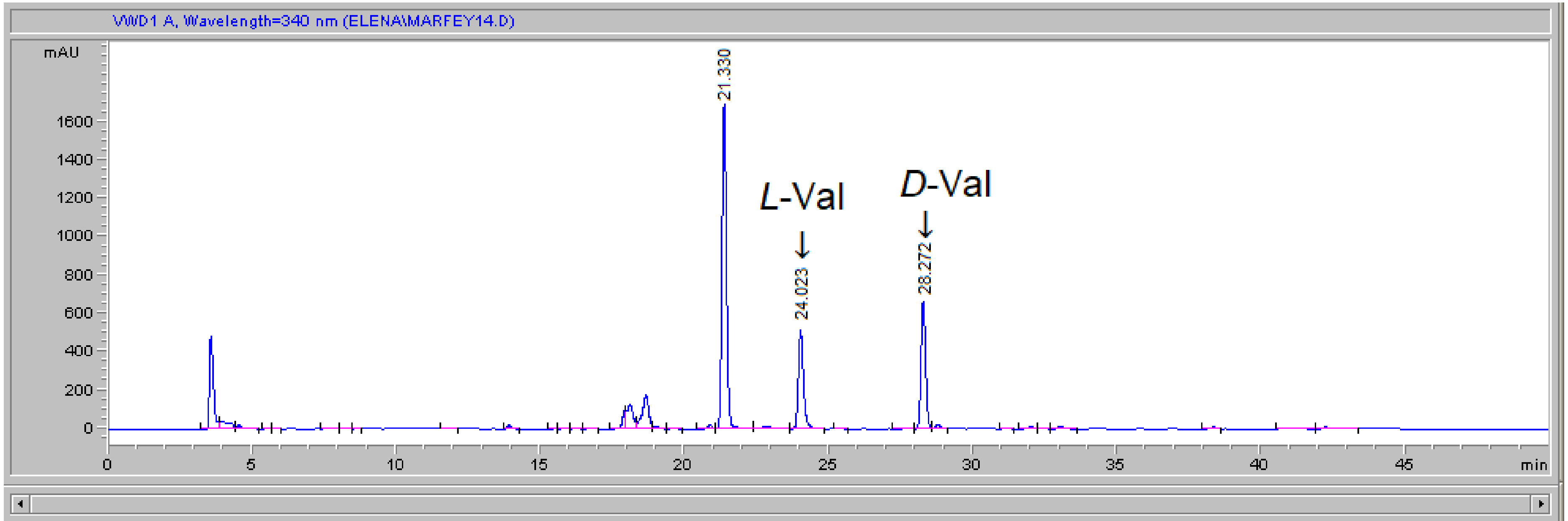

File Information

LC-File	MARFEY14.D
File Path	C:\HPCHEM\1\DATA\ELENA\
Date	15-Jul-22, 16:21:28
Sample	CF-29-LD-VAL
Sample Info	Superdex 30
	CF-29-LD-VAL 15 mkl
Barcode	
Operator	belik
Method	LCMM.M
Analysis Time	49.984 min
Sampling Rate	0.0049 min (0.294 sec), 10300 datapoints

#	Time	Area	Height	Width	Symmetry
11	15.446	19.4	2.1	0.1447	0.843
12	15.798	45.2	3.5	0.1996	0.738
13	16.188	33.1	2.5	0.198	0.8
14	16.717	50.4	3.7	0.2004	0.871
15	17.927	852	95.5	0.1346	2.169
16	18.1	1711.7	124.8	0.2037	0.834
17	18.635	2800.8	177.4	0.2278	1.381
18	18.976	179.2	15.5	0.1725	0.596
19	19.577	92.3	8.5	0.1651	0.8
20	20.846	233.7	20.8	0.1716	0.851
21	21.33	18039.5	1691	0.1652	0.824
22	22.836	475.1	9.9	0.715	0.902
23	24.023	6245.4	512.5	0.1859	0.775

F

Figure S49. HPLC profiles of *L*-FDAA -derivatives of *L*-Val (a), *L*-FDAA -derivatives of *D*,*L*-Val (b), *L*-FDAA -derivatives of felicarnezoline C (3) HP (c), felicarnezoline C (3) HP+*D*,*L*-Val (d) and felicarnezoline C (3) HP+*L*-Val (f)

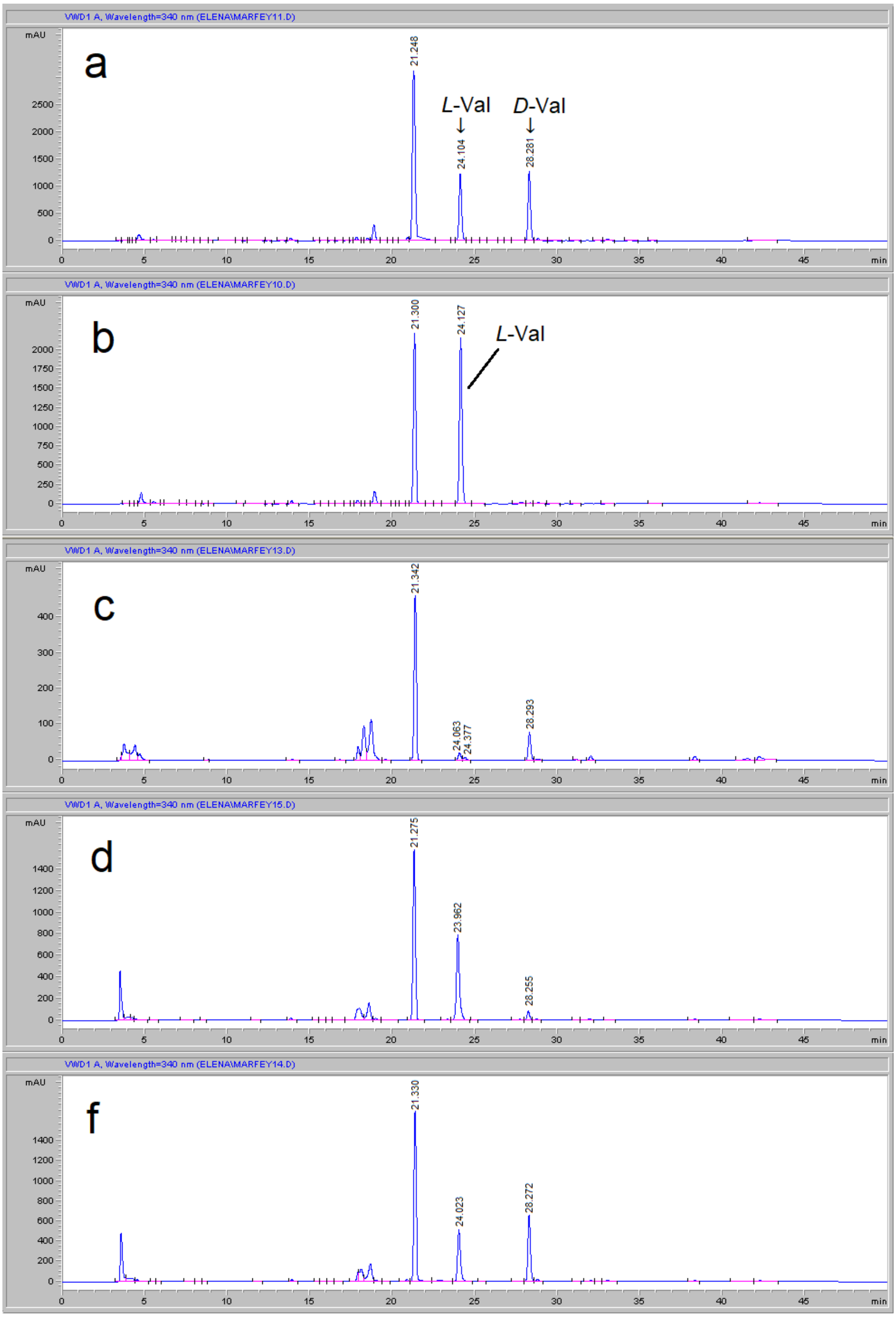

Figure S50. HPLC profile of *L*-FDAA -derivatives of felicarnezoline D (4) HP

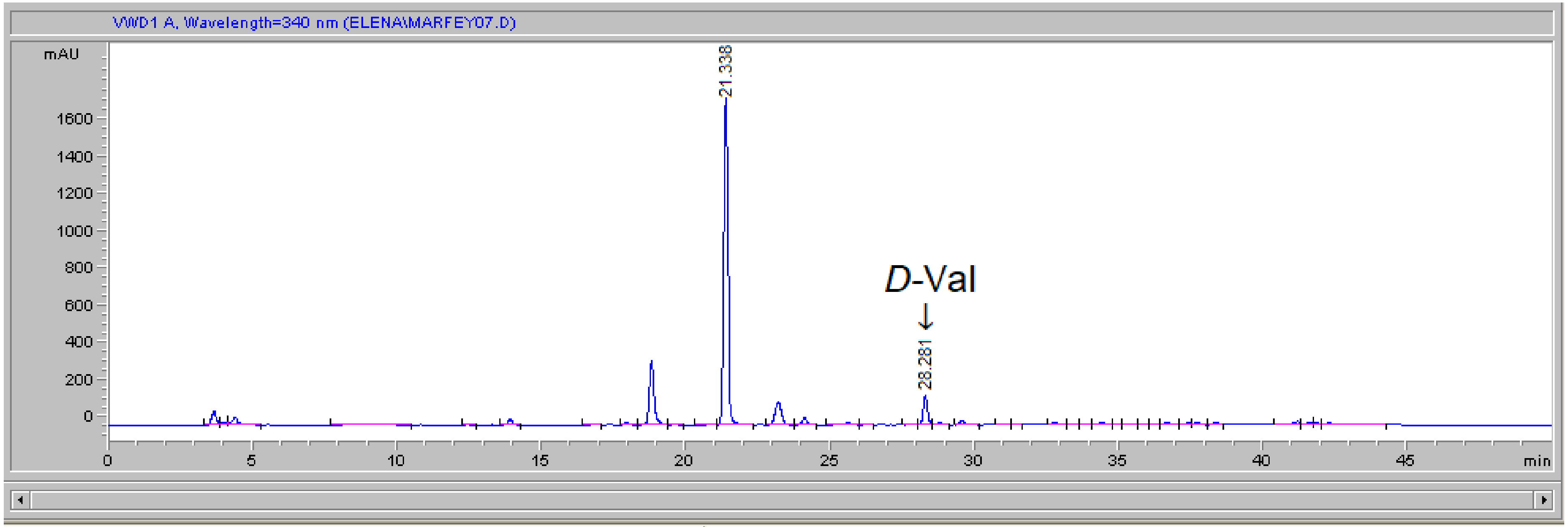

File Information	
LC-File	MARFEY07.D
File Path	C:\HPCHEM\1\DATA\ELENA\
Date	14-Jul-22, 16:55:47
Sample	CF-18-FDAA
Sample Info	Superdex 30
	CF-18-FDAA 2 10 mkl
Barcode	
Operator	belik
Method	LCMM.M
Analysis Time	49.994 min
Sampling Rate	0.0049 min (0.294 sec), 10302 datapoints

#	Time	Area	Height	Width	Symmetry
11	20.875	35.6	2.1	0.2433	1.634
12	21.338	19074.4	1748.4	0.168	0.801
13	23.155	1877	121.4	0.2386	0.924
14	24.097	413.8	35.6	0.1763	0.817
15	25.581	292.5	17	0.2505	1.333
16	26.271	32.4	2.4	0.194	1.23
17	27.843	107.6	5.5	0.2758	1.579
18	28.281	1632.5	158.4	0.1581	0.877
19	28.752	187.8	11.5	0.2367	1.241
20	29.513	236.8	20.1	0.1785	0.691
21	31.105	78.1	4.7	0.2311	1.608
22	31.326	33.6	2.2	0.2155	0.345
23	32.732	219.8	14.4	0.2217	0.623

Figure S51. HPLC profile of *L*-FDAA -derivatives of felicarnezoline D (4) HP +*L*-Val

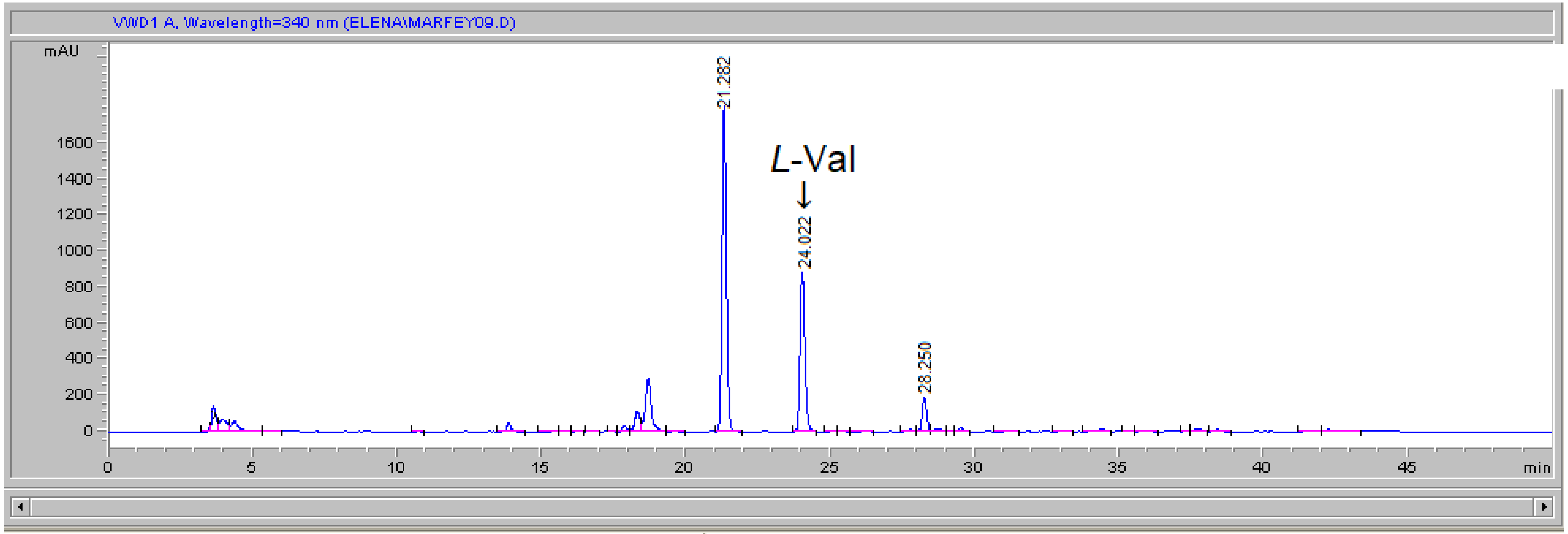

File Information

LC-File	MARFEY09.D
File Path	C:\HPCHEM\1\DATA\ELENA\
Date	15-Jul-22, 12:05:16
Sample	CF-18-L-VAL
Sample Info	Superdex 30
	CF-18-L-VAL 15 mkl
Barcode	
Operator	belik
Method	LCMM.M
Analysis Time	49.984 min
Sampling Rate	0.0049 min (0.294 sec), 10300 datapoints

#	Time	Area	Height	Width	Symmetry
16	19.538	57.3	5.1	0.1679	0.775
17	21.282	18599.6	1803.7	0.1611	0.864
18	24.022	10040.6	894.1	0.1718	0.793
19	25.066	44.3	3.3	0.2077	1.009
20	25.308	25.6	2.3	0.166	0.733
21	26.255	39.8	2.8	0.1978	1.454
22	27.736	173.8	12.5	0.2118	1.011
23	28.25	1993.1	188.2	0.1643	0.864
24	28.711	291.3	17.8	0.2613	1.064
25	29.174	20.2	1.7	0.177	1.252
26	29.488	258.4	23.4	0.1667	0.812
27	31.096	100	7.1	0.2049	0.606
28	32.972	67.2	3.4	0.2663	0.408

Figure S52. HPLC profile of *L*-FDAA -derivatives of felicarnezoline D (4) HP *D,L*-Val

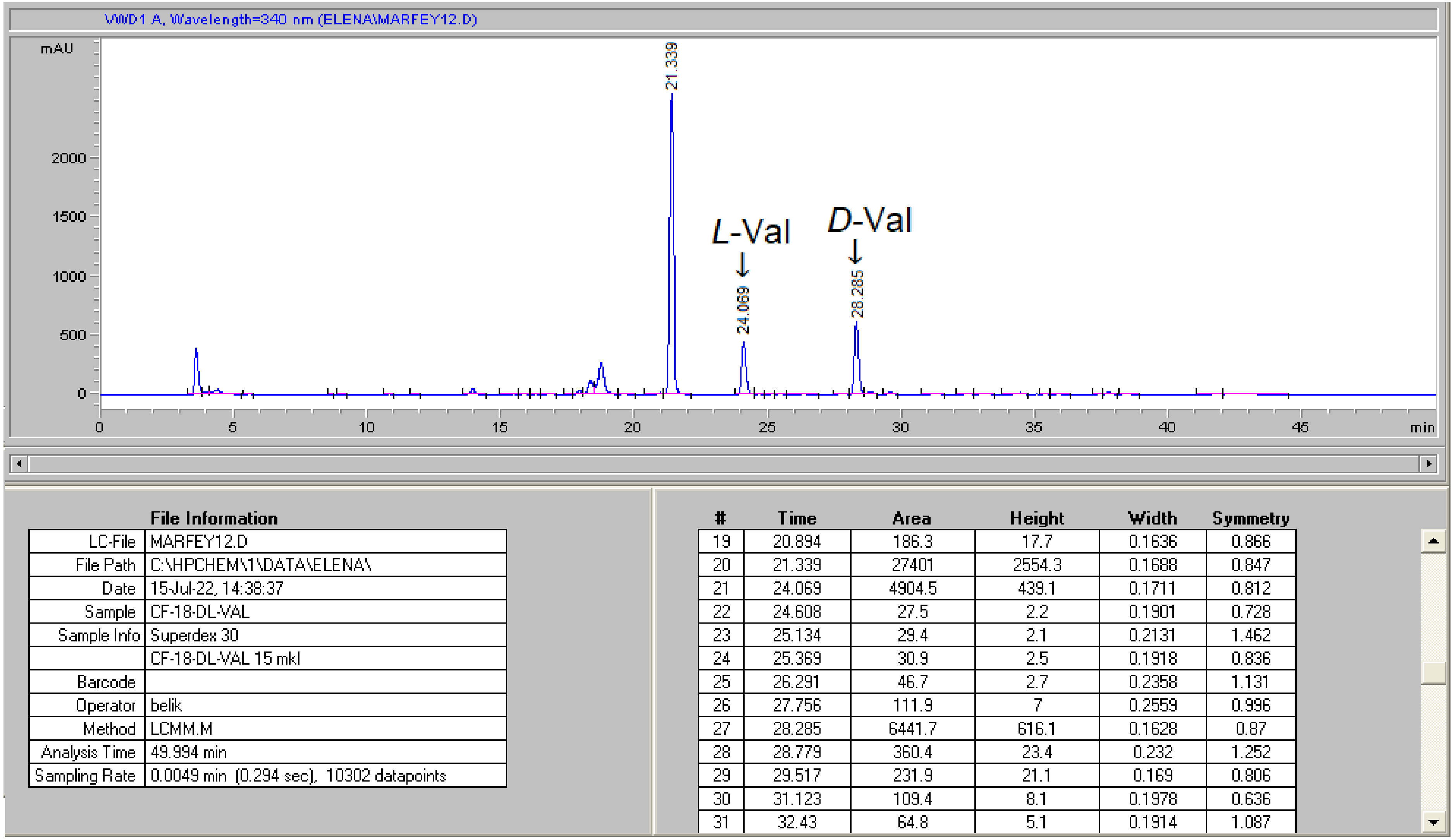

File Information

LC-File	MARFEY12.D
File Path	C:\HPCHEM\1\DATA\ELENA\
Date	15-Jul-22, 14:38:37
Sample	CF-18-DL-VAL
Sample Info	Superdex 30
	CF-18-DL-VAL 15 mkl
Barcode	
Operator	belik
Method	LCMM.M
Analysis Time	49.994 min
Sampling Rate	0.0049 min (0.294 sec), 10302 datapoints

#	Time	Area	Height	Width	Symmetry
19	20.894	186.3	17.7	0.1636	0.866
20	21.339	27401	2554.3	0.1688	0.847
21	24.069	4904.5	439.1	0.1711	0.812
22	24.608	27.5	2.2	0.1901	0.728
23	25.134	29.4	2.1	0.2131	1.462
24	25.369	30.9	2.5	0.1918	0.836
25	26.291	46.7	2.7	0.2358	1.131
26	27.756	111.9	7	0.2559	0.996
27	28.285	6441.7	616.1	0.1628	0.87
28	28.779	360.4	23.4	0.232	1.252
29	29.517	231.9	21.1	0.169	0.806
30	31.123	109.4	8.1	0.1978	0.636
31	32.43	64.8	5.1	0.1914	1.087

Figure S53. HPLC profiles of *L*-FDAA -derivatives of *L*-Val (a), *L*-FDAA -derivatives of *D*,*L*-Val (b), *L*-FDAA -derivatives of felicarnezoline D (4) HP (c), felicarnezoline D (4) HP+*D*,*L*-Val (d) and felicarnezoline D (4) HP+*L*-Val (f)

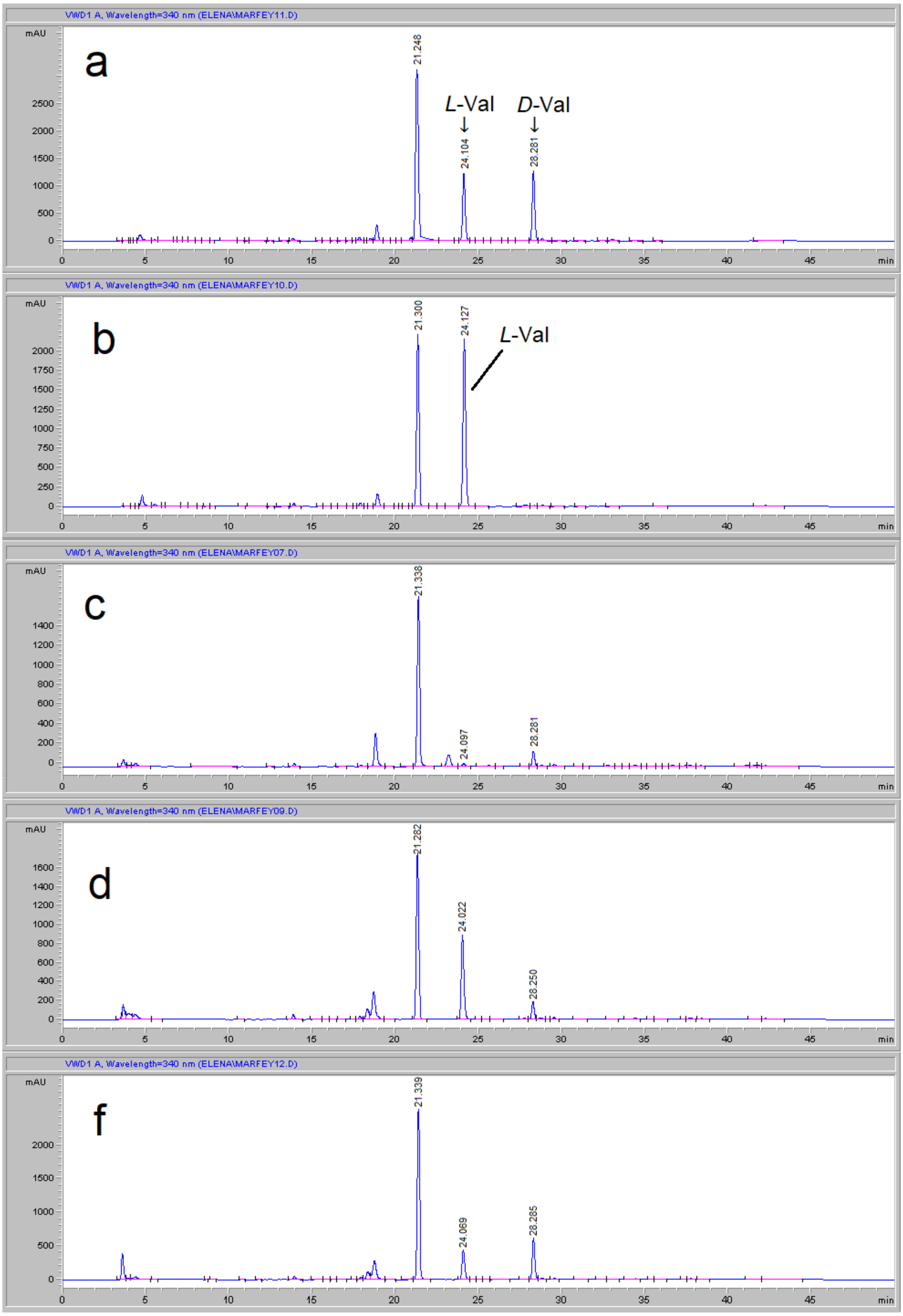

Figure S54. HPLC profile of *L*-FDAA -derivatives of felicarnezoline E (5) HP

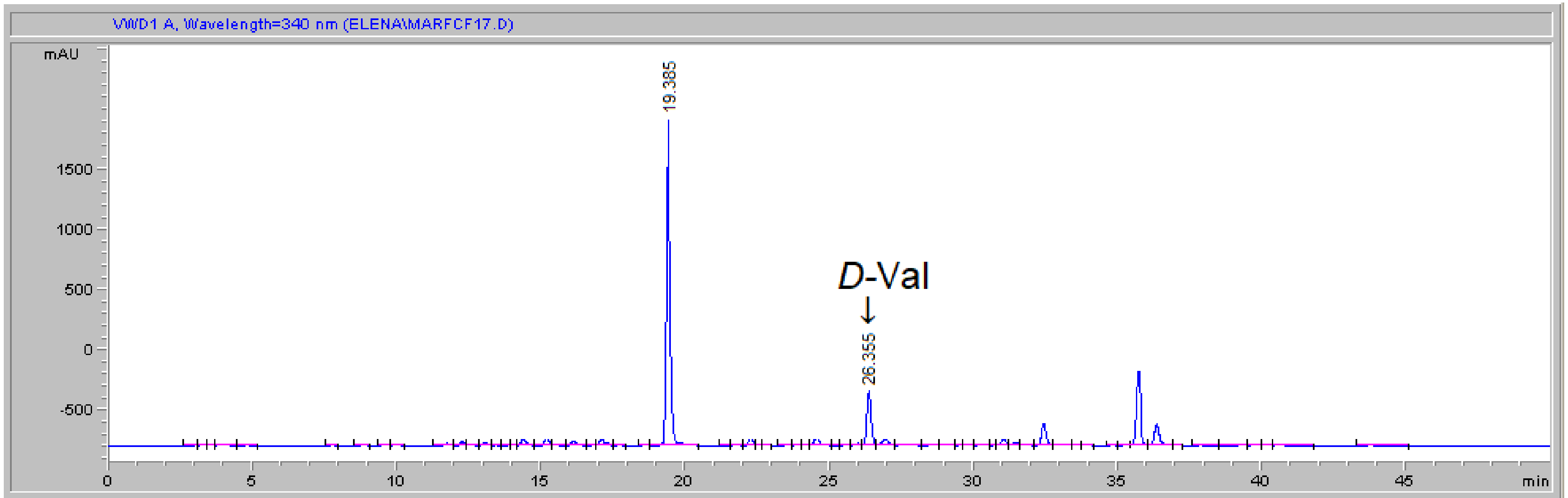

File Information

LC-File	MARFCF17.D
File Path	C:\HPCHEM\1\DATA\ELENA\
Date	07-Oct-22, 11:19:03
Sample	CF-15
Sample Info	
Barcode	
Operator	Kalina
Method	MURFY.M
Analysis Time	49.994 min
Sampling Rate	0.0049 min (0.294 sec), 10302 datapoints

#	Time	Area	Height	Width	Symmetry
42	28.974	131	8.7	0.2145	0.6
43	29.555	63.7	5.4	0.1717	1.374
44	29.801	138.1	11.5	0.1811	0.871
45	30.368	117.2	6.8	0.2497	1.235
46	30.703	20.5	2.2	0.1348	1.831
47	31.009	604.2	49.8	0.1822	0.9
48	31.401	315.8	24.6	0.1962	1.01
49	31.743	167.3	12	0.2028	0.732
50	32.418	2000.1	179.5	0.1708	0.846
51	33.041	270.6	12.2	0.3265	0.892
52	33.646	103.3	8.2	0.1852	1.339
53	33.871	105.9	8.3	0.1902	0.738
54	34.8	43.4	2.9	0.2129	0.884

Figure S55. HPLC profile of *L*-FDAA -derivatives of felicarnezoline E (5) HP +*L*-Val

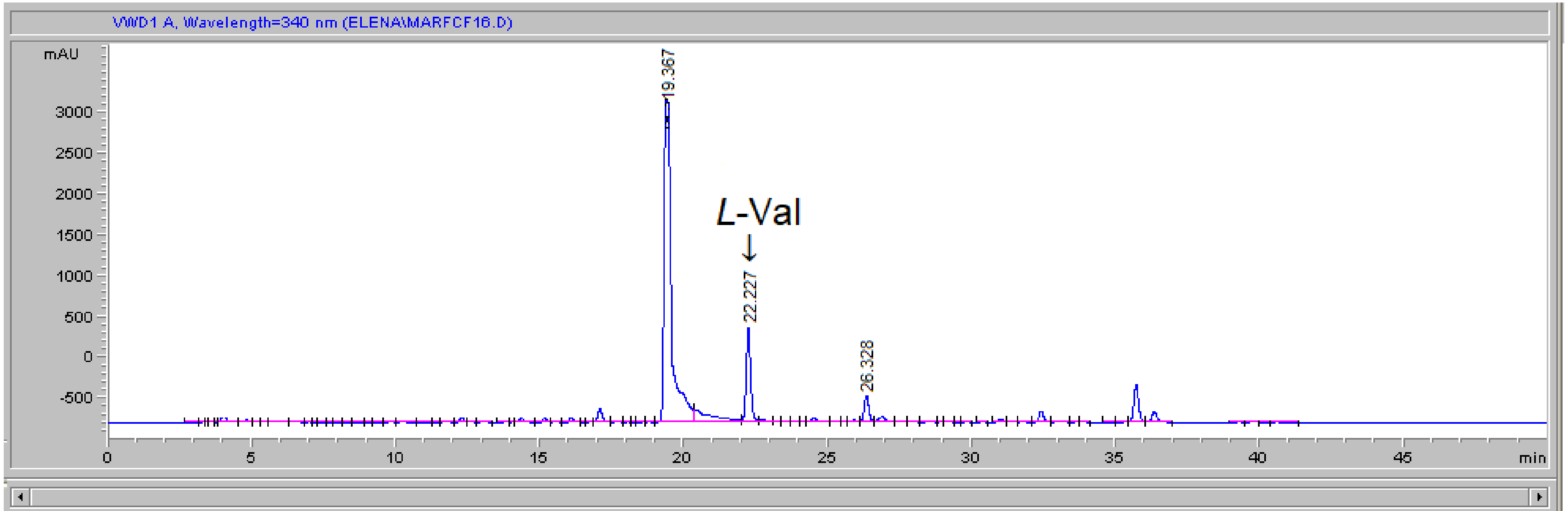

File Information	
LC-File	MARFCF16.D
File Path	C:\HPCHEM\1\DATA\ELENA\
Date	07-Oct-22, 10:28:35
Sample	CF-15 L-Val
Sample Info	
Barcode	
Operator	Kalina
Method	MURFY.M
Analysis Time	49.984 min
Sampling Rate	0.0049 min (0.294 sec), 10300 datapoints

#	Time	Area	Height	Width	Symmetry
42	18.729	67	4.1	0.2151	0.336
43	19.367	74646.4	3971.6	0.2775	0.487
44	20.433	6742.2	158.2	0.5378	0.103
45	22.227	12217.7	1149.4	0.1619	0.849
46	22.721	599	25.7	0.3128	0.337
47	23.172	186.7	14.7	0.1836	0.271
48	23.489	287	14.2	0.2948	0.643
49	23.868	221.5	13.2	0.2486	0.88
50	24.148	181.5	14.1	0.1826	0.98
51	24.502	917	57.9	0.2257	0.742
52	25.412	165.4	7.6	0.2816	5.325
53	25.559	94.9	8	0.1709	0.763
54	25.888	413.3	27.4	0.2223	0.828

Figure S56. HPLC profile of *L*-FDAA -derivatives of felicarnezoline E (5) HP *D,L*-Val

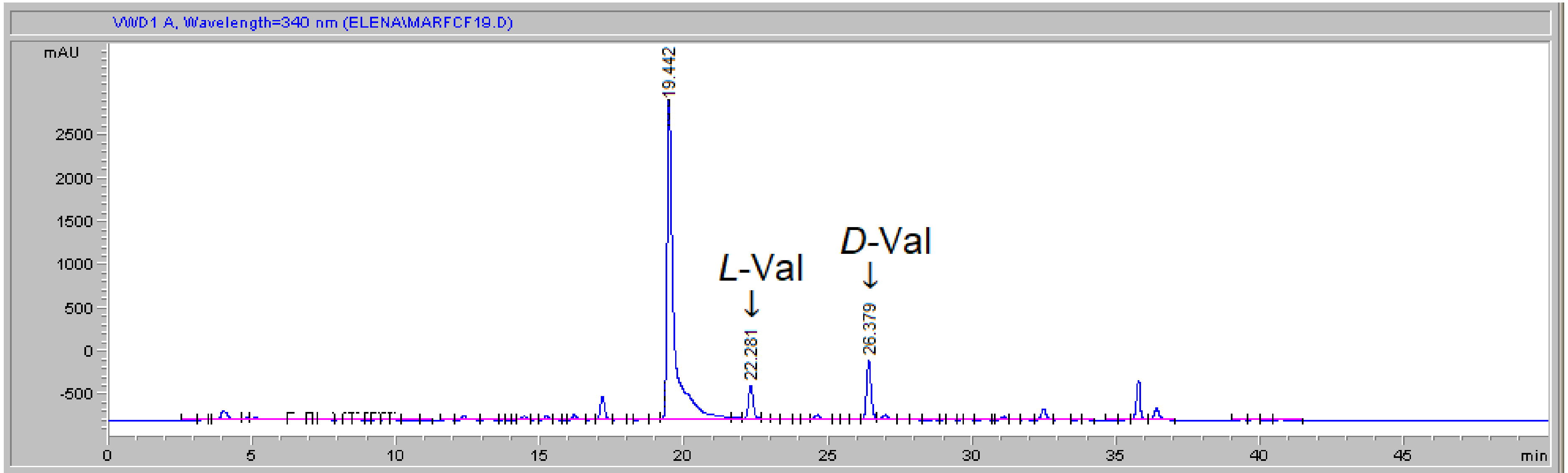

File Information

LC-File	MARFCF19.D
File Path	C:\HPCHEM\1\DATA\ELENA\
Date	07-Oct-22, 13:00:14
Sample	LD-Val CF-17
Sample Info	
Barcode	
Operator	Kalina
Method	MURFY.M
Analysis Time	49.994 min
Sampling Rate	0.0049 min (0.294 sec), 10302 datapoints

#	Time	Area	Height	Width	Symmetry
42	23.03	229.8	13.4	0.2321	0.168
43	23.56	363.9	14.5	0.3364	1.129
44	23.931	186.6	13	0.2023	1.249
45	24.217	274.7	17.5	0.2213	1.211
46	24.564	1063.5	60.4	0.2462	0.596
47	25.256	148.5	9.9	0.2103	0.915
48	25.616	197.5	10.9	0.2457	2.214
49	25.979	407.2	20.5	0.2959	1.403
50	26.379	7438.2	687	0.1671	0.866
51	26.929	1025.3	59	0.2463	1.09
52	27.624	216.9	13	0.2352	1.273
53	27.898	81.9	4.9	0.2659	0.372
54	28.614	78.9	3.3	0.3204	1.284

Figure S57. HPLC profiles of *L*-FDAA -derivatives of *L*-Val (a), *L*-FDAA -derivatives of *D*,*L*-Val (b), *L*-FDAA -derivatives of felicarnezoline E (5) HP (c), felicarnezoline E (5) HP +*D*,*L*-Val (d) and felicarnezoline E (5) HP+*L*-Val (f)

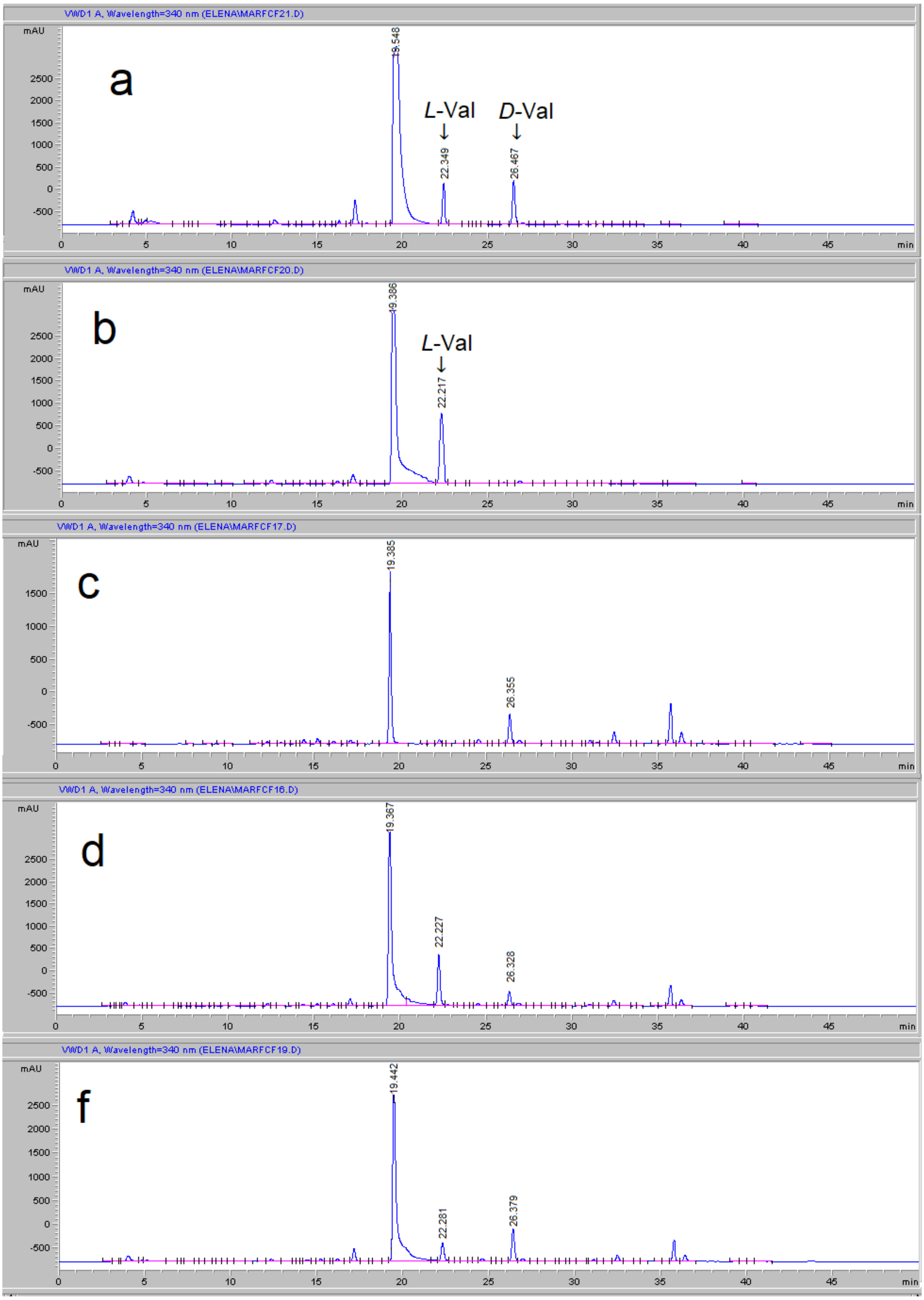

Figure S58. MS spectrum of felicarnezoline A (1)

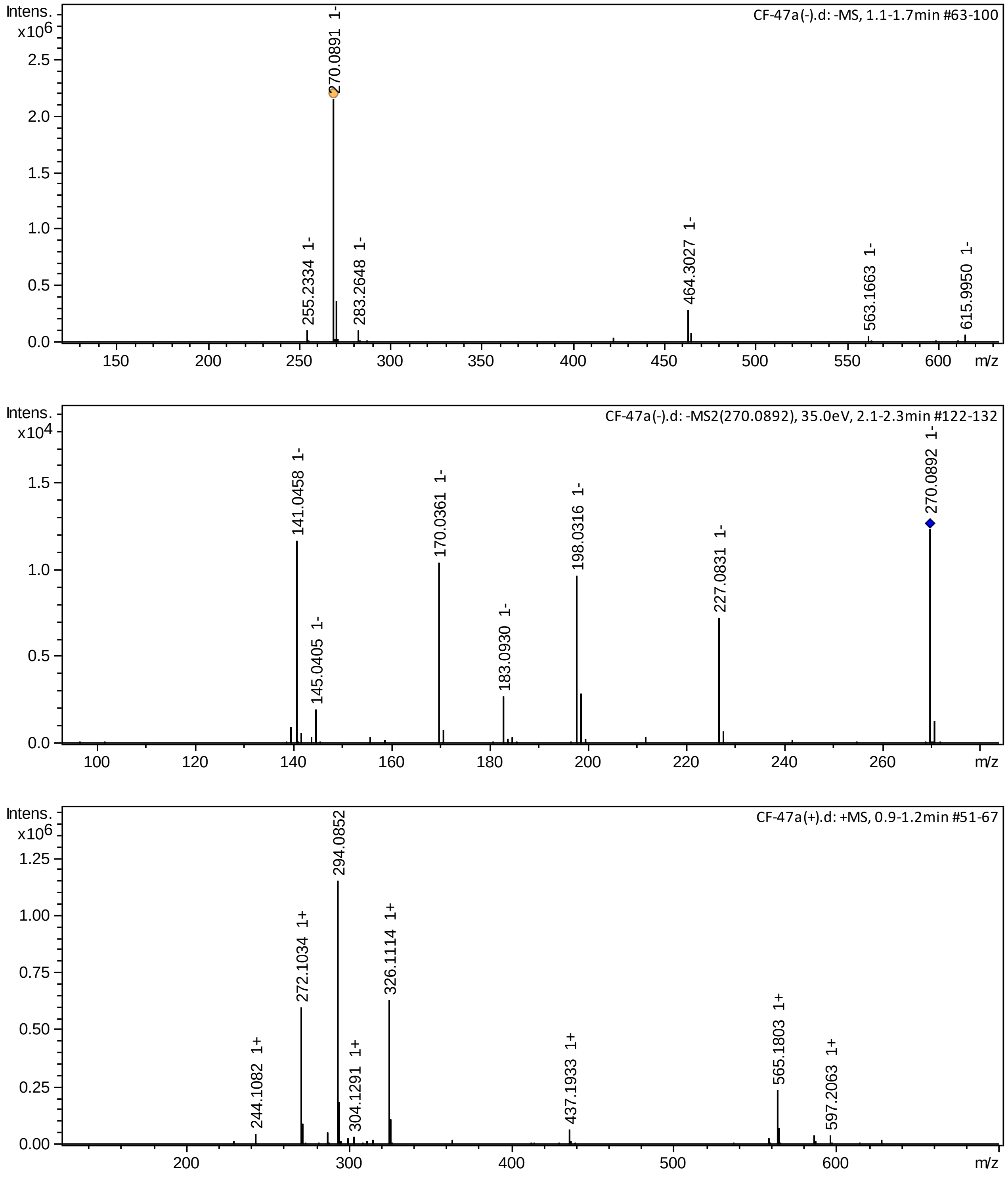

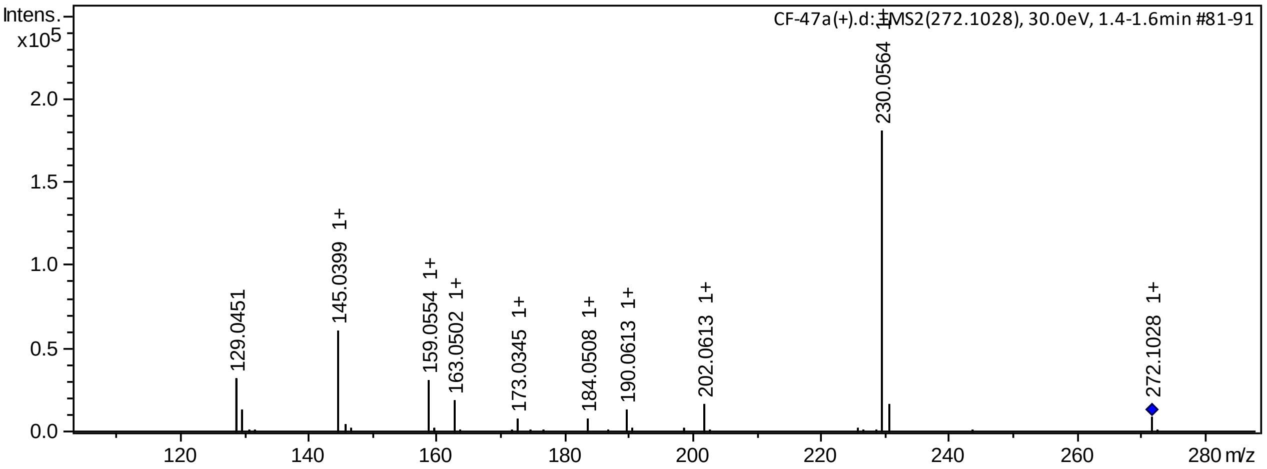

Figure S59. MS spectrum of felicarnezoline B (2)

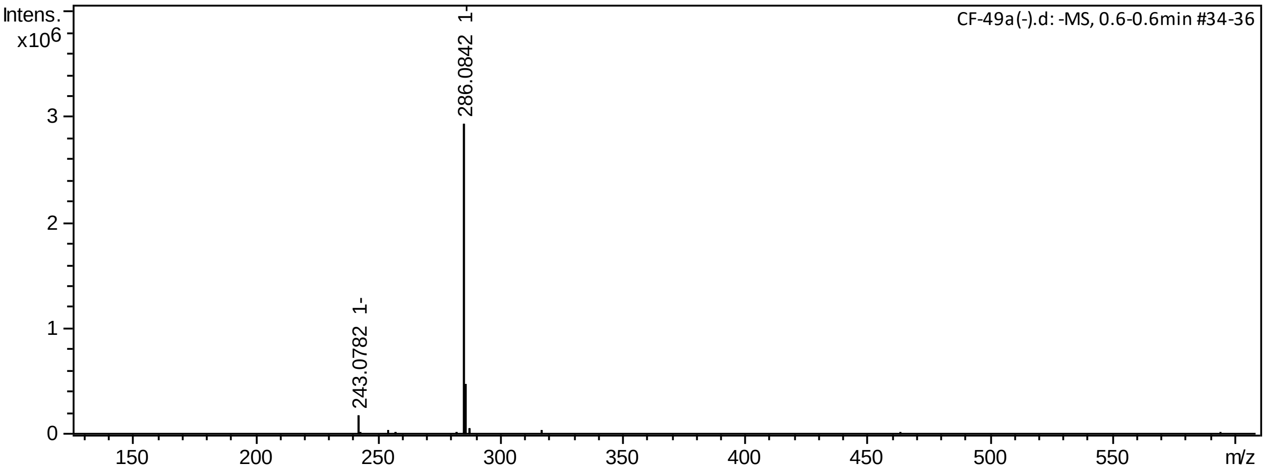

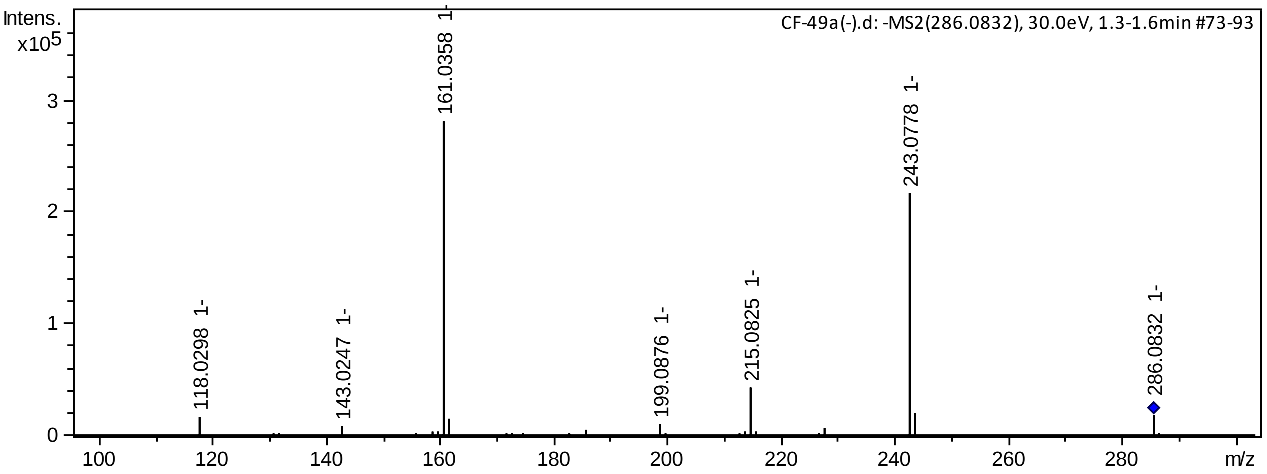

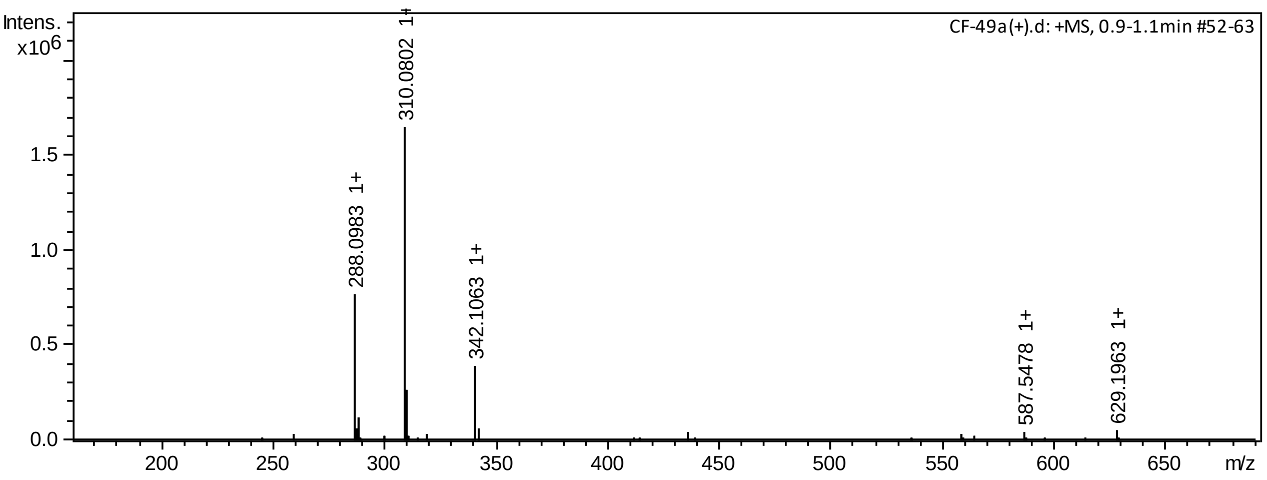

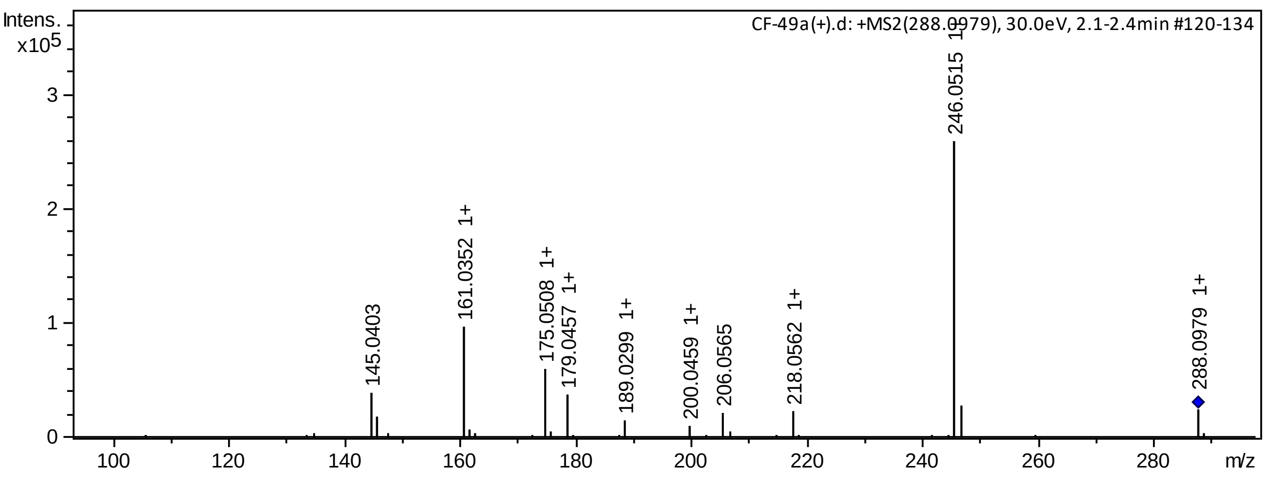

Figure S60. MS spectrum of felicarnezoline D (4)

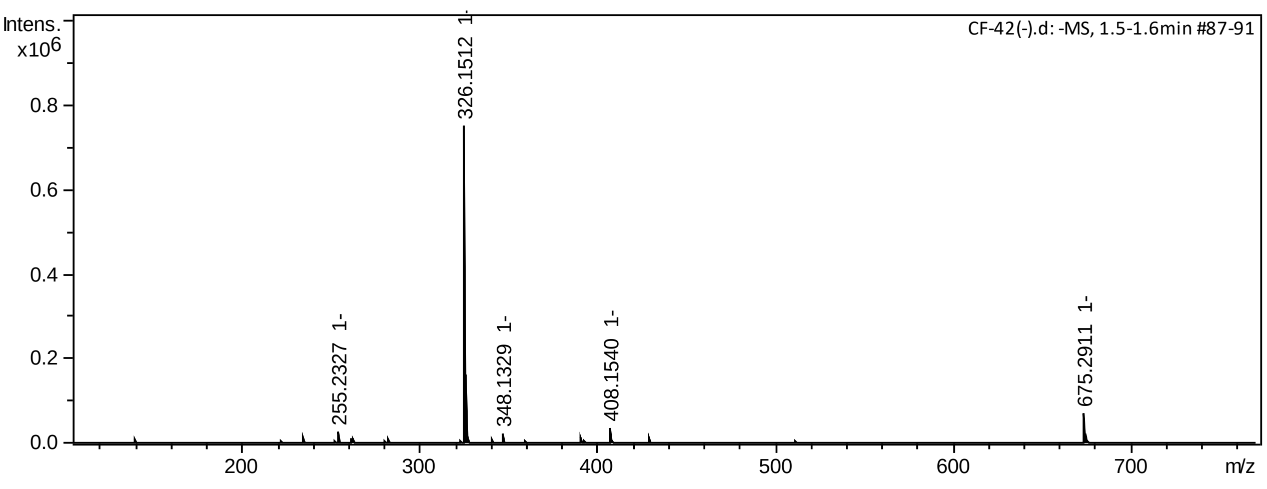

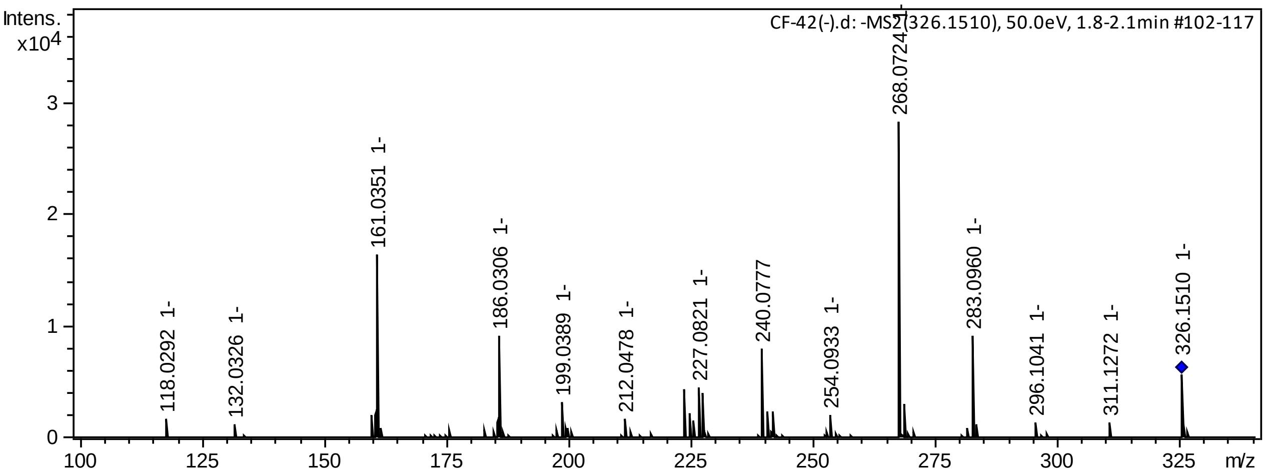
Intens.
x10^4
CF-42(-).d: -MS2(326.1510), 50.0eV, 1.8-2.1min #102-117
118.0292 1-
132.0326 1-
161.0351 1-
186.0306 1-
199.0389 1-
212.0478 1-
227.0821 1-
240.0777
254.0933 1-
268.0724
283.0960 1-
296.1041 1-
311.1272 1-
326.1510 1-
m/z

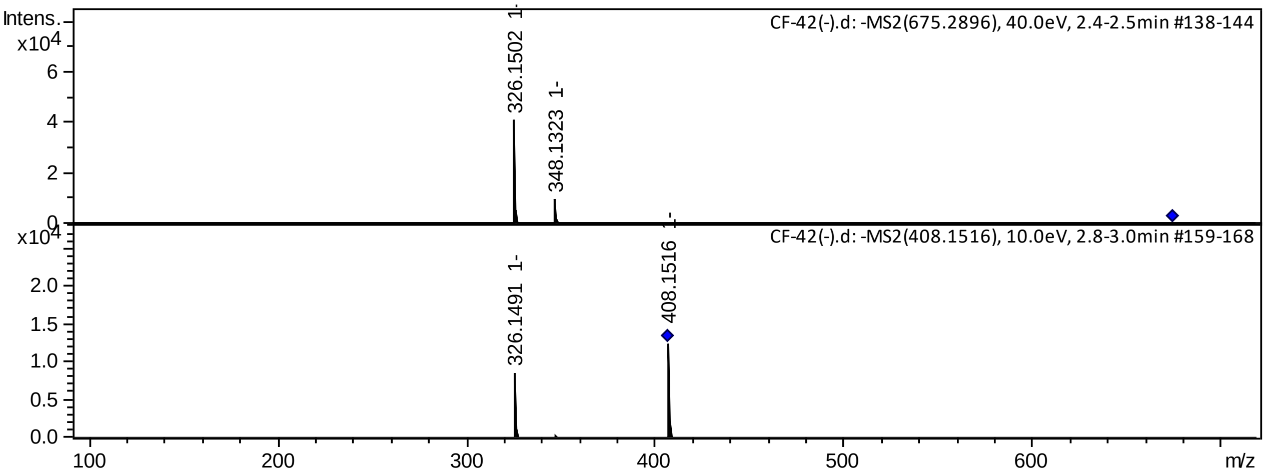
Intens.
x10^4
CF-42(-).d: -MS2(675.2896), 40.0eV, 2.4-2.5min #138-144
326.1502 1-
348.1323 1-
x10^4
CF-42(-).d: -MS2(408.1516), 10.0eV, 2.8-3.0min #159-168
326.1491 1-
408.1516
m/z

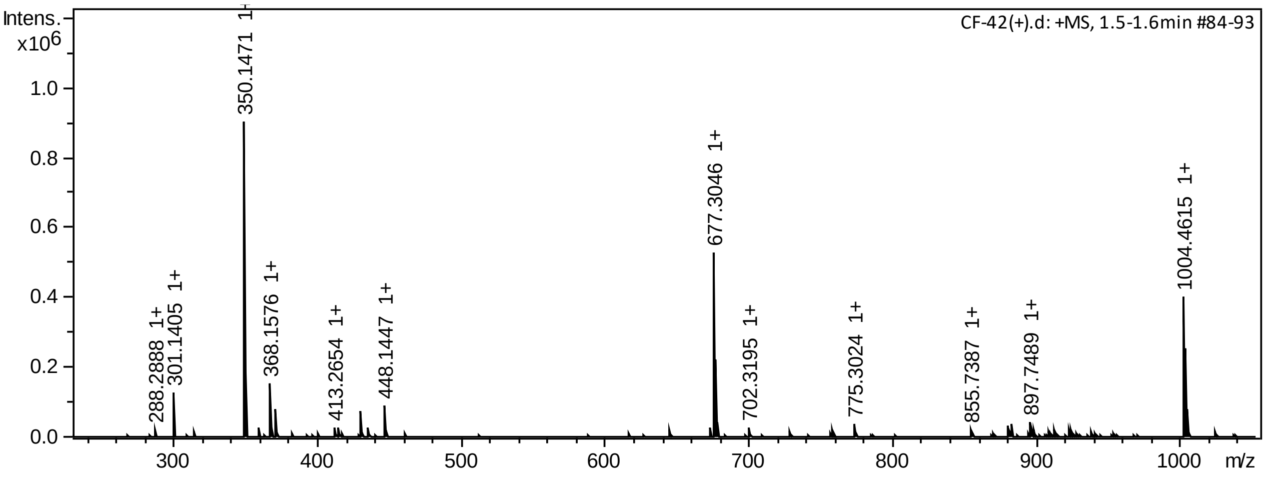
Intens.
x10^6
CF-42(+).d: +MS, 1.5-1.6min #84-93
288.2888 1+
301.1405 1+
350.1471 1+
368.1576 1+
413.2654 1+
448.1447 1+
677.3046 1+
702.3195 1+
775.3024 1+
855.7387 1+
897.7489 1+
1004.4615 1+
m/z

Figure S61. MS spectrum of oxyrapentin M (6)

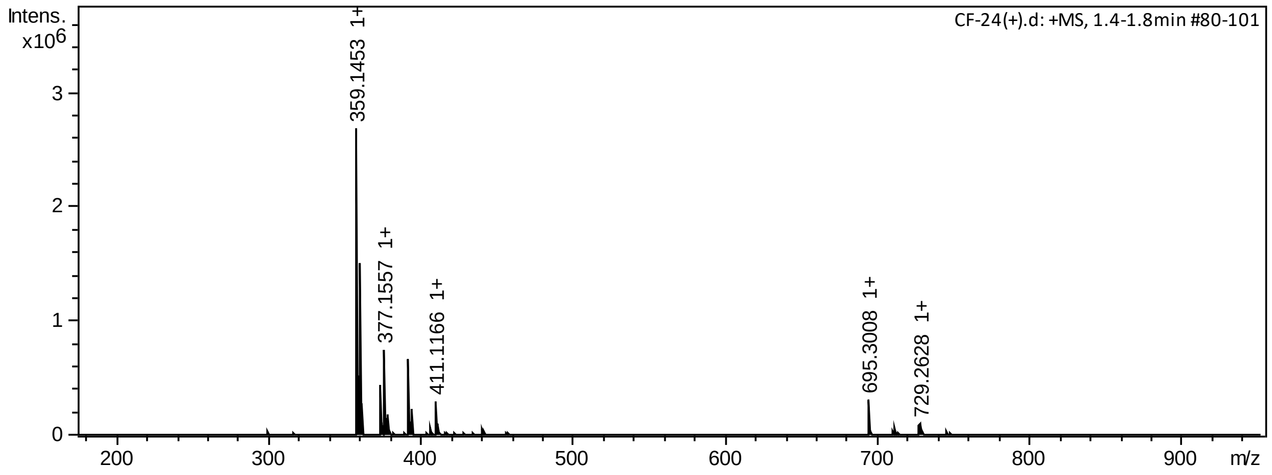

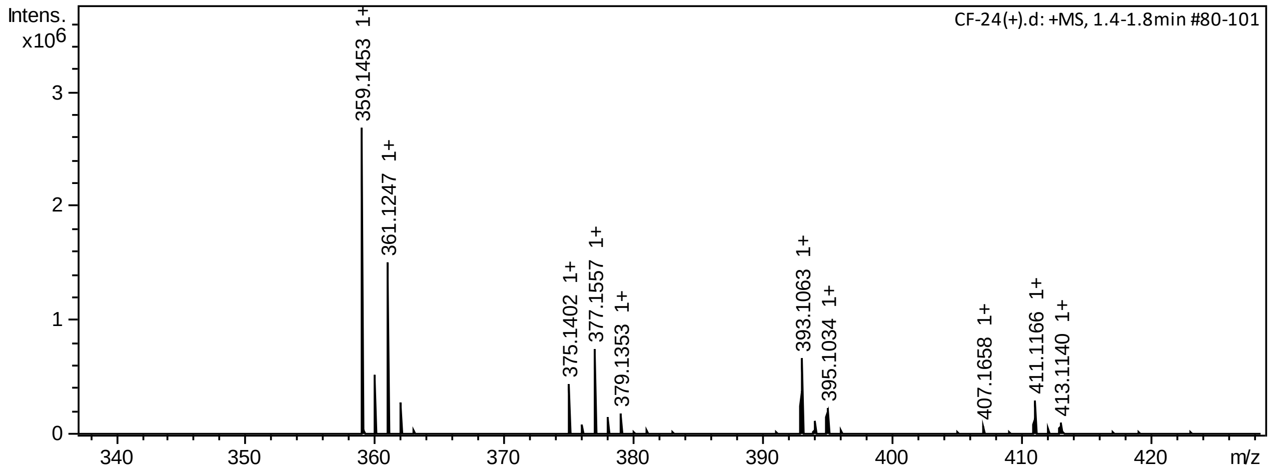

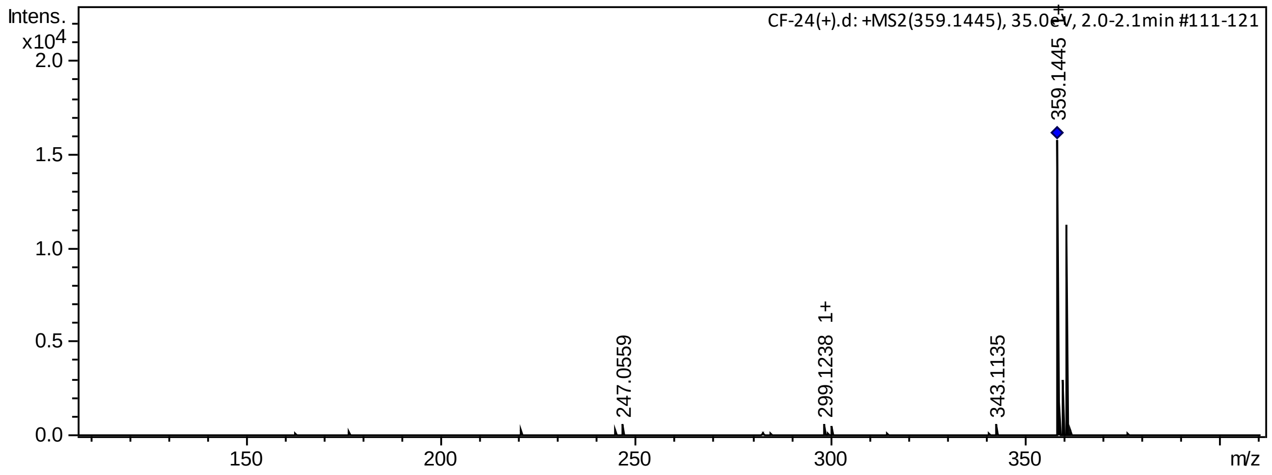

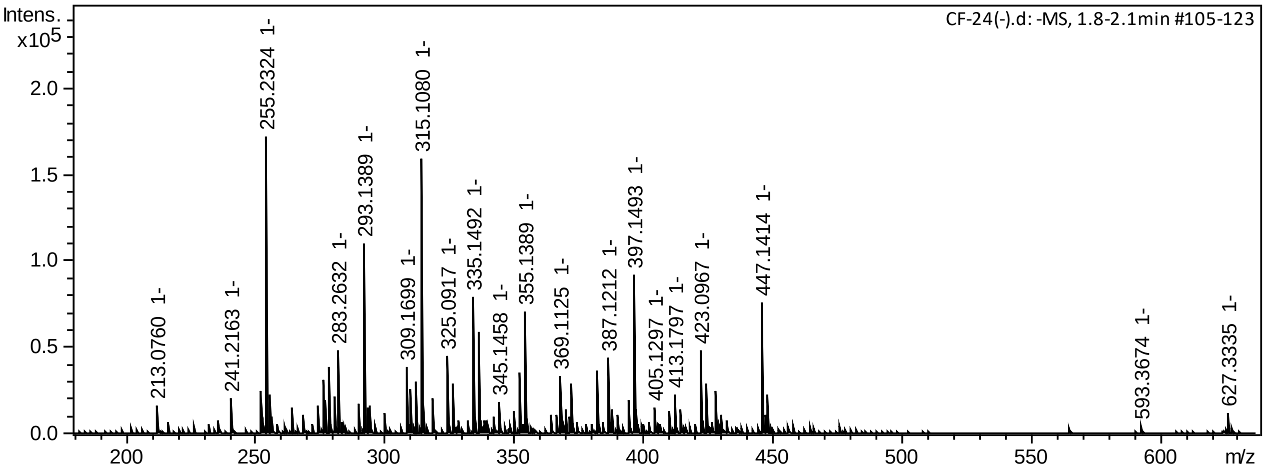

Intens.
x10^5
2.0
1.5
1.0
0.5
0.0
CF-24(-).d: -MS, 1.8-2.1min #105-123
213.0760 1-
241.2163 1-
255.2324 1-
283.2632 1-
293.1389 1-
309.1699 1-
315.1080 1-
325.0917 1-
335.1492 1-
345.1458 1-
355.1389 1-
369.1125 1-
387.1212 1-
397.1493 1-
405.1297 1-
413.1797 1-
423.0967 1-
447.1414 1-
593.3674 1-
627.3335 1-
200
250
300
350
400
450
500
550
600
m/z

Figure S62. CD spectrum of felicarnezoline A (1)

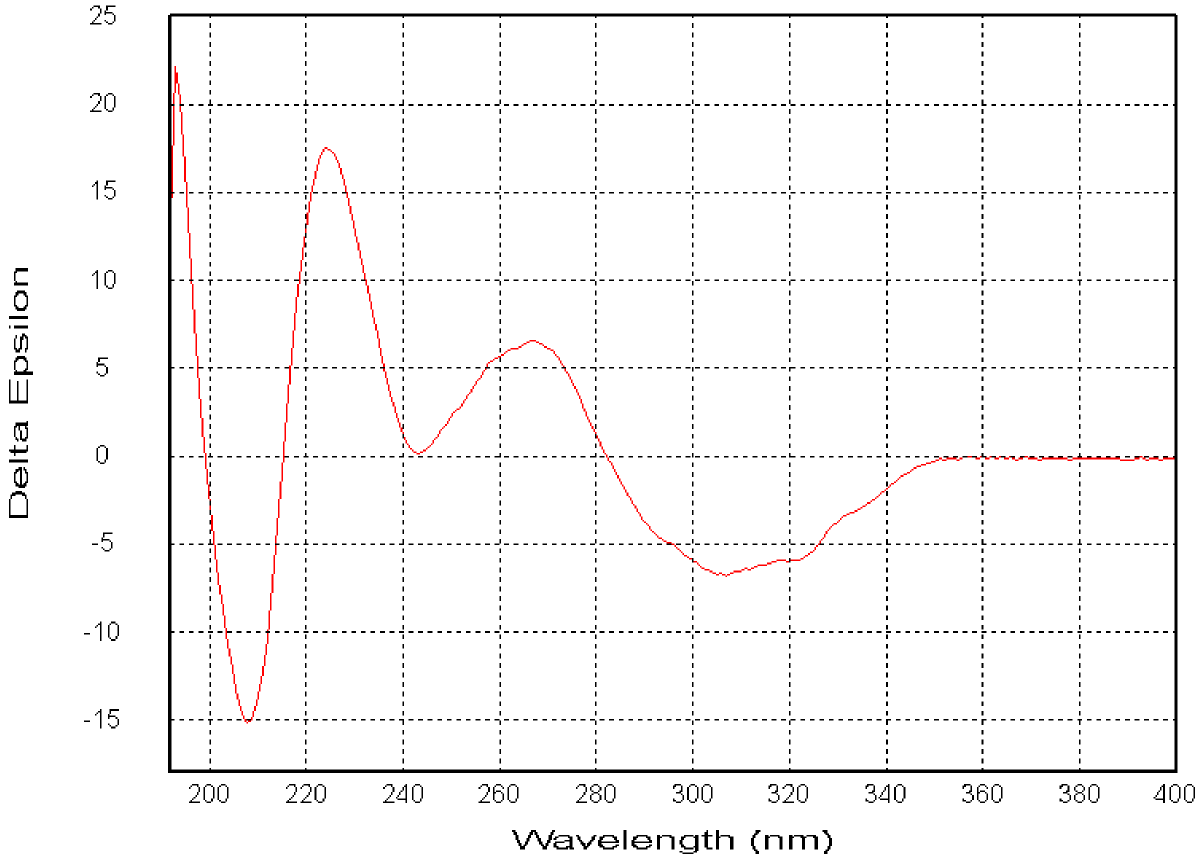

Figure S63. UV spectrum of felicarnezoline A (1)

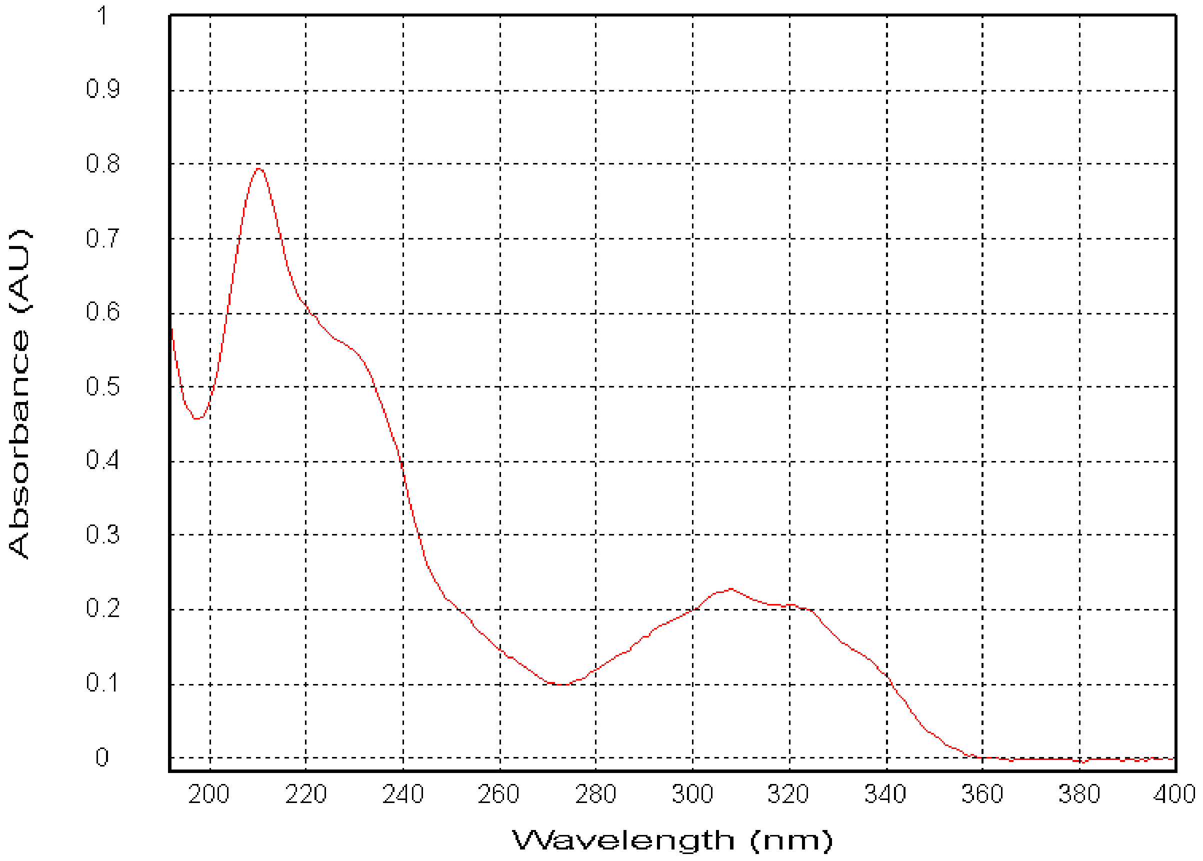

Figure S64. CD spectrum of felicarnezoline B (2)

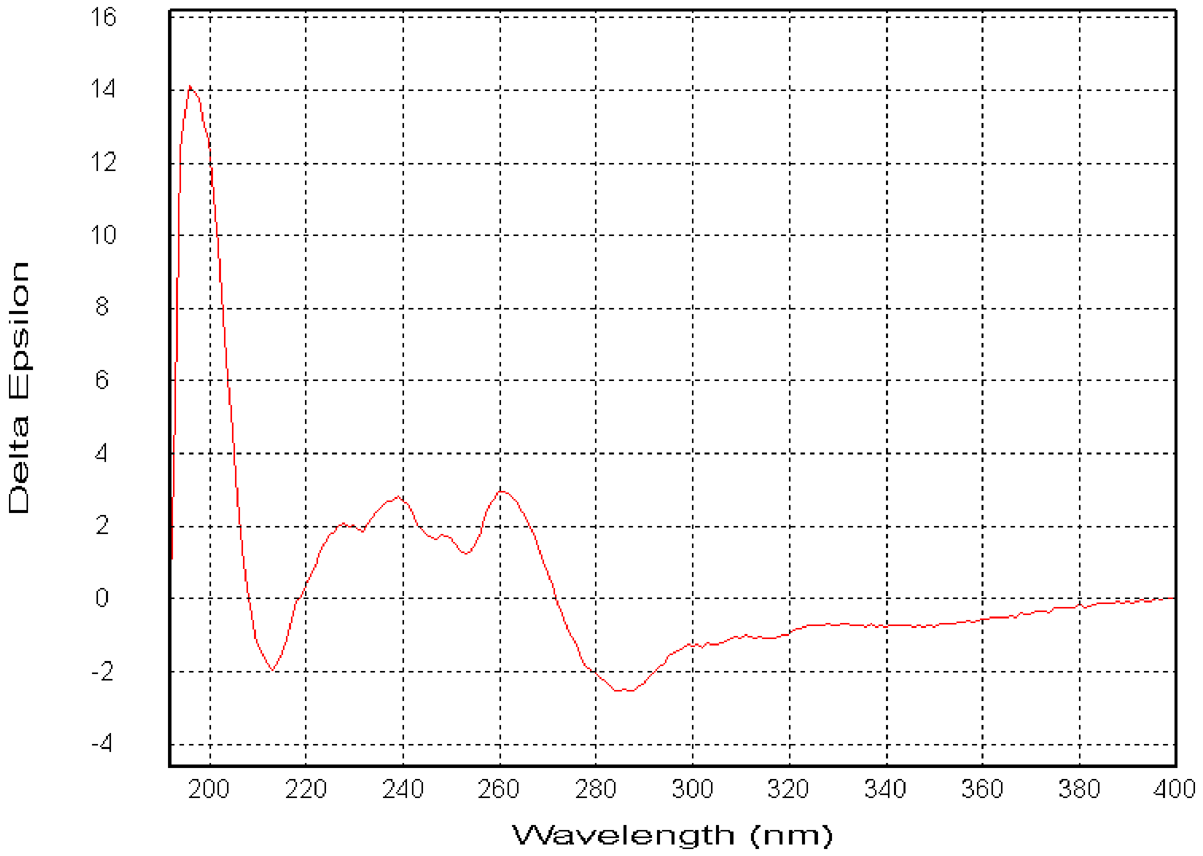

Figure S65. UV spectrum of felicarnezoline B (2)

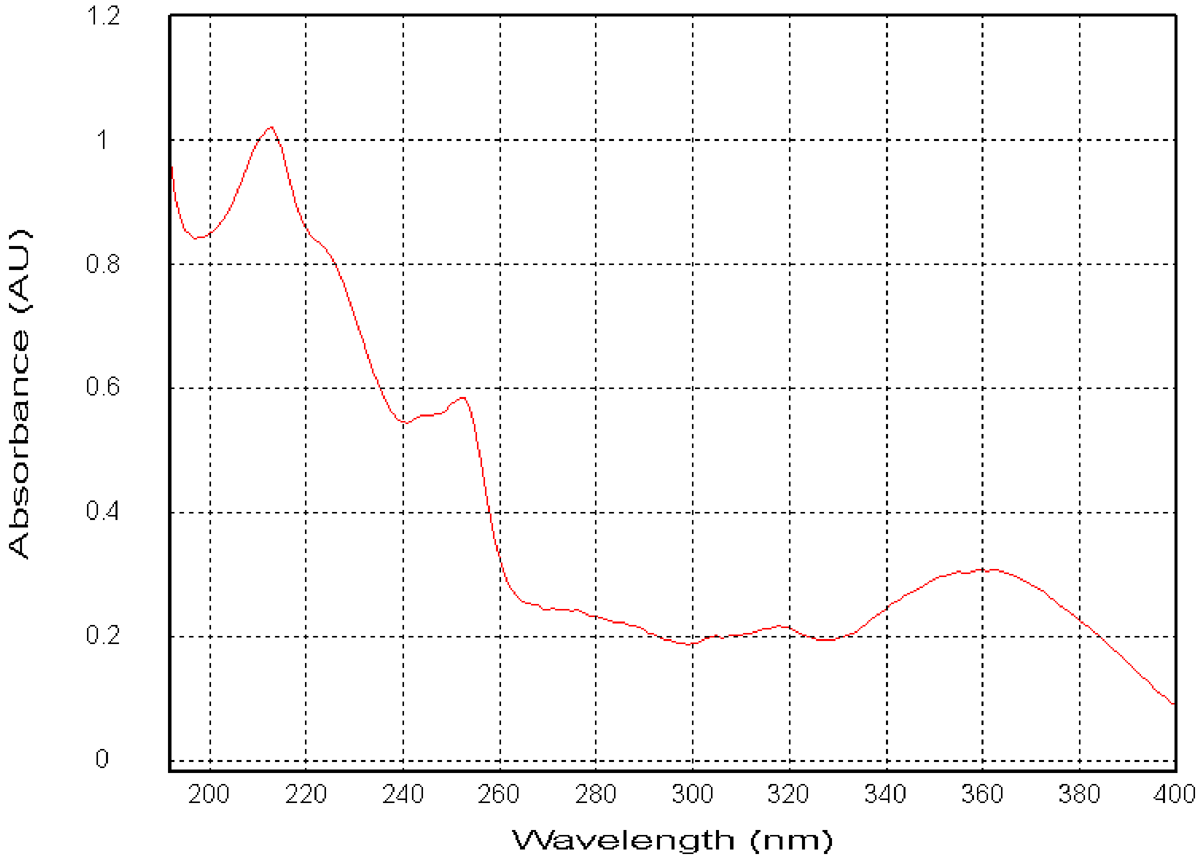

Figure S66. CD spectrum of felicarnezoline D (4)

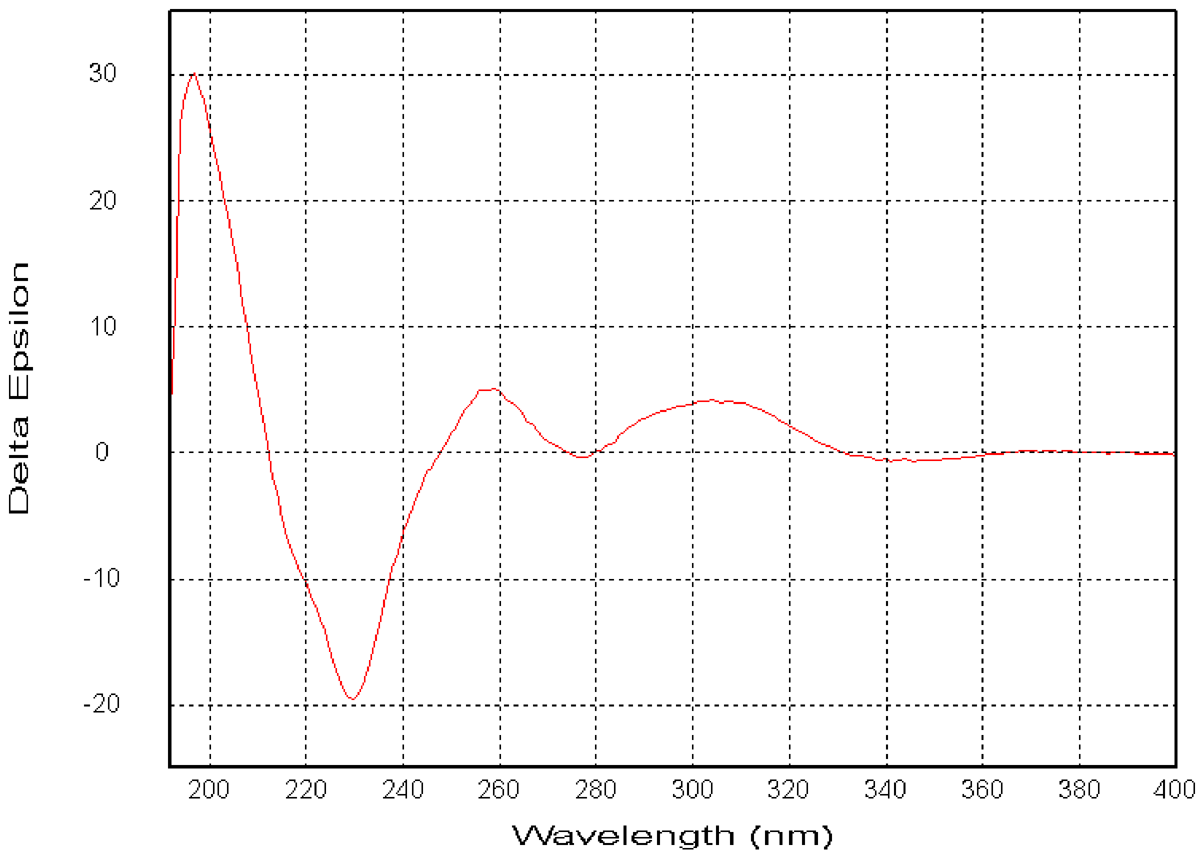

Figure S67. UV spectrum of felicarnezoline D (4)

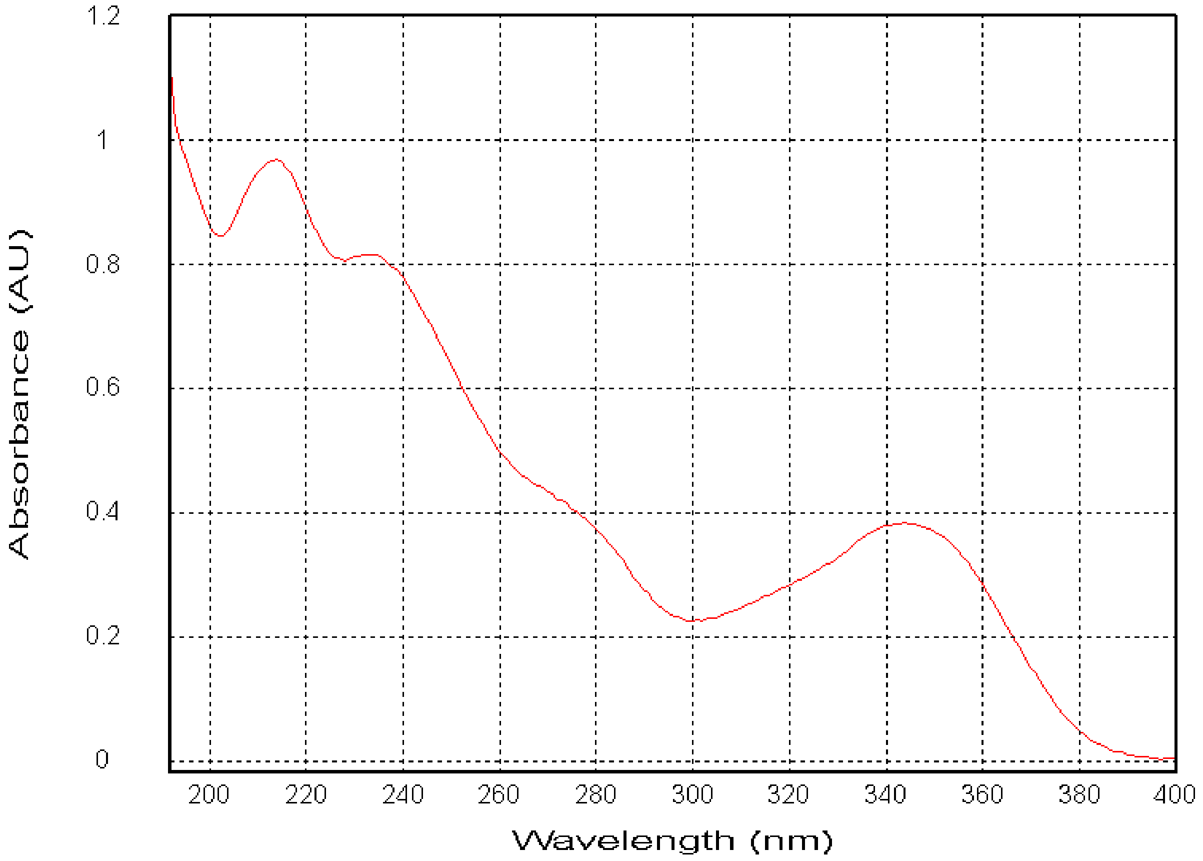

Figure S68. CD spectrum of oxyrapentin M (6)

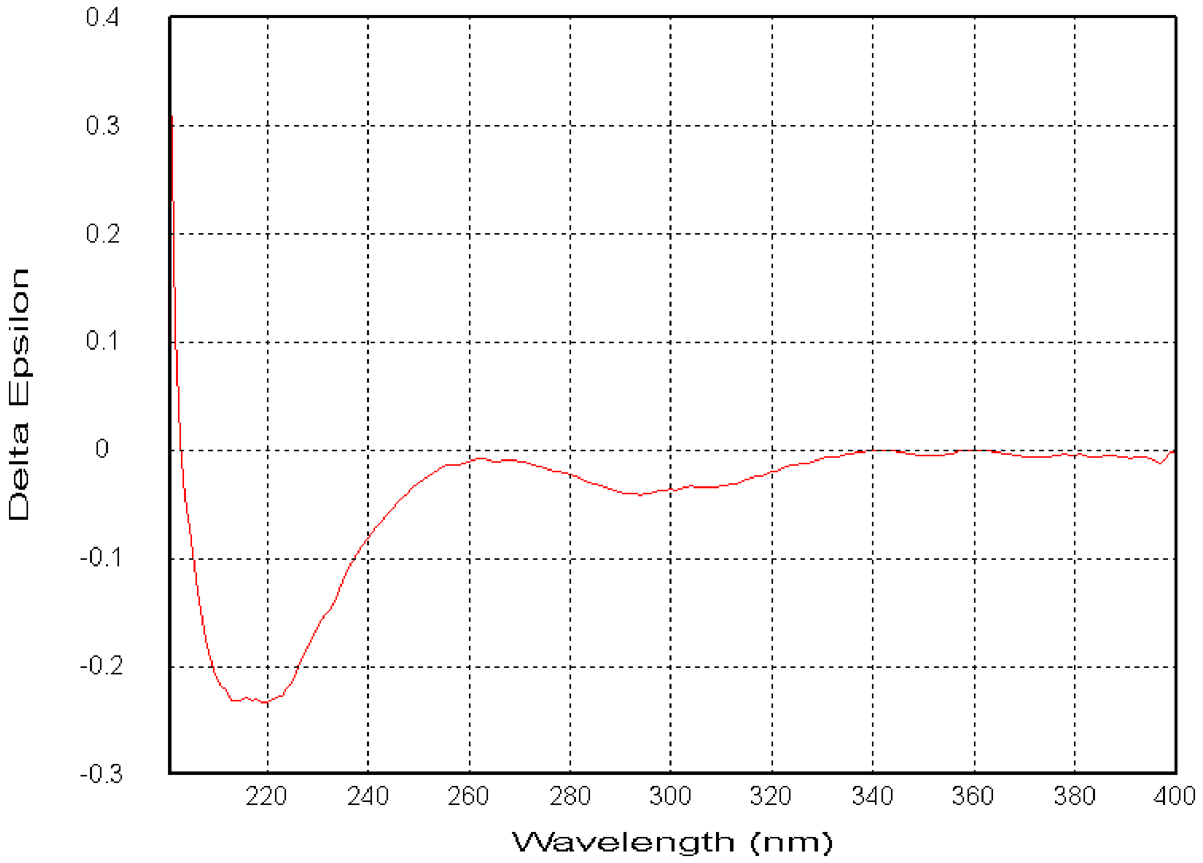

Figure S69. UV spectrum of oxyrapentin M (6)

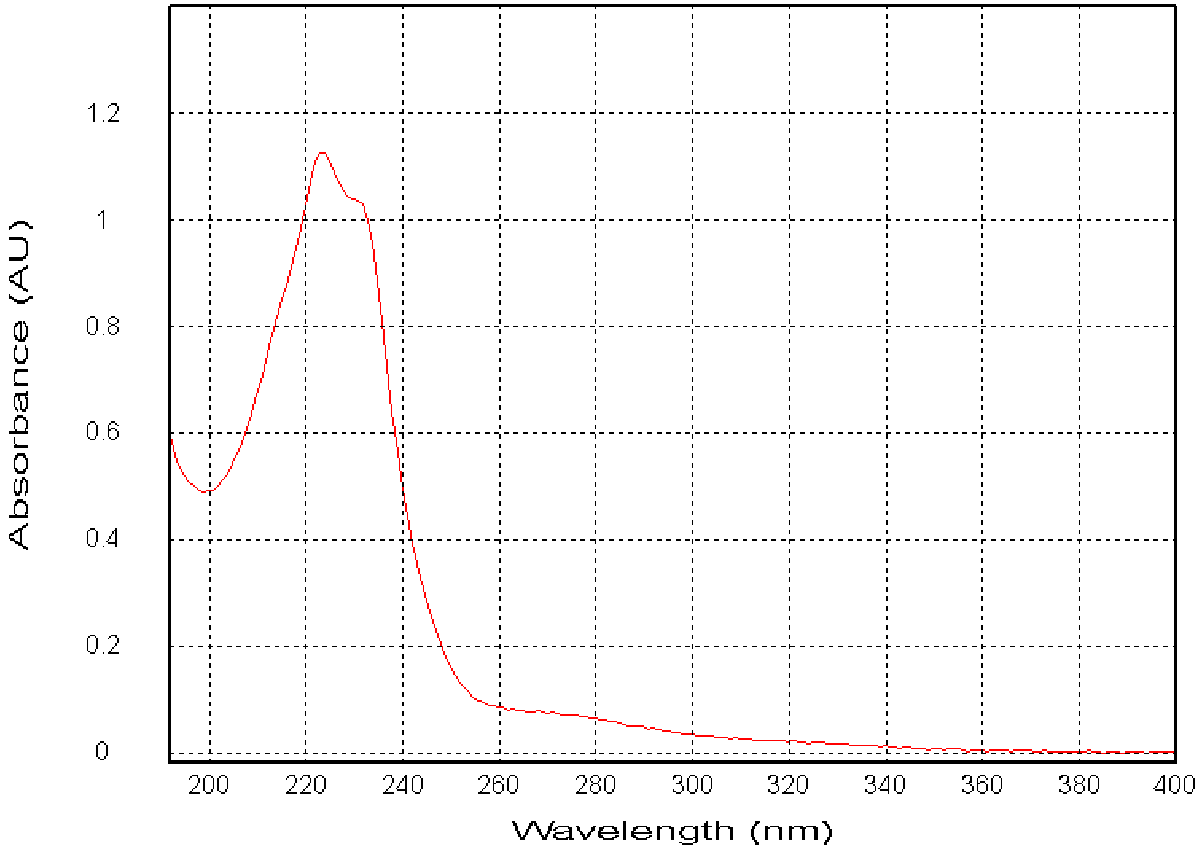

Table S1. ^{1}H NMR data for carnequinazoline A [1] and felicarnezoline C (**3**).

Position	**Carnequinazoline A** [a] δH, mult, *J* in Hz	**Felicarnezoline C (3)** [b] δH, mult, *J* in Hz
1	-	-
2 (NH)	7.55, s	8.00, brs
3	-	-
4	-	-
5	-	-
6	-	-
7	7.70, d (8.12)	7.70, brs
8	7.77, t (7.7)	7.82, t (7.6)
9	7.48, t (7.5)	7.54, t (7.5)
10	8.28, dd (8.0, 1.0)	8.29, dd (7.9, 1.2)
11	-	-
12	-	-
13	-	-
14	5.46, d (5.4)	5.42, d (5.5)
15	2.28, m	2.28, m
16	0.97, d (6.7)	0.98, d (6.8)
17	1.14, d (6.7)	1.14, d (6.8)
18	6.44, d (10.0)	6.75, d (9.5)
19	2.61, m	2.66, m
20	1.20, d (6.7)	1.25, d (6.5)
21	1.17, d (6.7)	1.18, d (6.6)

[a] chemical shifts were measured in $CDCl_3$ at 700.13 MHz.
[b] chemical shifts were measured in $CDCl_3$ at 300.13 MHz.

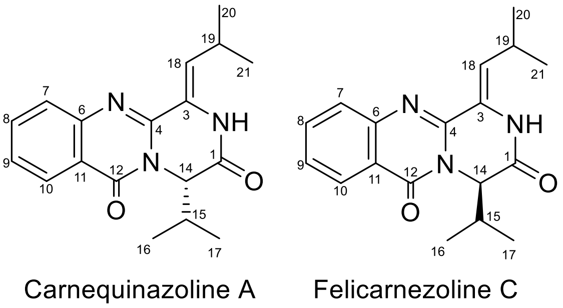

1. Zhuravleva, O.I.; Afiyatullov, S.S.; Denisenko, V.A.; Ermakova, S.P.; Slinkina, N.N.; Dmitrenok, P.S.; Kim, N.Y. Secondary metabolites from a marine-derived fungus *Aspergillus carneus* Blochwitz. *Phytochemistry* **2012**, *80*, 123-131.

Table S2. ^{1}H and ^{13}C NMR data ($CDCl_3$, 700 MHz) for carnequinazoline B [1] and felicarnezoline D (**4**).

Position	**carnequinazoline B** δ_H, mult, *J* in Hz	**felicarnezoline D (4)** δ_H, mult, *J* in Hz
1	-	-
2 (NH)	8.29 s	8.48, brs
3	-	-
4	-	-
5	-	-
6	-	-
7	-	-
8	7.29, dd (7.9, 1.3)	7.29, d (7.8)
9	7.37, t (7.9)	7.37, t (7.9)
10	7.75, dd (7.9, 1.2)	7.74, brd (8.0)
11	-	-
12	-	-
13	-	-
14	5.41, d (5.4)	5.40, brd (5.4)
15	2.28, m	2.28, m
16	0.97, d (7.0)	0.97, d (6.5)
17	1.14, d (7.0)	1.13, d (6.6)
18	6.37, d (10.0)	6.39, d (10.0)
19	2.70, m	2.74, m
20	1.21, d (6.7)	1.21, d (6.7)
21	1.17, d (6.7)	1.16, d (6.6)

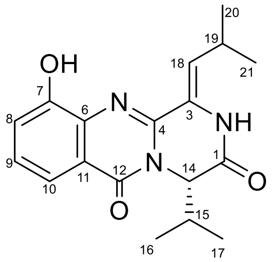

Carnequinazoline B

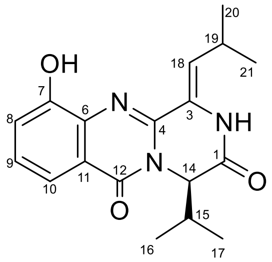

Felicarnezoline D

1. Zhuravleva, O.I.; Afiyatullov, S.S.; Denisenko, V.A.; Ermakova, S.P.; Slinkina, N.N.; Dmitrenok, P.S.; Kim, N.Y. Secondary metabolites from a marine-derived fungus *Aspergillus carneus* Blochwitz. *Phytochemistry* **2012**, *80*, 123-131.

Table S3. ^{1}H and ^{13}C NMR data ($CDCl_3$, 500 MHz) for dihydrocinereain [1] and compound felicarnezoline E (**5**).

Position	**Dihydrocinereain**<br>δ_H, mult, *J* in Hz	**Felicarnezoline E (5)**<br>δ_H, mult, *J* in Hz
1	-	-
2 (NH)	4.55, dd (9.9, 3.3)	4.55, dd (10.0, 3.3)
3	-	-
4	-	-
5	-	-
6	-	-
7	-	-
8	6.06, d (5.6)	6.07, d (5.6)
9	5.62, t (5.6)	5.62, t (5.6)
10	6.15, dd (11.1, 5.6)	6.16, dd (11.1, 5.7)
11	6.73, d (11.1)	6.73, d (11.1)
12	-	-
13	-	-
14	-	-
15	5.17, d (6.4)	5.17, d (7.0)
16	2.28, m	2.27, m
17	1.09, d (7.0)	1.10, d (6.7)
18	1.11, d (7.0)	1.11, d (6.8)
19	1.69, m	1.69, dd (14.3, 9.9, 4.1)
	2.29, m	2.30, m
20	1.76, m	1.78, m
21	1.04, d (6.5)	1.04, d (6.5)
22	1.00, d (6.5)	1.00, d (6.6)

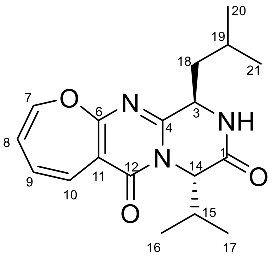

Dihydrocinereain

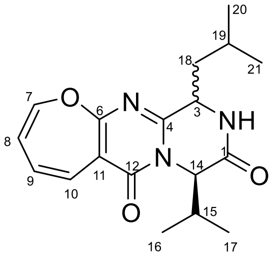

Felicarnezoline E (**5**)

1. Zhuravleva, O.I.; Afiyatullov, S.S.; Yurchenko, E.A.; Denisenko, V.A.; Kirichuk, N.N.; Dmitrenok, P.S. New Metabolites from the Algal Associated Marine-derived Fungus *Aspergillus carneus*. *Natural Product Communications* **2013**, *8*, 1071-1074.
